# Cys–Lys stapling for unprotected peptides via tunable linkers

**DOI:** 10.1093/nsr/nwaf406

**Published:** 2025-09-22

**Authors:** Kaizhen Miao, Bei Fu, Leiyang Bai, Chengliang Li, Xuefeng Jiang

**Affiliations:** Hainan Institute of East China Normal University, Shanghai Key Laboratory of Green Chemistry and Chemical Processes, State Key Laboratory of Petroleum Molecular & Process Engineering, School of Chemistry and Molecular Engineering, East China Normal University, Shanghai 200062, China; Hainan Institute of East China Normal University, Shanghai Key Laboratory of Green Chemistry and Chemical Processes, State Key Laboratory of Petroleum Molecular & Process Engineering, School of Chemistry and Molecular Engineering, East China Normal University, Shanghai 200062, China; Hainan Institute of East China Normal University, Shanghai Key Laboratory of Green Chemistry and Chemical Processes, State Key Laboratory of Petroleum Molecular & Process Engineering, School of Chemistry and Molecular Engineering, East China Normal University, Shanghai 200062, China; Hainan Institute of East China Normal University, Shanghai Key Laboratory of Green Chemistry and Chemical Processes, State Key Laboratory of Petroleum Molecular & Process Engineering, School of Chemistry and Molecular Engineering, East China Normal University, Shanghai 200062, China; Hainan Institute of East China Normal University, Shanghai Key Laboratory of Green Chemistry and Chemical Processes, State Key Laboratory of Petroleum Molecular & Process Engineering, School of Chemistry and Molecular Engineering, East China Normal University, Shanghai 200062, China; School of Chemistry and Chemical Engineering, Henan Normal University, Xinxiang 453007, China; State Key Laboratory of Organometallic Chemistry, Shanghai Institute of Organic Chemistry, Chinese Academy of Sciences, Shanghai 200032, China

**Keywords:** Cys–Lys stapling, unprotected peptides, tunable linkers, cyclic peptides, chemoselectivity, site selectivity

## Abstract

Stapling has emerged as a transformative paradigm in peptide chemistry, enabling precise conformational control to endow peptides with augmented biophysical properties, including enhanced proteolytic stability and target-binding affinity. Although symmetric macrocyclization strategies have been investigated, non-symmetric stapling of native peptide scaffolds remains underexplored, owing to intricate synthetic challenges associated with achieving concurrent chemoselectivity and site selectivity. This limitation primarily stems from the requirement for orthogonal reactivity in modifying distinct proteinogenic residues while preserving native side-chain functionalities under biocompatible conditions. Herein, a non-symmetric stapling is disclosed for cysteine–lysine (Cys–Lys) crosslinking in unprotected peptides and proteins via unsymmetrically tunable linkers with high chemoselectivity and regioselectivity in a self-assembly manner, in which a library of 17 stapling reagents with adjustable length, angle, flexibility, rigidity and lipophilicity was comprehensively established for relay Cys–Lys ligation, facilitating the macrocyclization of intervening loops (6–30 amino acids) into 25–40-membered rings under physiologically compatible conditions. The conformationally restricted peptide displayed strengthened *α*-helicity, proteolytic stability, serum stability and enhanced anti-bladder cancer activity, demonstrating the potential of this protocol for drug discovery.

## INTRODUCTION

**Figure 1.**
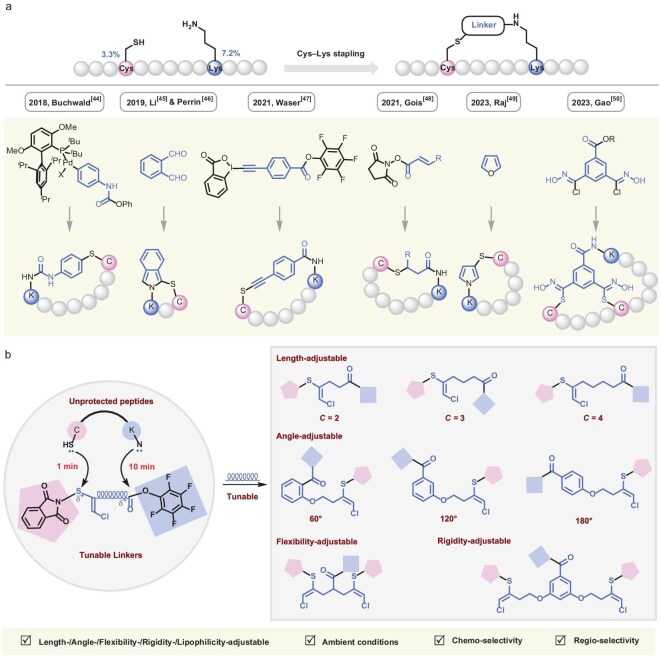
Non-symmetric Cys–Lys stapling. (a) Previous strategies. (b) This work: Cys–Lys relay stapling in one step for unprotected peptides via tunable linkers.

Peptides have emerged as pharmacologically potent agents characterized by exquisite target specificity and enhanced safety profiles compared to conventional small-molecule therapeutics, driving their escalating clinical adoption across diverse therapeutic domains [1,2]. As of 2020, over 100 peptide-based compounds have received regulatory approval for therapeutic or diagnostic applications, underscoring their translational potential [3]. However, peptides are facing three major pharmacological challenges: (i) strong susceptibility to proteolytic degradation *in vivo*; (ii) poor membrane permeability; and (iii) low solubility in aqueous solutions, severely limiting their therapeutic efficacy [4,5]. Peptide stapling has proven ability for transforming linear peptides into conformationally constrained peptides, enhancing multiple properties of peptides (*α*-helix conformation, cell penetration, proteolytic stability, target selectivity, aqueous solubility, etc.) for drug development [6–8]. Building upon the seminal work of Blackwell and Grubbs [9], Verdine and co-workers [10,11] first introduced the term ‘stapled peptides,’ which has inspired multifaceted endeavors in peptide and protein stapling [12–15]. The stapling was typically mediated by symmetric linkers that crosslinked two identical native residues within the peptide sequence, thereby ensuring stereochemical uniformity and avoiding regioisomers. Representative examples have included the cysteine–cysteine (Cys–Cys) sulfhydryl stapling, [16–29] lysine–lysine (Lys–Lys) *N*-terminus stapling [23,30–33] and tyrosine–tyrosine (Tyr–Tyr) stapling [34,35] of native peptides. Unlike symmetric stapling, its non-symmetric counterpart relies on unsymmetric crosslinkers to conjugate two different native residues within a peptide sequence, carrying multidimensional topologies and functionalities that are in high demand in peptide drug discovery [36–38]. However, non-symmetric locking is confronted with formidable challenges: (i) two residue-specific modifications lead to much more complexity; (ii) peptides bearing multiple identical residues demand higher orthogonality; (iii) simultaneously modifying distinct proteinogenic residues requires higher chemoselectivity and site selectivity; (iv) designing unsymmetric linkers needs to balance the reactivities and compatibilities for two terminals; and (v) undesired side pathways prove competitive, chiefly among peptide oligomerization, degradation of the staple itself, and the generation of regioisomeric products. The strong nucleophilicity of their side chains (thiol in cysteine and amine in lysine), coupled with their distinct natural abundances (3.3% and 7.2%, respectively), makes cysteine (Cys, C) and lysine (Lys, K) predominant targets for chemospecific and site-specific protein modification [39–42]. The challenging non-symmetric Cys–Lys sulfhydryl/*N*-terminus stapling has been achieved via combining Cys arylation with Lys amidation by Kubota *et al*. [43], via *o*-phthalaldehyde-mediated intermolecular crosslinking of Cys and Lys by Zhang *et al*. [44] and Todorovic *et al*. [45], respectively, via hypervalent iodine reagents for Cys initiation and pentafluorophenyl (PFP) ester for Lys handles by Ceballos *et al*. [46], via *N*-hydroxysuccinimide-activated acrylic ester for Cys bioconjugation and Lys activation by Silva *et al*. [47], via a bis-aldehyde species generated *in situ* through furan oxidation by Wang *et al*. [48], via leveraging bis-chlorooxime–Cys bioconjugation and Lys amidation by Chen *et al*. [49] and in a stepwise manner [50,51] (Fig. 1a). In continuation of our effort on organosulfur chemistry [28,52–59], we designed bilateral unsymmetrical Cys–Lys stapling reagents for non-symmetric Cys–Lys crosslinking with unprotected peptides (Fig. 1b). Based on the hard/soft acids and bases principle [60], Cys preferentially crosslinked at the sulfur site leaving the phthalimide group in 1 min, whereas Lys favored amidation targeting at PFP ester in 10 min. The length, angle, flexibility, rigidity and lipophilicity of unsymmetrical linkers are readily adjustable for enhancing helical conformation, solubility, hydrolytic stability, membrane permeability, binding affinity and biological activity. The matching ligation speed of the Cys and Lys labeling process enables the orthogonal crosslinking under ambient conditions with high chemoselectivity and regioselectivity in a self-assembly manner [61,62], successfully constructing diverse monocyclic/bicyclic peptides (Fig. 1b).

## RESULTS AND DISCUSSION

### Library of unsymmetrical Cys–Lys stapling linkers

**Figure 2.**
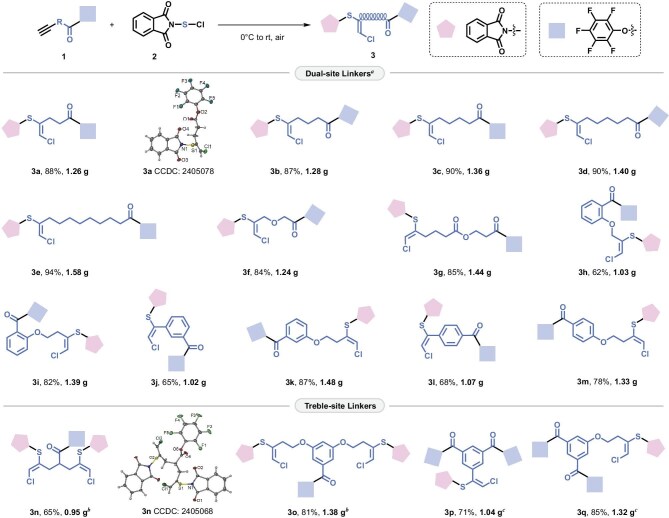
Library of unsymmetrical Cys–Lys stapling linkers. *^a^*Reaction conditions: **1** (3.0 mmol), phthalimidosulfenyl chloride **2** (3.3 mmol), CH_2_Cl_2_ (0.1 M), 0°C to rt, air, 2–12 h. All yields are for the isolated products. *^b^*Reaction conditions: **1** (2.0 mmol), **2** (4.4 mmol). *^c^*Reaction conditions: **1** (2.0 mmol), **2** (2.2 mmol).

The alkyne-substituted PFP ester **1** (see Fig. 2) was initially assembled via a condensation reaction between commercially available alkynoic acids and perfluorophenol. The alkynyl ester **1** retained terminal alkynyl sites for the second addition with phthalimidosulfenyl chloride **2** under an ambient atmosphere [28], generating highly *E*-selective electrophilic sulfur functional group **3**. The difunctional reactions established a series of bilaterally unsymmetrical reagents **3**, in which readily available alkynoic acids enabled the adjustable length, angle, flexibility, rigidity and lipophilicity. As further represented in Fig. 2, linker **3a**, featuring an ethyl linkage, was synthesized with a good yield and its *E*-configuration was unambiguously confirmed by X-ray crystallographic analysis. To incorporate adjustable length and flexibility, we designed extended linkers **3b–3e**, which were synthesized with excellent yield on a gram scale. The *O*-embedded **3f** and ester-embedded **3g** further promoted the diversiform stapling with tunable lipophilicity. Aryl moieties were installed to adjust the rigidity and angle of linkers, in which *o*-, *m*- and *p*-substituted modes were well compatible (**3h–3m**). In addition to dual-site staples, we also developed trifunctional linkers (Cys–Cys–Lys, **3n–3o**; Lys–Lys–Cys, **3p–3q**), establishing a promising platform for constructing bicyclic peptides. X-ray study of **3n** confirmed one PFP ester site and two electrophilic sulfur sites with *E*-configuration. Density functional theory (DFT) calculations gained further insight into the *Z*/*E* selectivity for stapling reagents (Fig. S1 in the Supplementary data). The nucleophilic addition from ethylethyne to phthalimidosulfenyl chloride generated a cyclopropenylthionium ion Int-6, spontaneously discharging a chloride ion. It triggered the ring opening via attack at the site with less steric hindrance (TS-7), delivering a thermodynamically stable *E*-configuration. All stapling reagents exhibited excellent solubility in acetonitrile and dimethylformamide (DMF) with concentrations ranging from 34 to 230 mM (Fig. S4). Although they demonstrated limited solubility in water, they were found to be soluble in the reaction system—a mixed solvent of acetonitrile and water (3 : 1, v : v)—ensuring the efficient proceeding of the stapling reaction.

### Unsymmetrically stapled cyclic peptides

**Figure 3.**
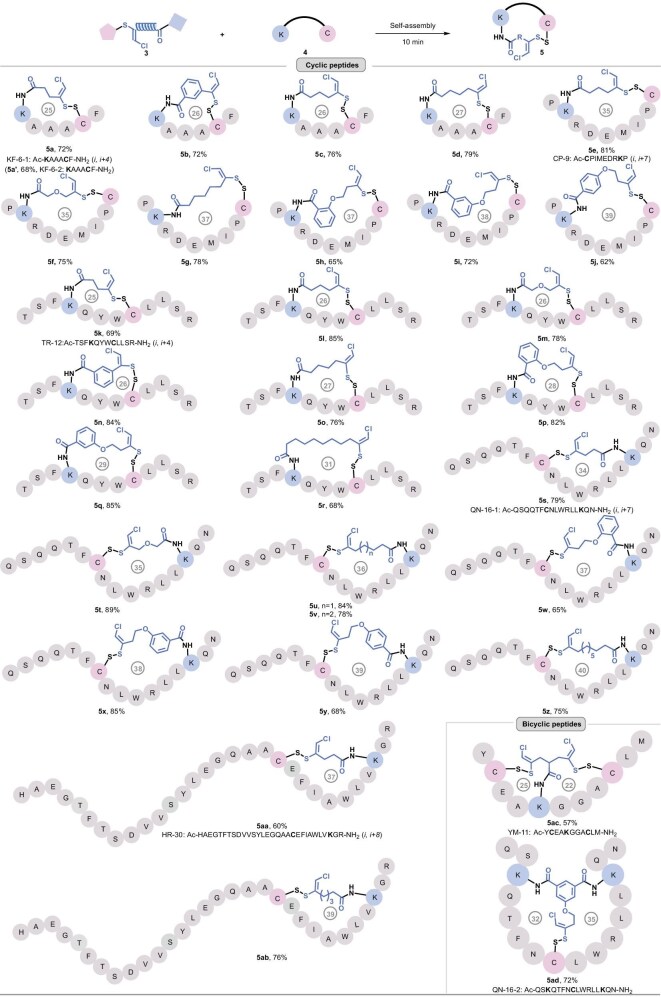
Unsymmetrically stapled cyclic peptides prepared by preparative HPLC. Reaction conditions: **4** (3 or 5 µmol), **3** (1.5 equiv.), CH_3_CN:H_2_O = 3:1, rt, air, DIPEA (4.0 equiv.), 10 min.

Subsequently, unsymmetrical peptide stapling was comprehensively explored (Fig. 3). Stapling reagent **3a** crosslinked with unprotected peptide KF-6-1: Ac-KAAACF-CONH_2_ (*i, i* + 4) via Cys connection within 1 min under ambient conditions; subsequent Lys ligation was achieved with addition of *N,N*-diisopropylethylamine (DIPEA) for 10 min. The relay stapling cooperatively generated the cyclic peptide **5a** with 72% yield. Notably, the native peptide KF-6-2: KAAACF-CONH_2_ without *N*-terminal acetylation protection was launched for the stapling process, generating **5a′** successfully, with 68% yield. It is worth noting that the fast disulfide click process was quantitative and specific, thereby enabling a one-step dual-stapling protocol within the same yield via adding **3a** and DIPEA together to the peptide KF-6-1. Following the single-step method, **5b–5d** were efficiently obtained in 72%–79% yields. The observed high chemoselectivity and regioselectivity were demonstrated via the control experiment of CP-9: AcHN-CPIMEDRKP-CONH_2_ (*i, i* + 7) and reagent **3b** (Figs S5–S10). The mass spectrometry fragmentation analysis unambiguously evidenced that the specific crosslinking at the sulfur site was launched by Cys rather than by Lys (Figs S9 and S10). The reagent **3d** with single Cys [(*tert*-butoxycarbonyl)-d-cysteine] (Figs S11–S15) and single Lys [*tert*-butyl (*tert*-butoxycarbonyl)-l-lysinate] (Figs S16–S19) further proved that Cys preferentially crosslinked with the sulfur site leaving the phthalimide group, whereas Lys favored amidation targeting at the PFP ester. Additionally, CP-9 was readily stapled by diversely functional linkers generating constrained peptides **5e–5j** in elegant conversions, such as the *o*-substituted **5f** (75%) and aryl-modified **5h–5j** (62%–72%). The *o*-, *m*- and *p*-linked manner brought distinct rigidity and angle for binding the *α*-helix conformation (Fig. 3). The versatility of this approach was further demonstrated through the stapling of peptide TR-12 (Ac-TSFKQYWCLLSR-CONH_2_) at (*i, i* + 4) positions, successfully generating 25–31-membered macrocyclic peptides **5k–5r** in yields ranging from 68% to 85%. Furthermore, more linkers with diverse length, angle, flexibility, rigidity and lipophilicity were assembled on peptide QN-16-1: Ac-QSQQTFCNLWRLLKQN-CONH_2_ (*i, i* + 7), delivering structurally locked peptides **5s–5z** with 35- to 40-membered loops with satisfactory yields. Building on the success of dual-site crosslinking, we next applied the Cys–Cys–Lys reagent **3n** and Lys–Lys–Cys reagent **3q** to triple-site stapling, successfully generating bicyclic peptide architectures. To address the potential limitations of peptide length in this stapling methodology, a linear 30-mer peptide HR-30 was stapled with reagents **3a** and **3c**, successfully generating the target 37- and 39-membered macrocyclic peptides **5aa** and **5ab** with 60% and 76% isolated yields, respectively. Under fixed conditions with stapling reagent **3d**, peptides KF-6-1, TR-12, QN-16-1 and HR-30 with varying lengths (6‒30-mer) delivered target stapled counterparts **5d, 5o, 5u** and **5ab** with good yields (76%‒84%), demonstrating that the stapling protocol exhibits broad applicability across diverse peptide sizes. Notably, the unprotected peptides YM-11 (Ac-YCEAKGGACLM-CONH_2_) and QN-16-2 (Ac-QSKQTFNCLWRLLKQN-CONH_2_) underwent smooth triple ligation. This transformation effectively locked the peptides into preorganized, rigid conformations, generating the respective bicyclic structures **5ac** and **5ad** (Fig. 3). Bicyclic peptides demonstrated superior properties over their monocyclic counterparts, including enhanced structural rigidity, improved target binding affinity and selectivity, greater metabolic stability and superior membrane permeability. [33,49,63,64]. The efficient relay conjugation highlights the orthogonal reactivity designed within the linkers: the phthalimide (PhthN^−^) group, with its superior leaving ability, provided a rapid and specific click site for highly nucleophilic Cys residues, whereas the PFP esters could only be activated by the higher nucleophilic Lys residues with the assistance of DIPEA, enabling the efficient construction of a library of multi-disulfide 25- to 40-membered macrocyclic peptides under biocompatible conditions.

### Biological characterization

**Figure 4.**
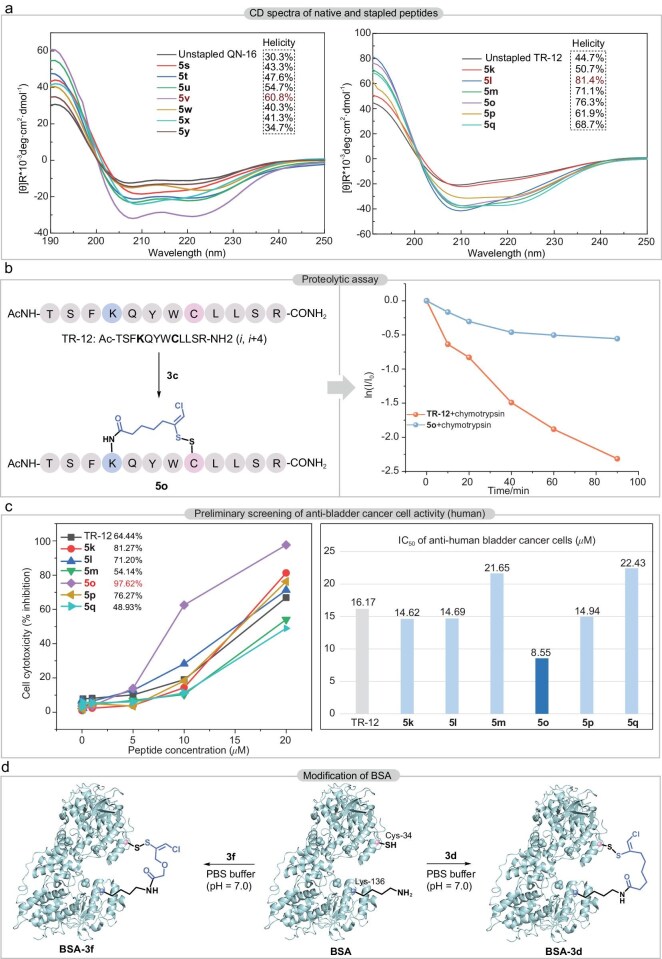
(a) CD spectra of native and stapled peptides in 40% TFE/water. (b) Proteolytic assay of stapled peptide **5o** and its native precursor TR-12. (c) Biological characterization of anti-human-bladder cancer cells. Three independent experiments were conducted, and the results were normalized. (d) Modification of BSA with **3d** and **3f** in PBS buffer (50 mM, pH = 7.0), rt, air, DIPEA (10.0 equiv.), 10 min.

The circular dichroism (CD) spectrum [65] of **5y** was measured in six different solvent systems (H_2_O, 5% trifluoroethanol (TFE)/H_2_O, 10% TFE/H_2_O, 20% TFE/H_2_O, 30% TFE/H_2_O and 40% TFE/H_2_O), in which the optimum *α*-helicity of 34.7% was observed in 40% TFE/H_2_O (Fig. S20). Two linear peptides TR-12 and QN-16 were contrasted with their stapled form under this condition (Fig. 4a). Compared to the 30.3% helicity of native peptide QN-16, the best helicity of its stapled peptide was **5v**, reaching 60.8%. A similar reinforcement of the helicity was observed between TR-12 (44.7%) and its stapled peptide **5l** (81.4%). Collectively, the data demonstrated that our stapling strategy reinforced the native *α*-helical fold, validating the rational design of the linkers. To evaluate the proteolytic stability of the stapled peptide [66], TR-12 and stapled peptide **5o** were exposed to chymotrypsin (Fig. 4b). High-performance liquid chromatography (HPLC) analysis indicated that under chymotrypsin for 1.5 h, 90% of linear peptide degraded, in contrast with 43% of **5o**. These results revealed an obviously enhanced proteolytic stability for stapled peptide compared to that of the unstapled precursor. To evaluate the bovine serum stability of stapled peptides, three representative linear peptide-derived binding peptides **5h, 5o** and **5w** were selected for comprehensive analysis under physiologically relevant conditions (37°C) (Figs S24–S26). CP-9-derived 5h demonstrated exceptional stability, requiring 14 h for complete degradation, while it took 8 and 6 h to fully decompose for TR-12-modified **5o** and QN-16-engineered 5w, respectively. The differential stability patterns observed among the three architectures demonstrated structure-dependent resistance to enzymatic degradation in serum environments. Activity tests at a concentration of 20 μM showed that peptide TR-12 had an inhibitory effect on human bladder cancer cells (64.44%) (Fig. 4c). We conducted anti-bladder cancer activity screening, in which stapled peptides **5k** (81.27%), **5l** (71.20%), **5o** (97.62%) and **5p** (76.27%) showed superior inhibitory activity. Additionally, the activity of the stapled peptides **5m** (54.14%) and **5q** (48.93%) were not compromised either. Furthermore, half-maximal inhibitory concentration (IC_50_) assays indicated that the constrained peptides exhibited increased IC_50_ values: TR-12 (16.17 μM), **5k** (14.62 μM), **5l** (14.69 μM), **5m** (21.65 μM), **5o** (8.55 μM), **5p** (14.94 μM) and **5q** (22.43 μM), which was consistent with the inhibitory activity (Fig. 4c). The observed disparity between the native and unstapled peptides served as direct validation of the strategy’s superiority. Furthermore, the amino-sulfhydryl stapling of bovine serum albumin (BSA) was performed, a protein with a molecular weight of 66 kDa containing 59 lysine residues and a single free cysteine residue (Cys-34, Fig. 4d). The thiol group of cysteine (Cys-34) and amine group of lysine residues in BSA were stapled through reagents **3d** and **3f** under the conditions of PBS buffer (pH 7.0) and the presence of DIPEA with a protein concentration of 50 μM within 10 min. MALDI-TOF MS analysis revealed characteristic molecular weights of the modified protein (67274.58 Da for BSA-**3d**, Fig. S29; and *m*/*z* = 67248.66 Da for BSA-**3f**, Fig. S30), demonstrating the biocompatibility of this stapling methodology in biological environments. Based on the amino acid sequence of BSA, we predicted its structure using AlphaFold2 and visualized the distribution of lysine residues proximal to Cys residues in VMD software. Given that the chain length of the stapling reagent used here is similar to that employed by the Gois group in their BSA stapling [67], we hypothesized that BSA underwent stapling with the reagent at Cys34 and Lys136 (Fig. S31).

## CONCLUSION

We have demonstrated Cys–Lys relay non-symmetric stapling with unprotected peptides/protein via a 17-membered library of unsymmetrical linkers with high chemoselectivity and regioselectivity in a self-assembly manner, stitching 6–30 amino acid residues to generate 25- to 40-membered conformationally restricted peptides in biocompatible media. The tunable geometry, including length, angle, flexibility and rigidity of stapling reagents, manipulated the crosslinking efficiency, cooperatively enhancing the pharmacological properties, such as *α*-helicity, proteolytic stability, serum stability and anti-bladder cancer activity. This protocol can be further applied for the establishment of various functional cyclic/bicyclic peptide libraries in both chemical biology study and peptide-based drug discovery.

## Supplementary Material

nwaf406_Supplemental_Files

## Data Availability

Deposition number CCDC 2405078 (**3a**) and CCDC 2405068 (**3n**) contain the supplementary crystallographic data for this paper. These data are provided free of charge by the joint Cambridge Crystallographic Data Centre and Fachinformationszentrum Karlsruhe Access Structures service.

## References

[1] Muttenthaler M, King GF, Adams DJ et al. Trends in peptide drug discovery. Nat Rev Drug Discov 2021; 20: 309–25. 10.1038/s41573-020-00135-8 33536635

[2] Liao Y, Jiang X. Chemo-selective modification of cysteine residue: synthesis and application in the discovery of potential drug candidates. Explor Drug Sci 2024; 540–54. 10.37349/eds.2024.00060

[3] D’Aloisio V, Dognini P, Hutcheon GA et al. PepTherDia: database and structural composition analysis of approved peptide therapeutics and diagnostics. Drug Discov Today 2021; 26: 1409–19. 10.1016/j.drudis.2021.02.019 33647438

[4] Tyndall DA, Nall T, Fairlie D. Proteases universally recognize beta strands in their active sites. Chem Rev 2005; 105: 973–1000. 10.1021/cr040669e 15755082

[5] Jenssen H, Aspmo S. Serum stability of peptides. Methods Mol Biol 2008; 494: 177–86. 18726574 10.1007/978-1-59745-419-3_10

[6] Walensky LD, Bird GH. Hydrocarbon-stapled peptides: principles, practice, and progress. J Med Chem 2014; 57: 6275–88. 10.1021/jm4011675 PMC4136684 24601557

[7] Vinogradov AA, Yin Y, Suga H. Macrocyclic peptides as drug candidates: recent progress and remaining challenges. J Am Chem Soc 2019; 141: 4167–81. 10.1021/jacs.8b13178 30768253

[8] Colas K, Bindl D, Suga H. Selection of nucleotide-encoded mass libraries of macrocyclic peptides for inaccessible drug targets. Chem Rev 2024; 124: 12213–41. 10.1021/acs.chemrev.4c00422 PMC11565579 39451037

[9] Blackwell H, Grubbs R. Highly efficient synthesis of covalently cross-linked peptide helices by ring-closing metathesis. Angew Chem Int Ed 1998; 37: 3281–4. 10.1002/(SICI)1521-3773(19981217)37:23<3281::AID-ANIE3281>3.0.CO;2-V 29711420

[10] Schafmeister CE, Po J, Verdine GL. An all-hydrocarbon cross-linking system for enhancing the helicity and metabolic stability of peptides. J Am Chem Soc 2000; 122: 5891–2. 10.1021/ja000563a

[11] Walensky LD, Kung AL, Escher I et al. Activation of apoptosis in vivo by a hydrocarbon-stapled BH_3_ helix. Science 2004; 305: 1466–70. 10.1126/science.1099191 PMC1360987 15353804

[12] Lau Y, De Andrade P, Wu Y et al. Peptide stapling techniques based on different macrocyclization chemistries. Chem Soc Rev 2015; 44: 91–102. 10.1039/C4CS00246F 25199043

[13] Iegre J, Gaynord JS, Robertson NS et al. Two-component stapling of biologically active and conformationally constrained peptides: past, present, and future. Adv Ther 2018; 1: 1800052. 10.1002/adtp.201800052

[14] Reguera L, Rivera D. Multicomponent reaction toolbox for peptide macrocyclization and stapling. Chem Rev 2019; 119: 9836–60. 10.1021/acs.chemrev.8b00744 30990310

[15] Zhan W, Duan H, Li C. Recent advances in metal-free peptide stapling strategies. Chem Bio Eng 2024; 1: 593–605. 10.1021/cbe.3c00123 PMC11835171 39974699

[16] Jo H, Meinhardt N, Wu Y et al. Development of *α*-helical calpain probes by mimicking a natural protein–protein interaction. J Am Chem Soc 2012; 134: 17704–13. 10.1021/ja307599z 22998171 PMC3523126

[17] Spokoyny AM, Zou Y, Ling J et al. A perfluoroaryl-cysteine S_N_Ar chemistry approach to unprotected peptide stapling. J Am Chem Soc 2013; 135: 5946–9. 10.1021/ja400119t PMC3675880 23560559

[18] Vinogradova EV, Zhang C, Spokoyny AM et al. Organometallic palladium reagents for cysteine bioconjugation. Nature 2015; 526: 687–91. 10.1038/nature15739 PMC4809359 26511579

[19] Brown SP, Smith AB III. Peptide/protein stapling and unstapling: introduction of s-tetrazine, photochemical release, and regeneration of the peptide/protein. J Am Chem Soc 2015; 137: 4034–7. 10.1021/ja512880g PMC4394111 25793939

[20] Assem N, Ferreira DJ, Wolan DW et al. Acetone-linked peptides: a convergent approach for peptide macrocyclization and labeling. Angew Chem Int Ed 2015; 54: 8665–8. 10.1002/anie.201502607 PMC4824200 26096515

[21] Wang Y, Chou DH. A thiol–ene coupling approach to native peptide stapling and macrocyclization. Angew Chem Int Ed 2015; 54: 10931–4. 10.1002/anie.201503975 26189498

[22] Zhang C, Welborn M, Zhu T et al. π-Clamp-mediated cysteine conjugation. Nat Chem 2016; 8 120–8. 10.1038/nchem.2413 26791894 PMC4861612

[23] Fadzen CM, Wolfe JM, Cho CF et al. Perfluoroarene–based peptide macrocycles to enhance penetration across the blood–brain barrier. J Am Chem Soc 2017; 139: 15628–31. 10.1021/jacs.7b09790 PMC5818988 28992407

[24] Jankovic B, Gulzar A, Zanobini C et al. Photocontrolling protein–peptide interactions: from minimal perturbation to complete unbinding. J Am Chem Soc 2019; 141: 10702–10. 10.1021/jacs.9b03222 31184111

[25] Luo Q, Tao Y, Sheng W et al. Dinitroimidazoles as bifunctional bioconjugation reagents for protein functionalization and peptide macrocyclization. Nat Commun 2019; 10: 142. 10.1038/s41467-018-08010-2 PMC6329768 30635561

[26] Zheng X, Li Z, Gao W et al. Condensation of 2-((alkylthio)(aryl)methylene)malononitrile with 1,2-aminothiol as a novel bioorthogonal reaction for site-specific protein modification and peptide cyclization. J Am Chem Soc 2020; 142: 5097–103. 10.1021/jacs.9b11875 32108479

[27] Fryszkowska A, An C, Alvizo O et al. A chemoenzymatic strategy for site-selective functionalization of native peptides and proteins. Science 2022; 376: 1321–7. 10.1126/science.abn2009 35709255

[28] Yu Q, Bai L, Jiang X. Disulfide click reaction for stapling of s-terminal peptides. Angew Chem Int Ed 2023; 62: e202314379. 10.1002/anie.202314379 37950389

[29] Hartmann P, Bohdan K, Hommrich M et al. Chemoselective umpolung of thiols to episulfoniums for cysteine bioconjugation. Nat Chem 2024; 16: 380–8. 10.1038/s41557-023-01388-7 PMC10914617 38123842

[30] Lautrette G, Touti F, Lee HG et al. Nitrogen arylation for macrocyclization of unprotected peptides. J Am Chem Soc 2016; 138: 8340–3. 10.1021/jacs.6b03757 27332147 PMC6150454

[31] Ricardo MG, Llanes D, Wessjohann LA et al. Introducing the Petasis reaction for late-stage multicomponent diversification, labeling, and stapling of peptides. Angew Chem Int Ed 2019; 58: 2700–4. 10.1002/anie.201812620 30589179

[32] Li H, Hu Y, Pu Q et al. Novel stapling by lysine tethering provides stable and low hemolytic cationic antimicrobial peptides. J Med Chem 2020; 63: 4081–9. 10.1021/acs.jmedchem.9b02025 32216308

[33] Li B, Wan Z, Zheng H et al. Construction of complex macromulticyclic peptides via stitching with formaldehyde and guanidine. J Am Chem Soc 2022; 144: 10080–90. 10.1021/jacs.2c04620 35639413

[34] Zhang Y, Yin R, Jiang H et al. Peptide stapling through site directed conjugation of triazine moieties to the tyrosine residues of a peptide. Org Lett 2023; 25: 2248–52. 10.1021/acs.orglett.3c00499 36966420

[35] Zuo Q, Song X, Yan J et al. Triazination/IEDDA cascade modular strategy installing pyridines/pyrimidines onto tyrosine enables peptide screening and optimization. J Am Chem Soc 2025; 147: 9576–89. 10.1021/jacs.4c17615 39885681

[36] Li X, Chen S, Zhang W et al. Stapled helical peptides bearing different anchoring residues. Chem Rev 2020; 120: 10079–144. 10.1021/acs.chemrev.0c00532 32794722

[37] Chen FJ, Lin W, Chen FE. Non-symmetric stapling of native peptides. Nat Rev Chem 2024; 8: 304–18. 10.1038/s41570-024-00591-5 38575678

[38] Zhan WL, Duan HL, Li CX. Recent advances in metal-free peptide stapling strategies. Chem Bio Eng 2024; 1: 593–605. 10.1021/cbe.3c00123 PMC11835171 39974699

[39] Li B, Tang H, Turlik A et al. Cooperative stapling of native peptides at lysine and tyrosine or arginine with formaldehyde. Angew Chem Int Ed 2021; 60: 6646–52. 10.1002/anie.202016267 33338303

[40] Li B, Wang L, Chen X et al. Extendable stapling of unprotected peptides by crosslinking two amines with *o*-phthalaldehyde. Nat Commun 2022; 13: 311. 10.1038/s41467-022-27985-7 35031608 PMC8760283

[41] Guo P, Chu X, Wu C et al. Peptide stapling by crosslinking two amines with *a*-ketoaldehydes through diverse modified glyoxal-lysine dimer linkers. Angew Chem Int Ed 2024; 63: e202318893. 10.1002/anie.202318893 38376389

[42] Chu X, Zhang Z, Xu X et al. Formamidine as an easy-on and easy-off linker for reversible crosslinking of two alkyl amines in peptide stapling and conjugation. Angew Chem Int Ed 2025; 64: e202422844. 10.1002/anie.202422844 39792487

[43] Kubota K, Dai P, Pentelute BL et al. Palladium oxidative addition complexes for peptide and protein cross-linking. J Am Chem Soc 2018; 140: 3128–33. 10.1021/jacs.8b00172 29406701 PMC5831526

[44] Zhang Y, Zhang Q, Wong CT et al. Chemoselective peptide cyclization and bicyclization directly on unprotected peptides. J Am Chem Soc 2019; 141: 12274–9. 10.1021/jacs.9b03623 31314512

[45] Todorovic M, Schwab KD, Zeisler J et al. Fluorescent isoindole crosslink (FlICk) chemistry: a rapid, user-friendly stapling reaction. Angew Chem Int Ed 2019; 58: 14120–4. 10.1002/anie.201906514 31211905

[46] Ceballos J, Grinhagena E, Sangouard G et al. Cys–Cys and Cys–Lys stapling of unprotected peptides enabled by hypervalent iodine reagents. Angew Chem Int Ed 2021; 60: 9022–31. 10.1002/anie.202014511 PMC8048981 33450121

[47] Silva M, Faustino H, Coelho J et al. Efficient amino-sulfhydryl stapling on peptides and proteins using bifunctional NHS-activated acrylamides. Angew Chem Int Ed 2021; 60: 10850–7. 10.1002/anie.202016936 33513271

[48] Wang Y, Czabala P, Raj M. Bioinspired one-pot furan-thiol-amine multicomponent reaction for making heterocycles and its applications. Nat Commun 2023; 14: 4086. 10.1038/s41467-023-39708-7 37429878 PMC10333318

[49] Chen F, Pinnette N, Yang F et al. Cysteine-directed proximity-driven crosslinking method for native peptide bicyclization. Angew Chem Int Ed 2023; 62: e202306813. 10.1002/anie.202306813 PMC10527288 37285100

[50] Brunel FM, Dawson PE. Synthesis of constrained helical peptides by thioether ligation: application to analogs of Gp41. Chem Commun 2005; 2552–4. 10.1039/b419015g 15900323

[51] Li X, Tolbert WD, Hu HG et al. Dithiocarbamate-inspired side chain stapling chemistry for peptide drug design. Chem Sci 2019; 10: 1522–30. 10.1039/C8SC03275K PMC6357863 30809370

[52] Xiao X, Feng M, Jiang X. New design of a disulfurating reagent: facile and straightforward pathway to unsymmetrical disulfanes by copper-catalyzed oxidative cross-coupling. Angew Chem Int Ed 2016; 55: 14121–5. 10.1002/anie.201608011 27726267

[53] Xiao X, Xue J, Jiang X. Polysulfurating reagent design for unsymmetrical polysulfide construction. Nat Commun 2018; 9: 2191. 10.1038/s41467-018-04306-5 PMC5989225 29875443

[54] Xue J, Jiang X. Unsymmetrical polysulfidation via designed bilateral disulfurating reagents. Nat Commun 2020; 11: 4170. 10.1038/s41467-020-18029-z 32820174 PMC7441163

[55] Zeng D, Ma Y, Deng W et al. Divergent sulfur(VI) fluoride exchange linkage of sulfonimidoyl fluorides and alkynes. Nat Synth 2022; 1: 455–63. 10.1038/s44160-022-00060-1

[56] Zeng D, Ma Y, Deng W et al. The linkage of sulfonimidoyl fluorides and unactivated alkenes via hydrosulfonimidoylation’ Angew Chem Int Ed 2022; 61: e202207100. 10.1002/anie.202207100 36104295

[57] Liao Y, Zhang S, Jiang X. Construction of thioamide peptides from chiral amino acids. Angew Chem Int Ed 2023; 62: e202303625. 10.1002/anie.202303625 37118109

[58] Yu Q, Zhang X, Jiang X. Bilateral unsymmetrical disulfurating reagent design for polysulfide construction. Angew Chem Int Ed 2024; 63: e202408158. 10.1002/anie.202408158 38923731

[59] Bai L, Jiang X. Smelless/stable/sustainable sulfur chemistry. CCS Chem 2025; 7: 1889–902. 10.31635/ccschem.025.202505568

[60] Pearson RG . Hard and soft acids and bases. J Am Chem Soc 1963; 85: 3533–9. 10.1021/ja00905a001

[61] Praetorius F, Dietz H. Self-assembly of genetically encoded DNA-protein hybrid nanoscale shapes. Science 2017; 355: eaam5488. 10.1126/science.aam5488 28336611

[62] Tan J, Wu J, Liu S et al. Macrocyclization of peptidoarylacetamides with self-assembly properties through late-stage palladium-catalyzed C(*sp*^2^)-H olefination. Sci Adv 2019; 5: eaaw0323. 10.1126/sciadv.aaw0323 PMC6408153 30873434

[63] Johnson WC . Protein secondary structure and circular dichroism: practical guide. Proteins 1990; 7: 205–14. 10.1002/prot.340070302 2194218

[64] Greenfield N . Methods to estimate the conformation of proteins and polypeptides from circular dichroism data. Anal Biochem 1996; 235: 1–10. 10.1006/abio.1996.0084 8850540

[65] Chen B, Li Y, Bai H et al. Unleashing the potential of natural biological peptide macropin: hydrocarbon stapling for effective breast cancer treatment. Bioorg Chem 2023; 140: 106770. 10.1016/j.bioorg.2023.106770 37604094

[66] Wang M, Pan D, Zhang Q et al. Site-selective polyfluoroaryl modification and unsymmetric stapling of unprotected peptides. J Am Chem Soc 2024; 146: 6675–85. 10.1021/jacs.3c12879 38427024

[67] Silva Maria JSA, Faustino H, Coelho Jaime AS et al. Efficient amino-sulfhydryl stapling on peptides and proteins using bifunctional NHS-activated acrylamides. Angew Chem Int Ed 2021; 60: 10850–7. 10.1002/anie.202016936 33513271

