# Supplementary material for: Cys–Lys stapling for unprotected peptides via tunable linkers
Source: Natl Sci Rev. 2025 Sep 22;12(11):nwaf406. doi: 10.1093/nsr/nwaf406 (PMC12596731; doi:10.1093/nsr/nwaf406)

Supporting Information

Cys–Lys Stapling for Unprotected Peptides via Tunable Linkers

Kaizhen Miao,#[a] Bei Fu,#,[a] Leiyang Bai,#[a] Chengliang Li[a] and Xuefeng Jiang*[a-c]

[1]Hainan Institute of East China Normal University, Shanghai Key Laboratory of Green Chemistry and Chemical Processes, State Key Laboratory of Petroleum Molecular & Process Engineering, School of Chemistry and Molecular Engineering, East China Normal University, 3663 North Zhongshan Road, Shanghai 200062, P. R. China.

[2]School of Chemistry and Chemical Engineering, Henan Normal University, Xinxiang, Henan, 453007, P.R. China.

[3]State Key Laboratory of Organometallic Chemistry, Shanghai Institute of Organic Chemistry, Chinese Academy of Sciences, 345 Lingling Road, Shanghai 200032, P. R. China.

#The authors contribute equally to this work.

*E-mail: xfjiang@chem.ecnu.edu.cn

Table of Contents

I.	General Information…………………………………………….………....	S2
II.	General Procedure and Data for Stapling Reagents 3………………	S3
III	Characterization of Native Peptides…………………………………	S13
IV	General Procedure and Data for Stapling Peptides 5………..…….	S19
V.	DFT calculations ..……………….................................…………	S40
VI	The solubility of stapling reagents……………………………………	S52
VII	Control experiments.………..……………………………………..	S53
VIII.	Circular Dichroism Measurements…...……………………………..	S61
IX.	Proteolysis assays…...……………………………………………....	S63
X.	Serum stability study…...………………………………………….	S65
XI.	Biological Experiments…...…………………………………………..	S72
XII.	BSA Macrocyclization…………………………………………………	S75
XIII.	References.……………………………………………………………..	S77
XIV.	X-ray Crystal Data……………………………………………...........	S79
XV.	NMR Spectra of Stapling Reagents.………..	S83

I. General Information

NMR Spectrum

^{1}H and (^{13}C and ^{19}F) spectra were collected on 300/400/500 MHz NMR spectrometers (Bruker AVANCE). Chemical shifts for protons are reported in parts per million (ppm) downfield and are referenced to residual protium in the NMR solvent ($CHCl_3$ = δ 7.26, CD_3CN = δ 1.94). Chemical shifts for carbon are reported in parts per million downfield and are referenced to the carbon resonances of solvent ($CHCl_3$ = δ 77.00, CD_3CN: (CD_3) = 13.0, (CN) = 118.0). Dates are represented as follows: chemical shift, multiplicity (br = broad, s = singlet, d = double, t = triplet, q = quartet, m = multiplet-), coupling constants in Hertz (Hz), integration.

Mass Spectroscopy

Mass spectra were in general recorded on a Shimadzu GCMS-QP2010 Ultra and an HP 5989A mass selective detector. Electrospray ionization (ESI) mass spectra (MS) experiments were performed on an Agilent Technologies 6224 TOF LC/MS. MALDI-TOF MS were performed on Bruker microflex.

Chromatography

Column chromatography was performed with silica gel (300-400 mesh ASTM). SHIMADZU LC-20AT Liquid Chromatograph (InertSustain C18 Column, 4.6×250 mm, 5μm) and SHIMADZU LC-20AR Liquid Chromatograph (Shim-pack GIS C18 Column, 20×250 mm, 5μm).

IR

SHIMADZU IR Tracer-100 Spectrometers.

Solvent

Acetonitrile ACS/HPLC certified (CH_3CN), Deionized water (H_2O), were bought and used without further purification. All native peptides were purchased from Gil Generation (Shanghai) Co., Ltd.

II. General Procedure and Data for Stapling Reagents 3.

Synthesis of Phthalimidosulfenyl Chloride.

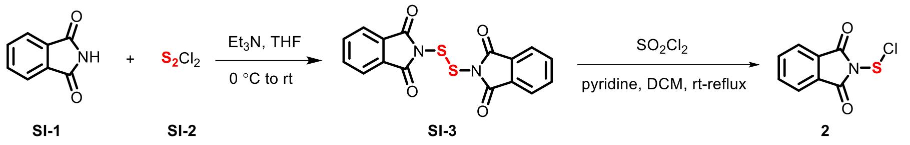

Phthalimide **SI-1** (2.9 g, 19.7 mmol) was dissolved in THF (40 mL) and triethylamine (4.1 mL, 30 mmol). The mixture was cooled in a salt ice bath, and then sulfur monochloride **SI-2** (0.8 mL, 10 mmol) was added dropwise to the cooled mixture. The solution was stirred for 2 h and then quenched with 60 mL of H_2O. The resulting precipitate was filtered and washed with diethyl ether. Crystallization from $CHCl_3$:CH_3OH [2:1 (v:v), 45 mL], yielded 2,2'-disulfanediylbis (isoindoline-1,3-dione) **SI-3** as a white solid (3.5 g, yield: 98%).[1]

Synthesis of phthalimidosulfenyl chloride **2**.[2]. Following a slightly modified procedure described in literature to a solution of di(1-phthalimidyl) disulfane **SI-3** (750 mg, 2 mmol) and anhydrous pyridine (0.1 mL, 1.2 mmol) in 10 mL of CH_2Cl_2 was added sulfuryl chloride (2 mL, 25 mmol) dropwise at room temperature. The yellow mixture was stirred at room temperature for 24 h, and reflux for 4 h (Note: Please ensure no water come inside during this period!). The solvent and excess sulfuryl chloride were removed under vacuum to give N-(chlorosulfenyl)phthalimide **2** as a yellow solid. (380 mg, yield: 90%).

General Procedure for Dual-site Stapling Reagents 3a-3m. [3]

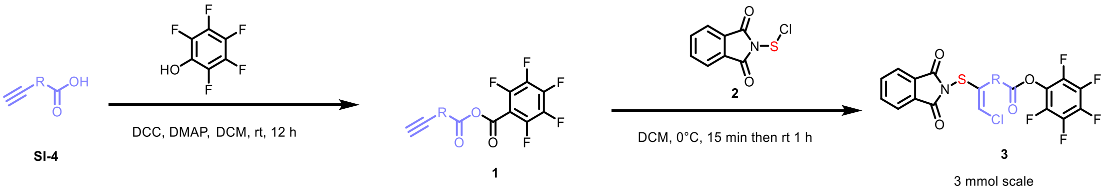

A flame-dried Schlenk-tube equipped with a magnetic stir bar was sealed with a septum, and degassed by alternating vacuum evacuation and argon backfilling (three times) before the acetylenic acid **SI-4** (5.00 mmol, 1.0 equiv.) was added, then a solution of pentafluorophenol (6.00 mmol, 1.2 equiv.), DMAP (0.500 mmol, 10 mol%), and DCC (6.00 mmol, 1.2 equiv.) in DCM (50 mL, 0.1M) was slowly added at 0 ºC (ice bath).

The reaction mixture was stirred for 20 min. Then the reaction was allowed to warm up to room temperature and stirred for an additional 2 h - 12 h (TLC monitoring). The alkynyl ester **1** was obtained by column chromatography. Then alkynyl ester **1** (3 mmol) was dissolved in dry DCM (16 mL). The mixture was cooled in a salt ice bath and then phthalimidosulfenyl chloride (3.3 mmol, 511 mg) was dissolved in DCM (6 mL) adding dropwise to the cooled mixture. the mixture was stirred at 0 °C 15 min, then at room temperature for 2 h - 12 h (TLC monitoring). The crude mixture was directly charged on silica gel and purified by column chromatography to give **3a-3m** as a white solid.

General Procedure for Treble-site Stapling Reagents 3n, 3o.

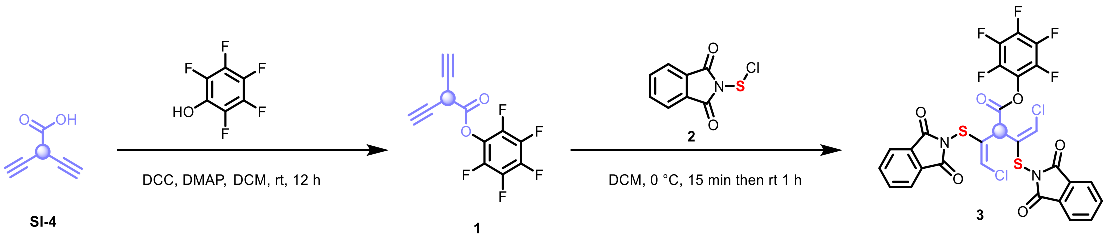

A flame-dried Schlenk-tube equipped with a magnetic stir bar was sealed with a septum, and degassed by alternating vacuum evacuation and argon backfilling (three times) before the tri-site acetylenic acid **SI-4** (5.00 mmol, 1.0 equiv.) was added, then a solution of pentafluorophenol (6.00 mmol, 1.2 equiv.), DMAP (0.500 mmol, 10 mol%), and DCC (6.00 mmol, 1.2 equiv.) in DCM (50 mL) was slowly added at 0 ºC (ice bath). The reaction mixture was stirred for 20 min. Then the reaction was allowed to warm up to room temperature and stirred for an additional 12 h. The alkynyl ester **1** was obtained by column chromatography. Then the alkynyl ester **1** (2.0 mmol) was dissolved in dry DCM (10 mL). The mixture was cooled in a salt ice bath and then phthalimidosulfenyl chloride (4.4 mmol, 933 mg) was dissolved in DCM (8 mL) adding dropwise to the cooled mixture. the mixture was stirred at 0 °C 15 min, then at room temperature for 2 h - 12 h (TLC monitoring). The crude mixture was directly charged on silica gel and purified by column chromatography to give **3n, 3o** as a white solid.

General Procedure for Treble-site Stapling Reagents 3p, 3q.

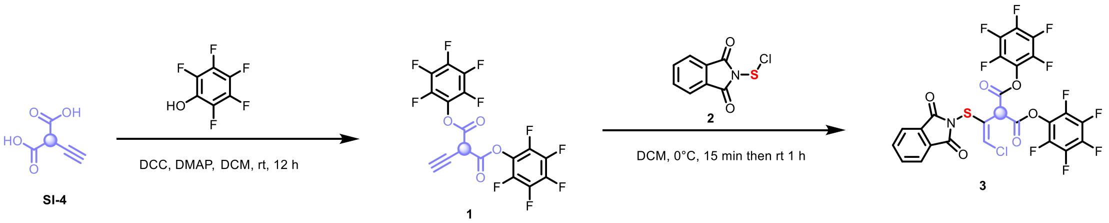

A flame-dried Schlenk-tube equipped with a magnetic stir bar was sealed with a septum, and degassed by alternating vacuum evacuation and argon backfilling (three times) before the tri-site acetylenic acid **SI-4** (5.00 mmol, 1.0 equiv.) was added, then a solution of pentafluorophenol (12.00 mmol, 2.4 equiv.), DMAP (1.00 mmol, 20 mol%), and DCC (12.00 mmol, 2.4 equiv.) in DCM (50 mL, 0.1M) was slowly added at 0 ºC (ice bath). The reaction mixture was stirred for 20 min. Then the reaction was allowed to warm up to room temperature and stirred for an additional 12 h. The alkynyl ester **1** was obtained by column chromatography. Then alkynyl ester **1** (2.0 mmol) was dissolved in dry DCM (6 mL). The mixture was cooled in a salt ice bath and then phthalimidosulfenyl chloride (2.2 mmol, 469 mg) was dissolved in DCM (2 mL) adding dropwise to the cooled mixture. the mixture was stirred at 0 °C 15 min, then at room temperature for 2-12 h (TLC monitoring). The crude mixture was directly charged on silica gel and purified by column chromatography to give **3p, 3q** as a white solid.

Characterization of Stapling Reagents 3. [4]

Perfluorophenyl (E)-5-chloro-4-((1,3-dioxoisoindolin-2-yl) thio) pent-4-enoate (3a).

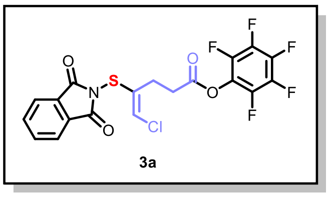

Yield: 88%; white solid; Mp 98 - 99 ºC; ^{1}H NMR (400 MHz, $CDCl_3$) δ 7.95 – 7.91 (m, 2H), 7.82 – 7.78 (m, 2H), 7.01 (s, 1H), 3.20 (t, J = 7.6 Hz, 2H), 2.78 (t, J = 7.6 Hz, 2H).; ^{13}C NMR (125 MHz, $CDCl_3$) δ 168.33, 167.60, 142.16 – 141.96 (m, C_{Ar}-F), 140.62 – 139.96 (m, C_{Ar}-F), 138.97 – 136.68 (m, C_{Ar}-F), 136.06, 134.97, 131.73, 127.26, 125.06 – 124.80 (m, C_{Ar}-O), 124.22, 30.48, 25.4.; ^{19}F NMR (376 MHz, $CDCl_3$) δ -152.22 – -152.32 (m, 2F), -157.98 (t, J = 21.6 Hz, 1F), -162.25 – -162.42 (m, 2F).; HRMS (ESI) Calcd for $C_{19}H_9ClF_5NO_4SNa$ $[M+Na]^+$ 499.9753, found 499.9752. IR: 709.80, 794.67, 995.27, 1041.56, 1103.28, 1273.02, 1519.91, 1712.79, 1743.65,

1782.23.

Perfluorophenyl (E) -6-chloro-5- ((1,3-dioxoisoindolin-2-yl) thio) hex-5-enoate (3b).

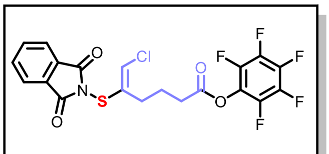

Yield: 87%; white solid; Mp 118-119 ºC; ^{1}H NMR (400 MHz, $CDCl_3$) δ 7.95 – 7.90 (m, 2H), 7.82 – 7.78 (m, 2H), 6.87 (s, 1H), 2.75 (t, J = 7.2 Hz, 2H), 2.48 (t, J = 7.4 Hz, 2H), 2.23 – 2.16 (m, 2H).; ^{13}C NMR (125 MHz, $CDCl_3$) δ 168.94, 167.62, 142.20 – 142.00 (m, C_{Ar}-F), 140.56 – 140.00 (m, C_{Ar}-F), 138.97 – 136.68 (m, C_{Ar}-F), 137.25, 134.93, 131.77, 125.14 – 124.97 (m, C_{Ar}-O), 124.91, 124.17, 32.19, 29.20, 22.01.; ^{19}F NMR (376 MHz, $CDCl_3$) δ -152.45 – -152.55 (m, 2F), -158.14 (t, J = 21.6 Hz, 1F), -162.33 – -162.48 (m, 2F).; HRMS (ESI) Calcd for $C_{20}H_{11}ClF_5NO_4SNa$ $[M+Na]^+$ 513.9910, found 513.9913.; IR:717.52, 871.82, 1002.98, 1103.28, 1280.73, 1527.62, 1743.65, 1789.94.

Perfluorophenyl (E) -7-chloro-6- ((1,3-dioxoisoindolin-2-yl) thio) hept-6-enoate (3c).

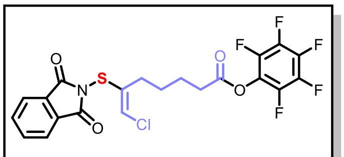

Yield: 90%; white solid; Mp 135 - 136 ºC; ^{1}H NMR (400 MHz, $CDCl_3$) δ 7.95 – 7.90 (m, 2H), 7.82 –7.78 (m, 2H), 6.83 (s, 1H), 2.72 (t, J = 6.9 Hz, 2H), 2.40 (t, J = 6.9 Hz, 2H), 1.86 – 1.79 (m, 2H); ^{13}C NMR (125 MHz, $CDCl_3$) δ 169.22, 167.62, 141.21 – 142.01 (m, C_{Ar}-F), 140.53 – 140.01 (m, C_{Ar}-F), 138.97 – 136.68 (m, C_{Ar}-F), 137.95, 134.90, 131.78, 125.22 – 124.94 (m, C_{Ar}-O), 124.27, 124.16, 32.96, 29.87, 26.26, 23.97.; ^{19}F NMR (376 MHz, $CDCl_3$) δ -152.63 – -152.73 (m, 2F), -158.24 (t, J = 21.6 Hz, 1F), -162.37 – -162.53 (m, 2F).; HRMS (ESI) Calcd for $C_{21}H_{13}ClF_5NO_4SNa$ $[M+Na]^+$ 528.0066, found 528.0069.; IR:725.23, 871.82, 1002.98, 1111.00, 1188.15, 1280.73, 1527.62, 1743.65, 1789.94.

Perfluorophenyl (E) -8-chloro-7- ((1,3-dioxoisoindolin-2-yl) thio) oct-7-enoate (3d).

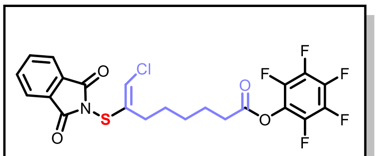

Yield: 90%; white solid; Mp 87 - 88 ºC; ^{1}H NMR (400 MHz, $CDCl_3$) δ 7.92 – 7.90 (m, 2H), 7.80 – 7.78 (m, 2H), 6.76 (s, 1H), 2.67 (t, J = 7.4 Hz, 2H), 2.35 (t, J =

7.6 Hz, 2H), 1.84 – 1.73 (m, 4H), 1.49 – 1.41 (m, 2H).; ^{13}C NMR (100 MHz, $CDCl_3$) δ 168.39, 166.59, 141.51 – 141.26 (m, C_{Ar}-F), 139.80 – 139.77 (m, C_{Ar}-F), 138.27 – 135.41 (m, C_{Ar}-F), 137.37, 133.85, 130.81, 124.31 – 123.97 (m C_{Ar}-O), 123.09, 122.49, 32.09, 29.03, 27.09, 25.46, 23.36.; ^{19}F NMR (376 MHz, $CDCl_3$) δ -152.73 – -152.81 (m, 2F), -158.32 (t, J = 21.6 Hz, 1F), -162.45 – -162.58 (m, 2F).; HRMS (ESI) Calcd for $C_{22}H_{15}ClF_5NO_4SNa$ $[M+Na]^+$ 542.0223, found 542.0226.; IR:717.52, 1002.98, 1049.28, 1280.73, 1519.91, 1743.65, 1789.94.

Perfluorophenyl (E)-11-chloro-10-((1,3-dioxoisoindolin-2-yl) thio) undec-10-enoate (3e).

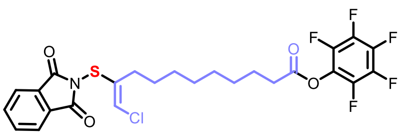

3e

Yield: 94%; white solid; Mp 64 - 65 ºC; ^{1}H NMR (400 MHz, $CDCl_3$) δ 7.93 – 7.88 (m, 2H), 7.80 – 7.76 (m, 2H), 6.72 (s, 1H), 2.64 (t, J = 7.4 Hz, 2H), 2.32 (t, J = 7.6 Hz, 2H), 1.79 – 1.70 (m, 2H), 1.68 – 1.63 (m, 2H), 1.37 – 1.31 (m, 8H).; ^{13}C NMR (125 MHz, $CDCl_3$) δ 169.53, 167.56, 142.23 – 142.03 (m, C_{Ar}-F), 140.48 – 140.04 (m, C_{Ar}-F), 138.97 – 136.68 (m, C_{Ar}-F), 138.67, 134.80, 131.83, 125.28 – 125.01 (m, C_{Ar}-O), 124.04, 123.00, 33.29, 30.41, 28.99, 28.93, 28.86, 28.76, 26.95, 24.72.; ^{19}F NMR (376 MHz, $CDCl_3$) δ -152.77 – -152.86 (m, 2F), -158.32 (t, J = 21.6 Hz, 1F), -162.42 – -162.57 (m, 2F).; HRMS (ESI) Calcd for $C_{25}H_{21}ClF_5NO_4SNa$ $[M+Na]^+$ 584.0692, found 584.0691.; 709.80, 794.67, 864.11, 995.27, 1041.56, 1080.14, 1273.02, 1519.91, 1712.79, 1743.65, 1789.94.

Perfluorophenyl (E)-2-((2-chloro-3-((1,3-dioxoisoindolin-2-yl)thio)allyl)oxy)acetate (3f).

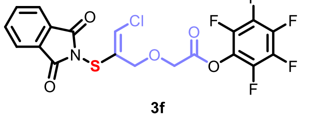

3f

Yield: 84%; white solid; Mp 106 - 107 ºC; ^{1}H NMR (500 MHz, $CDCl_3$) δ 7.93 – 7.89 (m, 2H), 7.79 – 7.77 (m, 2H), 7.06 (s, 1H), 4.57 (s, 2H), 4.45 (s, 2H).; ^{13}C NMR (125 MHz, $CDCl_3$) δ 167.47, 165.87, 142.06 – 141.86(m, C_{Ar}-F), 140.79 – 139.86(m, C_{Ar}-F), 138.97 – 136.68(m, C_{Ar}-F), 134.76, 133.18, 131.90, 128.65, 124.46 – 124.21(m, C_{Ar}-O), 124.05, 68.36, 66.59.; ^{19}F NMR (470 MHz, $CDCl_3$) δ -152.34 – -152.31 (m, 2F), -157.29 (t, J = 21.6 Hz, 1F), -161.89 – -162.00 (m, 2F). HRMS (ESI) Calcd for

$C_{19}H_9ClF_5NO_5SNa$ [M+Na]$^+$ 515.9702, found 515.9703.; IR: 709.80, 794.67, 995.27, 1049.28, 1280.73, 1342.46, 1519.91, 1712.79, 1743.65, 1797.66.

3-oxo-3-(perfluorophenoxy) propyl (E) -6-chloro-5-((1,3-dioxoisoindolin-2-yl) thio) hex-5-enoate (3g).

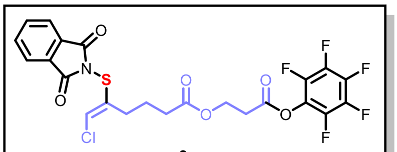

3g

Yield: 85%; colorless liquid; ^{1}H NMR (400 MHz, CD_3CN) δ 7.71 – 7.68 (m, 2H), 7.67 – 7.63 (m, 2H), 6.52 (s, 1H), 3.90 (t, J = 6.2 Hz, 2H), 2.78 (t, J = 6.5 Hz, 2H), 2.50 (t, J = 6.5 Hz, 2H), 2.21 (t, J = 7.5 Hz, 2H), 1.74 (dt, J = 4.9, 2.5 Hz, 2H).; ^{13}C NMR (125 MHz, CD_3CN) δ 171.58, 168.96, 167.75, 142.24 – 142.07 (m, C_{Ar}-F), 140.68– 142.09 (m, C_{Ar}-F), 139.10 – 136.88 (m C_{Ar}-F), 138.28, 135.03, 132.02, 125.07 – 124.80 (m, C_{Ar}-O), 123.71, 120.95, 63.56, 28.47, 28.11, 26.17, 25.94.; ^{19}F NMR (376 MHz, $CDCl_3$) δ -152.40 – 152.51 (m, 2F), -157.78 (t, J = 21.6 Hz, 1F), -162.09 – -162.26 (m, 2F).; HRMS (ESI) Calcd for $C_{23}H_{15}ClF_5NO_6SNa$ [M+Na]$^+$ 586.0121, found 586.0116.; IR: 709.80, 794.67, 910.27, 1049.28, 1149.57, 1273.02, 1342.46, 1519.91, 1712.79, 1743.65, 1797.66.

Perfluorophenyl (E)-2-((3-chloro-2-((1,3-dioxoisoindolin-2-yl) thio) allyl) oxy) benzoate (3h).

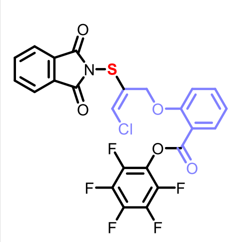

3h

Yield: 62%; white solid; Mp 143 - 144 ºC; ^{1}H NMR (400 MHz, $CDCl_3$) δ 7.92 (dd, J = 7.8, 1.8 Hz, 1H), 7.78 – 7.75 (m, 2H), 7.72 – 7.68 (m, 2H), 7.59 – 7.54 (m, 1H), 7.14 (s, 1H), 7.08 – 7.00 (m, 2H), 5.06 (s, 2H).; ^{13}C NMR (100 MHz, $CDCl_3$) δ 167.31, 160.47, 159.04, 142.69 – 142.45 (m, C_{Ar}-F), 140.61 – 139.94 (m, C_{Ar}-F), 139.21 – 136.21 (m, C_{Ar}-F), 135.65, 134.46, 132.93, 132.88, 131.92, 130.46, 125.46 – 124.26 (m, C_{Ar}-O), 123.80, 121.29, 116.66, 114.02, 67.55.; ^{19}F NMR (376 MHz, $CDCl_3$) δ -151.64 – -151.73 (m, 2F), -158.70 (t, J = 21.6 Hz, 1F), -162.65 – 162.80 (m, 2F).; ^{13}C NMR (100 MHz, $CDCl_3$) HRMS (ESI) Calcd for $C_{24}H_{11}ClF_5NO_5SNa$ [M+Na]$^+$ 577.9859, found 577.9863.; IR:717.52, 802.39, 1026.13, 1072.42, 1118.71, 1219.01, 1280.73, 1450.47, 1527.62, 1743.65, 1774.51.

Perfluorophenyl (E)-2-((3-chloro-4-((1,3-dioxoisoindolin-2-yl) thio) but-3-en-1-yl)oxy) benzoate (3i).

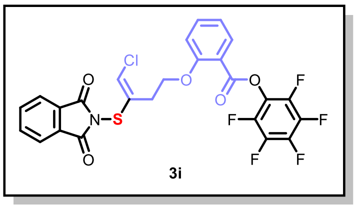

Yield 82%; liquid; ^{1}H NMR (400 MHz, $CDCl_3$) δ 7.92 (dd, J = 7.8, 1.8 Hz, 1H), 7.83 – 7.80 (m, 2H), 7.74 – 7.72 (m, 2H), 7.62 – 7.57 (m, 1H), 7.15 – 7.13 (m, 1H), 7.06 (s, 1H), 7.02 (dd, J = 7.5, 0.9 Hz, 1H), 4.50 (t, J = 6.5 Hz, 2H), 2.91 (t, J = 6.5 Hz, 2H).; ^{13}C NMR (125 MHz, $CDCl_3$) δ 167.60, 161.23, 159.31, 142.47 – 142.27 (m, C_{Ar}-F), 140.43 – 140.32 (m, C_{Ar}-F), 139.01 – 136.73 (m, C_{Ar}-F), 135.56, 134.72, 134.35, 132.80, 131.73, 129.11, 125.56 – 125.29 (m, C_{Ar}-O), 123.97, 120.69, 116.51, 113.95, 65.53, 30.45.; ^{19}F NMR (376 MHz, $CDCl_3$) δ -152.07 – -152.18 (m, 2F), -158.60 (t, J = 21.6 Hz, 1F), -162.61 – -162.76 (m, 2F).; HRMS (ESI) Calcd for $C_{25}H_{13}ClF_5NO_5SNa$ $[M+Na]^+$ 592.0015, found 592.0025. IR: 725.23, 1026.13, 1126.43, 1195.87, 1296.16, 1450.47, 1572.62, 1743.65.

Perfluorophenyl (E)-3-(2-chloro-1-((1,3-dioxoisoindolin-2-yl) thio) vinyl) benzoate (3j).

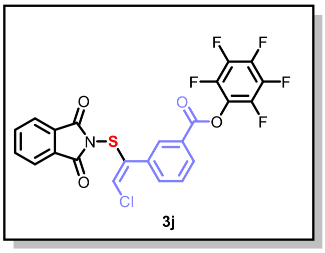

Yield 65%; white solid; Mp 128-129 ºC; ^{1}H NMR (300 MHz, $CDCl_3$) δ 8.38 (t, J = 1.6 Hz, 1H), 8.10 (dt, J = 7.8, 1.4 Hz, 1H), 7.97 (dt, J = 7.8, 1.4 Hz, 1H), 7.84 –7.80 (m, 2H), 7.76 –7.72 (m, 2H), 7.55 (t, J = 7.8 Hz, 1H), 7.12 (s, 1H).; ^{13}C NMR (125 MHz, $CDCl_3$) δ 166.96, 161.93, 142.40 – 142.21 (m, C_{Ar}-F), 140.72 – 140.20 (m, C_{Ar}-F), 139.07 – 136.78 (m, C_{Ar}-F), 137.06, 136.10, 134.84, 133.73, 132.01, 131.55, 131.41, 129.11, 127.08, 125.35 – 125.09 (m, C_{Ar}-O), 124.05, 122.40.; ^{19}F NMR (282 MHz, $CDCl_3$) δ -152.21 – -152.34 (m, 2F), -157.77 (t, J = 21.6 Hz, 1F), -162.15 – -162.36 (m, 2F).; HRMS (ESI) Calcd for $C_{23}H_{10}ClF_5NO_4S$ $[M+H]^+$ 525.9934, found 525.9938. IR: 717.52, 802.39, 995.27, 1049.28, 1157.29, 1242.16, 1273.02, 1519.91, 1712.79, 1743.65.

Perfluorophenyl (E)-3-((3-chloro-4-((1,3-dioxoisoindolin-2-yl) thio) but-3-en-1-yl) oxy) benzoate (3k).

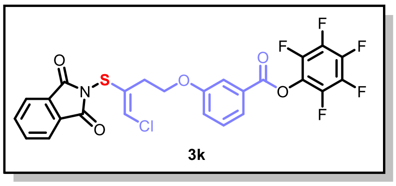

Yield: 87%; white solid; Mp 108 - 109 ºC; ^{1}H NMR (400 MHz, $CDCl_3$) δ 7.87 – 7.85 (m, 2H), 7.76 – 7.74 (m, 2H), 7.54 (t, J = 2.0 Hz, 1H), 7.38 (t, J = 8.0 Hz, 1H), 7.14 – 7.09 (m, 2H), 4.38 (t, J = 6.3 Hz, 2H), 2.93 (t, J = 6.3 Hz, 2H).; ^{13}C NMR (100 MHz, $CDCl_3$) δ 167.66, 162.35, 158.75, 142.70 – 142.49(m, C_{Ar}-F), 141.03 – 139.94(m, C_{Ar}-F), 139.35 – 136.54(m, C_{Ar}-F), 134.83, 134.68, 131.77, 129.89, 128.39, 128.00, 125.56 – 125.08 (m, C_{Ar}-O), 124.08, 123.30, 121.45, 116.24, 65.03, 31.04,; ^{19}F NMR (376 MHz, $CDCl_3$) δ -152.33 – -152.44 (m, 2F), -157.86 (t, J = 21.6 Hz, 1F), -162.17 – 162.32 (m, 2F); HRMS (ESI) Calcd for $C_{25}H_{13}ClF_5NO_5SNa$ $[M+Na]^+$ 592.0015, found 592.0024.; IR: 717.52, 995.27, 1041.56, 1195.87, 1273.02, 1519.91, 1597.06, 1712.79, 1743.65.

Perfluorophenyl (E)-4-(2-chloro-1-((1,3-dioxoisoindolin-2-yl) thio) vinyl) benzoate (3l).

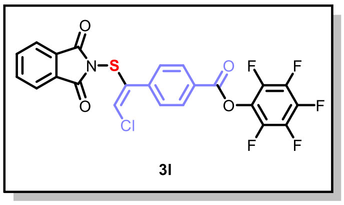

Yield: 68%; white solid; Mp 96 - 97 ºC; ^{1}H NMR (400 MHz, $CDCl_3$) δ 8.15 (d, J = 8.4 Hz, 2H), 7.85 – 7.82 (m, 2H), 7.78 (d, J = 8.8 Hz, 2H), 7.77 – 7.74 (m, 2H), 7.18 (s, 1H); ^{13}C NMR (125 MHz, $CDCl_3$) δ 167.02, 161.98, 142.38 – 142.19 (m, C_{Ar}-F), 140.73 – 140.19 (m, C_{Ar}-F), 139.18, 139.08 – 136.79 (m, C_{Ar}-F), 137.21, 134.92, 131.50, 130.65, 130.44, 127.37, 125.30 – 125.02 (m, C_{Ar}-O), 124.15, 123.46.; ^{19}F NMR (376 MHz, $CDCl_3$) δ -152.31 – -152.41 (m, 2F), -157.70 (t, J = 21.6 Hz, 1F), -162.09 – -162.24 (m, 2F).; HRMS (ESI) Calcd for $C_{23}H_9ClF_5NO_4SNa$ $[M+Na]^+$ 547.9753, found 547.9755.; IR: 756.10, 848.68, 995.27, 1049.28, 1172.72, 1249.87, 1519.91, 1612.49, 1759.08.

Perfluorophenyl (E)-4-((3-chloro-4-((1,3-dioxoisoindolin-2-yl) thio) but-3-en-1-yl) oxy) benzoate (3m).

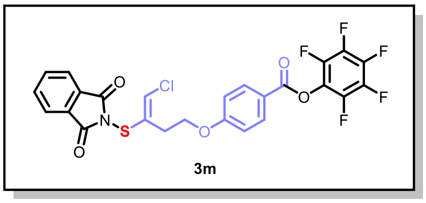

Yield: 78%; white solid; Mp 135 - 136 ºC; ^{1}H NMR (400 MHz, $CDCl_3$) δ 8.04 (d, J = 8.5 Hz, 2H), 7.88 -7.86 (m, 2H), 7.78 –7.76 (m, 2H), 7.13 (s, 1H), 6.90 (d, J = 8.5 Hz, 2H), 4.43 (t, J = 6.4 Hz, 2H), 2.94 (t,

J = 6.4 Hz, 2H).;^{13}C NMR (100 MHz, $CDCl_3$) δ 167.61, 163.65, 162.15, 142.86 – 142.56 (m, C_{Ar}-F), 140.29 – 139.10 (m, C_{Ar}-F), 138.35 – 136.50 (m, C_{Ar}-F), 134.85, 134.33, 132.85, 131.70, 128.91, 125.62 – 125.28 (m, C_{Ar}-O), 124.08, 119.09, 114.70, 64.85, 30.80,; ^{19}F NMR (376 MHz, $CDCl_3$) δ -152.49 – -152.63 (m, 2F), -158.27 (t, *J* = 21.6 Hz, 1F), -162.42 – 162.55 (m, 2F).;HRMS (ESI) Calcd for $C_{25}H_{13}ClF_5NO_5SNa$ $[M+Na]^+$ 592.0015, found 592.0019. IR: 709.80, 848.68, 1002.98, 1041.56, 1172.72, 1242.16, 1273.02, 1342,46, 1427.32, 1465.90, 1519.91, 1604.77, 1712.79, 1743.65.

Perfluorophenyl (E)-5-chloro-2-((E)-3-chloro-2-((1,3-dioxoisoindolin-2-yl) thio) allyl)-4-((1,3-dioxoisoindolin-2-yl) thio) pent-4-enoate (3n).

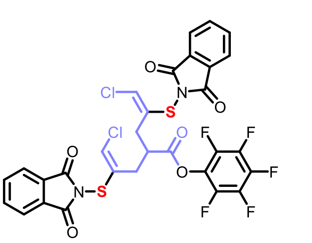

3n

Yield: 65%; white solid; Mp 157 - 158 ºC; ^{1}H NMR (400 MHz, $CDCl_3$) δ 7.91 – 7.88 (m, 4H), 7.79 – 7.77 (m, 4H), 7.14 (s, 2H), 4.39 – 4.32 (m, 1H), 3.02 (dd, *J* = 14.4, 7.9 Hz, 2H), 2.72 (dd, *J* = 14.4, 6.7 Hz, 2H).; ^{13}C NMR (125 MHz, $CDCl_3$) δ 167.63, 159.65, 142.40 – 142.28 (m, C_{Ar}-F), 140.69 – 140.27 (m, C_{Ar}-F), 138.09 – 136.85 (m, C_{Ar}-F), 134.84, 134.65, 131.75, 128.42, 125.46 – 125.20(m, C_{Ar}-O), 124.07, 109.24, 108.08, 65.05, 31.07.; ^{19}F NMR (376 MHz, $CDCl_3$) δ -151.24 – -151.43 (m, 2F), -157.93 (t, *J* = 21.6 Hz, 1F), -162.21 – -162.37 (m, 2F).; HRMS (ESI) Calcd for $C_{30}H_{15}Cl_2F_5N_2O_6S_2Na$ $[M+Na]^+$ 750.9561, found 750.9532. IR: 709.80, 794.67, 995.27, 1041.56, 1273.02, 1342,46, 1427.32, 1465.90, 1519.91, 1712.79, 1743.65.

Perfluorophenyl 3,5-bis(((E)-4-chloro-3-((1,3-dioxoisoindolin-2-yl) thio) but-3-en-1-yl) oxy) benzoate (3o)

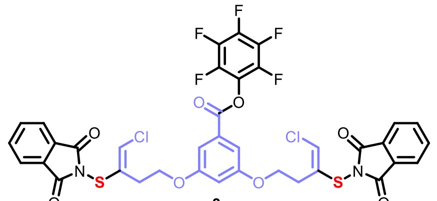

3o

Yield 81%; white solid; Mp 74 - 75 ºC; ^{1}H NMR (500 MHz, $CDCl_3$) δ 7.88 – 7.86 (m, 4H), 7.77 – 7.75 (m, 4H), 7.11 (s, 2H), 7.09 (d, *J* = 2.2 Hz, 2H), 6.49 (t, *J* = 2.2 Hz, 1H), 4.30 (t, *J* = 6.3 Hz, 4H), 2.91 (t, *J* = 6.3 Hz, 4H).; ^{13}C NMR (125 MHz, $CDCl_3$) δ 167.63, 159.65, 142.40 – 142.28 (m, C_{Ar}-F) , 140.69 – 140.27 (m, C_{Ar}-F), 139.09 –136.85 (m, C_{Ar}-F), 134.84, 134.65, 131.75, 128.42, 125.43 – 125.20 (m, C_{Ar}-O), 124.07, 109.24, 108.08, 65.05, 31.07.; ^{19}F NMR (282 MHz, $CDCl_3$) δ -152.28

– -152.41 (m, 2F), -157.82 (t, *J* = 21.6 Hz, 1F), -162.10 – -162.31 (m, 2F).; HRMS (ESI) Calcd for $C_{37}H_{21}Cl_2F_5N_2O_8S_2Na$ [M+Na]$^+$ 872.9929, found 872.9929.; IR: 717.52, 794.67, 864.11, 995.27, 1041.56, 1172.72, 1273.02, 1342.46, 1519.91, 1597.06, 1712.79, 1743.65.

Bis(perfluorophenyl) (E)-5-((4-chloro-3-((1,3-dioxoisoindolin-2-yl) thio) but-3-en-1-yl) oxy) isophthalate (3p)

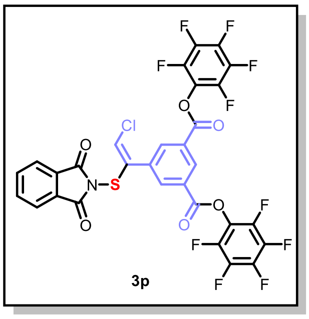

Yield 71%; white solid; Mp 152 - 153 ºC; ^{1}H NMR (400 MHz, $CDCl_3$) δ 8.91 (t, *J* = 1.5 Hz, 1H), 8.80 (d, *J* = 1.5 Hz, 2H), 7.87 – 7.85 (m, 2H), 7.78 – 7.75 (m, 2H), 7.33 (s, 1H).; ^{13}C NMR (125 MHz, $CDCl_3$) δ 166.93, 160.90, 142.30 – 142.18 (m, C_{Ar}-F), 140.99 – 140.17 (m, C_{Ar}-F), 139.14 – 136.87 (m, C_{Ar}-F), 137.55, 135.79, 135.34, 135.00, 133.13, 131.51, 128.35, 125.37, 125.05 – 124.79 (m, C_{Ar}-O), 124.22.; ^{19}F NMR (282 MHz, $CDCl_3$) δ -151.01 – -152.13 (m, 4F), -156.93 (t, *J* = 21.6 Hz, 2F), -161.64 – -161.85 (m, 4F).; HRMS (ESI) Calcd for $C_{30}H_8ClF_{10}NO_6SNa$ [M+Na]$^+$ 757.9473, found 757.9471. IR: 709.80, 864.11, 995.27, 1041.56, 1273.02, 1342,46, 1519.91, 1712.79, 1743.65.

Bis(perfluorophenyl) (E)-5-((4-chloro-3-((1,3-dioxoisoindolin-2-yl) thio) but-3-en-1-yl) oxy) isophthalate (3q).

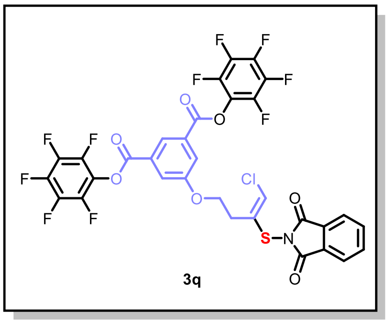

Yield: 85%; white solid; Mp 108 - 109 ºC; ^{1}H NMR (400 MHz, $CDCl_3$) δ 8.54 (s, 1H), 7.89 (s, 2H), 7.88 – 7.86 (m, 2H), 7.78 – 7.76 (m, 2H), 7.14 (s, 1H), 4.49 (t, *J* = 6.2 Hz, 2H), 2.97 (t, *J* = 6.2 Hz, 2H).; ^{13}C NMR (125 MHz, $CDCl_3$) δ 167.60, 161.25, 159.24, 142.33 – 142.20 (m, C_{Ar}-F), 140.92 – 140.19 (m, C_{Ar}-F), 139.14 – 136.88 (m, C_{Ar}-F), 134.90, 134.35, 131.72, 129.08, 128.85, 125.18 – 124.81 (m, C_{Ar}-O), 124.97, 124.09, 122.31, 65.62, 30.82; ^{19}F NMR (282 MHz, $CDCl_3$) δ -152.19 – -152.24 (m, 4F), -157.03 (t, *J* = 21.6 Hz, 2F), -161.70 – -161.80 (m, 4F).; HRMS (ESI) Calcd for : $C_{32}H_{12}ClF_{10}NO_7SNa$ [M+Na]$^+$ 801.9756, found 801.9754.; IR: 717.52, 794.67, 1033.85, 1273.02, 1519.91, 1743.65, 1766.80.

III. Characterization of Native Peptides.

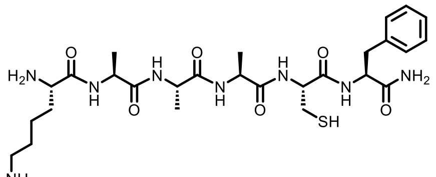

Peptide: **K**AAA**C**F-CONH2 **(**KF-6-2). HRMS (ESI) Calcd for $C_{27}H_{45}N_8O_6S$ $(M+H)^+$ 608.3167, found 608.3177.

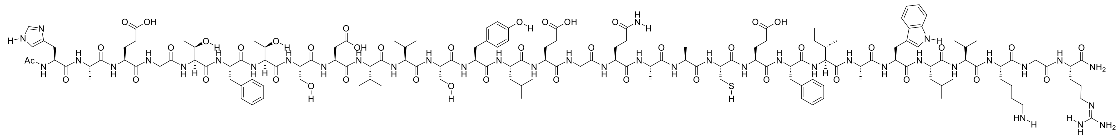

Peptide: Ac-HAEGTFTSDVVSYLEGQAA**C**EFIAWLV**K**GR-NH2 (**HR-30**). HRMS (ESI) Calcd for $C_{150}H_{228}N_{39}O_{45}S$ $(M+3H)^{3+}$ 1109.2151, found 1109.2147.

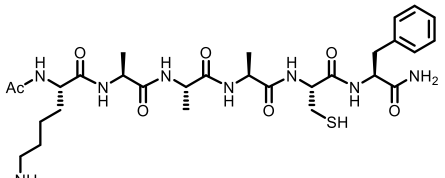

Peptide: Ac-**K**AAA**C**F-CONH2 **(**KF-6-1). HRMS (ESI) Calcd for $C_{29}H_{47}N_8O_7S$ $(M+H)^+$ 651.3283, found 651.3274.

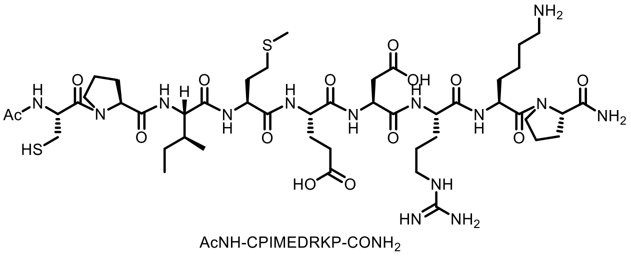

Peptide: Ac-CPIMEDRKP-CONH2 (CP-9). HRMS (ESI) Calcd for $C_{47}H_{80}N_{14}O_{14}S_2$ $[M+H]^+$ 1129.5493, found 1129.5494.

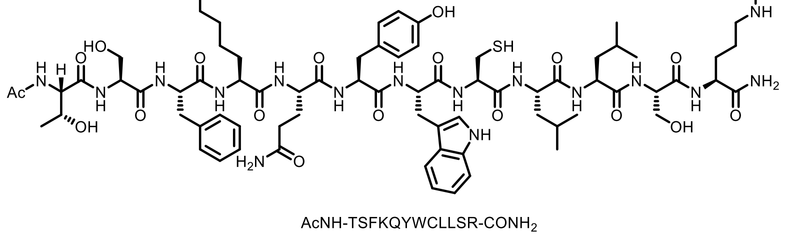

Peptide: Ac-TSFKQYWCLLSR-CONH2 (TR-12). HRMS (ESI) Calcd for

$C_{73}H_{110}N_{19}O_{18}S$ $[M+H]^+$ 1572.7991, found 1572.7993.

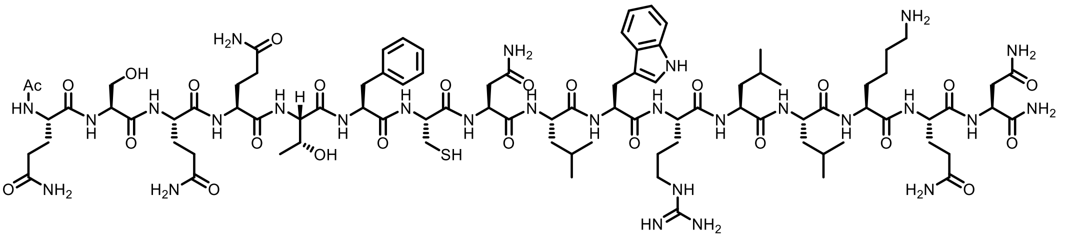

AcNH-QSQQTFCNLWRLLKQN-CONH$_2$

Peptide: Ac-QSQQTFCNLWRLLKQN-CONH$_2$ (QN-16-1). HRMS (ESI) Calcd for $C_{90}H_{145}N_{28}O_{25}S$ $[M+2H]^{2+}$ 1024.5284, found 1024.5282.

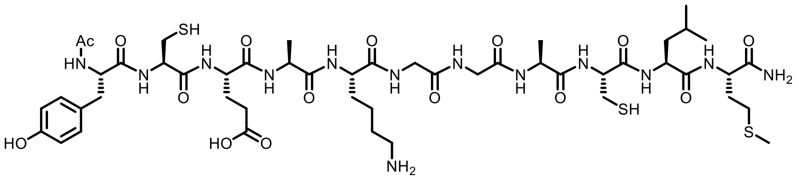

AcNH-YCEAKGGACLM-CONH$_2$

Peptide: Ac-YCEAKGGACLM-CONH$_2$ (YM-11). HRMS (ESI) Calcd for $C_{49}H_{80}N_{13}O_{15}S_3$ $[M+H]^+$ 1186.5053, found 1186.5055.

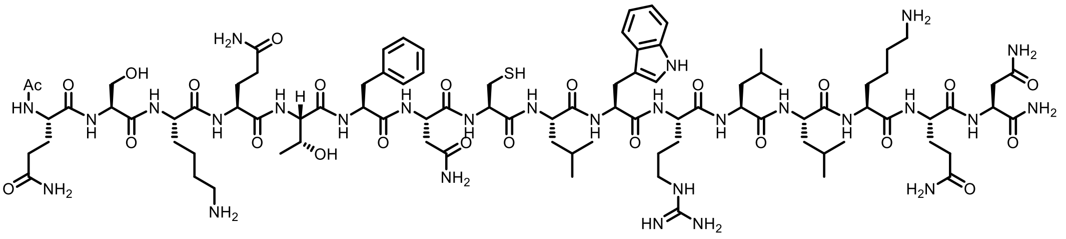

AcNH-QSKQTFNCLWRLLKQN-CONH$_2$

Peptide Ac-QSKQTFNCLWRLLKQN-CONH$_2$ (QN-16-2). HRMS (ESI) Calcd for $C_{91}H_{148}N_{28}O_{24}S$ $[M+H]^+$ 1024.5465, found 1024.5463.

LC-Chromatogram of native peptides

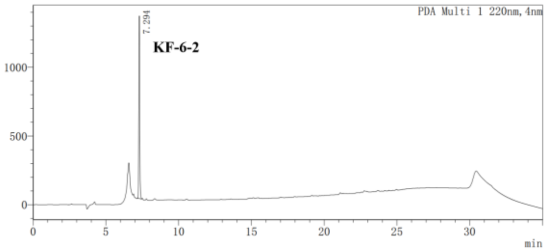

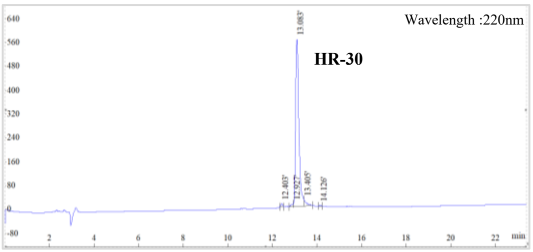

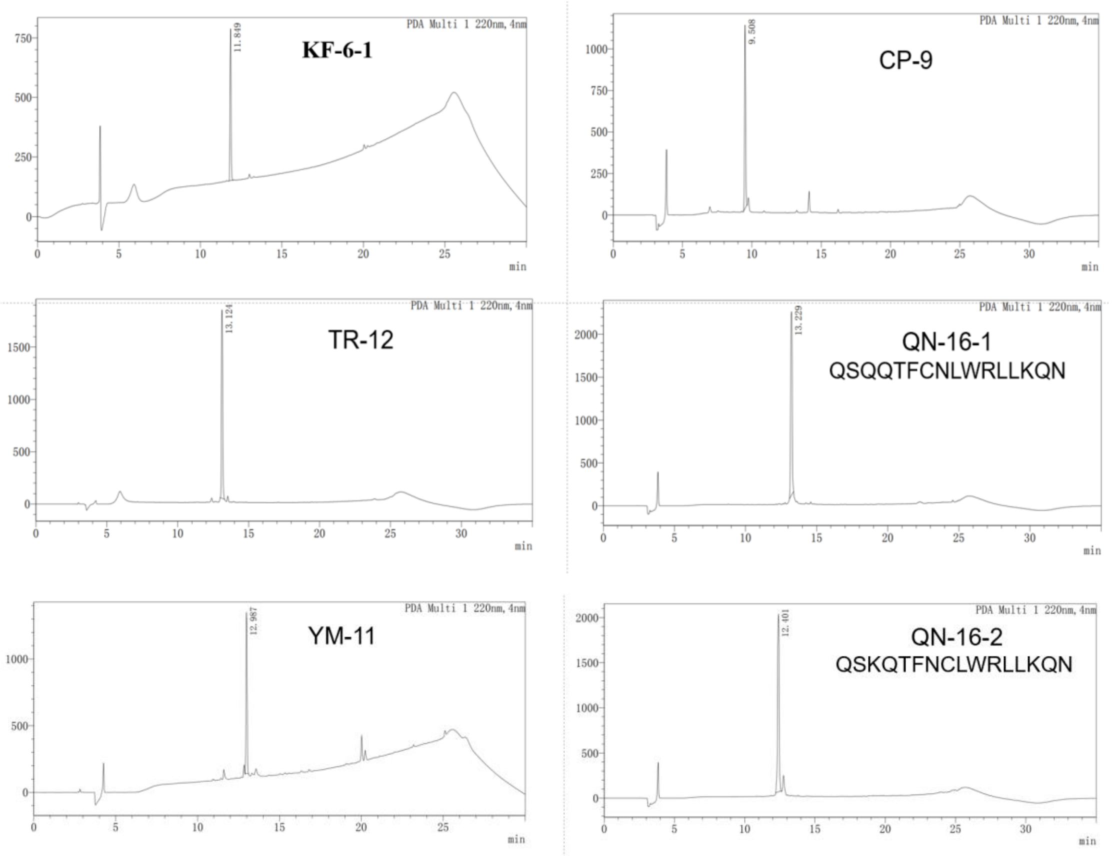

Optimization and the Ratio of Acetonitrile and Water by the LC-MS.

1.Screening the equivalents of reagent 3b.

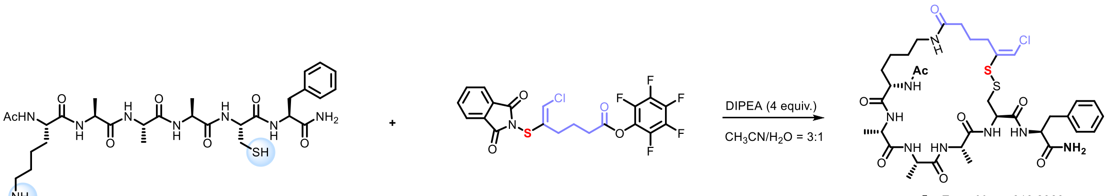

Ac-KAAACF-NH2   **3b**, X eq.   **5c**, Exact Mass: 810.2960

To a pre-mixed solution containing 100 μL of **KF-6-1** (prepared in deionized water at 1.0 μmol/mL, 1.0 equiv.) and 200 μL of acetonitrile (MeCN), X μL of **3b** (prepared in acetonitrile at 1.5 μmol/mL) was added dropwise under controlled stirring. After 1 minute of reaction, DIPEA (4.0 equiv.) was added, and the mixture was allowed to react at room temperature for 30 minutes, with reaction progress monitored in real time via LC-MS.

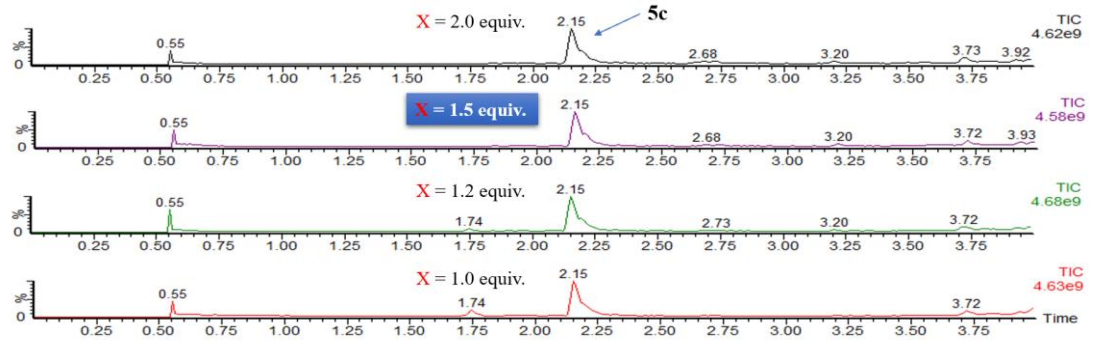

Gradient exploration of **3b** (1.0 equiv., 1.2 equiv., 1.5 equiv., 2.0 equiv.), the minimum dosage of **3b** was determined to be 1.5 equivalent for stapling.

2. Screening the ratio of deionized water to acetonitrile.

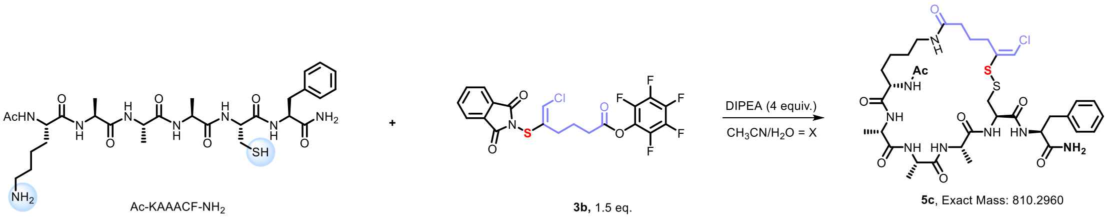

To a pre-mixed solution containing 100 µL of **KF-6-1** (prepared in deionized water at 1.0 µmol/mL, 1.0 equiv.) and X µL of acetonitrile (MeCN), 100 µL of **3b** (prepared in acetonitrile at 1.5 µmol/mL, 1.5 equiv.) was added dropwise under controlled stirring. After 1 minute of reaction, DIPEA (4.0 equiv.) was added, and the mixture was allowed to react at room temperature for 30 minutes, with reaction progress monitored in real time via LC-MS.

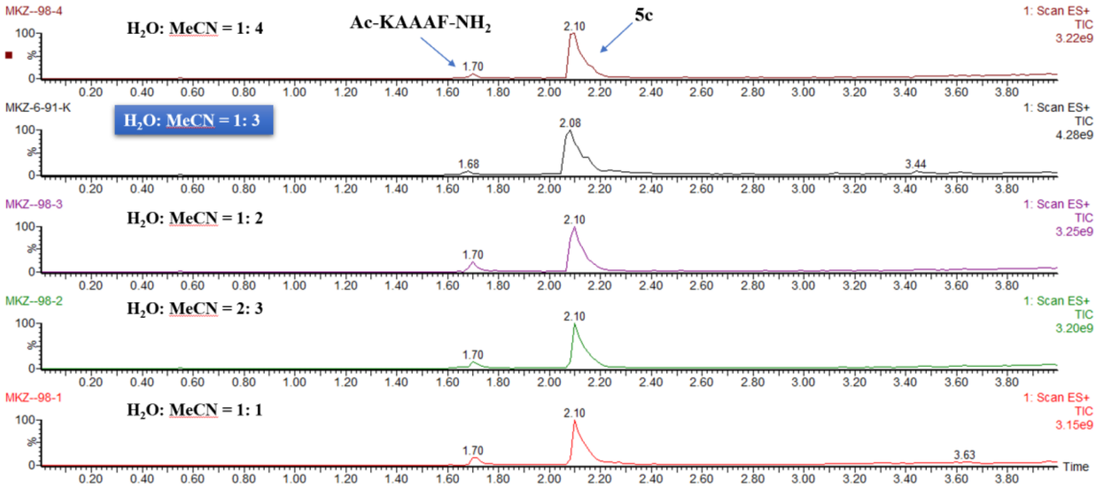

The ratio of H2O to MeCN varied from 1:1, 2:3, 1:2, 1:3, 1:4; Among these, the reaction effect was optimal when the ratio of H2O to MeCN was 1:3.

3. Screening the equivalents of DIPEA.

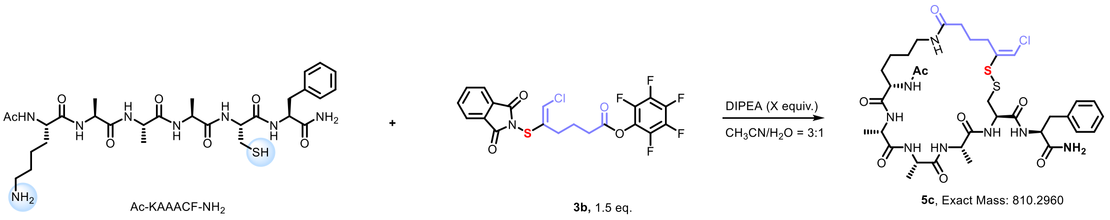

To a pre-mixed solution containing 100 µL of **KF-6-1** (prepared in deionized water at 1.0 µmol/mL, 1.0 equiv.) and 200 µL of acetonitrile (MeCN), 100 µL of **3b** (prepared in acetonitrile at 1.5 µmol/mL, 1.5 equiv.) was added dropwise under controlled stirring. After 1 minute of reaction, DIPEA (X equiv.) was added, and the mixture was allowed to react at room temperature for 30 minutes, with reaction progress monitored in real time via LC-MS.

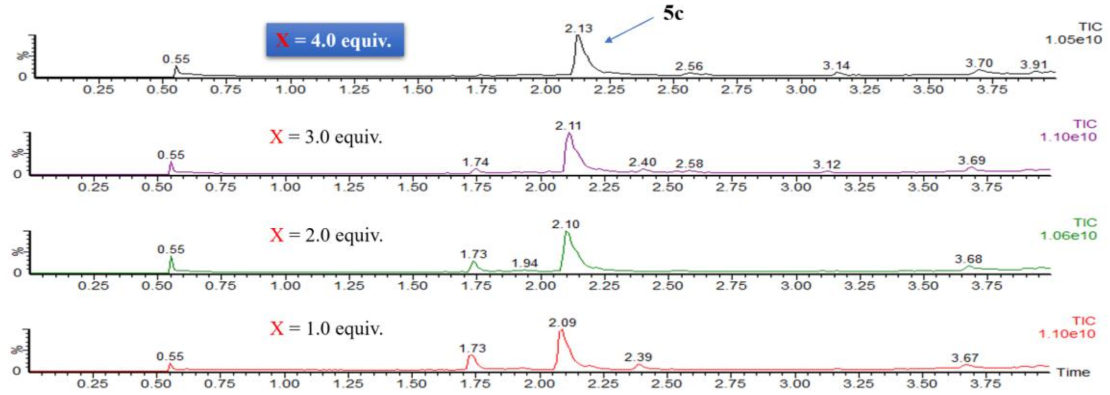

Gradient exploration of DIPEA (1.0 equiv., 2.0 equiv., 3.0 equiv., 4.0 equiv.), the best dosage of DIPEA was determined to be 4.0 equivalent for stapling.

4. Screening the reaction time for complete formation of stapled peptide 5c.

To a pre-mixed solution containing 100 μL of **KF-6-1** (prepared in deionized water at 1.0 μmol/mL, 1.0 equiv.) and 200 μL of acetonitrile (MeCN), 100 μL of **3b** (prepared in acetonitrile at 1.5 μmol/mL, 1.5 equiv.) was added dropwise under controlled stirring. After 1 minute of reaction, DIPEA (4.0 equiv.) was added, and the mixture was allowed to react at room temperature for 1-60 minutes, with reaction progress monitored in real time via LC-MS.

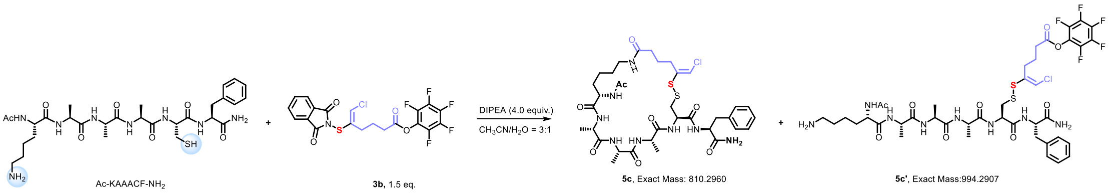

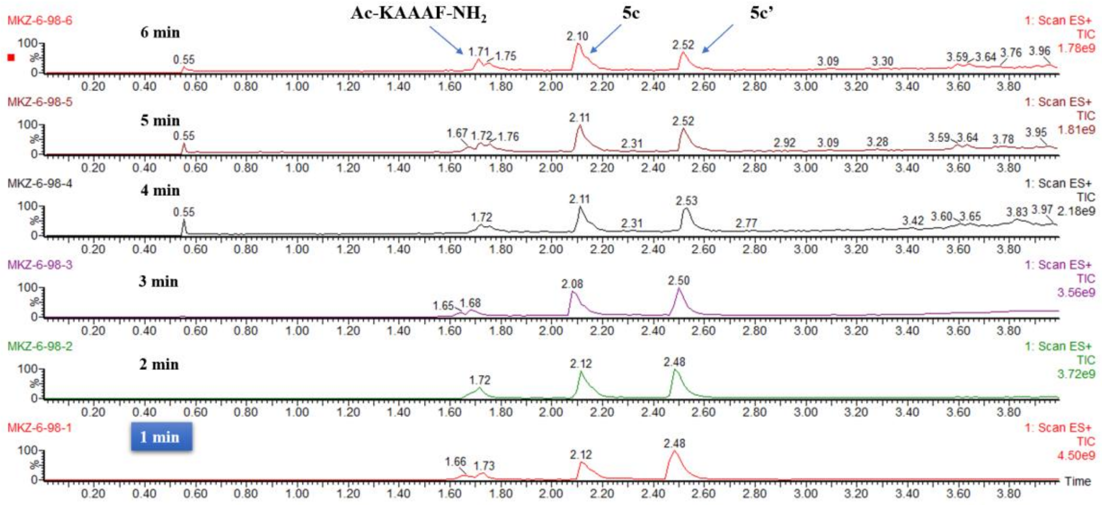

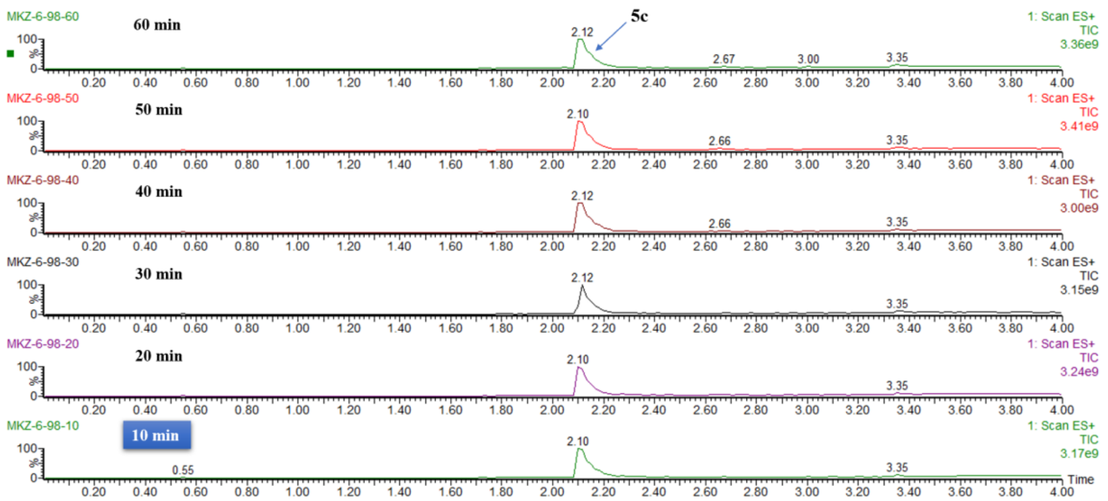

The product **5c** was fully formed within 10 minutes of reaction, and no further increase in yield was observed upon extending the reaction time.

Subsequently, we explored the simultaneous addition of reagent **3b** (100 μL) and DIPEA (4.0 equiv.) to pre-mixed solution containing 100 μL of **KF-6-1** and 200 μL of acetonitrile (MeCN) solution, and HPLC analysis confirmed that the yield of the cyclized peptide **5c** remained unchanged. We suspected that the fast disulfide click process was quantitative and specific, thereby enabling a one-step dual-stapling protocol within the same yield via adding **3b** and DIPEA together to the peptide KF-6-1.

IV. General Procedure and Data for Stapling Peptides 5.[5]

Analytical HPLC method information

SHIMADZU LC-20AT Liquid Chromatograph (InertSustain C18 Column, 4.6×250 mm, 5μm) and SHIMADZU LC-20AR Liquid Chromatograph (Shim-pack GIS C18 Column, 20×250 mm, 5μm). Water (solvent A) and acetonitrile (solvent B), each containing 0.1% TFA, were used as the mobile phase, at a flow rate of 1 mL/min. The gradient was programmed as A-phase follows:

Time (min)	Flow Rate (mL/min)	A%	B%
0.01	1	100-X	X
10	1	50	50
20	1	10	90
25	1	10	90
30	1	95	5
35	1	95	5

Preparative HPLC method information

Preparative RP-HPLC was performed on an Agilent 1260 Infinity (Agilent SB- semi-preparative C18 column, 9.4×250 mm, 5μm). Water (solvent A) and acetonitrile (solvent B), each containing 0.1% TFA, were used as the mobile phase, at a flow rate of 3 $mL.min^{-1}$. The gradient was programmed as A-phase follows:

Time (min)	Flow Rate (mL/min)	A%	B%
0	3	100-X	X
10-15	3	50	50
20	3	10	90
25	3	10	95
30	3	95	5
35	3	95	5

Method A: X = 5; **Method B**: X = 10; **Method C**: X = 20; **Method D**: X = 30;

General Procedure for the synthesis of stapled peptide 5.

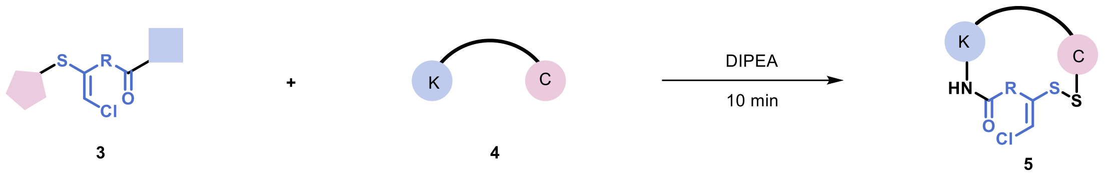

To a solution of **4** (3 or 5 μmol, 1.0 equiv.) in H_2O (1.0 mL) and 2.0 mL CH_3CN, then **3** (4.5 or 7.5 μmol, 1.5 equiv.) was dissolved in 1 mL CH_3CN adding dropwise to the solution. Then add DIPEA and react at room temperature for 10 min (analytical HPLC monitoring). After filtration, the mixture was directly injected into a preparative RP-HPLC (Method A-E) and lyophilized to afford the stapled products **5**. (analytical HPLC monitoring).

Examples of the construction of monocyclic peptides.

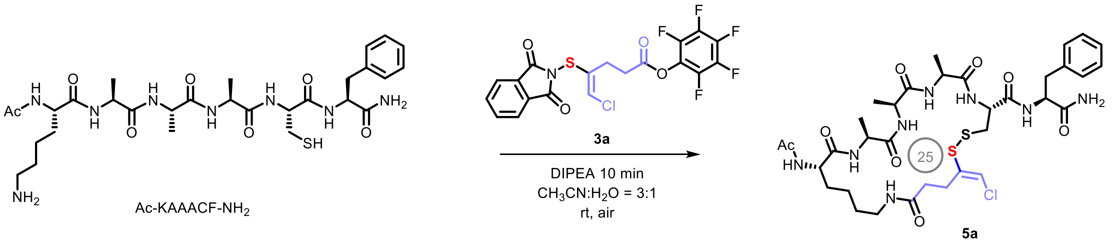

A solution of unstapled-peptide Ac-KAAACF-NH_2 (3.3 mg, 5.0 μmol, 1.00 equiv.) in H_2O (1.0 mL) and 2.0 mL CH_3CN, then **3a** (3.58 mg, 7.5 μmol, 1.5 equiv.), was dissolved in 1 mL CH_3CN adding dropwise to the solution. Then adding DIPEA (4.0 equiv.) and react at room temperature for 10 min (analytical HPLC monitoring). The mixture was directly injected into a preparative RP-HPLC (Method B) and lyophilized to afford the stapled product **5a** (2.9 mg, 72%).

Examples of the construction of dicyclopeptides 5ab.

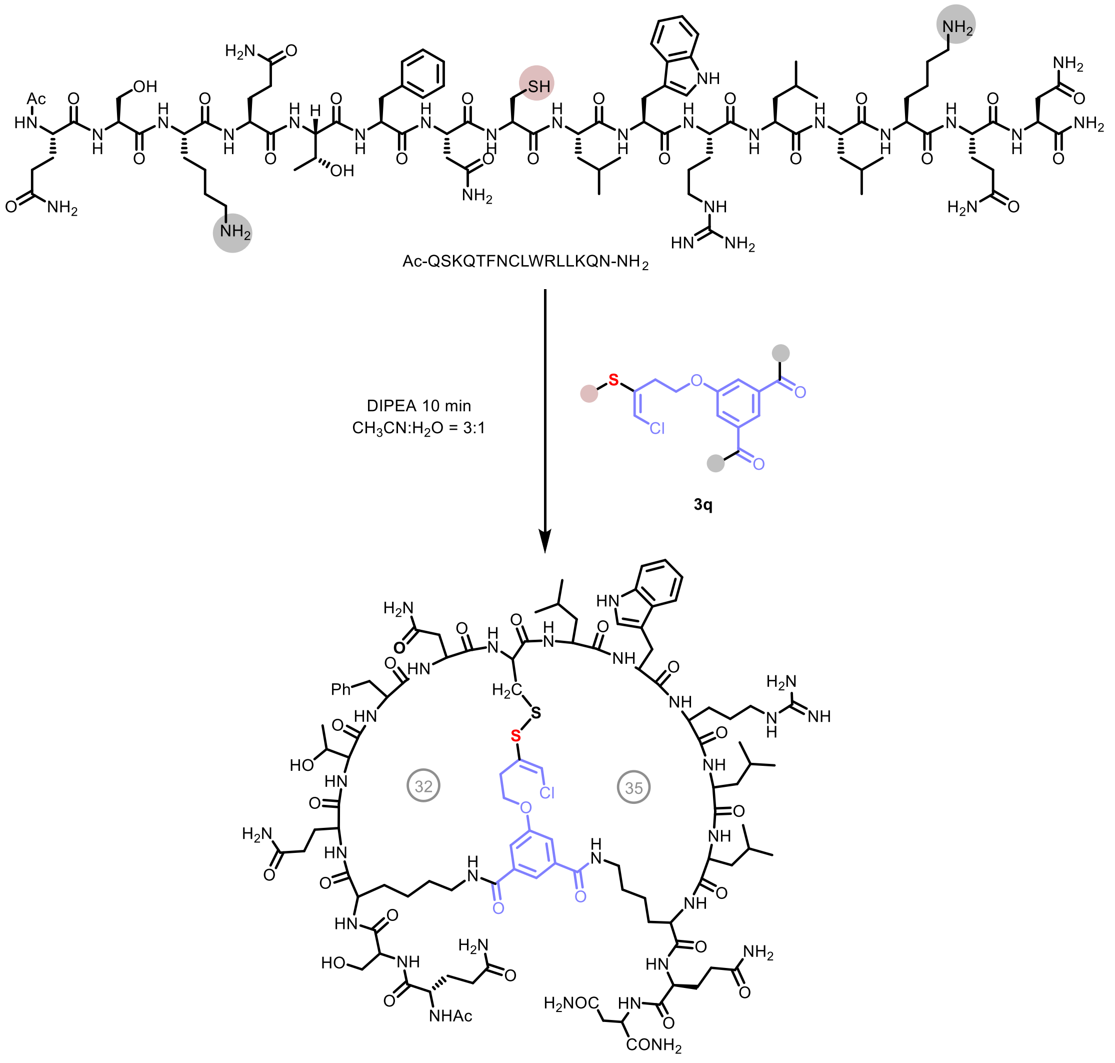

5ab, 72%

A solution of unstapled-peptide Ac-QSKQTFNCLWRLLKQN-CONH2 (3 μmol, 1.0 equiv.) in H_2O (1.0 mL) and 2.0 mL CH_3CN, then **3q** (4.5 μmol, 1.5 equiv.) was dissolved in 1 mL CH_3CN adding dropwise to the solution. Then adding DIPEA (8.0 equiv.) and react at room temperature for 10 min (analytical HPLC monitoring). The mixture was directly injected into a preparative RP-HPLC (Method C) and lyophilized to afford the stapled product **5ab** (5.0 mg, 72%).

Characterization of Stapling Peptides 5.

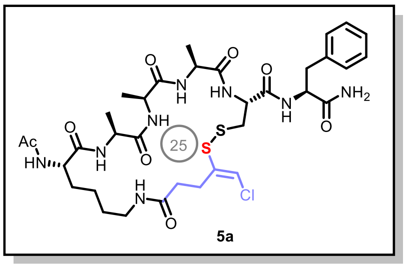

Peptide **5a**: Peptide **Ac-KAAACF-NH2** (KF-6-1) (3.2 mg, 5 μmol) with reagent **3a** (1.5 equiv.) and DIPEA (4.0 equiv.) was subjected to the general phase-transfer protocol to construct **5a**. The crude reaction mixture was purified by reverse-phase HPLC (Method A) (gradient 5-50% organic over 15 min) to give the stapled **5a** (2.9 mg, 72%). HRMS (ESI) Calcd for $C_{34}H_{50}ClN_8O_8S_2$ $[M+H]^+$ 797.2876, found 797.2870.

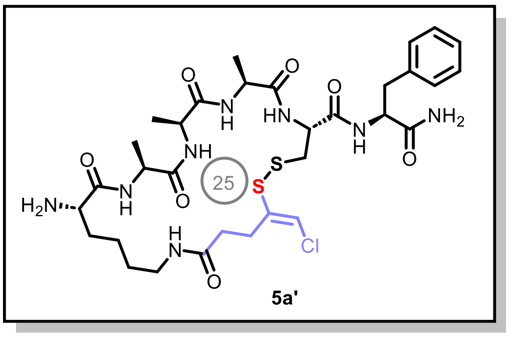

Peptide **5a'**: Peptide **KAAACF-NH2** (KF-6-2) (5 mg, 8.2 μmol) and DIPEA (4.0 equiv.) with reagent **3a** (1.5 equiv.) was subjected to the general phase-transfer protocol to construct **5a'**. The crude reaction mixture was purified by reverse-phase HPLC (Method C) (gradient 20-50% organic over 12 min) to give the stapled **5a'** (4.2 mg, 68%). HRMS (ESI) Calcd for $C_{32}H_{48}ClN_8O_7S_2$ $[M+H]^+$ 755.2705, found 755.2703.

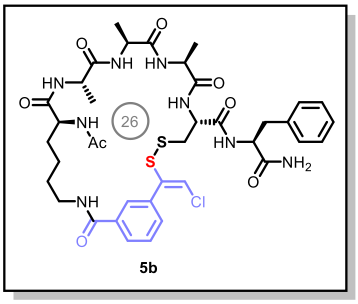

Peptide **5b**: Peptide **Ac-KAAACF-NH2** (KF-6-1) (3.2 mg, 5 μmol) with reagent **3j** (1.5 equiv.) and DIPEA (4.0 equiv.) was subjected to the general phase-transfer protocol to construct **5b**. The crude reaction mixture was purified by reverse-phase HPLC (Method A) (gradient 5-50% organic over 15 min) to give the stapled **5b** (3.0 mg, 72%). HRMS (ESI) Calcd for $C_{38}H_{50}ClN_8O_8S_2$ $[M+H]^+$ 845.2876, found 845.2867.

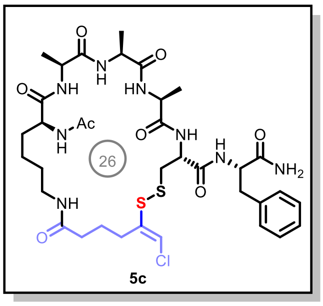

Peptide **5c**: Peptide **Ac-KAAACF-NH$_2$** (KF-6-1) (3.2 mg, 5 μmol) with reagent **3b** (1.5 equiv.) and DIPEA (4.0 equiv.) was subjected to the general phase-transfer protocol to construct **5c**. The crude reaction mixture was purified by reverse-phase HPLC (Method A) (gradient 5-50% organic over 15 min) to give the stapled **5c** (3.1 mg, 76%). HRMS (ESI) Calcd for $C_{35}H_{52}ClN_8O_8S_2$ $[M+H]^+$ 811.3033, found 811.3025.

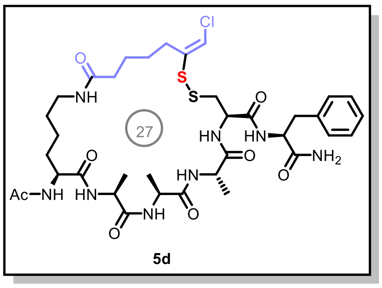

Peptide **5d**: Peptide **Ac-KAAACF-NH$_2$** (KF-6-1) (3.2 mg, 5 μmol) with reagent **3c** (1.5 equiv.) and DIPEA (4.0 equiv.) was subjected to the general phase-transfer protocol to construct **5d**. The crude reaction mixture was purified by reverse-phase HPLC (Method A) (gradient 5-50% organic over 15 min) to give the stapled **5d** (3.3 mg, 79%). HRMS (ESI) Calcd for $C_{36}H_{54}ClN_8O_8S_2$ $[M+H]^+$ 825.3189, found 825.3195.

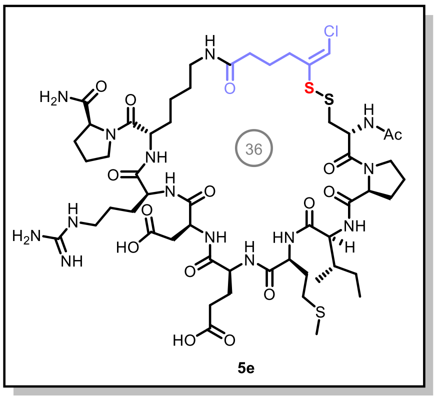

Peptide **5e**: Peptide **Ac- CPIMEDRKP -NH$_2$** (CP-9) (5.6 mg, 5 μmol) with reagent **3b** (1.5 equiv.) and DIPEA (4.0 equiv.) was subjected to the general phase-transfer protocol to construct **5e**. The crude reaction mixture was purified by reverse-phase HPLC (Method A) (gradient 5-50% organic over 15 min) to give the stapled **5e** (5.2 mg, 81%). HRMS (ESI) Calcd for $C_{53}H_{86}ClN_{14}O_{15}S_3$ $[M+H]^+$ 1289.5242, found 1289.5235.

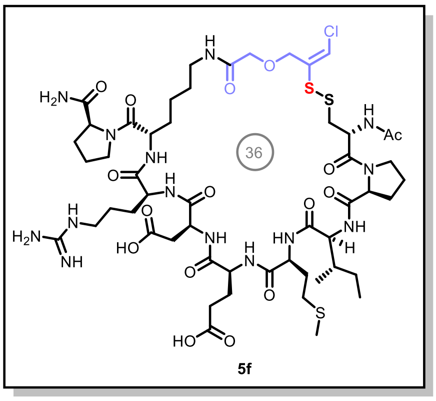

Peptide **5f**: Peptide **Ac- CPIMEDRKP -NH₂** (CP-9) (5.6 mg, 5 μmol) with reagent **3f** (1.5 equiv.) and DIPEA (4.0 equiv.) was subjected to the general phase-transfer protocol to construct **5f**. The crude reaction mixture was purified by reverse-phase HPLC (Method A) (gradient 5-50% organic over 15 min) to give the stapled **5f** (4.8 mg, 75%). HRMS (ESI) Calcd for $C_{52}H_{84}ClN_{14}O_{16}S_3$ $[M+H]^+$ 1291.5035, found 1291.5035.

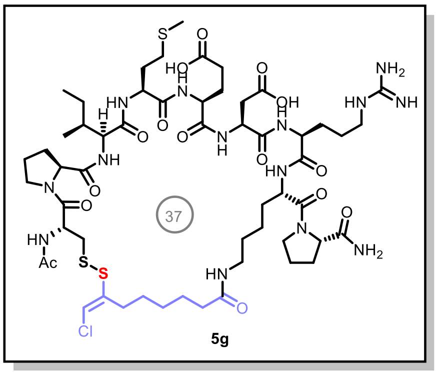

Peptide **5g**: Peptide **Ac-CPIMEDRKP-NH₂** (CP-9) (5.6 mg, 5 μmol) with reagent **3d** (1.5 equiv.) and DIPEA (4.0 equiv.) was subjected to the general phase-transfer protocol to construct **5g**. The crude reaction mixture was purified by reverse-phase HPLC (Method C) (gradient 20-50% organic over 15 min) to give the stapled **5g** (5.1 mg, 78%). HRMS (ESI) Calcd for $C_{55}H_{89}ClN_{14}O_{15}S_3$ $[M+H]^+$ 1317.5555, found 1317.5554.

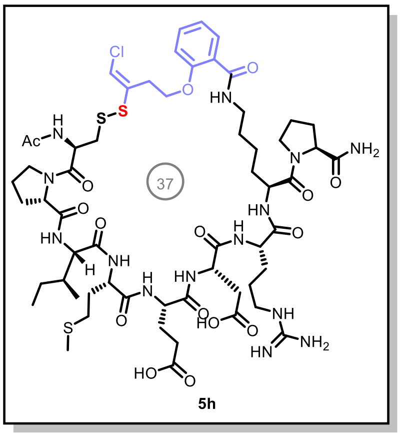

Peptide **5h**: Peptide **Ac-CPIMEDRKP-NH₂** (CP-9) (5.6 mg, 5 μmol) with reagent **3i** (1.5 equiv.) and DIPEA (4.0 equiv.) was subjected to the general phase-transfer protocol to construct **5h**. The crude reaction mixture was purified by reverse-phase HPLC (Method C) (gradient 20-50% organic over 15 min) to give the stapled **5h** (4.5 mg, 65%). HRMS (ESI) Calcd for $C_{58}H_{88}ClN_{14}O_{16}S_3$ $[M+H]^+$ 1367.5348, found 1367.5349.

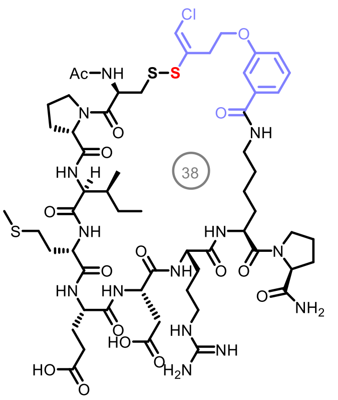

5i

Peptide **5i**: Peptide **Ac-CPIMEDRKP-NH$_2$** (CP-9) (5.6 mg, 5 μmol) with reagent **3k** (1.5 equiv.) and DIPEA (4.0 equiv.) was subjected to the general phase-transfer protocol to construct **5i**. The crude reaction mixture was purified by reverse-phase HPLC (Method C) (gradient 20-50% organic over 15 min) to give the stapled **5i** (4.9 mg, 72%). HRMS (ESI) Calcd for $C_{58}H_{88}ClN_{14}O_{16}S_3$ $[M+H]^+$ 1367.5348, found 1367.5342.

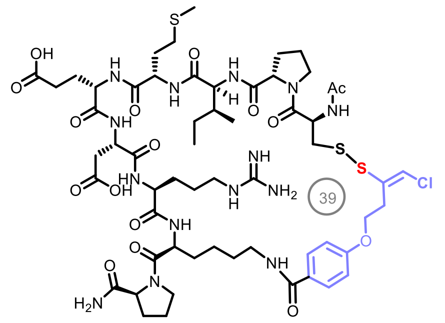

5j

Peptide **5j**: Peptide **Ac-CPIMEDRKP-NH$_2$** (CP-9) (5.6 mg, 5 μmol) with reagent **3m** (1.5 equiv.) and DIPEA (4.0 equiv.) was subjected to the general phase-transfer protocol to construct **5j**. The crude reaction mixture was purified by reverse-phase HPLC (Method A) (gradient 5-50% organic over 15 min) to give the stapled **5j** (4.2 mg, 62%). HRMS (ESI) Calcd for $C_{58}H_{88}ClN_{14}O_{16}S_3$ $[M+H]^+$ 1367.5348, found 1367.5348.

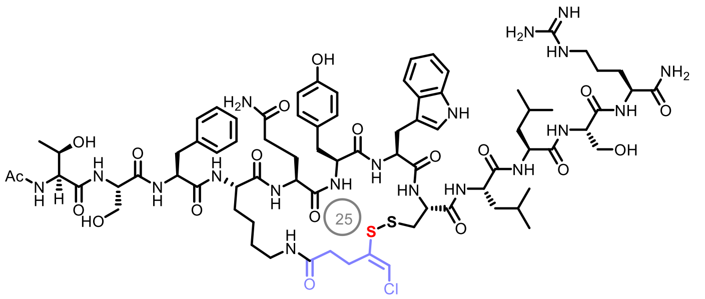

5k

Peptide **5k**: Peptide **Ac-TSFKQYWCLLSR-NH$_2$** (TR-12) (4.7 mg, 3 μmol) with reagent **3a** (1.5 equiv.) and DIPEA (4.0 equiv.) was subjected to the general phase-transfer protocol to construct **5k**. The crude reaction mixture was purified by reverse-phase HPLC (Method C) (gradient 20-50% organic over 12 min) to give the stapled **5k** (3.6 mg, 69%). HRMS (ESI) calcd for $C_{78}H_{113}ClN_{19}O_{19}S_2$ $[M+H]^+$ 1718.7585, found 1718.7585.

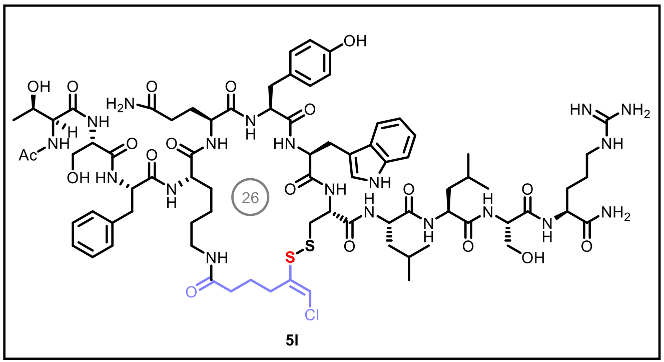

Peptide **5l**: Peptide **Ac-TSFKQYWCLLSR-NH₂** (TR-12) (4.7 mg, 3 µmol) with reagent **3b** (1.5 equiv.) and DIPEA (4.0 equiv.) was subjected to the general phase-transfer protocol to construct **5l**. The crude reaction mixture was purified by reverse-phase HPLC (Method C) (gradient 20-50% organic over 12 min) to give the stapled **5l** (4.4 mg, 85%). HRMS (ESI) Calcd for $C_{79}H_{115}ClN_{19}O_{19}S_2$ $[M+H]^+$ 1732.7741, found 1732.7749.

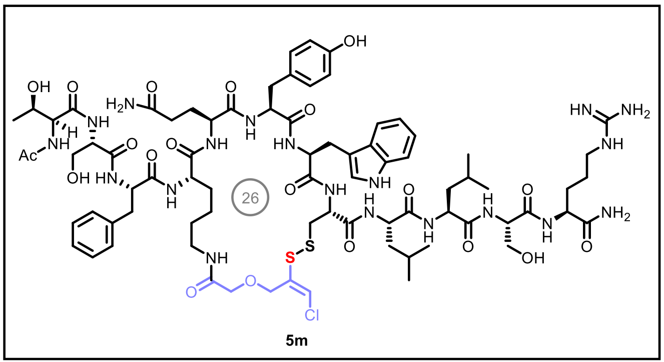

Peptide **5m**: Peptide **Ac-TSFKQYWCLLSR-NH₂** (TR-12), (4.7 mg, 3 µmol) with reagent **3f** (1.5 equiv.) and DIPEA (4.0 equiv.) was subjected to the general phase-transfer protocol to construct **5m**. The crude reaction mixture was purified by reverse-phase HPLC (Method C) (gradient gradient 20-50% organic over 15 min) to give the stapled **5m** (4.1 mg, 78%). HRMS (ESI) Calcd for $C_{78}H_{113}ClN_{19}O_{20}S_2$ $[M+H]^+$ 1734.7534, found 1734.7538.

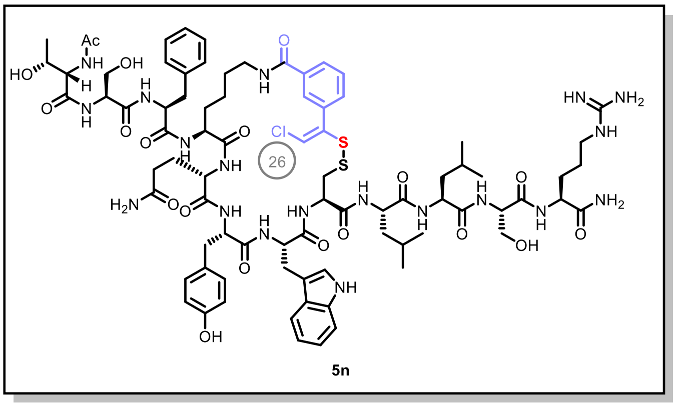

Peptide **5n**: Peptide **Ac-TSFKQYWCLLSR-NH₂** (TR-12), (4.7 mg, 3 µmol) with reagent **3j** (2.5 equiv.) and DIPEA (4.0 equiv.) was subjected to the general phase-transfer protocol to construct **5n**.

The crude reaction mixture was purified by reverse-phase HPLC (Method D) (gradient 30-50% organic over 12 min) to give the stapled **5n** (4.4 mg, 84%). HRMS (ESI) Calcd for $C_{82}H_{113}ClN_{19}O_{19}S_2$ $[M+H]^+$1766.7585, found 1766.7532.

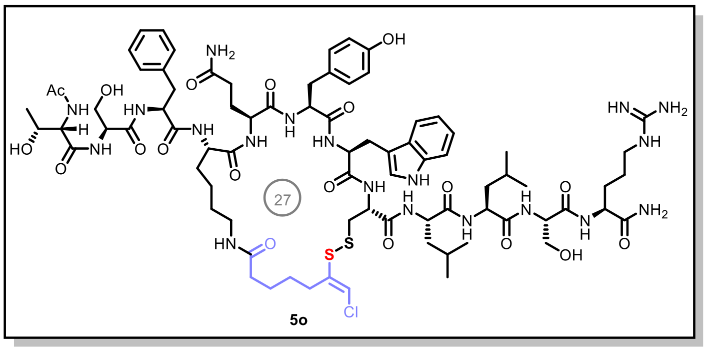
5o

Peptide **5o**: Peptide **Ac-TSFKQYWCLLSR-NH2** (TR-12) (4.7 mg, 3 µmol) and DIPEA (4.0 equiv.) with reagent **3c** (1.5 equiv.) was subjected to the general phase-transfer protocol to construct **5o**. The crude reaction mixture was purified by reverse-phase HPLC (Method C) (gradient 20-50% organic over 12 min) to give the stapled **5o** (4.0 mg, 76%). HRMS (ESI) Calcd for $C_{80}H_{117}ClN_{19}O_{19}S_2$ $[M+H]^+$ 1746.7898, found 1746.7894.

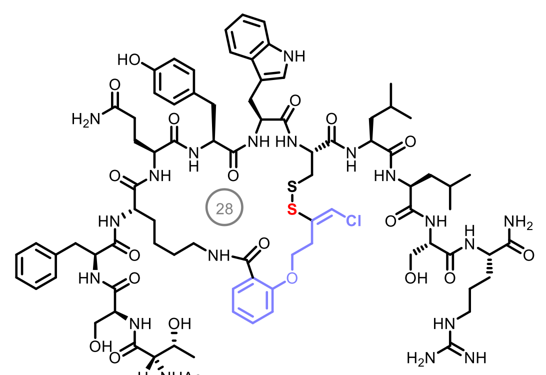
5p

Peptide **5p**: Peptide **Ac-TSFKQYWCLLSR-NH2** (TR-12), (4.7 mg, 3 µmol) with reagent **3i** (1.5 equiv.) and DIPEA (4.0 equiv.) was subjected to the general phase-transfer protocol to construct **5p**. The crude reaction mixture was purified by reverse-phase HPLC (Method C) (gradient 20-50% organic over 15 min) to give the stapled **5p** (4.5 mg, 82%). HRMS (ESI) Calcd for $C_{84}H_{117}ClN_{19}O_{20}S_2$ $[M+H]^+$ 1810.7847, found 1810.7851.

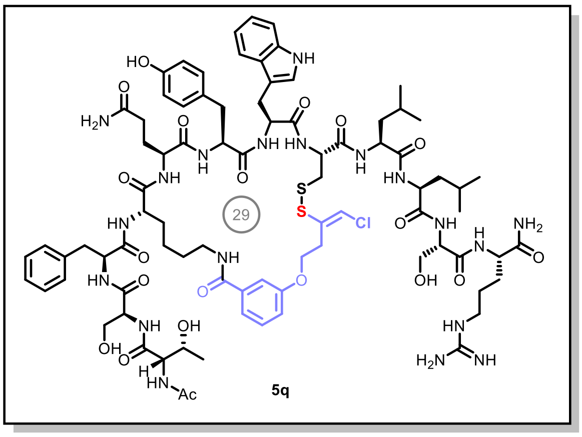

Peptide **5q**: Peptide **Ac-TSFKQYWCLLSR-NH₂** (TR-12), (4.7 mg, 3 μmol) with reagent **3k** (1.5 equiv.) and DIPEA (4.0 equiv.) was subjected to the general phase-transfer protocol to construct **5q**. The crude reaction mixture was purified by reverse-phase HPLC (Method C) (gradient 20-50% organic over 15 min) to give the stapled **5q** (4.6 mg, 85%). HRMS (ESI) Calcd for $C_{84}H_{117}ClN_{19}O_{20}S_2$ $[M+H]^+$ 1810.7847, found 1810.7848.

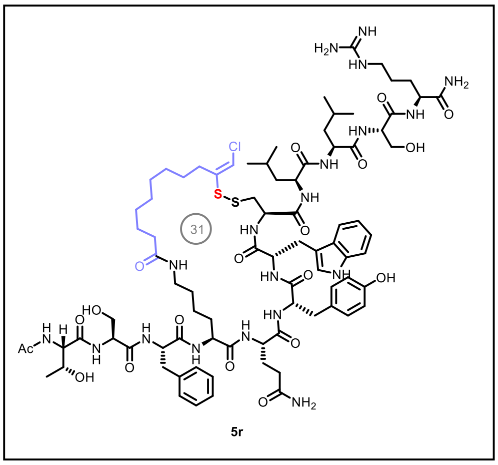

Peptide **5r**: Peptide **Ac-TSFKQYWCLLSR-NH₂** (TR-12) (4.7 mg, 3 μmol) with reagent **3e** (1.5 equiv.) and DIPEA (4.0 equiv.) was subjected to the general phase-transfer protocol to construct **5r**. The crude reaction mixture was purified by reverse-phase HPLC (Method C) (gradient 20-50% organic over 12 min) to give the stapled **5r** (3.7 mg, 68%). HRMS (ESI) Calcd for $C_{84}H_{125}ClN_{19}O_{19}S_2$ $[M+H]^+$ 1802.8524, found 1802.8556.

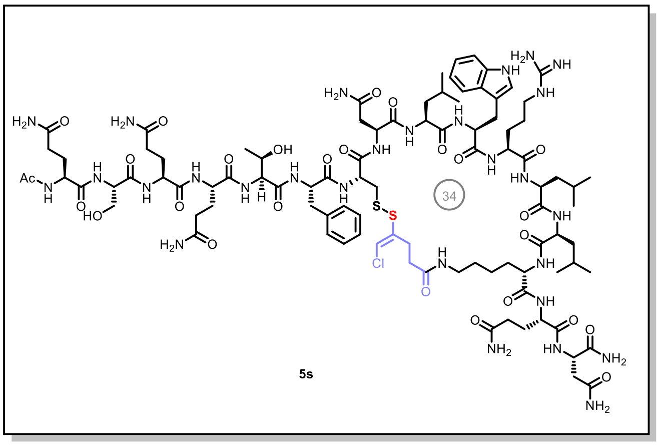

Peptide : **5s**: Peptide A **Ac-QSQQTFCNLWRLLKQN-NH₂** (QN-16) (6.1 mg, 3 μmol) with reagent **3a** (1.5 equiv.) and DIPEA (4.0 equiv.) was subjected to the general phase-transfer protocol to construct **5s**. The crude reaction mixture was purified by reverse-phase HPLC (Method C) (gradient 20-

50% organic over 12 min) to give the stapled **5s** (5.2 mg, 79%). HRMS (ESI) Calcd for $C_{96}H_{147}ClN_{28}O_{26}S_2$ $[M+2H]^{2+}$ 1097.5080, found 1097.5086.

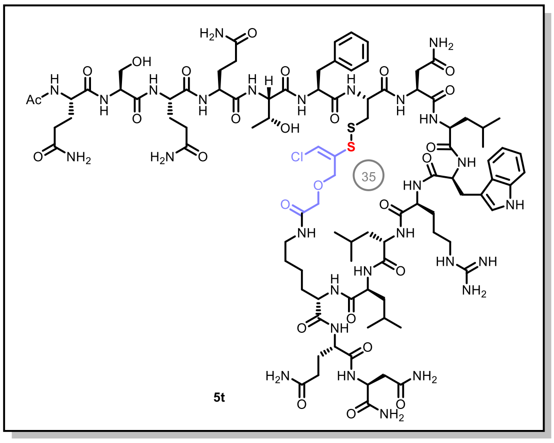

Peptide **5t**: Peptide : **Ac-QSQQTFCNLWRLLKQN-NH$_2$** (QN-16) (6.1 mg, 3 µmol) with reagent **3f** (1.5 equiv.) and DIPEA (4.0 equiv.) was subjected to the general phase-transfer protocol to construct **5t**. The crude reaction mixture was purified by reverse-phase HPLC (Method C) (gradient 20-50% organic over 12 min) to give the stapled **5t** (5.9 mg, 89%). HRMS (ESI) Calcd for $C_{95}H_{147}ClN_{28}O_{27}S_2$ $[M+2H]^{2+}$ 1105.5055, found 1105.5046.

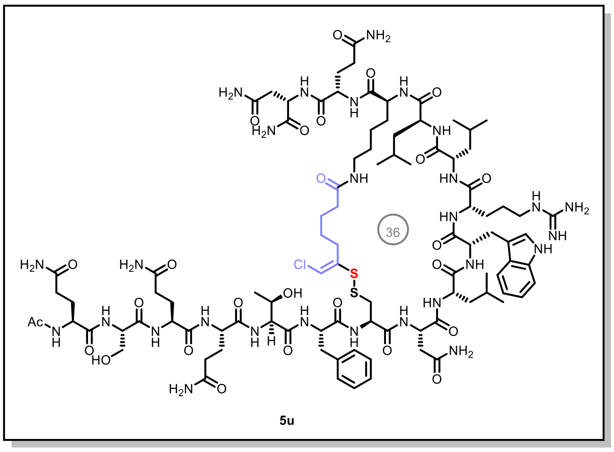

Peptide **5u**: Peptide: **Ac-QSQQTFCNLWRLLKQN-NH$_2$** (QN-16) (6.1 mg, 3 µmol) with reagent **3c** (1.5 equiv.) and DIPEA (4.0 equiv.) was subjected to the general phase-transfer protocol to construct **5u**. The crude reaction mixture was purified by reverse-phase HPLC (Method C) (gradient 20-50% organic over 12 min) to give the stapled **5u** (5.6 mg, 84%). HRMS (ESI) Calcd for $C_{97}H_{151}ClN_{28}O_{26}S_2$ $[M+2H]^{2+}$ 1111.5236, found 1111.5220.

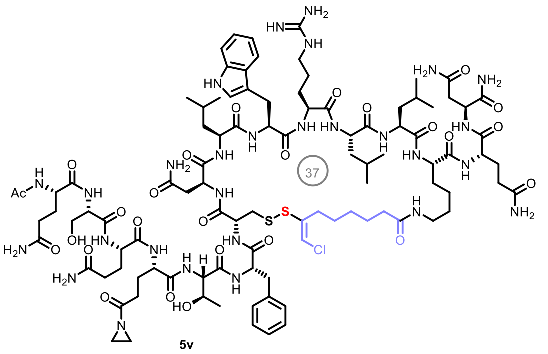

5v

Peptide **5v**: Peptide: **Ac-QSQQTFCNLWRLLKQN-NH2** (QN-16) (6.1 mg, 3 μmol) with reagent **3d** (1.5 equiv.) and DIPEA (4.0 equiv.) was subjected to the general phase-transfer protocol to construct **5v**. The crude reaction mixture was purified by reverse-phase HPLC (Method C) (gradient 20-50% organic over 12 min) to give the stapled **5v** (5.3 mg, 78%). HRMS (ESI) Calcd for $C_{100}H_{155}ClN_{28}O_{26}S_2$ $[M+2H]^{2+}$ 1131.5393, found 1131.5401.

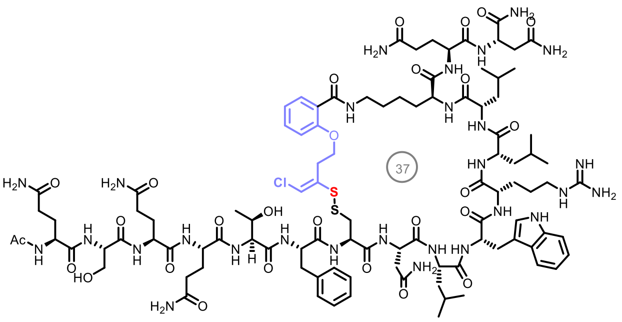

5w

Peptide **5w**: Peptide A **Ac-QSQQTFCNLWRLLKQN-NH2** (QN-16) (10.2 mg, 5 μmol) with reagent **3i** (1.5 equiv.) and DIPEA (4.0 equiv.) was subjected to the general phase-transfer protocol to construct **5w** The crude reaction mixture was purified by reverse-phase HPLC (Method C) (gradient 20-50% organic over 12 min) to give the stapled **5w** (7.4 mg, 65%). HRMS (ESI) calcd for $C_{101}H_{151}ClN_{28}O_{27}S_2$ $[M+2H]^{2+}$ 1143.5211, found 1143.5210.

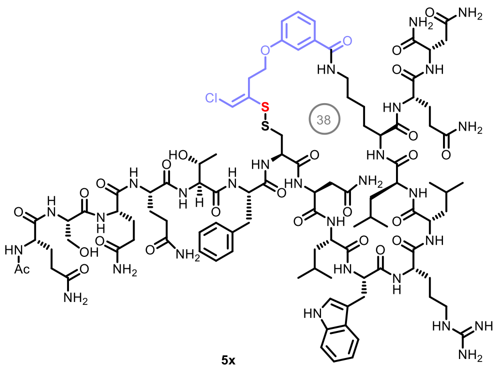

5x

Peptide **5x**: Peptide A **Ac-QSQQTFCNLWRLLKQN-NH2** (QN-16) (10.2mg, 5 μmol) with reagent **3k** (1.5 equiv.) and DIPEA (4.0 equiv.) was subjected to the general phase-transfer protocol to construct **5x**. The crude reaction mixture was purified by reverse-phase

HPLC (Method C) (gradient 20-50% organic over 12 min) to give the stapled **5x** (9.7 mg, 85%). HRMS (ESI) Calcd for $C_{101}H_{151}ClN_{28}O_{27}S_2$ $[M+2H]^{2+}$ 1143.5211, found 1143.5219.

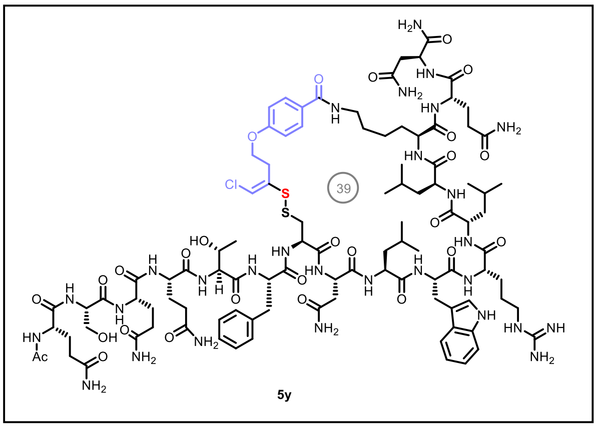

Peptide **5y**: Peptide A **Ac-QSQQTFCNLWRLLKQN-NH2** (QN-16) (10.2 mg, 5 μmol) with reagent **3m** (1.5 equiv.) and DIPEA (4.0 equiv.) was subjected to the general phase-transfer protocol to construct **5y**. The crude reaction mixture was purified by reverse-phase HPLC (Method C) (gradient 20-50% organic over 12 min) to give the stapled **5y** (7.7 mg, 68%). HRMS (ESI) Calcd for $C_{101}H_{151}ClN_{28}O_{27}S_2$ $[M+2H]^{2+}$ 1143.5211, found 1143.5219.

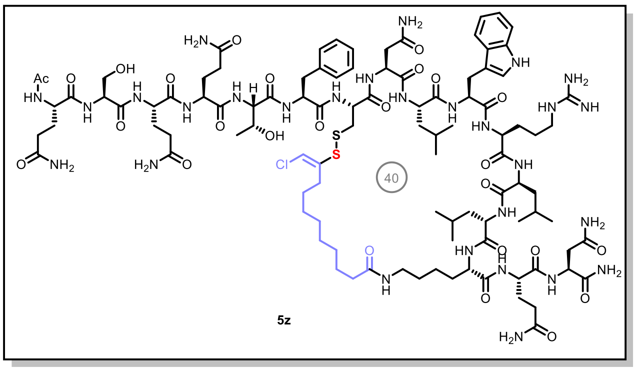

Peptide **5z**: Peptide A **Ac-QSQQTFCNLWRLLKQN-NH2** (QN-16) (6.7mg, 3 μmol) with reagent **3e** (1.5 equiv.) and DIPEA (4.0 equiv.) was subjected to the general phase-transfer protocol to construct **5z**. The crude reaction mixture was purified by reverse-phase HPLC (Method C) (gradient 20-50% organic over 12 min) to give the stapled **5z** (5.1 mg, 75%). HRMS (ESI) Calcd for $C_{101}H_{159}ClN_{28}O_{26}S_2$ $[M+2H]^{2+}$ 1139.5549, found 1139.5557.

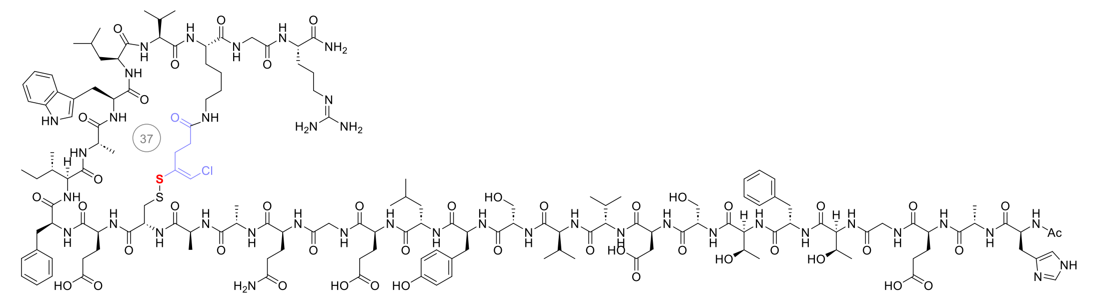
5aa

Peptide **5aa**: Peptide **Ac-HAEGTFTSDVVSYLEGQAACEFIAWLVKGR-NH2** (**HR-30**) (4.99 mg, 1.5 μmol) with reagent **3a** (1.5 equiv.) and DIPEA (4.0 equiv.) was subjected to the general phase-transfer protocol to construct **5aa**. The crude reaction mixture was purified by reverse-phase HPLC (Method C) (gradient 20-50% organic over 15 min) to give the stapled peptide **5aa** (3.1mg, 60%). HRMS (ESI) Calcd for $C_{155}H_{230}ClN_{39}O_{46}S_2$ $[M+2H]^{2+}$ 1750.3151, found 1750.3264.

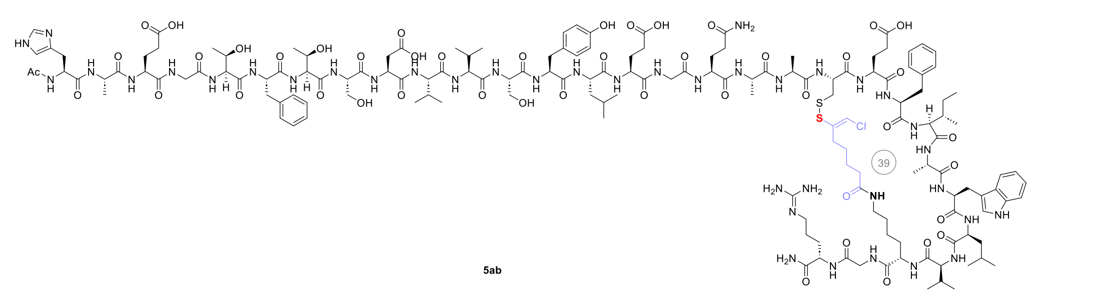
5ab

Peptide **5ab**: Peptide **Ac-HAEGTFTSDVVSYLEGQAACEFIAWLVKGR-NH2** (**HR-30**) (4.99 mg, 1.5 μmol) with reagent **3c** (1.5 equiv.) and DIPEA (4.0 equiv.) was subjected to the general phase-transfer protocol to construct **5ab**. The crude reaction mixture was purified by reverse-phase HPLC (Method C) (gradient 20-50% organic over 15 min) to give the stapled peptide **5ab** (4 mg, 76%). HRMS (ESI) Calcd for $C_{157}H_{235}ClN_{39}O_{46}S_2$ $[M+2H]^{2+}$ 1736.2993, found 1736.2883.

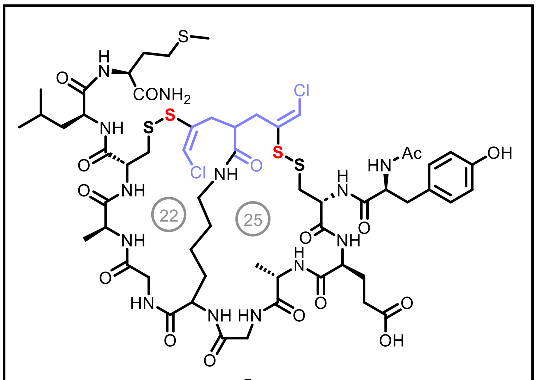

5ac

Peptide **5ac**: Peptide **Ac-YCEAKGGACLM-NH2** (YM-11) (3.5 mg, 3 µmol) with reagent **3n** (1.5 equiv.) and DIPEA (4.0 equiv.) was subjected to the general phase-transfer protocol to construct **5ac**. The crude reaction mixture was purified by reverse-phase HPLC (Method A) (gradient 20-50% organic over 12 min) to give the stapled **5ac** (2.5 mg, 57%). HRMS (ESI) calcd for $C_{57}H_{84}Cl_2N_{13}O_{16}S_5$ $[M+H]^+$ 1436.4134, found 1436.4114.

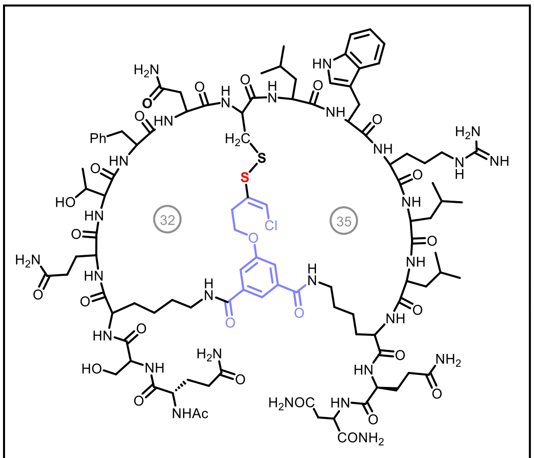

5ad

Peptide **5ad**: Peptide **Ac-QSKQTFNCLWRLLKQN-NH2** (QN-16-2) (6.1 mg, 3 µmol) with reagent **3q** (1.5 equiv.) and DIPEA (8.0 equiv.) was subjected to the general phase-transfer protocol to construct **5ad**. The crude reaction mixture was purified by reverse-phase HPLC (Method A) (gradient 8-50% organic over 10 min) to give the stapled **5ad** (5.0 mg, 72%). HRMS (ESI) Calcd for $C_{103}H_{153}ClN_{28}O_{27}S_2$ $[M+2H]^{2+}$ 1156.5290, found 1156.5295.

LC-Chromatogram for 5.

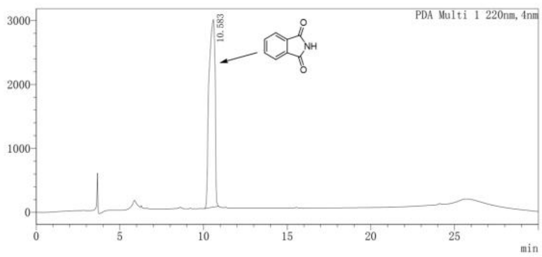

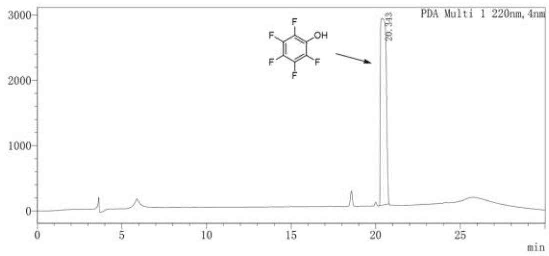

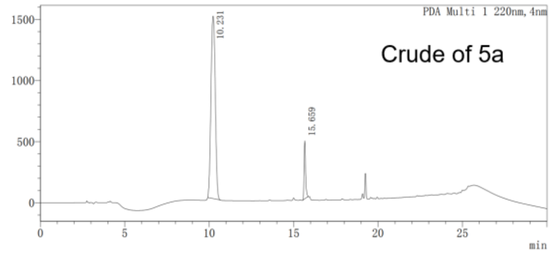
PDA Multi 1 220nm, 4nm
Crude of 5a
10.231
15.659
min

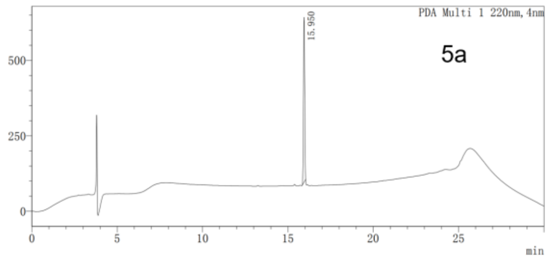
PDA Multi 1 220nm, 4nm
5a
15.950
min

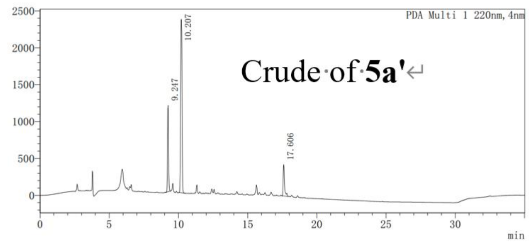
PDA Multi 1 220nm, 4nm
Crude of 5a'
10.207
9.247
17.606
min

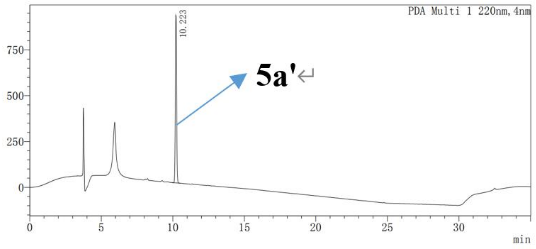
PDA Multi 1 220nm, 4nm
5a'
10.223
min

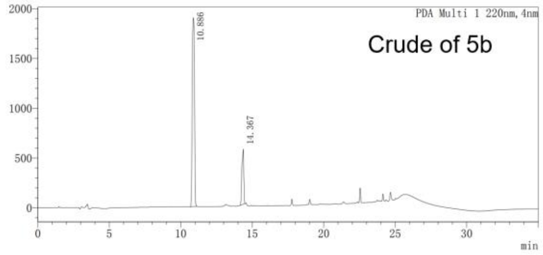
PDA Multi 1 220nm, 4nm
Crude of 5b
10.886
14.367
min

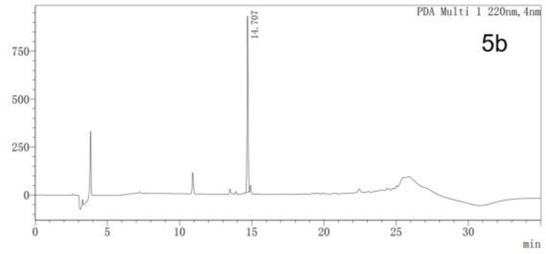
PDA Multi 1 220nm, 4nm
5b
14.707
min

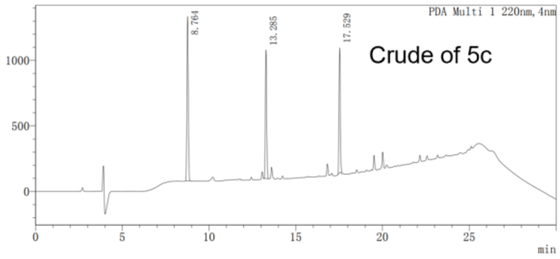
PDA Multi 1 220nm, 4nm
Crude of 5c
8.764
13.285
17.529
min

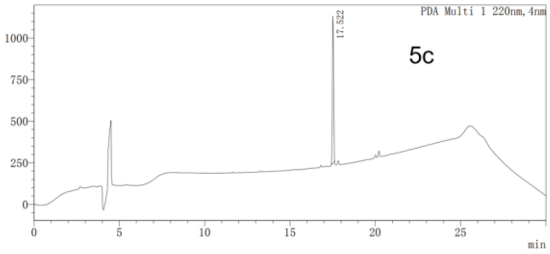
PDA Multi 1 220nm, 4nm
5c
17.522
min

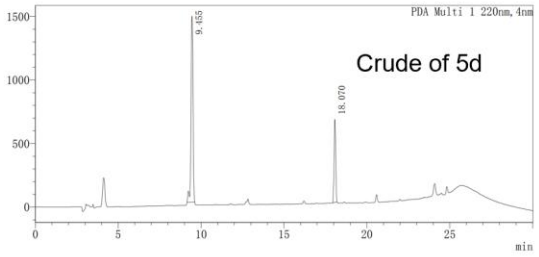
PDA Multi 1 220nm, 4nm
Crude of 5d
9.455
18.070
min

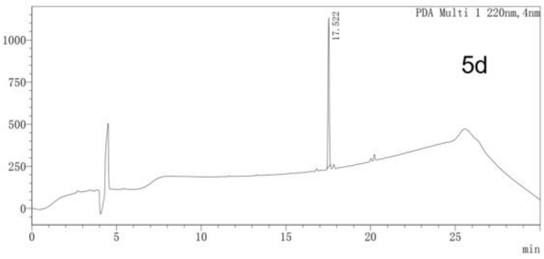
PDA Multi 1 220nm, 4nm
5d
17.522
min

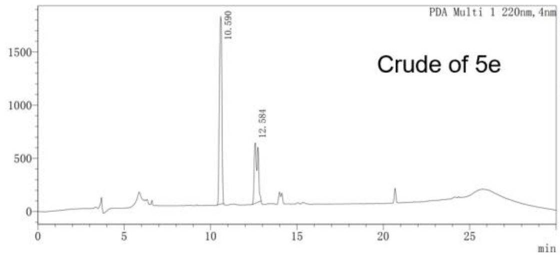
PDA Multi 1 220nm, 4nm
Crude of 5e
10.590
12.584
min

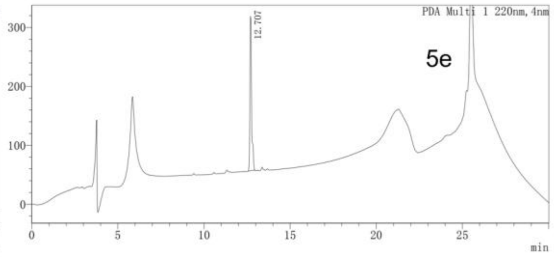
PDA Multi 1 220nm, 4nm
5e
12.707
min

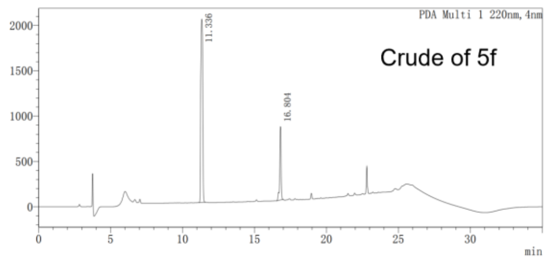
PDA Multi 1 220nm, 4nm
Crude of 5f
11.336
16.804
min

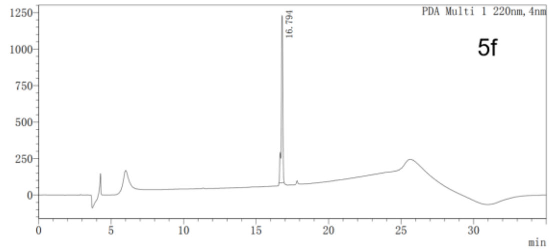
PDA Multi 1 220nm, 4nm
5f
16.794
min

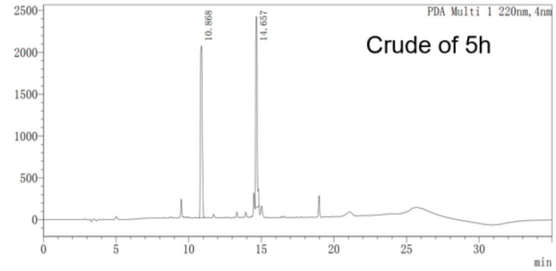
PDA Multi 1 220nm, 4nm
Crude of 5h
10.868
14.657
min

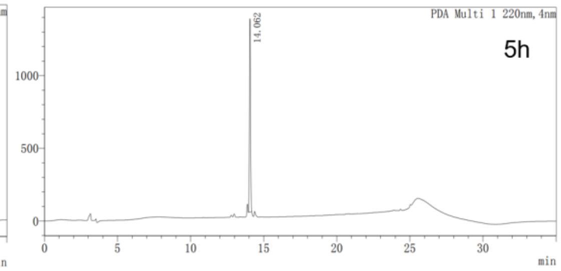
PDA Multi 1 220nm, 4nm
5h
14.062
min

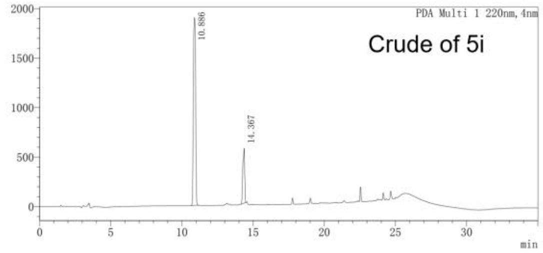
PDA Multi 1 220nm, 4nm
Crude of 5i
10.886
14.367
min

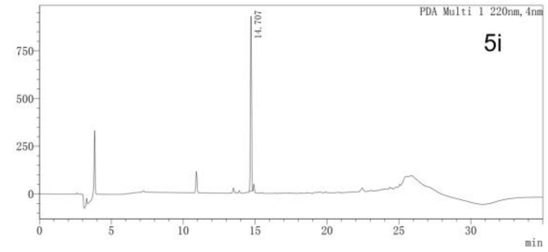
PDA Multi 1 220nm, 4nm
5i
14.707
min

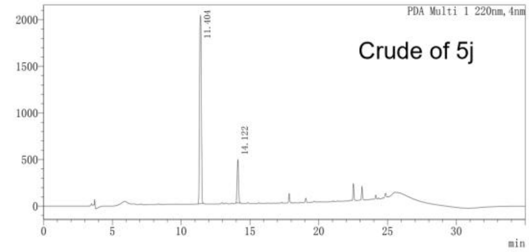
PDA Multi 1 220nm, 4nm
Crude of 5j
11.404
14.122
min

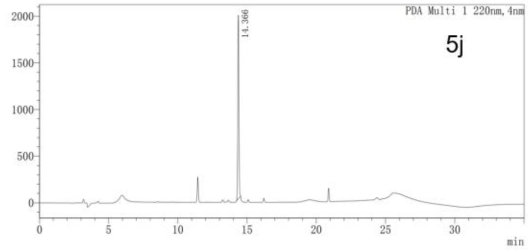
PDA Multi 1 220nm, 4nm
5j
14.366
min

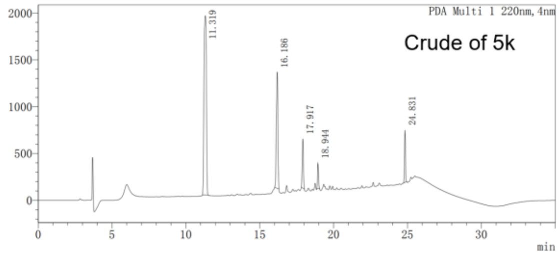
PDA Multi 1 220nm, 4nm
Crude of 5k
11.319
16.186
17.917
18.944
24.831
min

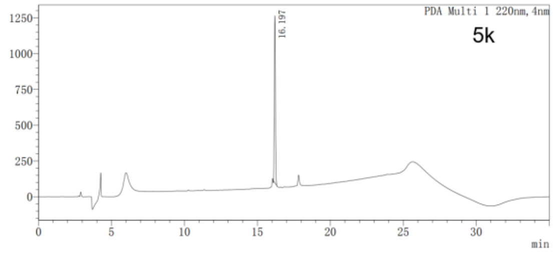
PDA Multi 1 220nm, 4nm
5k
16.197
min

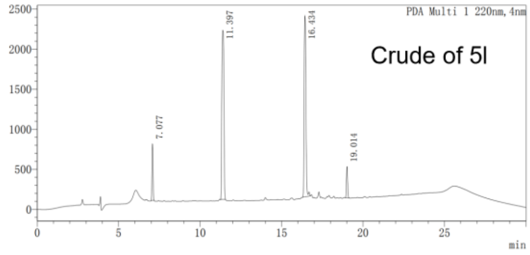
PDA Multi 1 220nm, 4nm
Crude of 5l
7.077
11.397
16.434
19.014
min

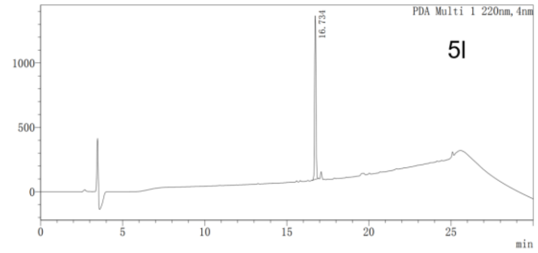
PDA Multi 1 220nm, 4nm
5l
16.734
min

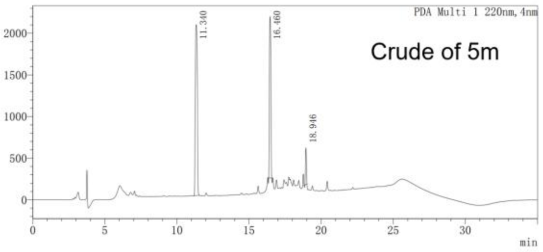
PDA Multi 1 220nm, 4nm
Crude of 5m
11.340
16.460
18.946
min

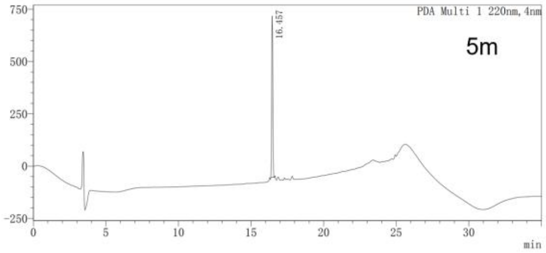
PDA Multi 1 220nm, 4nm
5m
16.457
min

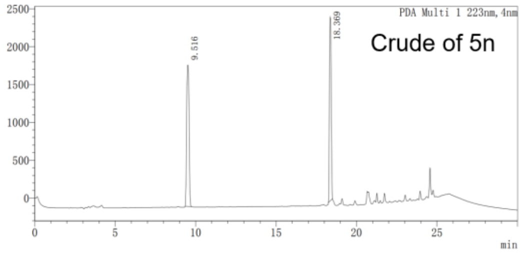
PDA Multi 1 223nm, 4nm
Crude of 5n
9.516
18.369
min

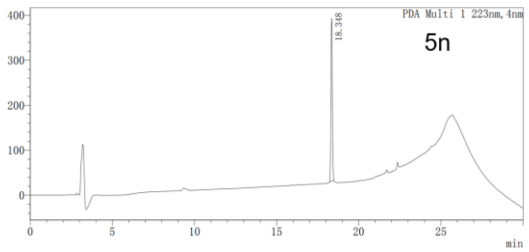
PDA Multi 1 223nm, 4nm
5n
18.348
min

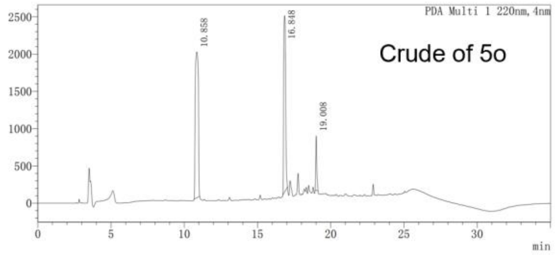
PDA Multi 1 220nm, 4nm
Crude of 5o
10.858
16.848
19.008
min

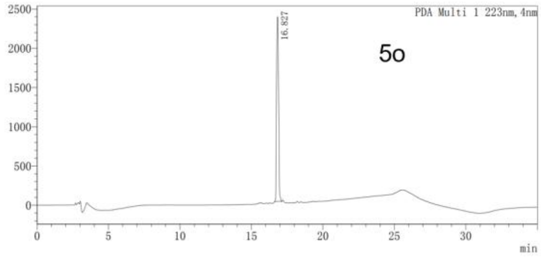
PDA Multi 1 223nm, 4nm
5o
16.827
min

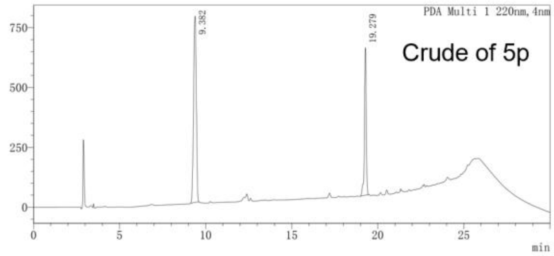
PDA Multi 1 220nm, 4nm
Crude of 5p
9.382
19.279
min

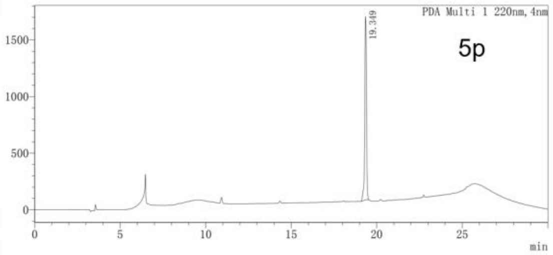
PDA Multi 1 220nm, 4nm
5p
19.349
min

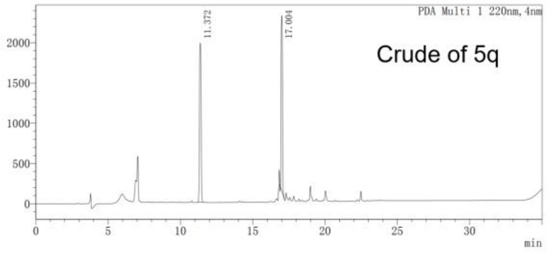
PDA Multi 1 220nm, 4nm
Crude of 5q
11.372
17.004
min

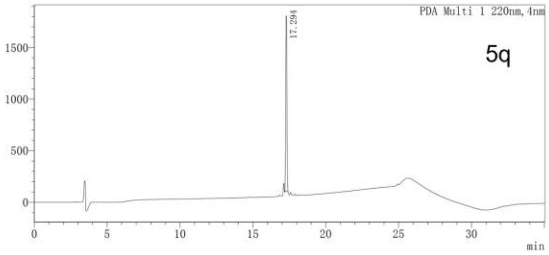
PDA Multi 1 220nm, 4nm
5q
17.294
min

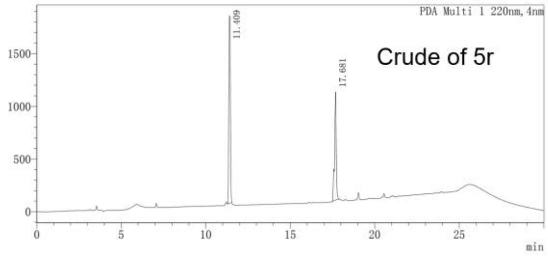
PDA Multi 1 220nm, 4nm
Crude of 5r
11.409
17.681
min

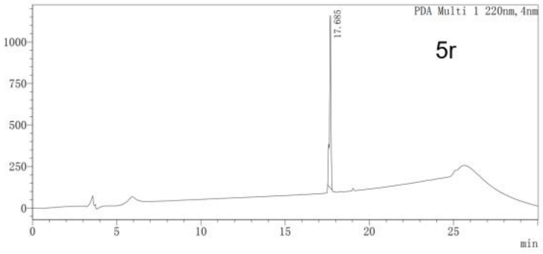
PDA Multi 1 220nm, 4nm
5r
17.685
min

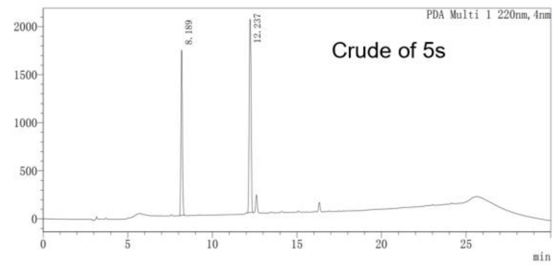
PDA Multi 1 220nm, 4nm
Crude of 5s
8.189
12.237
min

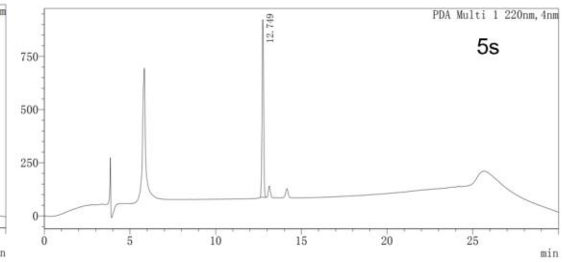
PDA Multi 1 220nm, 4nm
5s
12.749
min

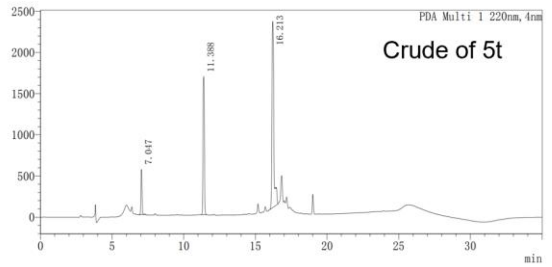
PDA Multi 1 220nm, 4nm
Crude of 5t
7.047
11.388
16.213
min

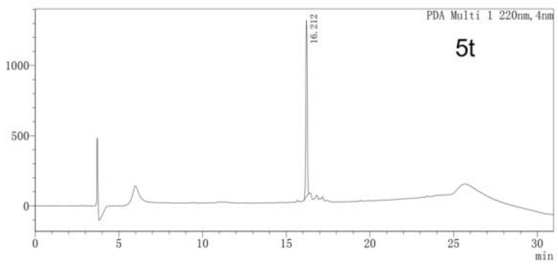
PDA Multi 1 220nm, 4nm
5t
16.212
min

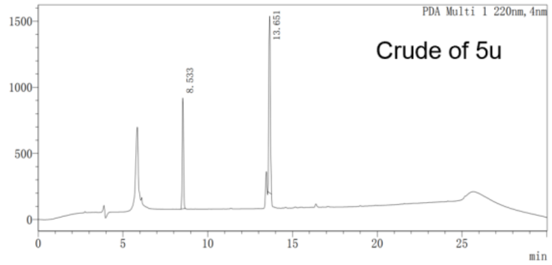
PDA Multi 1 220nm, 4nm
Crude of 5u
8.533
13.651
min

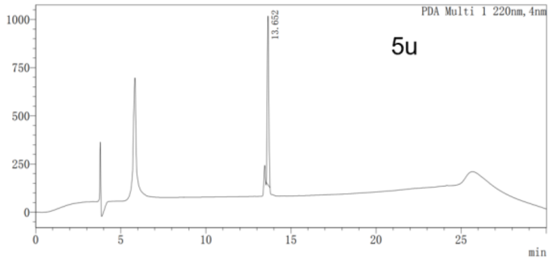
PDA Multi 1 220nm, 4nm
5u
13.652
min

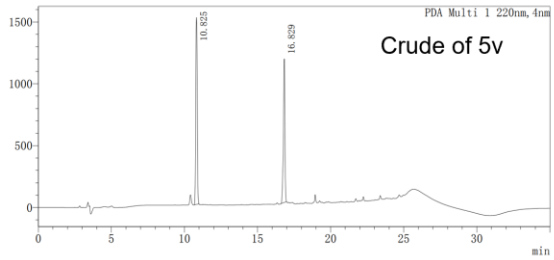
PDA Multi 1 220nm, 4nm
Crude of 5v
10.825
16.829
min

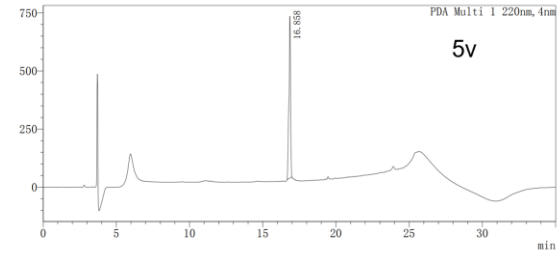
PDA Multi 1 220nm, 4nm
5v
16.858
min

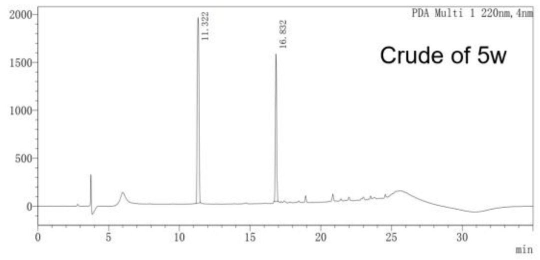
PDA Multi 1 220nm, 4nm
Crude of 5w
11.322
16.832
min

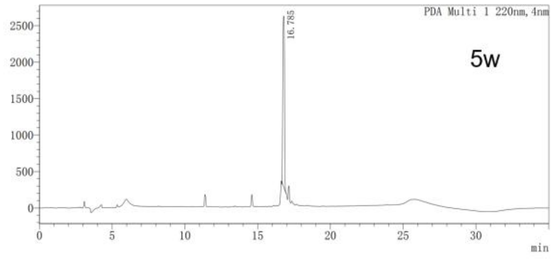
PDA Multi 1 220nm, 4nm
5w
16.785
min

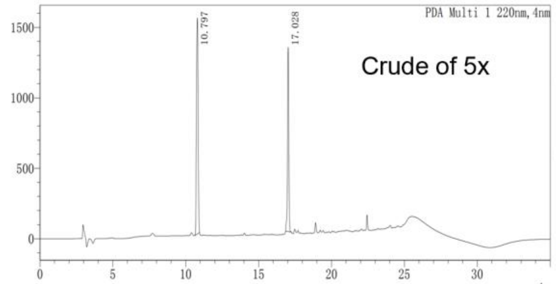
PDA Multi 1 220nm, 4nm
Crude of 5x
10.797
17.028
min

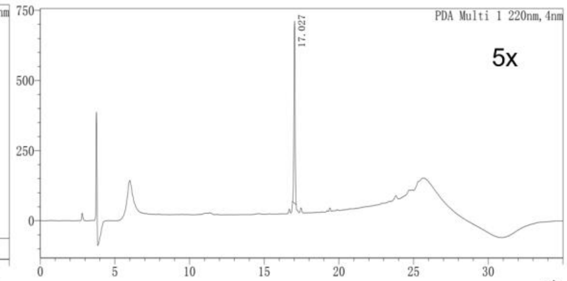
PDA Multi 1 220nm, 4nm
5x
17.027
min

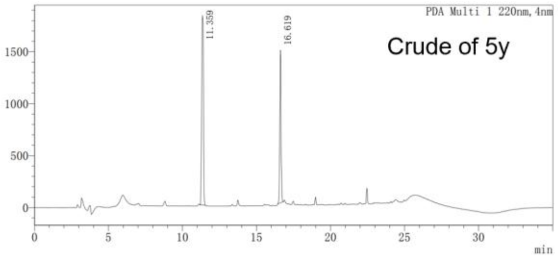
PDA Multi 1 220nm, 4nm
Crude of 5y
11.359
16.619
min

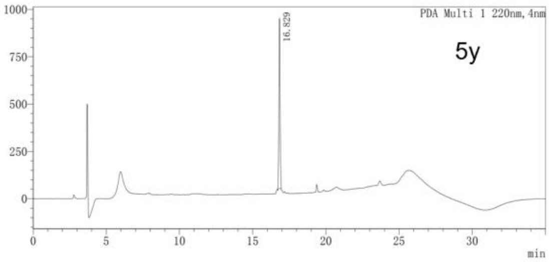
PDA Multi 1 220nm, 4nm
5y
16.829
min

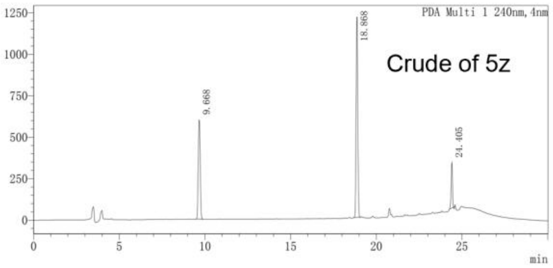
PDA Multi 1 240nm, 4nm
Crude of 5z
9.668
18.868
24.405
min

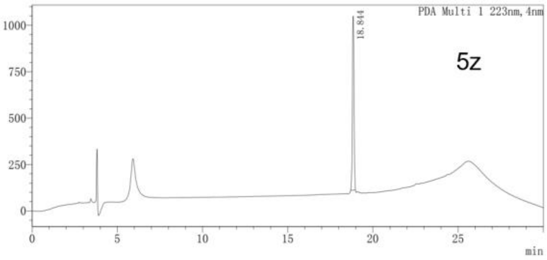
PDA Multi 1 223nm, 4nm
5z
18.844
min

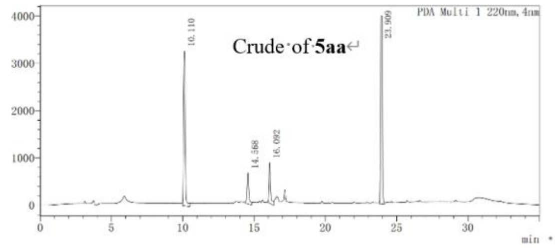
PDA Multi 1 220nm, 4nm
Crude of **5aa**
10.110
14.548
16.092
23.909
min

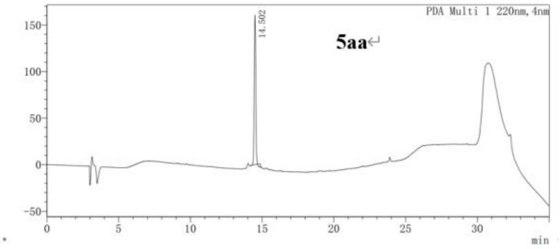
PDA Multi 1 220nm, 4nm
5aa
14.502
min

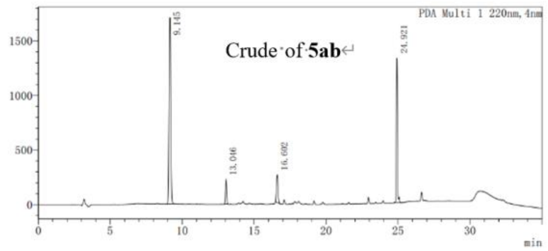
PDA Multi 1 220nm, 4nm
Crude of **5ab**
9.145
13.046
16.692
24.921
min

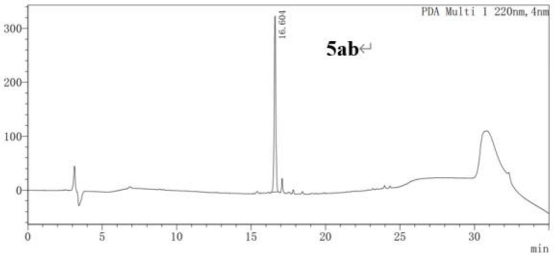
PDA Multi 1 220nm, 4nm
5ab
16.604
min

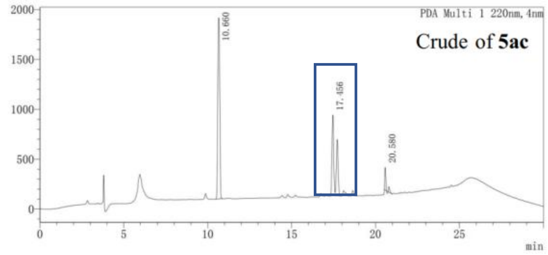
PDA Multi 1 220nm, 4nm
Crude of **5ac**
10.660
17.456
20.580
min

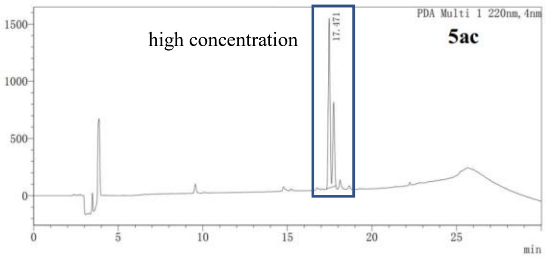
PDA Multi 1 220nm, 4nm
high concentration
5ac
17.471
min

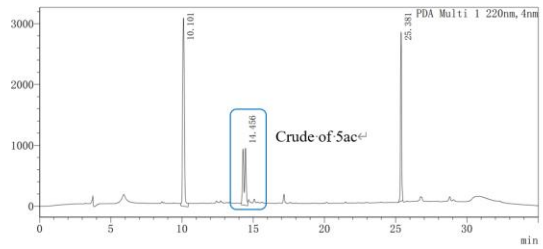
PDA Multi 1 220nm, 4nm
Crude of 5ac
10.101
14.456
25.381
min

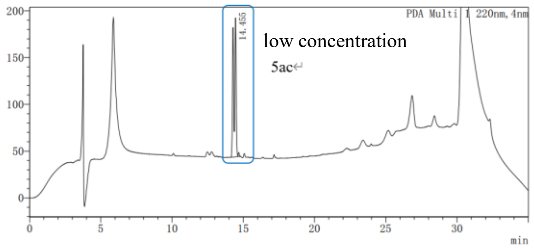
PDA Multi 1 220nm, 4nm
low concentration
5ac
14.455
min

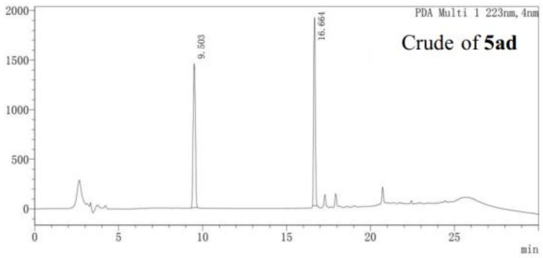

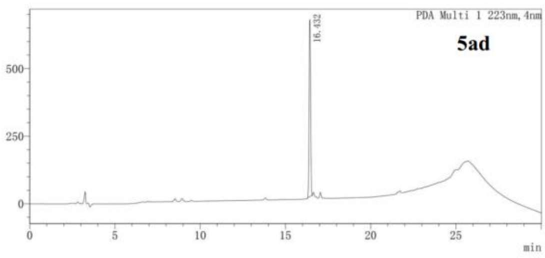

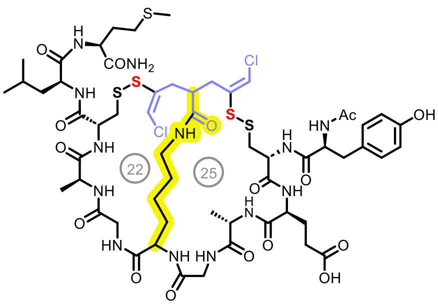

5ac

HPLC analysis demonstrated that this bicyclic peptide was a set of atropisomers in an approximately 1:1 ratio, specifically the “bridge-above” and “bridge-below” configurations, which aligns with observation in Reisberg et al. *Science* **2020**, *367*, 458–463.

V. DFT calculations. [6-21]

All calculations were performed with the Gaussian 16[6] package and ORCA[7] program. Geometry optimizations were performed with B3LYP-D3(BJ)[8] set with the PCM(DCM)[9] solvation model and the def2-SVP[10] basis set was used for all atoms. All optimized structures were verified by frequency calculations and only one imaginary frequency was found in the transition states, while the other structures had no imaginary frequency. Besides, the thermodynamic quantity is corrected by Shermo 2.6[11] (ZPE = 0.98, T = 298.15 K, ilowfreq = 2) after frequency calculations and Intrinsic Reaction Coordinate (IRC)[12] were utilized to confirm the reaction pathway. In addition, the single point calculations considering solvation effect were performed with PWPB95-D3(BJ)[13] set with the SMD(DCM)[14] solvation model and the ma-TZVPP[15] basis for all atoms. ESP[16]/LEAE[17]/MO analysis/Dual descriptor[18] was calculated with B3LYP-D3(BJ)/def2-SVP/PCM(DCM) by Multiwfn[19] and visualized by VMD[20] program. All optimized structures were visualized using CYLview20[21].

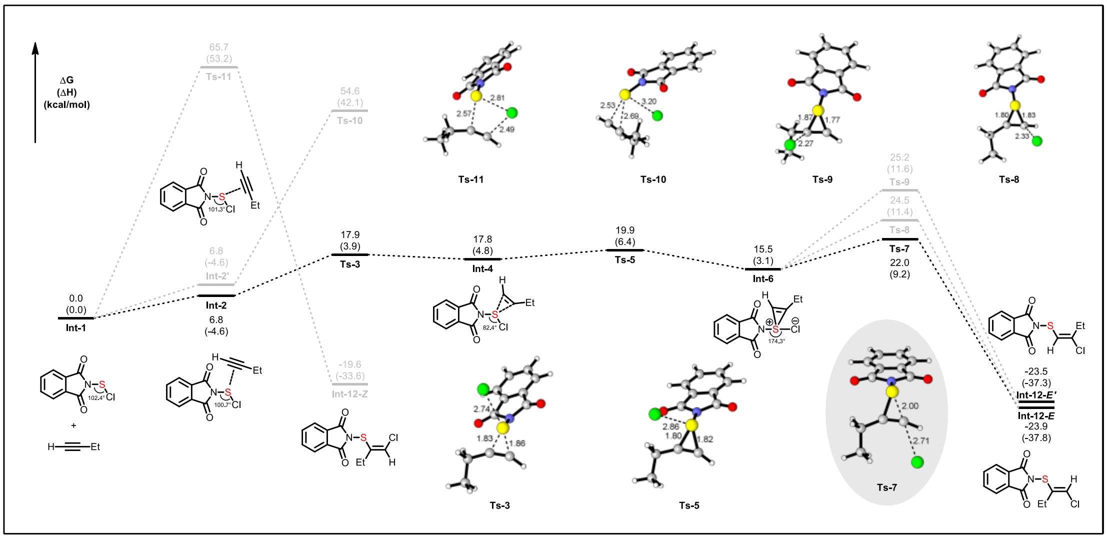

Figure S1. Regional selectivity and Z/E selectivity. Calculated energy data for structures at the level of PWPB95-D3(BJ)/ma-TZVPP/SMD(DCM)//B3LYP-D3(BJ)/def2-SVP/PCM(DCM).

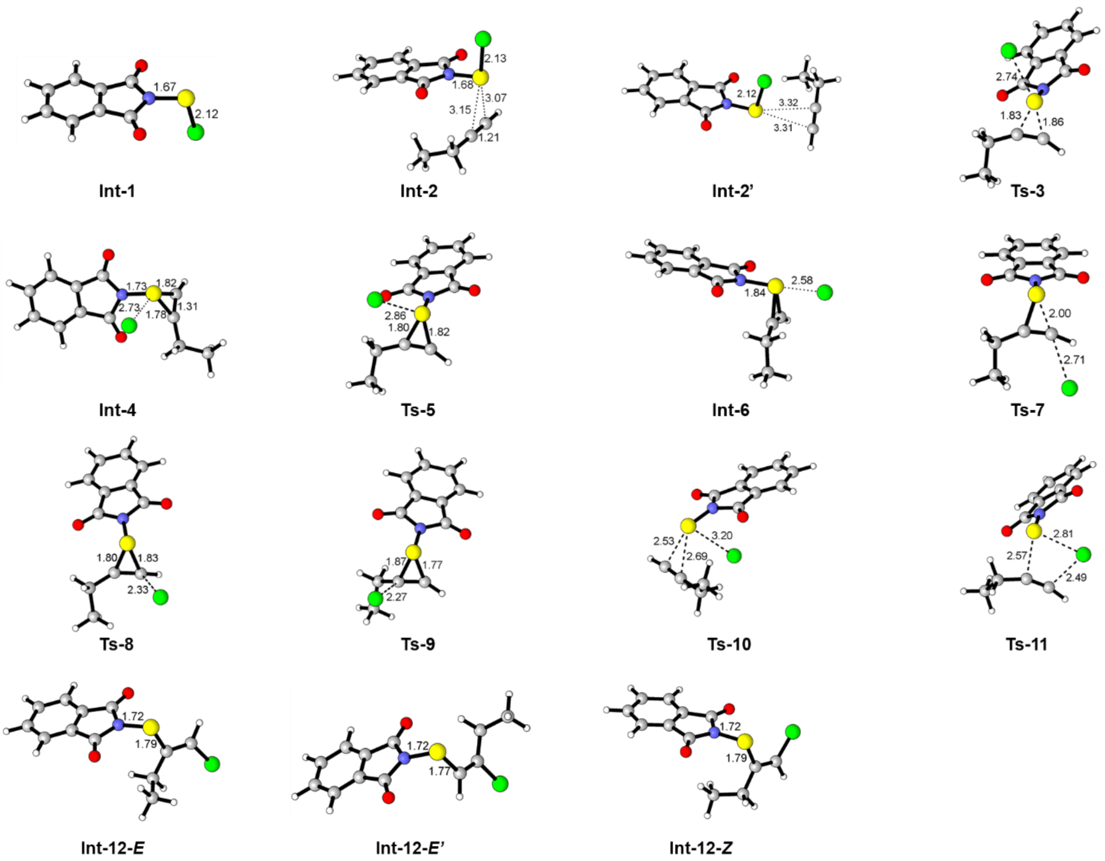

Figure S2. Structures of all intermediates and transition states. Geometry optimizations were performed at the level of B3LYP-D3(BJ)/def2-SVP/PCM(DCM).

Table S1. Energy data of regional selectivity and Z/E selectivity. Calculated energy data for structures at the level of PWPB95-D3(BJ)/ma-TZVPP/SMD(DCM)//B3LYP-D3(BJ)/def2-SVP/PCM(DCM).

	**G (Hartree)**	**H (Hartree)**	**ΔG (kcal/mol)**	**ΔH (kcal/mol)**
**Int-1**	-1370.603211	-1370.554625	0.0	0.0
**Int-2**	-1526.437024	-1526.373949	6.8	-4.6
**Int-2'**	-1526.434122	-1526.370619	8.6	-2.5
**Ts-3**	-1526.419424	-1526.360331	17.9	3.9
**Int-4**	-1526.419545	-1526.358960	17.8	4.8
**Ts-5**	-1526.416229	-1526.356376	19.9	6.4
**Int-6**	-1526.423200	-1526.361626	15.5	3.1
**Ts-7**	-1526.412879	-1526.351858	22.0	9.2
**Ts-8**	-1526.408781	-1526.348335	24.5	11.4
**Ts-9**	-1526.407752	-1526.348153	25.2	11.6
**Ts-10**	-1526.360840	-1526.299440	54.6	42.1
**Ts-11**	-1526.343209	-1526.281858	65.7	53.2
**Int-12-*E***	-1526.485985	-1526.426801	-23.9	-37.8
**Int-12-*E'***	-1526.485399	-1526.426050	-23.5	-37.3
**Int-12-*Z***	-1526.479150	-1526.420174	-19.6	-33.6
**Ethylethyne**	-155.844690	-155.811942	/	/

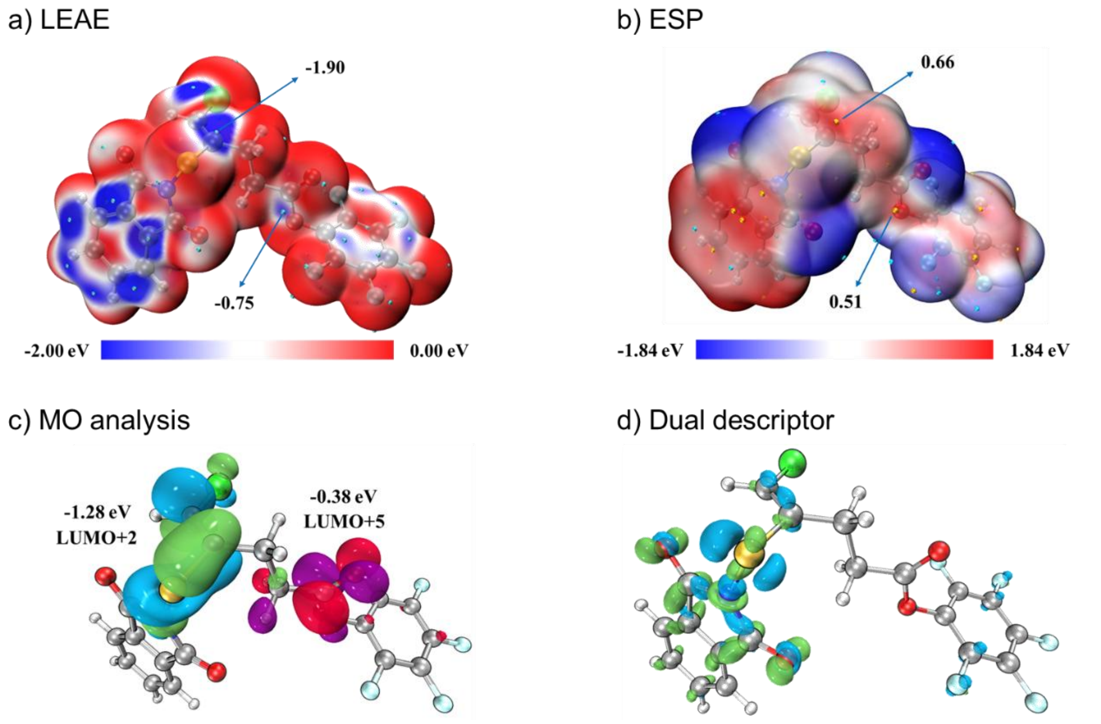

Figure S3. Prediction of nucleophilic reaction sites. Wavefuction was calculated with B3LYP-D3(BJ)/def2-SVP/PCM(DCM) by Multiwfn and visualized by VMD program. a) LEAE (Local Electron Attachment Energy). b) ESP (Electrostatic Potential). c) MO (Molecular Orbital) analysis. d) Dual descriptor.

To elucidate the origin of site-selectivity in unsymmetrical stapling, we conducted density functional theory (DFT) calculations. Initially, local electron attachment energy (LEAE) analysis, molecular electrostatic potential (ESP) mapping, and dual chemical descriptors were employed to identify preferential nucleophilic attack sites (Figures S3). Computational results demonstrated that phthalyl sulfide exhibits significantly higher susceptibility to nucleophilic attack compared to pentafluorophenyl ester. This reactivity trend was further corroborated by HOMO-LUMO energy gap analysis, with phthalyl sulfide showing a reduced energy gap versus its pentafluorophenyl counterpart. Importantly, these theoretical predictions showed excellent agreement with our experimental observations of regioselective bond formation.

Cartesian coordinates for all optimized structures

Int-1

C	-3.60585200	0.47175600	-0.12045200
C	-2.22807500	0.58536800	0.03136000
C	-1.60576500	1.83602400	0.14300000
C	-2.33897500	3.01720700	0.10622400
C	-3.72801900	2.91058100	-0.04696700
C	-4.35142900	1.65793200	-0.15818400
H	-4.08314300	-0.50579100	-0.20688300
H	-1.84652600	3.98719000	0.19291000
H	-4.33725500	3.81606300	-0.08095400
H	-5.43577700	1.60887100	-0.27655800
C	-0.14172500	1.64141300	0.29248500
C	-1.19739700	-0.48075400	0.10188300
O	0.74224000	2.44941700	0.38887100
O	-1.30860600	-1.67412500	0.01770900
N	0.04585100	0.21547000	0.29684600
S	1.53666700	-0.53363200	0.37624300
Cl	2.10351600	-0.63226100	-1.66239000

Int-12-*E′*

C	-0.31537300	-5.56937600	-3.61924800
C	0.05181200	-5.05674600	-2.38116400
C	-0.90575100	-4.70803000	-1.42111900
C	-2.26554800	-4.85984300	-1.66346100
C	-2.64548100	-5.37795600	-2.91064200
C	-1.68628100	-5.72694300	-3.87260500
H	0.43717300	-5.83768600	-4.36283100
H	-3.00577700	-4.58526700	-0.90986900
H	-3.70508800	-5.51198400	-3.13818000
H	-2.01385100	-6.12744200	-4.83430400
C	-0.20232800	-4.18994700	-0.21334800
C	1.40781700	-4.77379000	-1.82859200
O	-0.66545200	-3.77103300	0.81982400
O	2.48899400	-4.91334500	-2.34406700
N	1.17827900	-4.27493200	-0.52067800
S	2.42440200	-3.79277200	0.56639900
Cl	2.37377300	0.55522600	0.67607100
C	2.53349800	-2.05905800	0.20893000
H	3.04684600	-1.76683400	-0.71038800
C	2.06869400	-1.13391200	1.06712400
C	1.33746500	-1.34340100	2.35794400
H	0.93668100	-2.36624600	2.35487300
H	0.47798000	-0.65334900	2.36999400
C	2.21424500	-1.10126200	3.59259000
H	2.63178800	-0.08289000	3.58900000
H	1.62183500	-1.22621300	4.51128200
H	3.05177600	-1.81532800	3.62250200

Int-12-*E* (in DCM)

C	-2.96168900	0.64662000	-1.32908100
C	-1.77648900	0.74455200	-0.61032600
C	-1.71244900	1.44187500	0.60199300
C	-2.83059700	2.06716100	1.14017300
C	-4.03137500	1.97410800	0.42081400
C	-4.09577200	1.27541900	-0.79371200
H	-3.00408700	0.10050800	-2.27310600
H	-2.77199600	2.60804800	2.08631800
H	-4.93189400	2.45262700	0.81142900

H	-5.04549400	1.22077600	-1.32992100
C	-0.32136400	1.35817700	1.13015800
C	-0.42722500	0.18895400	-0.91352500
O	0.13708300	1.81342400	2.14904800
O	-0.06231100	-0.47445000	-1.85127100
N	0.39564600	0.59847000	0.17007900
S	2.07037200	0.23872300	0.28443700
Cl	3.11632800	-3.90806900	0.88526400
C	2.78894700	-2.29991000	0.30182100
H	3.28525900	-2.07132500	-0.64263000
C	2.02196900	-1.41611200	0.95991200
C	1.26529800	-1.65685600	2.23511200
H	1.21601600	-2.74262600	2.40255800
H	0.22574400	-1.31504700	2.09471200
C	1.87747900	-0.95459300	3.45219500
H	1.88567200	0.13557200	3.31006300
H	1.29042900	-1.17740500	4.35630900
H	2.91093300	-1.29549900	3.62019800

Int-12-*Z*

C	-2.02731700	2.06149300	-1.44647900
C	-1.78672800	1.14938600	-0.42637800
C	-2.48032300	1.20940500	0.78755600
C	-3.44034700	2.18476000	1.02750700
C	-3.69043000	3.11040600	0.00322500
C	-2.99551300	3.04983800	-1.21366500
H	-1.48146100	2.00753000	-2.39005500
H	-3.97703700	2.22509600	1.97704100
H	-4.43849000	3.89158500	0.15404600
H	-3.21311200	3.78485400	-1.99142300
C	-2.00337000	0.10188000	1.66407200
C	-0.82752000	0.01000200	-0.37273200
O	-2.34926300	-0.19323000	2.78003800
O	-0.04620500	-0.36751400	-1.21206800
N	-1.00399200	-0.56744000	0.90826000
S	-0.19088400	-1.98754700	1.44738900
Cl	-0.59761000	-2.54425100	4.63497200
C	0.72455600	-1.64121200	3.98054900
H	1.44297200	-1.33227500	4.74424700
C	0.92297300	-1.33342300	2.68692100
C	2.15412500	-0.56569900	2.26332100
H	2.74759300	-1.21005900	1.59182100
H	2.76823100	-0.39759300	3.16294600
C	1.89255500	0.77622500	1.57006500
H	2.83885400	1.32663100	1.45486000
H	1.47378900	0.63668400	0.56514900
H	1.20300700	1.40412300	2.15577000

Int-2'

C	-3.49561300	0.42912000	0.56413900
C	-2.11333700	0.54580900	0.46584200
C	-1.48543600	1.79843200	0.43586300
C	-2.21789600	2.97860900	0.50282800
C	-3.61175400	2.86902100	0.60161800
C	-4.24047000	1.61450000	0.63188700
H	-3.97704300	-0.54995600	0.58641700
H	-1.72168700	3.95023800	0.47823600
H	-4.22073000	3.77372900	0.65540800
H	-5.32844900	1.56316600	0.70875300
C	-0.01700500	1.60504500	0.32454300

C	-1.08081100	-0.51695600	0.37386100
O	0.86733600	2.41482700	0.24383400
O	-1.20286100	-1.71294100	0.33547700
N	0.17222800	0.18121500	0.32670200
S	1.65219100	-0.58433100	0.15788200
Cl	1.80352500	-0.73308800	-1.95422200
C	4.45481000	-2.32583600	0.41655600
H	5.05453900	-1.64357800	0.99036200
C	3.78352800	-3.09868400	-0.23508500
C	2.95880400	-4.02448800	-1.01234000
H	3.54188100	-4.94315000	-1.19862700
H	2.76918000	-3.57254800	-2.00054400
C	1.62268900	-4.37761200	-0.34074400
H	0.99499900	-3.48458800	-0.20906600
H	1.07086000	-5.10005600	-0.96123300
H	1.78718200	-4.83051000	0.64913900

Int-2

C	-3.52349300	0.59729700	0.07182900
C	-2.13341100	0.59818700	0.08385800
C	-1.40702500	1.72017000	0.50475100
C	-2.04447100	2.88091800	0.92863200
C	-3.44617800	2.88796500	0.91838200
C	-4.17381100	1.76452100	0.49652700
H	-4.08210200	-0.28106500	-0.25570200
H	-1.47099300	3.74926400	1.25722100
H	-3.98272800	3.78136800	1.24421900
H	-5.26506400	1.80174100	0.50072300
C	0.04412100	1.41648100	0.42206500
C	-1.18414800	-0.47990100	-0.29609600

O	0.99421600	2.10130300	0.69994700
O	-1.39680000	-1.59090100	-0.70354400
N	0.11657700	0.07186600	-0.06018600
S	1.54726800	-0.77001900	-0.29072900
Cl	2.05033800	-0.15895800	-2.27002500
C	0.63229100	-2.25189200	2.23306500
H	0.30301400	-3.15530800	1.75364000
C	1.00089000	-1.22880800	2.77370200
C	1.40457700	0.02038700	3.42004900
H	1.93694300	0.64156000	2.68094800
H	2.13592500	-0.20998400	4.21361500
C	0.22324800	0.80683700	4.00753300
H	-0.29966500	0.21934300	4.77713700
H	0.58129600	1.74023100	4.46667000
H	-0.50794600	1.06887700	3.22842500

Int-4

C	-3.15091100	0.46599200	-1.98195100
C	-2.11913600	0.24831300	-1.07681800
C	-1.65211100	1.26906600	-0.23987600
C	-2.20178000	2.54495500	-0.27405800
C	-3.24059200	2.77460500	-1.18750100
C	-3.70743700	1.75229000	-2.02744000
H	-3.50821100	-0.33416500	-2.63222500
H	-1.83281900	3.33490300	0.38212900
H	-3.69406700	3.76600800	-1.24865100
H	-4.51689900	1.96409000	-2.72896000
C	-0.55738200	0.73514600	0.61203200
C	-1.33834900	-0.98452600	-0.80434000
O	0.10959000	1.29363400	1.44446100

O	-1.36937200	-2.06598800	-1.33596400
N	-0.47625100	-0.64933500	0.27752700
S	0.91545300	-1.64956800	0.50399800
Cl	1.98288400	0.04283700	-1.35208200
C	0.88690600	-2.03745700	2.27727000
H	0.42597200	-2.68759600	3.01883500
C	1.72726800	-1.08179800	1.97977800
C	2.77598400	-0.08596700	2.25940600
H	2.25190300	0.85862500	2.47730000
H	3.31008500	0.08281300	1.31035700
C	3.70003500	-0.50437000	3.40509000
H	4.23556500	-1.43543600	3.16672900
H	4.44494800	0.28438400	3.58366200
H	3.13636000	-0.65836200	4.33779300

Int-6

C	-3.95357900	0.71668100	-0.61805900
C	-2.60440300	0.72183700	-0.28875100
C	-1.94114600	1.90540100	0.06146700
C	-2.60296200	3.12593700	0.09519800
C	-3.96612700	3.13065800	-0.23954200
C	-4.62993800	1.94642700	-0.59008200
H	-4.46443900	-0.20926100	-0.88821800
H	-2.08004100	4.04355200	0.37063700
H	-4.52042200	4.07145000	-0.22707700
H	-5.69113700	1.98307800	-0.84511100
C	-0.51768600	1.57243000	0.37021700
C	-1.62273900	-0.40289300	-0.21950100
O	0.38082800	2.29930600	0.73309900
O	-1.78081300	-1.58296000	-0.43410300
N	-0.40933800	0.18564900	0.17881100
S	1.18383600	-0.72808800	0.19625400
Cl	3.52272500	-1.82152200	0.07983700
C	0.85953300	-1.65306300	1.72896500
H	0.40526700	-2.61967400	1.92030900
C	1.50222100	-0.57927200	2.01097800
C	2.28689000	0.38385300	2.78777100
H	1.87544500	1.38754200	2.59613300
H	3.29382200	0.36058000	2.33556500
C	2.32664800	0.03877800	4.27837000
H	2.76211900	-0.95787600	4.44256000
H	2.94680700	0.77686200	4.80667900
H	1.31894100	0.05704300	4.71917400

Ts-10

C	-3.42318100	0.90974800	0.77612100
C	-2.18548300	0.41667400	0.37552000
C	-1.11976600	1.27719000	0.07052200
C	-1.25537800	2.65810900	0.16277300
C	-2.49990300	3.16073500	0.56351100
C	-3.56780300	2.29984400	0.86543700
H	-4.24736400	0.23350400	1.00820300
H	-0.42166400	3.32038100	-0.07517700
H	-2.64462500	4.24006100	0.64192900
H	-4.52557200	2.72343700	1.17399000
C	0.06609800	0.48815700	-0.31784600
C	-1.76643400	-0.98639800	0.19131700
O	1.17880600	0.82051700	-0.60709600
O	-2.36977200	-2.01400500	0.31122100
N	-0.35315300	-0.91483900	-0.17930700

S	0.52750200	-2.16685800	-0.70431900
Cl	-1.00582700	-0.49831000	-2.96851200
C	0.43884800	-3.83895400	-2.60174600
H	-0.14721700	-4.64106600	-2.18790700
C	1.13419400	-3.01176400	-3.18432900
C	1.83257400	-1.97281100	-3.90947600
H	1.08109300	-1.15780100	-4.00347500
H	2.01919300	-2.34579900	-4.93279000
C	3.11942400	-1.46412200	-3.25197900
H	3.83601600	-2.28065100	-3.07604700
H	3.59650600	-0.71926900	-3.90500900
H	2.88887300	-0.98467600	-2.28962400

Ts-11

C	-3.46578000	0.39069800	0.19414000
C	-2.08649900	0.46556300	0.35948500
C	-1.42360200	1.69949300	0.43085100
C	-2.11816700	2.90090100	0.34114000
C	-3.50782500	2.83300200	0.17332200
C	-4.17124500	1.59759200	0.10117600
H	-3.97320100	-0.57365400	0.13741100
H	-1.59560100	3.85716800	0.39713200
H	-4.08648600	3.75570700	0.09646000
H	-5.25491900	1.57937700	-0.03055900
C	0.02856900	1.46608800	0.61074000
C	-1.09649000	-0.62893500	0.47710000
O	0.93805500	2.24142700	0.71230800
O	-1.24415800	-1.82060600	0.43189600
N	0.17952100	0.02713900	0.65272600
S	1.62524600	-0.75158900	0.63597400
Cl	2.15476300	0.55537400	-1.79466900
C	1.67352700	-1.84064600	-2.28722100
H	1.76172200	-1.54029700	-3.31634900
C	1.52445900	-2.49698200	-1.24131700
C	1.29800200	-3.70192100	-0.43120300
H	1.09300100	-3.39310900	0.60787600
H	2.23966700	-4.27823400	-0.41054200
C	0.14585800	-4.57144400	-0.95320900
H	0.33775000	-4.91502100	-1.98081200
H	0.02480500	-5.45657400	-0.31000300
H	-0.79476400	-4.00318800	-0.94083200

Ts-3

C	-3.30700400	0.38876300	-1.66362300
C	-2.11881700	0.24789800	-0.95664400
C	-1.49800500	1.34321300	-0.34370300
C	-2.04520500	2.61892000	-0.41054100
C	-3.24065000	2.77114600	-1.12717600
C	-3.86136400	1.67412400	-1.74366700
H	-3.78392800	-0.46925400	-2.14028000
H	-1.55593200	3.46705700	0.07118700
H	-3.69675900	3.75981800	-1.20958400
H	-4.79070600	1.82611400	-2.29637600
C	-0.26139800	0.88144900	0.33650900
C	-1.29918500	-0.96440900	-0.71104200
O	0.54583600	1.50597500	0.97557300
O	-1.42855600	-2.09340200	-1.11216900
N	-0.24706000	-0.53537800	0.14941800
S	1.20226600	-1.46653300	0.13834400
Cl	1.60029500	0.10256100	-2.07383800

C	1.15697000	-2.17820000	1.85211700
H	0.68697000	-3.00386800	2.37909600
C	1.93570300	-1.15118800	1.78838500
C	2.89466300	-0.16075200	2.30306400
H	2.42298100	0.82699000	2.18428400
H	3.77083900	-0.16491900	1.63339900
C	3.28627400	-0.44704500	3.75476800
H	3.76236200	-1.43390500	3.85428500
H	3.99965500	0.31541500	4.09872800
H	2.40739500	-0.41749500	4.41598400

Ts-5

C	-4.03306700	0.76198000	-0.83445600
C	-2.72078000	0.69236700	-0.38307000
C	-2.03261100	1.83610500	0.04541400
C	-2.63198000	3.08951000	0.03863700
C	-3.95617600	3.16837200	-0.41776000
C	-4.64470200	2.02443200	-0.84682200
H	-4.56329600	-0.13228700	-1.16602200
H	-2.08993200	3.97495500	0.37469400
H	-4.46060300	4.13645500	-0.43981800
H	-5.67469300	2.11949000	-1.19638400
C	-0.66326600	1.44616300	0.47825000
C	-1.82337100	-0.48630900	-0.25039400
O	0.23067400	2.11665100	0.92764800
O	-2.00880600	-1.64994100	-0.50138700
N	-0.60995400	0.03766600	0.27825300
S	0.81707600	-0.93989200	0.44626100
Cl	3.35258800	0.00885500	-0.48133800
C	0.63105900	-1.69300000	2.08840200

H	0.19310800	-2.58476600	2.53148900
C	1.36531300	-0.62245300	2.13448100
C	2.27395300	0.36392100	2.72718200
H	1.72933100	1.32161700	2.76938000
H	3.06693800	0.50323200	1.96693300
C	2.80433400	-0.07199900	4.09360300
H	3.37316400	-1.01062900	4.01597100
H	3.47647900	0.70323800	4.48865100
H	1.98904100	-0.21918000	4.81833000

Ts-7

C	-3.93673600	0.73734900	-0.52419300
C	-2.57029400	0.74399000	-0.27234700
C	-1.88253200	1.93487500	0.00354200
C	-2.53763200	3.16006600	0.03840300
C	-3.91679800	3.16133000	-0.21681800
C	-4.60435100	1.97064500	-0.49351900
H	-4.46561000	-0.19331000	-0.73585400
H	-1.99658300	4.08220000	0.25665800
H	-4.46603600	4.10473800	-0.19900100
H	-5.67832900	2.00537400	-0.68684300
C	-0.44970400	1.62507000	0.24052800
C	-1.61323100	-0.39221900	-0.23062500
O	0.47127200	2.34199500	0.53867500
O	-1.78291100	-1.57192400	-0.38936800
N	-0.35012600	0.21223500	0.06343400
S	1.10309800	-0.66527500	0.18593000
Cl	0.75060100	-3.14395600	4.18082100
C	0.90602200	-1.73498000	1.86923000
H	0.45815400	-2.71024500	2.08962600

C	1.49496200	-0.59902200	1.97696700
C	2.18916500	0.43976300	2.76642800
H	1.66428400	1.39208800	2.58834100
H	3.19328700	0.56602500	2.32641700
C	2.26230800	0.08813900	4.25262500
H	2.76824800	-0.87383800	4.40763800
H	2.80710000	0.88014500	4.78674100
H	1.25660100	-0.01093100	4.68306300

Ts-8

C	-3.98307200	0.73081100	-0.83837800
C	-2.66278100	0.73816100	-0.40595100
C	-2.06322200	1.90203000	0.09514400
C	-2.76243600	3.09969200	0.18054500
C	-4.09615300	3.10062200	-0.25455300
C	-4.69608600	1.93618900	-0.75547600
H	-4.44365700	-0.17935500	-1.22614100
H	-2.28907700	4.00162400	0.57213900
H	-4.67835700	4.02285000	-0.20249200
H	-5.73619400	1.96959400	-1.08597700
C	-0.65759400	1.59547500	0.47789900
C	-1.67097200	-0.37128500	-0.36456500
O	0.19321100	2.30430400	0.95339800
O	-1.77823300	-1.52986700	-0.67697100
N	-0.48976300	0.21838200	0.16297000
S	0.97186700	-0.67431000	0.43260900
Cl	2.48038500	-3.27559100	1.76489600
C	0.88705500	-1.59440800	2.01451900
H	0.11618900	-2.30430600	2.30256100
C	1.34983800	-0.36001100	2.16064900

C	2.38039500	0.48320600	2.81196600
H	2.03919000	1.52938400	2.84089400
H	3.29069800	0.46161000	2.18737100
C	2.69143100	-0.06173500	4.20945900
H	3.02580700	-1.10777200	4.14031000
H	3.48870900	0.53349800	4.67812300
H	1.80490100	-0.02301300	4.86070500

Ts-9

C	-3.57187600	0.65463100	-1.04402100
C	-2.18806800	0.66196900	-0.92012900
C	-1.46893800	1.86073300	-0.81409900
C	-2.10851700	3.09410900	-0.82587700
C	-3.50586900	3.09520800	-0.95127100
C	-4.22519200	1.89612600	-1.05833900
H	-4.12494700	-0.28246900	-1.12694100
H	-1.54225800	4.02315500	-0.74147200
H	-4.04385200	4.04523300	-0.96594100
H	-5.31227400	1.93098100	-1.15445600
C	-0.01937800	1.54351000	-0.69011100
C	-1.23185000	-0.47937800	-0.87944800
O	0.93132000	2.27187200	-0.55176400
O	-1.42769200	-1.66637400	-0.94294300
N	0.04752000	0.12633700	-0.74619400
S	1.53750200	-0.76091900	-0.66680300
Cl	4.36599300	-1.08979800	0.66878400
C	1.34466000	-1.78671900	0.76680000
H	1.19657900	-2.86140300	0.80005400
C	2.15683800	-0.77308700	1.09299400
C	2.21626800	0.37796900	2.04076500

H	1.20049300	0.80722500	2.07814900
H	2.89719100	1.13669000	1.63700300
C	2.65435500	-0.07710500	3.43524600
H	3.66453900	-0.50605700	3.38762400
H	2.66322700	0.78420300	4.11930800
H	1.96741200	-0.83482400	3.84256300

VI. The solubility of stapling reagents

All stapling reagents exhibited excellent solubility in acetonitrile and DMF with concentrations ranging from 34 to 230 mM.

Stapling reagents / Solubility / Solvent	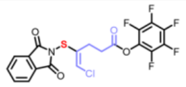 3a	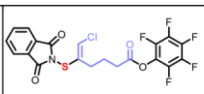 3b	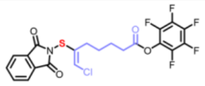 3c	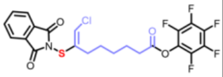 3d
**MeCN**	**5 mg/100 ul (105 mM)**	**5 mg/150 ul (68 mM)**	**5 mg/150 ul (66 mM)**	**5 mg/100 ul (96 mM)**
**DMF**	**5 mg/50 ul (210 mM)**	**5 mg/60 ul (170 mM)**	**5 mg/70 ul (141 mM)**	**5 mg/50 ul (193 mM)**
**H_2O**	**insoluble**			

Stapling reagents / Solubility / Solvent	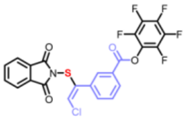 3j	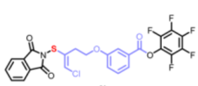 3k	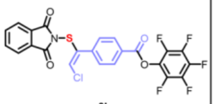 3l	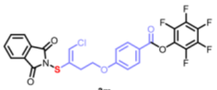 3m
**MeCN**	**5 mg/150 ul (63 mM)**	**5 mg/150 ul (59 mM)**	**5 mg/100 ul (95 mM)**	**5 mg/200 ul (44 mM)**
**DMF**	**5 mg/100 ul (95 mM)**	**5 mg/70 ul (126 mM)**	**5 mg/50 ul (191 mM)**	**5 mg/80 ul (110 mM)**
**H_2O**	**insoluble**			

Stapling reagents / Solubility / Solvent	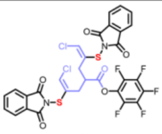 3n	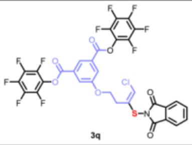 3q
**MeCN**	**5 mg/200 ul (34 mM)**	**5 mg/100 ul (98 mM)**
**DMF**	**5 mg/100 ul (69 mM)**	**5 mg/60 ul (59 mM)**
**H_2O**	**insoluble**	

Figure S4. The solubility of stapling reagents in MeCN、DMF、H_2O.

VII. Control experiments based on chemoselectivity and site selectivity.

1. Trapping of Intermediate Species

Using the nonapeptide Ac-CPIMEDRKP-CONH2 (CP-9) as an example, reagent **3b** was added under DIPEA-free conditions, and the reaction was allowed to proceed for 1 minute.

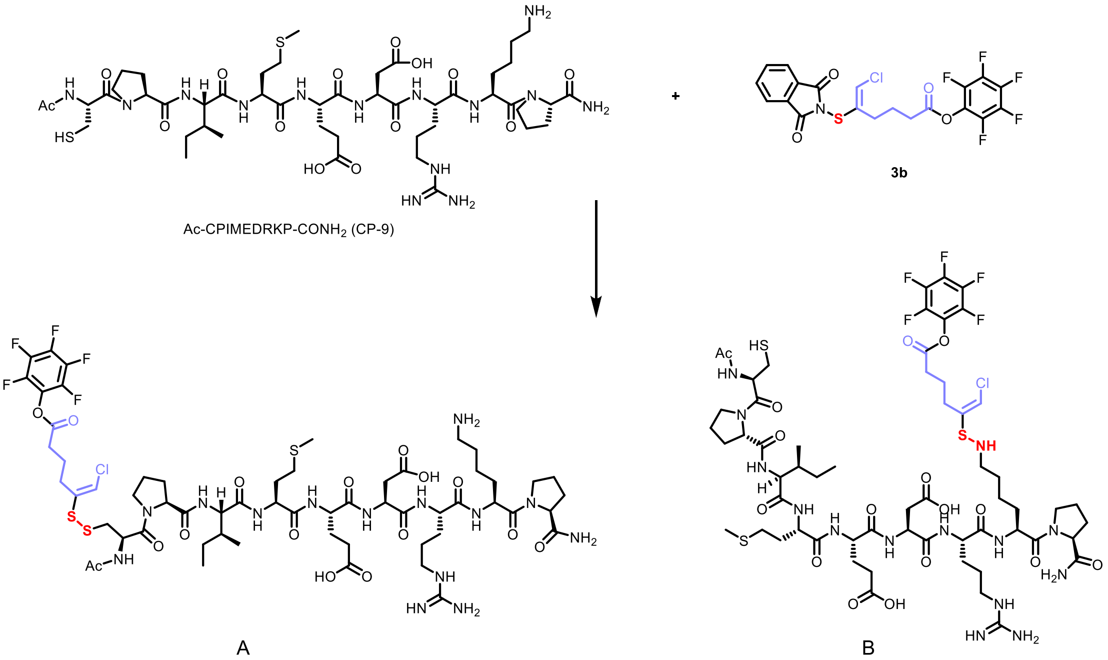

A B

Figure S5: Two plausible structural configurations

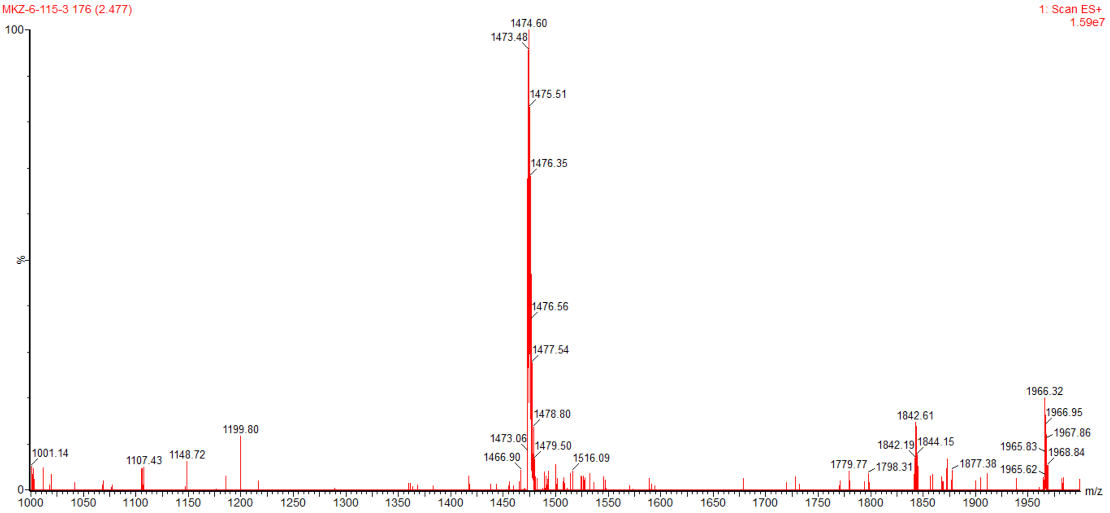

Figure S6: Observed molecular weight 1473.48

Molecular mass analysis indicates that the phthalimido thioester chloride group dissociates first, potentially forming either Structure **A** or **B**. To determine whether the product exists as a single compound (**A** or **B**) or a mixture of both, LC-MS and HPLC analyses were performed.

Distinct differences in polarity and acid-base properties between Structures **A** and **B** would result in two separate peaks in LC-MS and analytical HPLC chromatograms if a mixture were present.

Analytical Data for Structural Characterization：

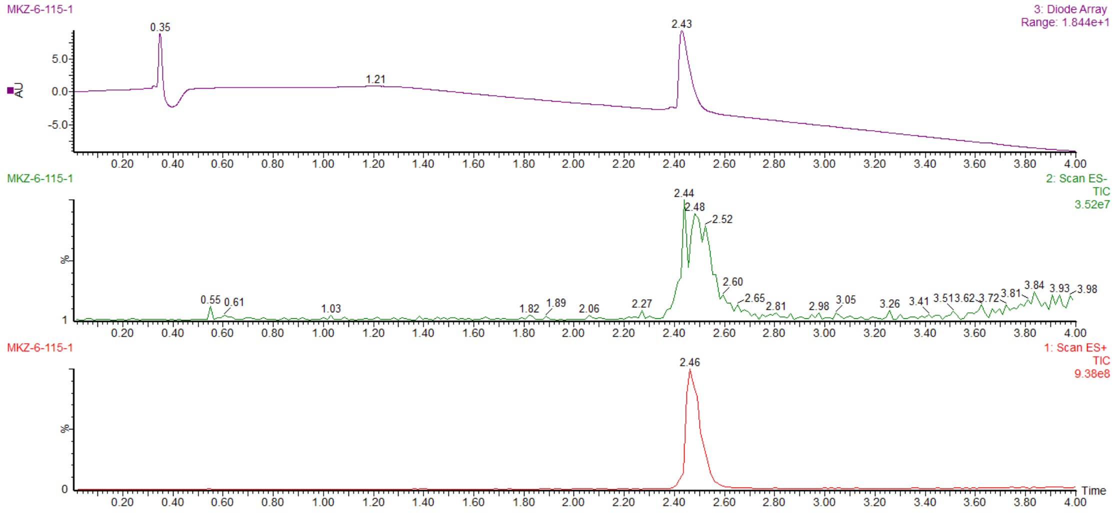

Figure S7: UV-Vis and Mass Spectrometry Molecular weight

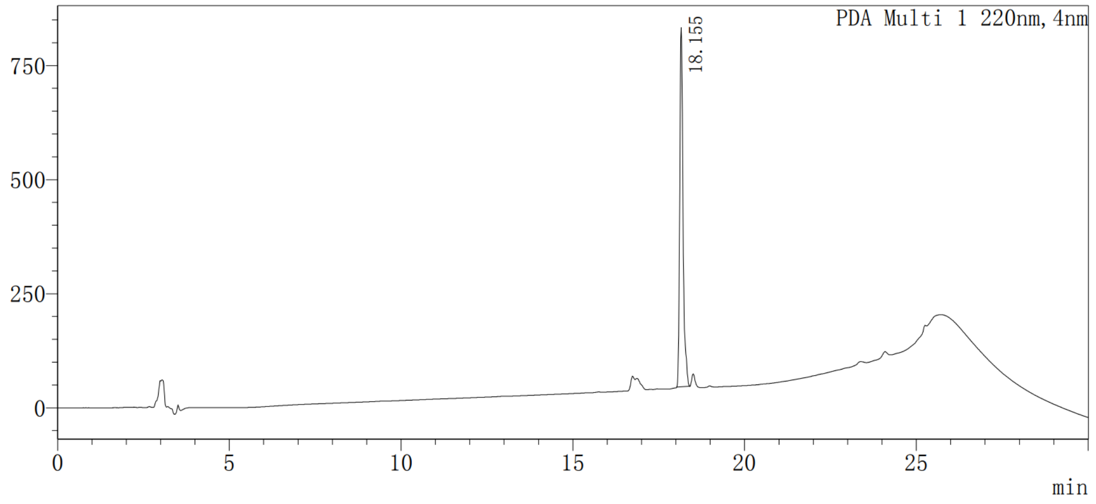

Figure S8: Analytical HPLC Figure

Conclusion: The intermediate trapping experiments successfully determined the molecular weight, with mass spectrometry (MS) and high-performance liquid chromatography (HPLC) analyses unambiguously confirming the homogeneity of the product.

2 Structural Confirmation via Mass/Mass Spectrometry：

a) Mass-Mass spectrometry fragmentation analysis: （Four chlorine-containing fragments detected at m/z： **587**, **700**, **1231** (**1248**), and **1359**.

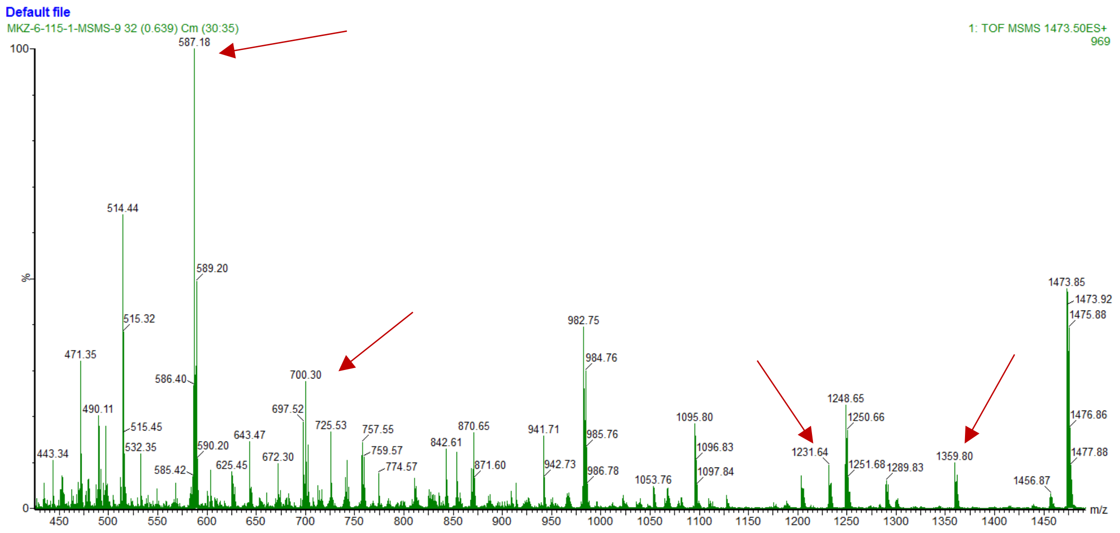

Figure S9 Mass-Mass spectrometry fragmentation analysis

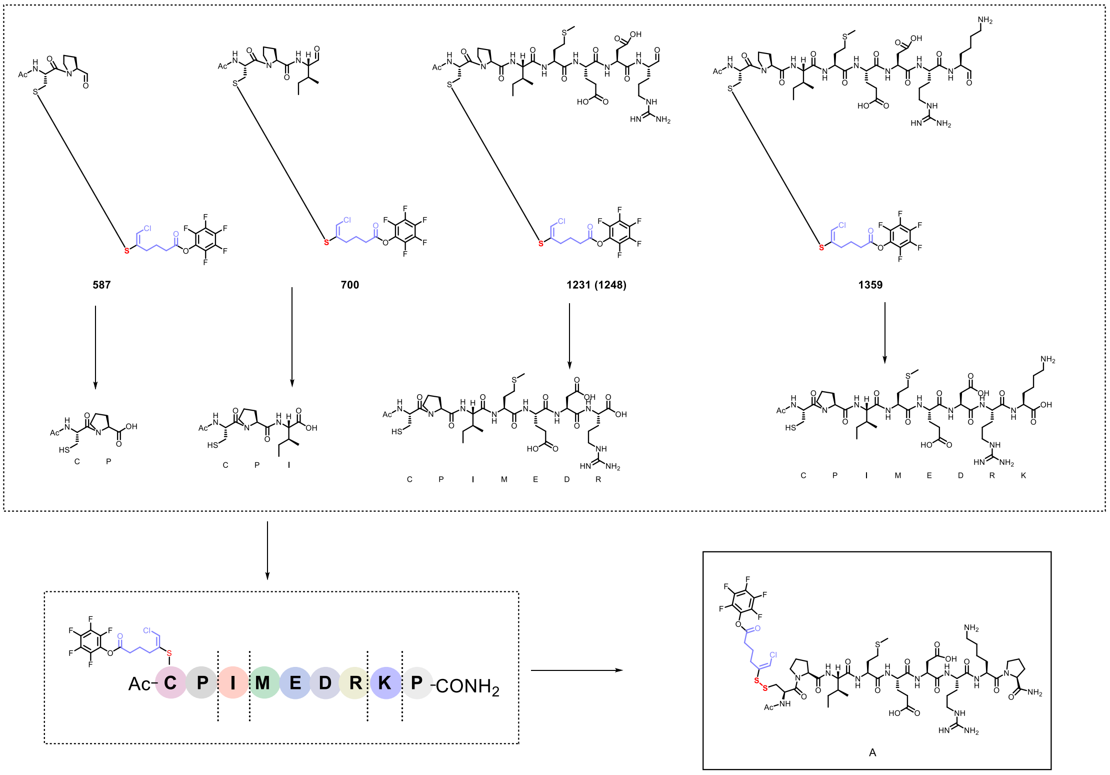

Figure S10: Validated structural configuration

Conclusion: Fragmentation patterns confirm the final structure corresponds to Structure **A**, characterized by a stable disulfide bond formation, rather than an unstable thioester linkage.

Supporting Investigations

a): Reagent **3d** was reacted with (tert-butoxycarbonyl)-D-cysteine at room temperature for 1 minute.

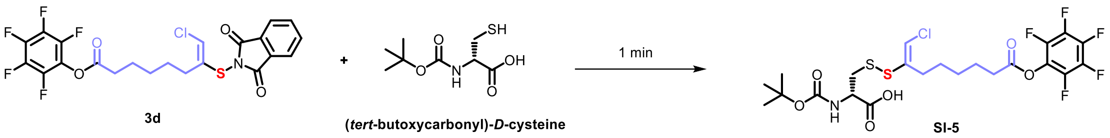

SI-5: ^{1}H NMR (400 MHz, $CDCl_3$) δ 6.45 (s, 1H), 5.35 (d, J = 7.6 Hz, 1H), 4.65 – 4.64 (m, 1H), 3.22 – 3.08 (m, 2H), 2.68 (t, J = 7.4 Hz, 2H), 2.55 (t, J = 6.9 Hz, 2H), 1.84 – 1.77 (m, 2H), 1.65 – 1.57 (m, 2H), 1.46 (s, 9H), 1.38 – 1.22 (m, 2H).; ^{19}F NMR (376 MHz, $CDCl_3$) δ -146.39 – -154.83 (m, 2F), -154.98 – -159.16 (m, 1F), -162.34 (t, J = 19.5 Hz, 2F).; ^{13}C NMR (100 MHz, $CDCl_3$) δ 175.19, 169.48, 155.30, 142.55 – 141.26 (m, C_{Ar}-F), 140.01 – 139.95 (m, C_{Ar}-F), 138.34 – 136.44 (m, C_{Ar}-F), 137.67, 125.11 – 123.51 (m, C_{Ar}-O), 118.96, 80.82, 52.77, 39.76, 33.17, 29.56, 28.29, 28.13, 26.54, 24.47.; HRMS (ESI) Calcd for $C_{22}H_{26}ClF_5NO_6S_2$ $[M+H]^+$ 594.0805, found 594.0810.; IR: 617.22, 686.66, 810.10, 933.55, 1165.00, 1280.73, 1303.88, 1327.03, 1581.63, 1674.21, 1705.07.

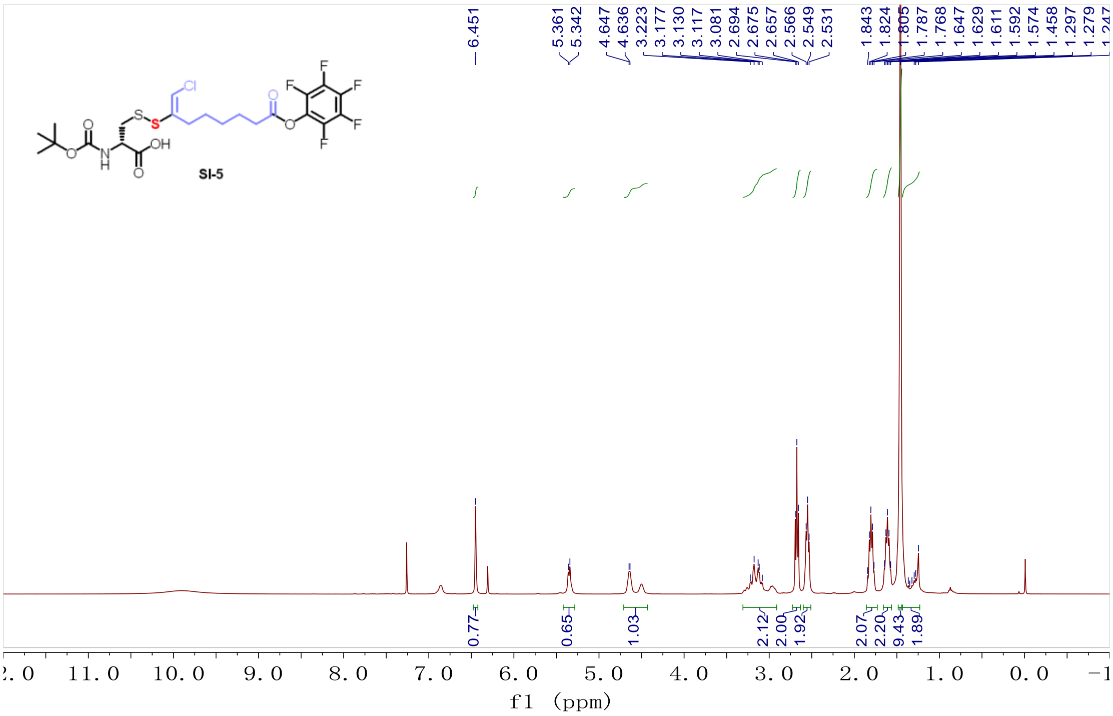

Figure S10: ^{1}H NMR 400 MHz $CDCl_3$ of **SI-5**

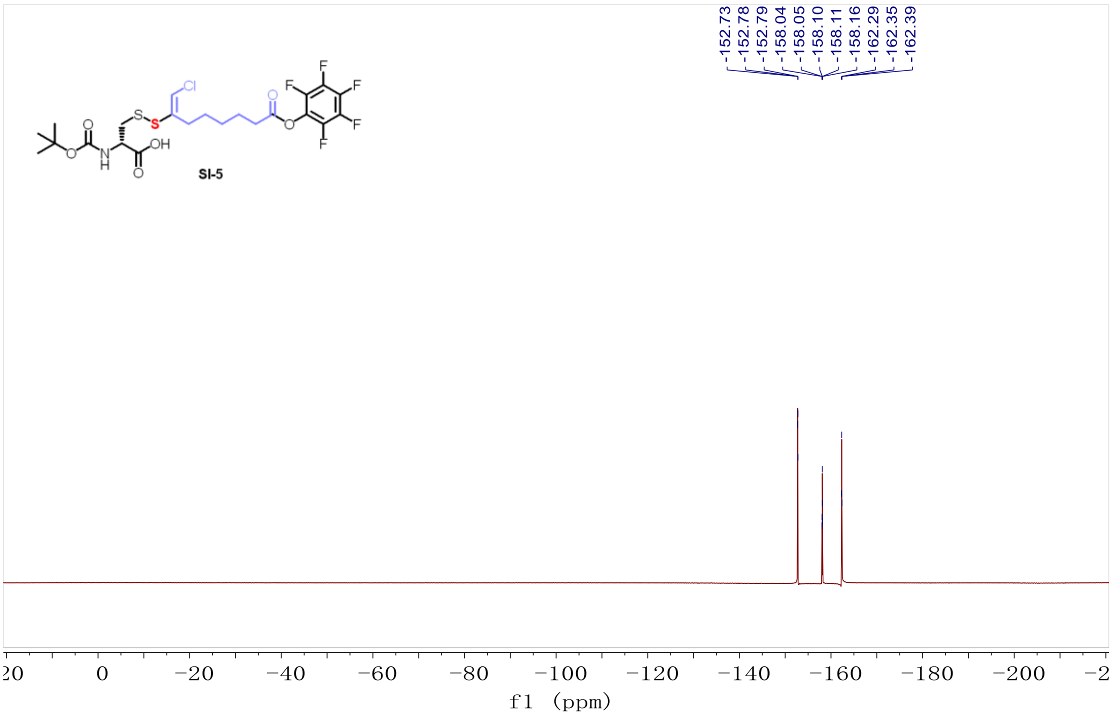

Figure S12: ^{19}F NMR 400 MHz $CDCl_3$ of **SI-5**

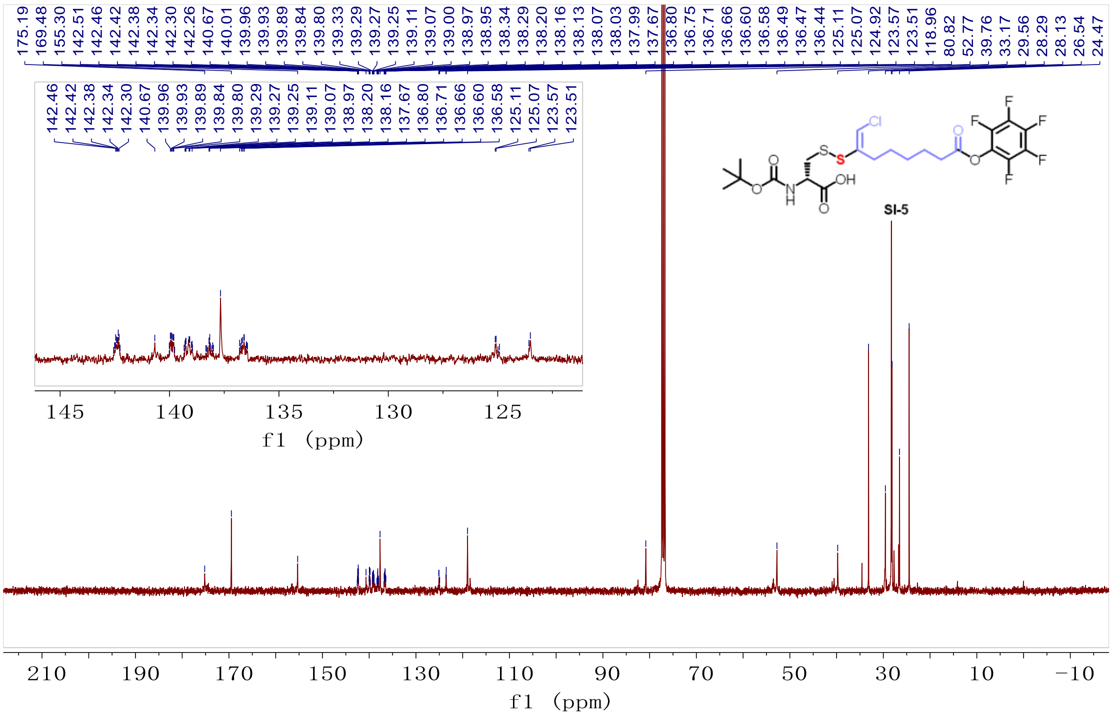

Figure S13: ^{13}C NMR 400 MHz $CDCl_3$ of **SI-5**

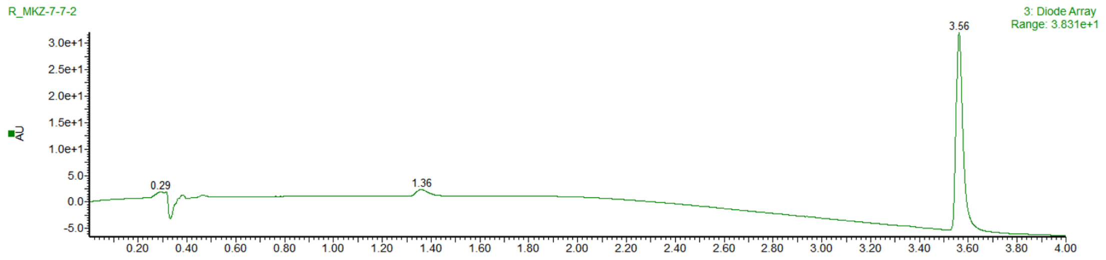

Figure S14: Ultraviolet absorption of **SI-5**

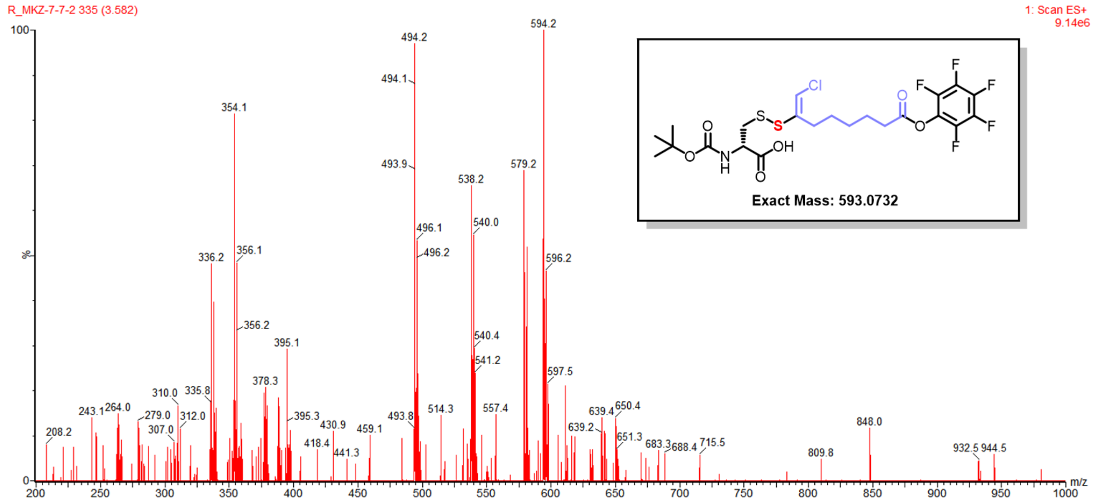

Figure S15: LC-MS Exact Mass analysis of **SI-5**

b): Reagent **3d** and tert-butyl (tert-butoxycarbonyl)-L-lysinate were reacted in the presence of DIPEA at room temperature for 10 minutes:

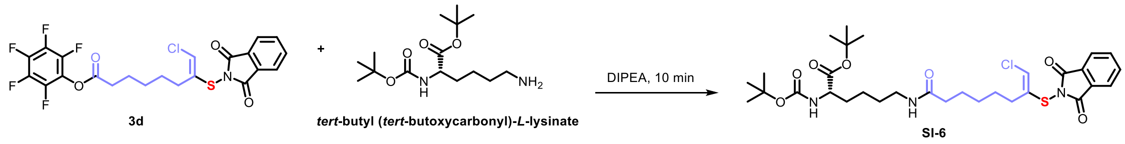

SI-6: ^{1}H NMR (400 MHz, $CDCl_3$) δ 7.91 (dd, *J* = 5.1, 3.1 Hz, 2H), 7.80 (dd, *J* = 5.0, 3.3 Hz, 2H), 6.75 (s, 1H), 5.80 (s, 1H), 5.07 (d, *J* = 8.0 Hz, 1H), 4.18 – 4.08 (m, 1H), 3.26 – 3.22 (m, 1H), 2.30 (t, *J* = 7.5 Hz, 2H), 2.17 (t, *J* = 7.4 Hz, 2H), 1.84 – 1.72 (m, 2H), 1.74 – 1.62 (m, 4H), 1.58 – 1.52 (m, 2H), 1.44 (s, 9H), 1.42 (s, 9H), 1.38 – 1.24 (m, 2H).; ^{13}C NMR (100 MHz, $CDCl_3$) δ 171.92, 167.71, 155.53, 138.48, 134.94, 131.79, 124.15, 123.50, 119.60, 81.88, 79.65, 53.68, 39.21, 36.56, 35.08, 32.78, 30.14, 29.17, 28.35, 28.02, 26.46, 25.24, 22.60.; HRMS (ESI) Calcd for $C_{31}H_{45}ClN_3O_7S$ $[M+H]^+$ 638.2661, found 638.2668.; IR: 717.52, 1049.28, 1157.29, 1273.02, 1365.60, 1651.07, 1712.79, 1743.65, 2931.80.

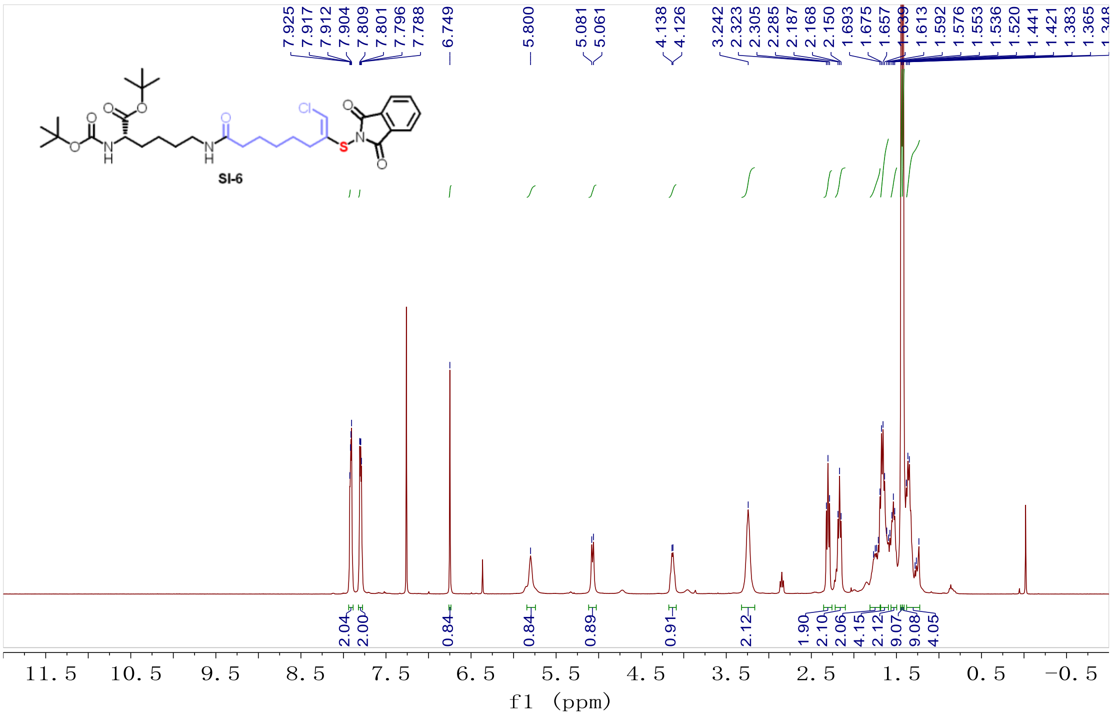

Figure S16: ^{1}H NMR 400 MHz $CDCl_3$ of **SI-6**

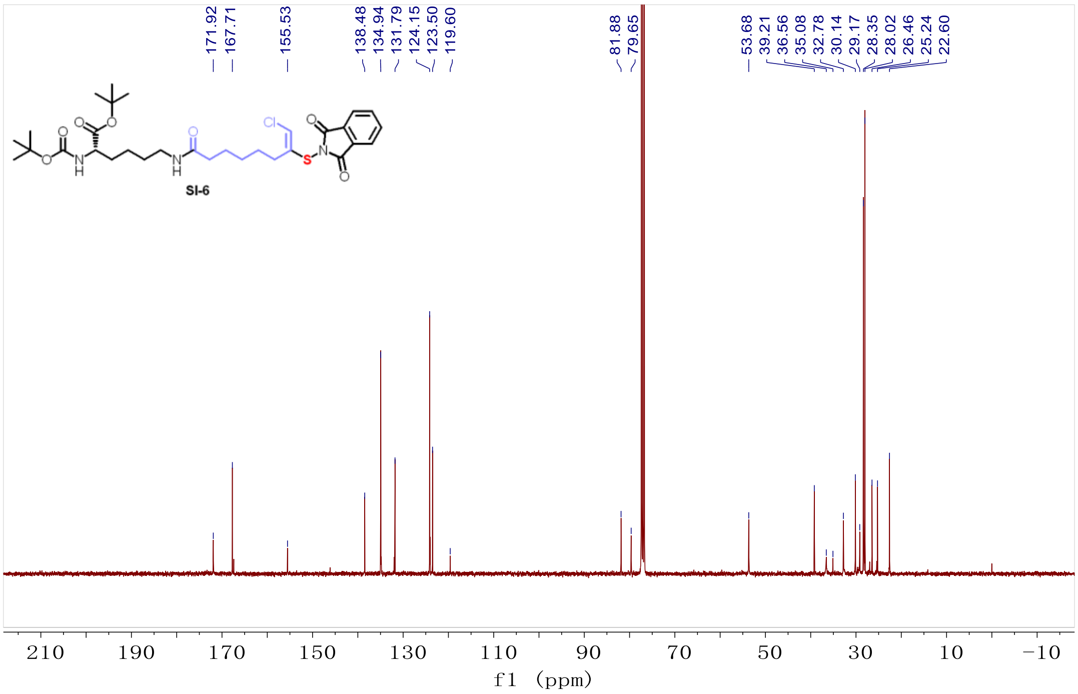

Figure S17: ^{13}C NMR 400 MHz $CDCl_3$ of **SI-6**

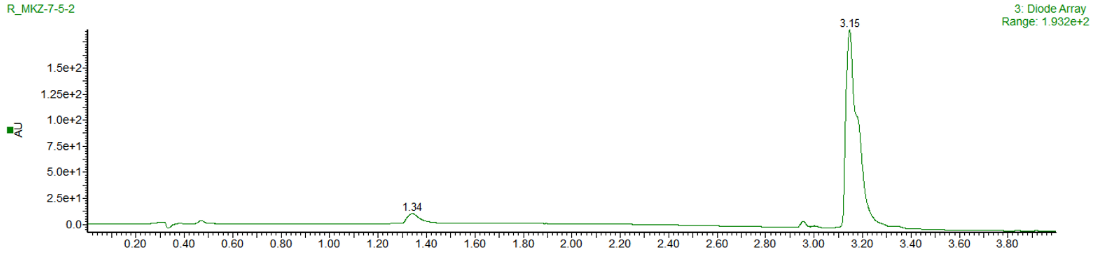

Figure S18: Ultraviolet absorption of **SI-6**

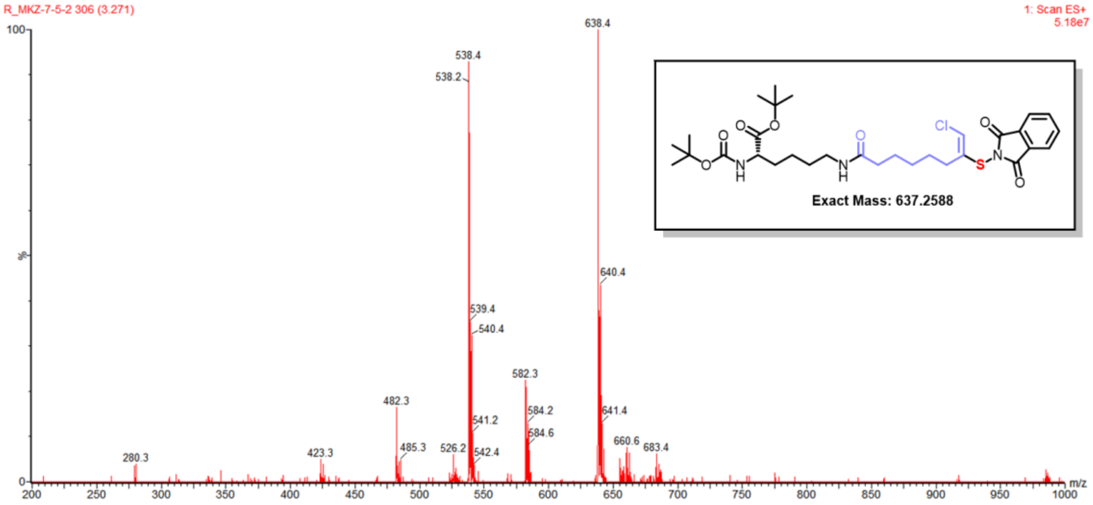

Figure S19: LC-MS molecular weight analysis of **SI-6**

Conclusion: Site-specific reactivity profiles between constituent amino acids and the staple-forming reagent provide corroborative evidence for the topological accuracy of the stapled peptide's constrained conformation.

VIII. Circular Dichroism Measurements.[22]

General Procedure for the measurement of CD spectra were mensurated at a wavelength ranging from 180 to 260 nm, with a path length of 1mm and a scanning speed of 200 nm/min at room temperature on the J-1500 (Jasco, Japan) spectropolarimeter. The lyophilized solid linear and staple peptides were dissolved in deionized water (bubbled with nitrogen for 30 minutes) to afford a 1 mM solution. The 1 mM stock solution was diluted with six different solvent systems (H_2O, 5%, 10%, 20%, 30%, 40% TFE/H_2O) to afford 0.05 mM solutions. The data described is the average of at least 3 values. The mean peptide ellipticity [θ] was calculated using equation (1) and plotted against wavelength.

$$[\theta] = \frac{\theta}{10 \times C \times Np \times I} \qquad (1)$$

θ: ellipticity [mdeg], C: peptide molar concentration [M], Np: number of peptide units, l: cell path length [cm].[6]

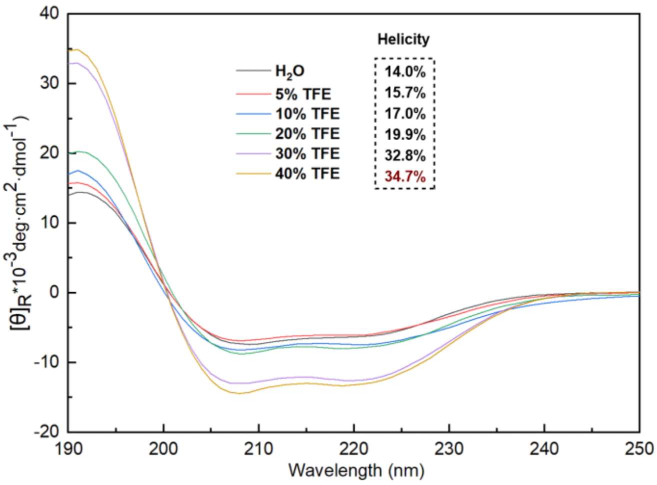

Figure S20: The CD spectroscopy of **5y** was measured in six different solvent systems

Through the above data, we obviously saw that the stapled peptide **5y** had the best helicity (**34.7%**) in 40% solution of TFE/H_2O, and then we conducted CD test on the active peptide QN-16 before and after stapling.

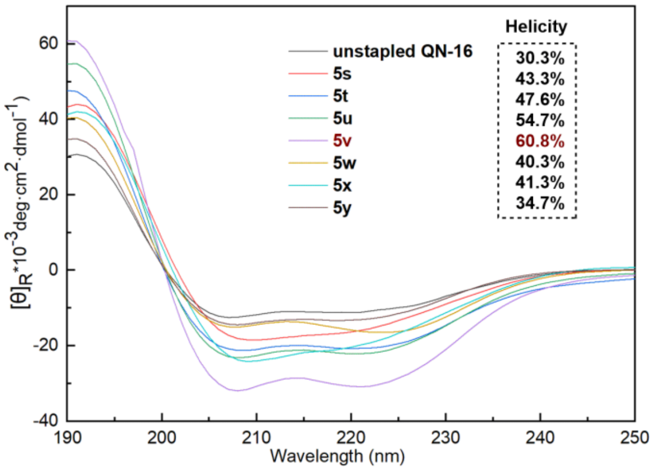

Figure S21: The CD spectroscopy of **QN-16** and its stapling peptide

Circular Dichroism curve of the unstapled (**QN-16**) and stapled peptide (**5s**, **5t**, **5u**, **5v**, **5w, 5x, 5y**) at 0.05 mM 40% TFE/H_2O. Interval for 190 to 250 nm.

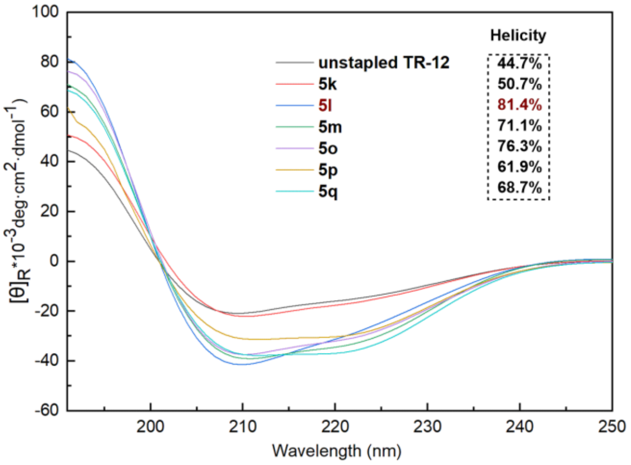

Figure S22: The CD spectroscopy of **TR-12** and its stapling peptide

Circular Dichroism curve of the unstapled (**TR-12**) and stapled peptide (**5k**, **5l**, **5m**, **5o**, **5p, 5q**) at 0.05 mM 40% TFE/H_2O. Interval for 190 to 250 nm.

IX. Proteolysis assays [23].

500 μL of peptide **TR-12** (500 μM, 2 μL DMSO was added to help solubilization) in phosphate buffer (pH 7.4) was mixed and incubated with 10 μL of the protease solution (Chymotrypsin – 100 μg/mL) at 37 °C. At 0, 10, 20, 40, 60, 90 minutes, aliquots of 50 μL were quenched with 50 μL of 1% TFA solution in MeCN respectively and subjected to HPLC analysis. Peptide concentrations at different time points were quantified by integration of the HPLC trace relative to the starting peptide sample. Results of these experiments are summarized in the Figure below.

500 μL of peptide **5o** (500 μM, 2 μL DMSO was added to help solubilization) in phosphate buffer (pH 7.4) was mixed and incubated with 10 μL of the protease solution (Chymotrypsin – 100 μg/mL) at 37 °C. At 0, 10, 20, 40, 60, 90 minutes, aliquots of 50 μL were quenched with 50 μL of 1% TFA solution in MeCN respectively and subjected to HPLC analysis. Peptide concentrations at different time points were quantified by integration of the HPLC trace relative to the starting peptide sample.

Results of these experiments are summarized in the Figure below:

Table S2. Proteolytic assays of peptide **TR-12** and **5o** with chymotrypsin.

Time/Peak area	0 min	10 min	20 min	40 min	60 min	90 min
**TR-12** + chymotrypin	11038124	5839000	4816000	2485000	1680000	1093000
**5o** + chymotrypin	12639189	10710000	9329000	7975000	7646000	7240000

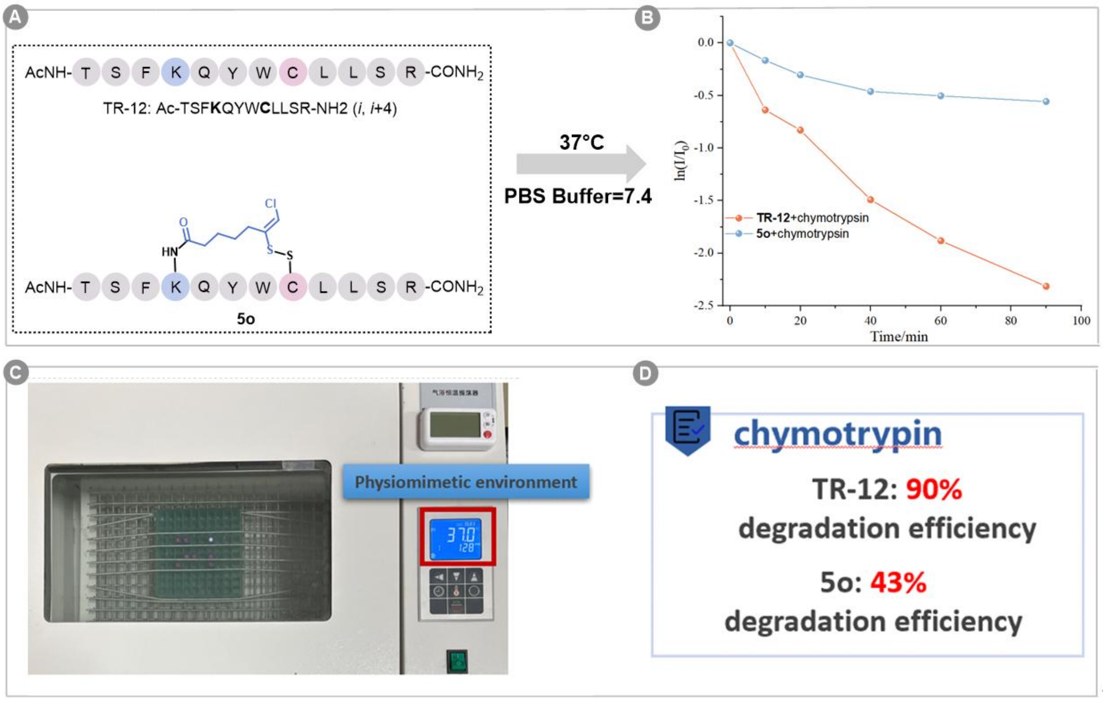

Figure S23. Proteolytic assays of peptide **TR-12** and **5o** with chymotrypsin. (a) Structure of stapled peptides **TR-12** and linear precursor **5o**. (b) Proteolytic degradation assay of **TR-12** and **5o**.

(c) Experiments were conducted under physiomimetic conditions using a 37℃ thermostatic orbital shaker system. (d) Degradation Efficiency Summary.

X. Serum stability study.

The peptide of interest (300 μL of 1 mg/mL stock solution in deionized water) was added to 300 μL of Foetal Bovine Serum and incubated at 37°C in an incubator. At each time point, an aliquot of 50 μL was drawn and an equal volume of ice-chilled CH_3CN was added to the aliquot to precipitate serum proteins. The cloudy mixture was centrifuged at 12,000 rpm for 10 min and then supernatant was removed. The level of degradation was determined by the integration of the area under the RP-HPLC peak corresponding to the intact peptide.

Before testing the serum stability, the standard curve (the relationship between concentration and peak area) was established by RP-HPLC for **CP-9** and **TR-12** and **QN-16** and **5h** and **5o** and **5w**.

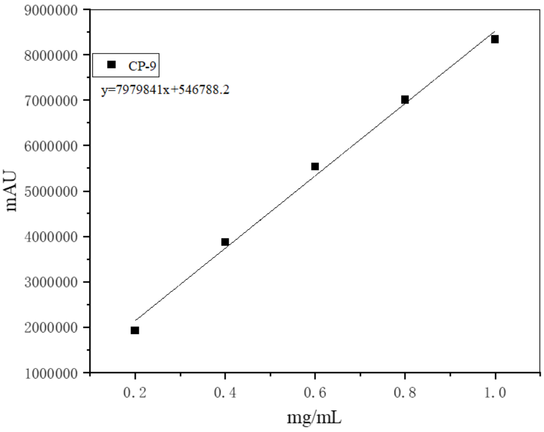

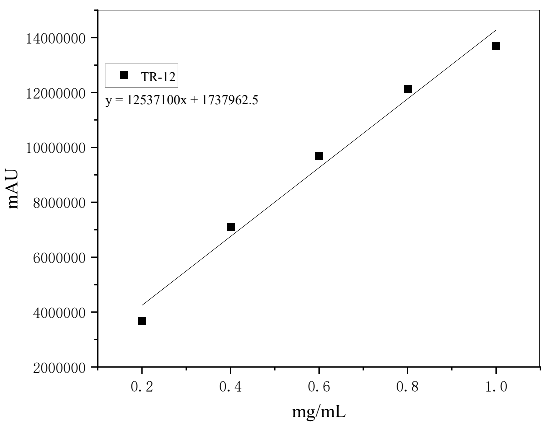

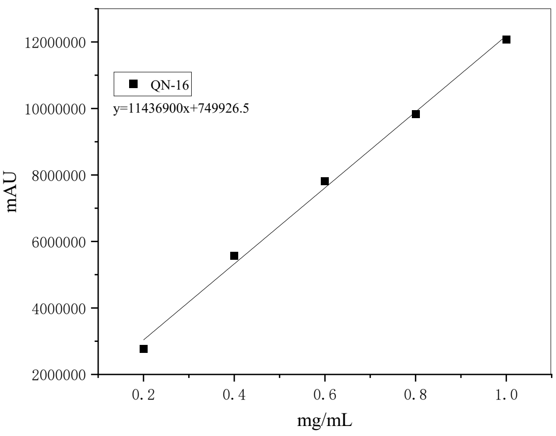

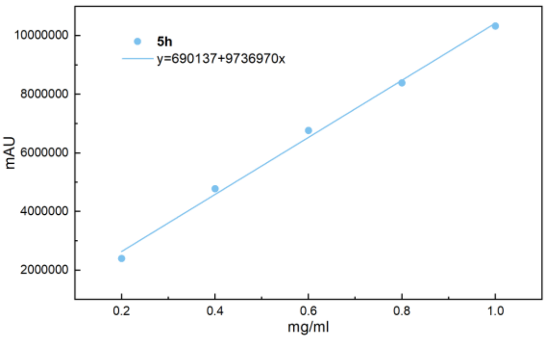

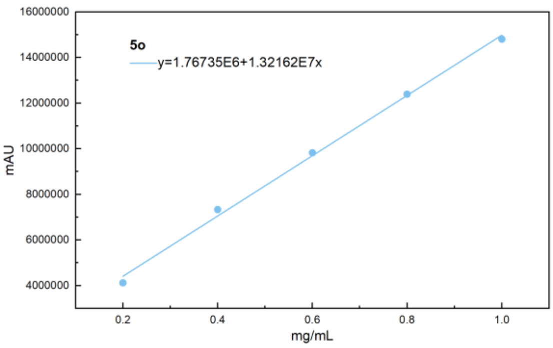

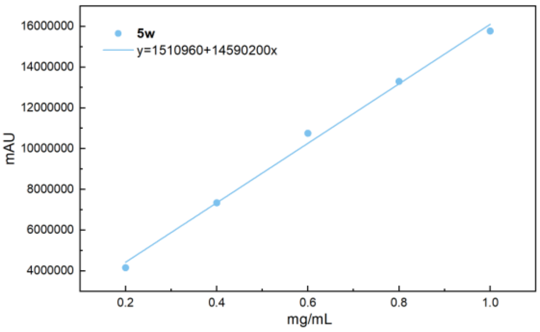

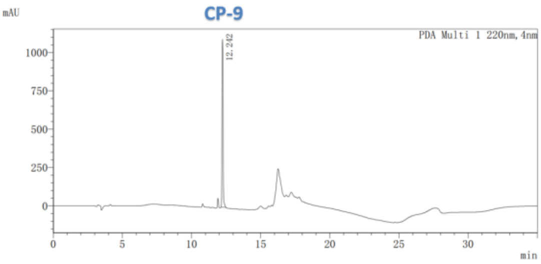

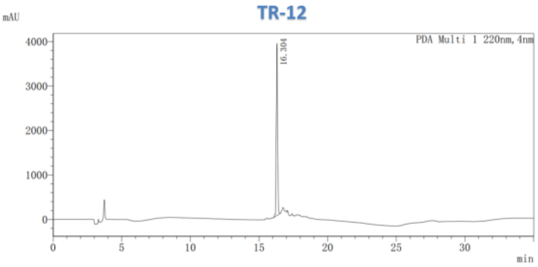

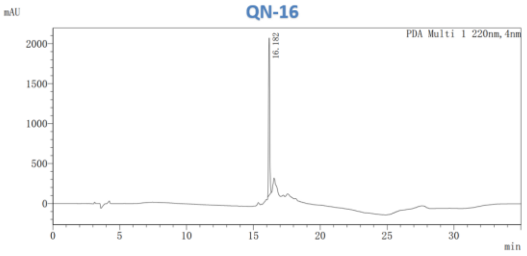

Note: Since the peaks of the previous methods CP-9, TR-12, and QN-16 appeared too early in HPLC, method A was re-run to shift their retention times later. This adjustment allows for a more accurate assessment of degradation in bovine serum during stability experiments.

Initially, we examined the serum stability of the linear peptide **CP-9** and stapled peptide **5h**.

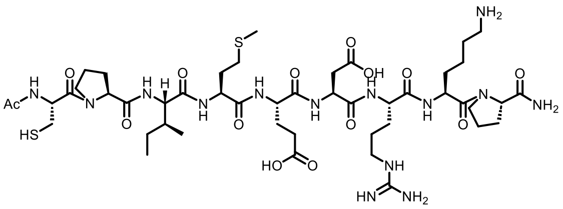

AcNH-CPIMEDRKP-CONH$_2$

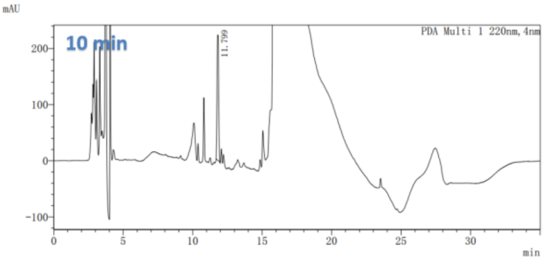

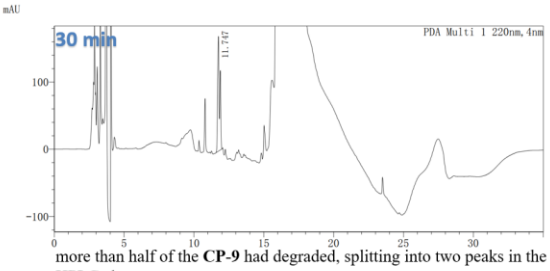

more than half of the **CP-9** had degraded, splitting into two peaks in the HPLC chromatogram

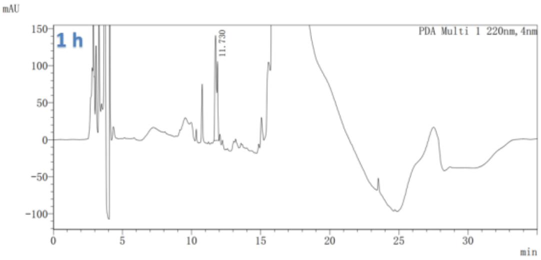

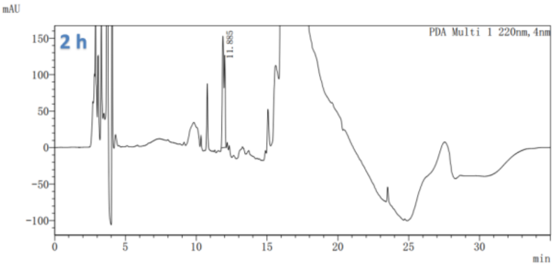

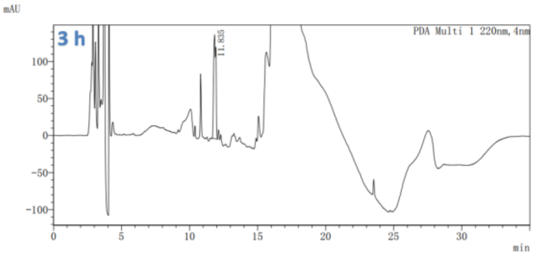

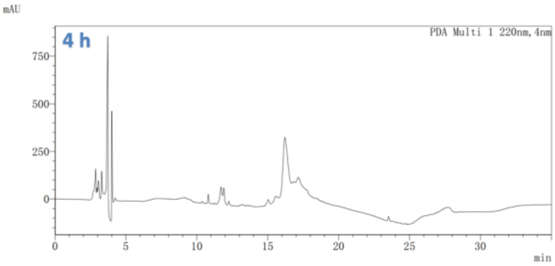

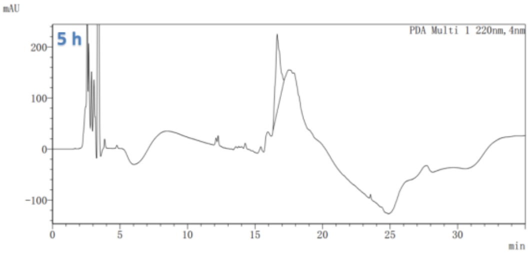

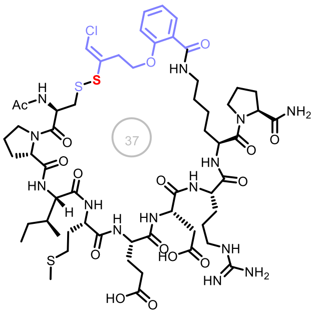

5h

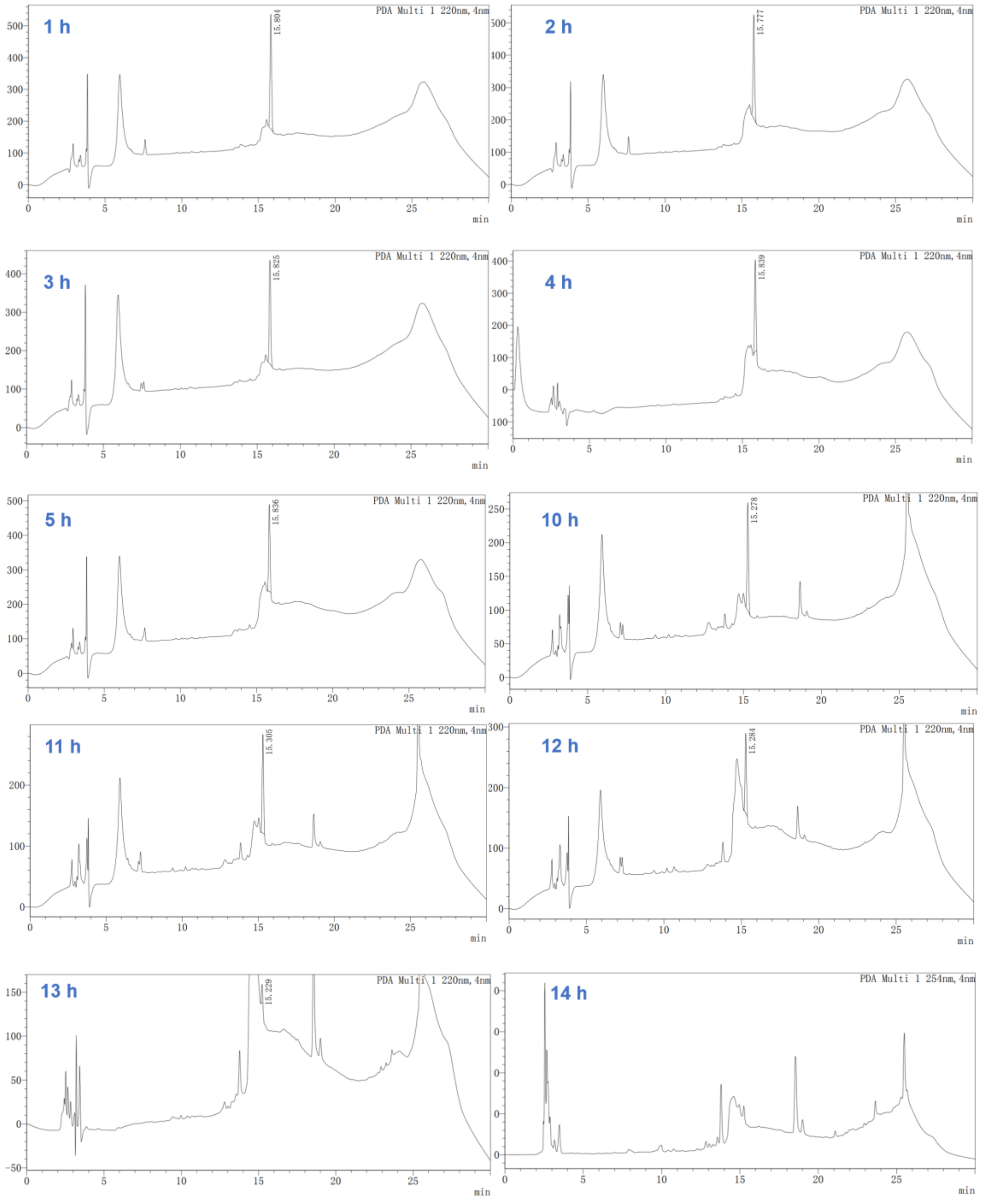

Figure S24: **CP-9** were degraded within 5 h; **5h** were degraded within 14 h

Next, we examined the serum stability of the linear peptide **TR-12** and stapled peptide **5o**.

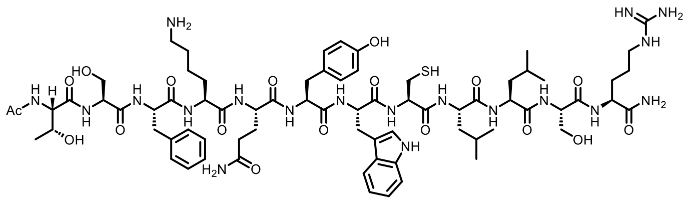

AcNH-TSFKQYWCLLSR-CONH$_2$

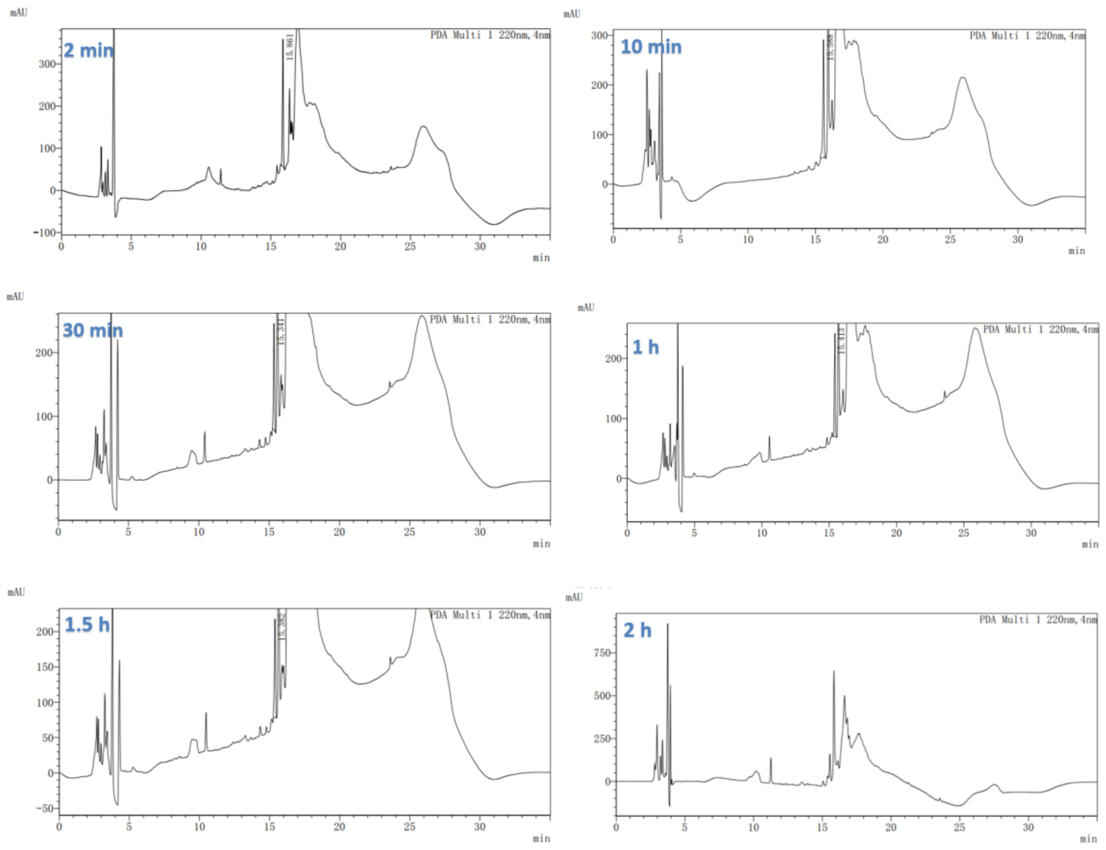
2 min
10 min
30 min
1 h
1.5 h
2 h
PDA Multi 1 220nm, 4nm
mAU
min

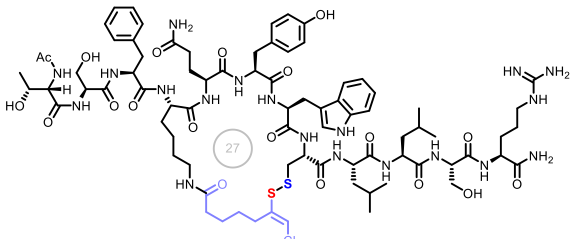
27

5o

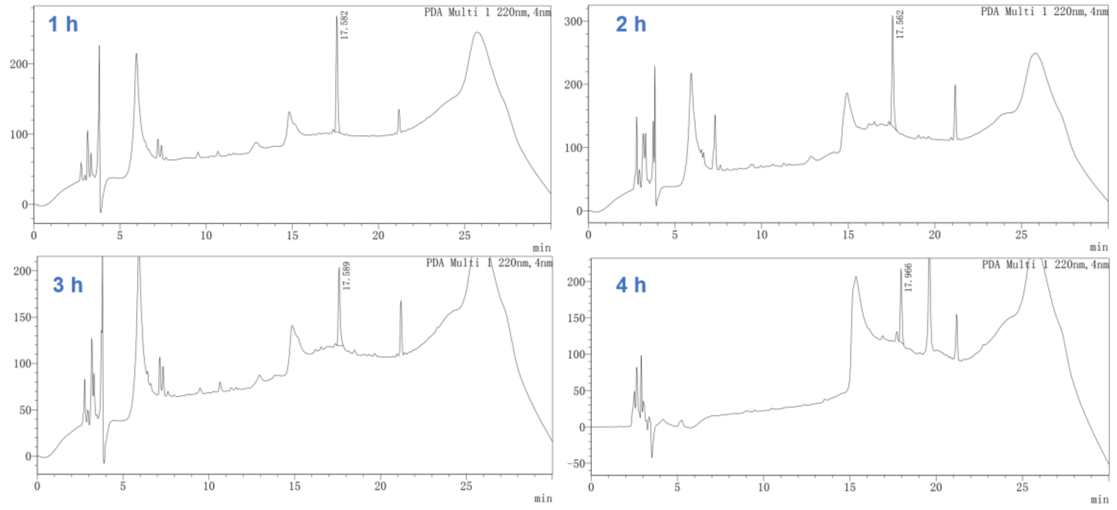
1 h
2 h
3 h
4 h
PDA Multi 1 220nm, 4nm
17.582
17.562
17.589
17.966
min

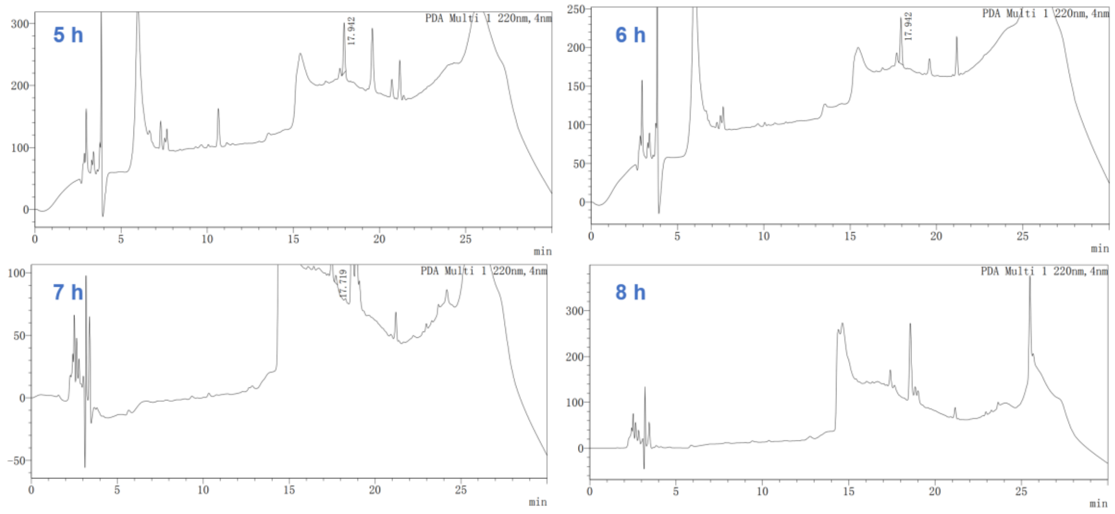

Figure S25: **TR-12** were degraded within 2 h; **5o** were degraded within 8 h

Ultimately, we examined the serum stability of the linear peptide **QN-16** and stapled peptide **5w**.

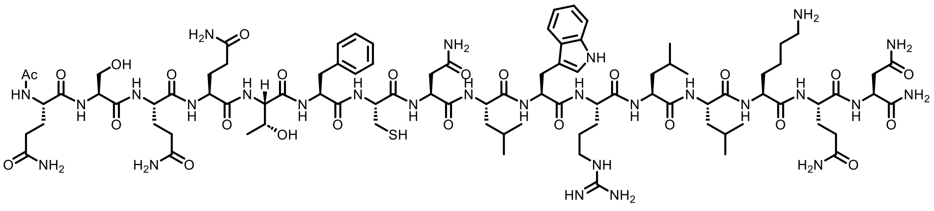

AcNH-QSQQTFCNLWRLLKQN-CONH$_2$

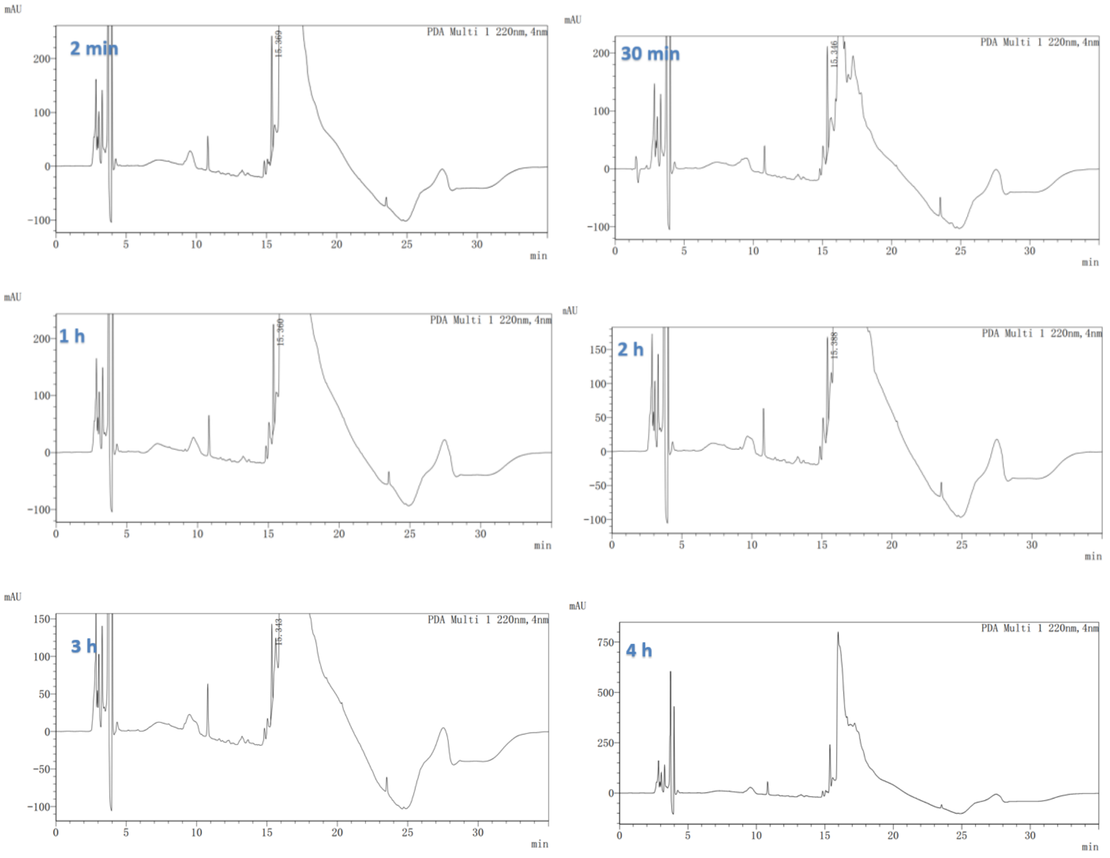

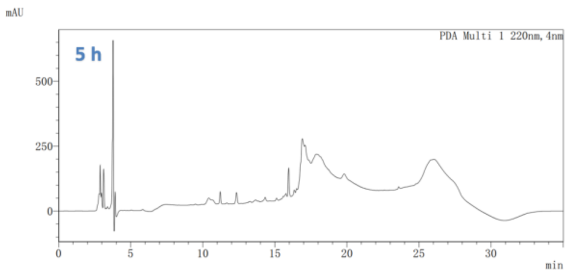

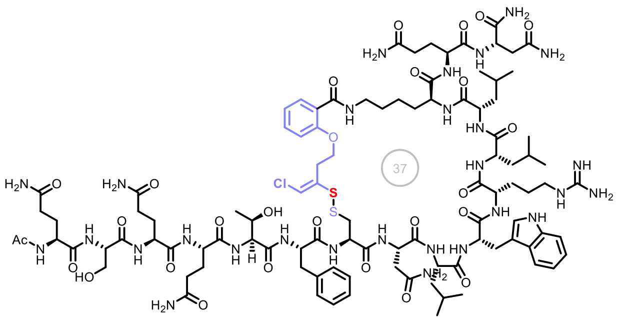

5w

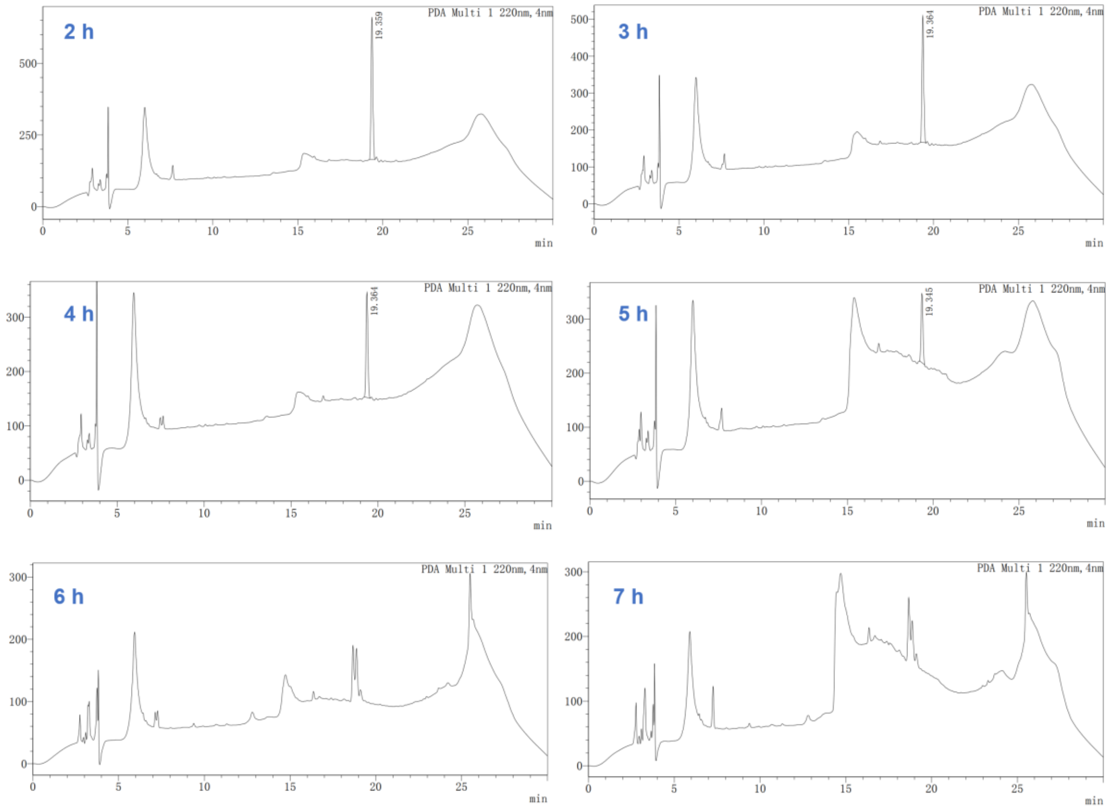

Figure S26: **QN-16** were degraded within 5 h; **5w** were degraded within 6 h

XI. Biological Experiments

Experimental purpose

Cell Counting Kit (CCK-8) was used to evaluate the proliferation inhibition activity of the compounds, and the half inhibitory concentration IC_{50} value was determined by single-concentration primary screening and multi-concentration.

Materials and reagents

Fetal Bovine Serum (FBS) 04-001-1acs, Biological industries; PBS phosphate buffer (PB180327), penicillin-streptomycin sulfate double resistant mixture (100 ×) (PB180120), RPMI1640 medium (PM150110), DMEM high glucose medium (PM150210), MEM medium (PM150410), McCoy's 5A medium (PM150710) and Leibovitz's L-15 medium (PM151010), Wuhan Punosai Life Technology Co., LTD. CCK-8 Kit, Biosharp life sciences; Trypsin-edta Digestive Solution (05200-056), GIBCO; Cellular DMSO (D2650-100ML), SIGMA; 96-well cell culture plate, Corning Life Sciences (Wujiang) Co., LTD. 25, 75cm2 cell culture bottle, Corning Life Sciences (Wujiang) Co., LTD. Blood cell counting plate, Shanghai Qiujing Biochemical Reagent Instrument Co., LTD. Cisplatin (D8810), Solarbio life sciences;

Instruments and equipment

Super clean workbench (sw-cj-2fd), Sujing Aetna; Microscope (nib-100), Ningbo Yongxin Optical Co., Ltd; Carbon dioxide cell incubator (MCO-18AC), Phcbi; Electric constant temperature water bath pot (HWS-24), Shanghai Yiheng Technology Co., Ltd; Multifunctional microplate reader (Multiskan MK3), Thermo; One ten-thousandth balance (MS105DU), METTLER; Centrifuge (TD4N), ChangshaYingtai Instrument Co., Ltd; Mixer (SCI-VS), Selo Czech Republic, USA.

Preparation of sample

The samples were dissolved in the cell-grade DMSO after accurately weighing to generate the 10 mM drug solution. The drug solution was further diluted to tenfold detection concentration by cell culture medium.

Standard Operation Procedure

Detection principle of cytotoxicity (CCK-8 method):

The detection principle is that the CCK-8 reagent contains WST-8, which is reduced S52 to a highly water-soluble yellow methyl product (formazan) by dehydrogenase in the cell mitochondria under the action of electron carrier 1-methoxy-5-methylphenazine dimethyl sulfate (1-methoxy PMS). The number of the nail products produced is directly proportional to the number of living cells.

Experimental method:

(1) Inoculate cells: The cells were prepared into single cell suspension with the culture medium containing 10% fetal bovine serum, and the 96 well plates were inoculated with 90 uL cell culture medium (Adherent cell viewed 5×104/mL and Suspension cell viewed 9×104/mL) per well, then cultured at 5% CO_2 and 37 °C for 24 hours.

(2) Add the sample solution to be tested: Add 10 μL sample solution to each well. One concentration was set for each sample during preliminary screening and three multiple holes were set for each concentration. Eight concentration gradients were set for each sample for IC_{50} determination and three multiple holes were set for each concentration. The 96 well plates were cultured at 5% CO_2 and 37 °C for 48 hours. The experiment was divided into a blank group, control group and drug group.

(3) Color development: The old culture medium and drug solution of adherent cells were sucked out, then 100 μL of CCK-8 solution (diluted ten times with the basic medium) was added and the suspension cells were directly added 10 μL of CCK-8 stock solution. Culture at 37 °C with 5% CO_2 for 1-4 h (dark operation, real-time observation).

(4) Result detection: The absorbance was measured at 450 nm with an enzyme labeling instrument and the original data and results were recorded.

(5) The toxicity is expressed by cell inhibition, and the calculation formula is as follows: Cell inhibition (%) = (ODControl-ODDrug) / (ODControl-ODBlank)×100%. The IC_{50} was calculated by the software graphpad prism 8 (version 8.0.2, from GraphPad SoftwareInc)， and the experimental results are expressed in ± SD.

6) Positive control: Cisplatin.

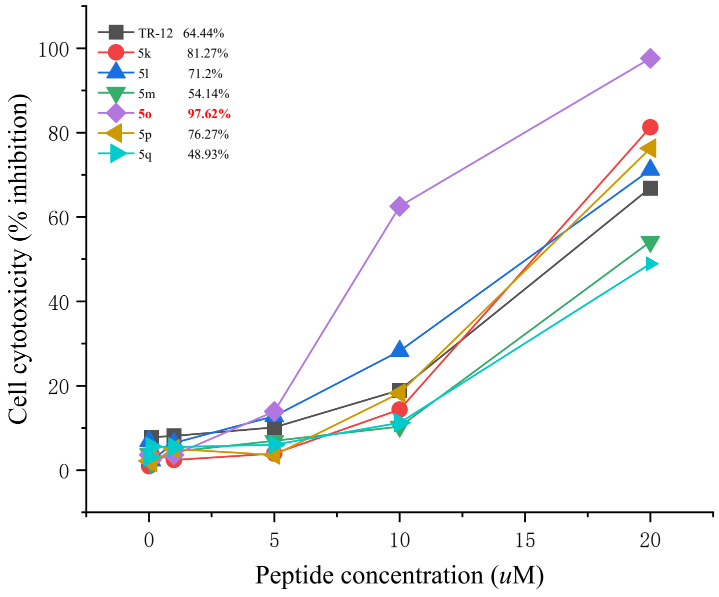

Figure S27. The anti-bladder cancer activity of the native peptide **TR-12** and its stapling peptides.

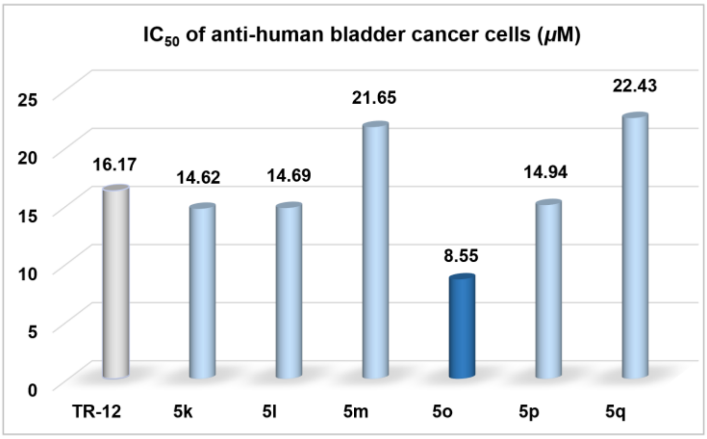

Figure S28. the IC_{50} values of the native peptide **TR-12** and its stapling peptides.

XII. BSA Macrocyclization [24]

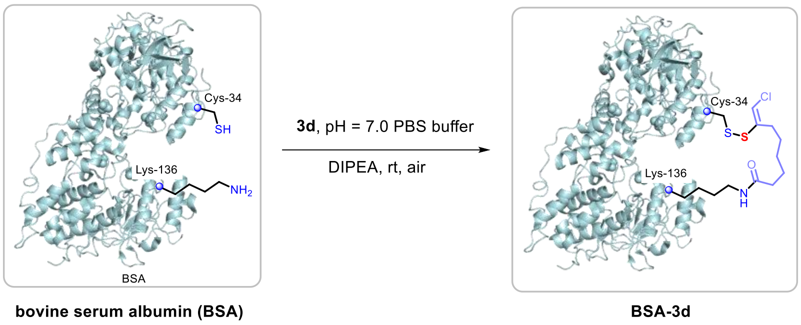

A solution of BSA (50 μM in water, 100 μL, 5.00 nmol, 1.0 equiv.) was added to PBS buffer (pH 7.0, 1 mL). Reagents **3d** (500 μM in ACN, 100 μL, 50 nmol, 10.0 equiv.) and DIPEA (500 μM in ACN, 100 μL, 50 nmol, 10.0 equiv.) were added, and the mixture was stirred at room temperature for 10 min. After centrifugation of the reaction mixture, 20 μL of the supernatant was collected and analyzed by MALDI-TOF-MS to determine the molecular weight.

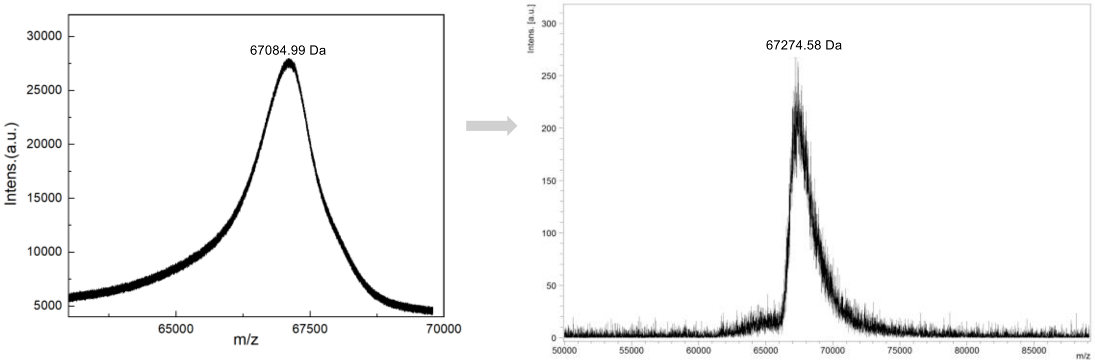

Figure S29. **BSA** MALDI-TOF m/z 67084.99 Da; **BSA-3d** MALDI-TOF m/z 67274.58 Da

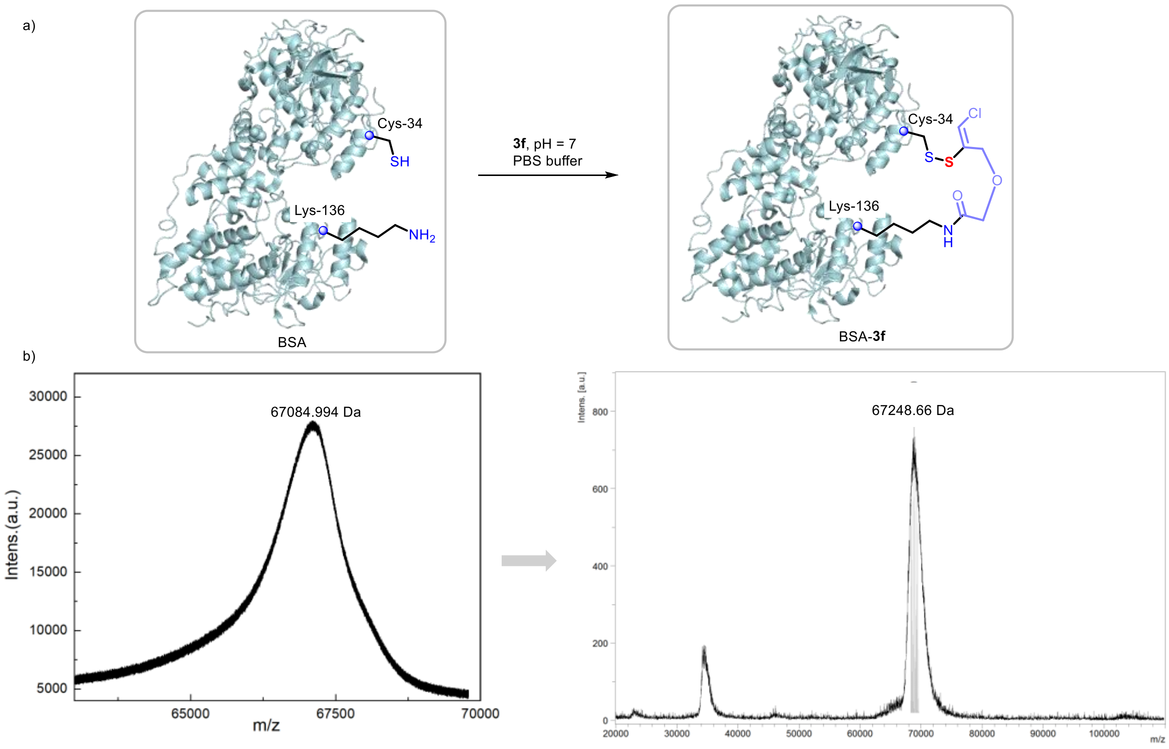

Figure S30. BSA Macrocyclization. (a) BSA before and after stapled. (b) Molecular weight of BSA before and after stapled.

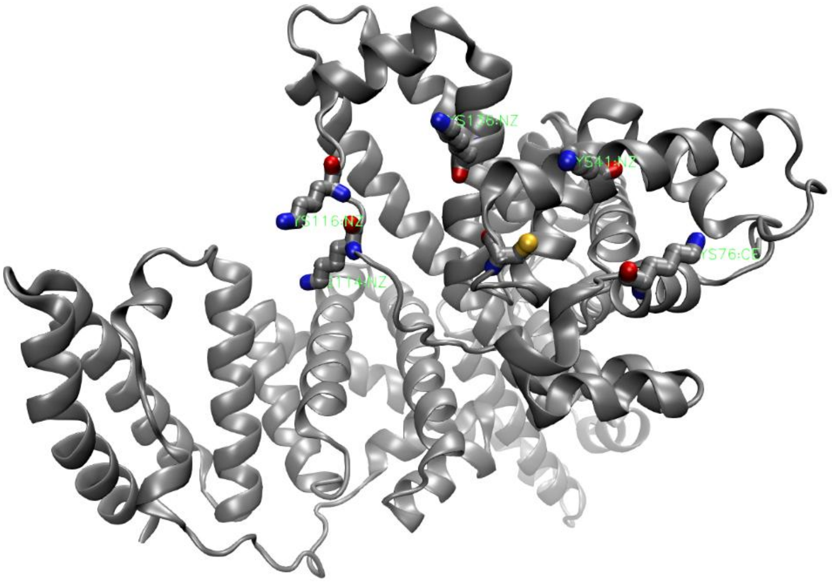

Figure S31. Based on the amino acid sequence of BSA, we predicted its structure using AlphaFold2 and visualized the distribution of lysine residues proximal to cysteine (Cys) residues in VMD software.

XIII. References

[1] T. Graf, J. Yoo, A. Brummett, R. Lin, M. Wohlgenannt, D. Quinn, N. Bowden, *Macromolecules* **2012**, *45*, 8193–8200.

[2] D. Zhu, Y. Gu, L. Lu, Q. Shen, *J. Am. Chem. Soc*. **2015**, *137*, 10547–10553.

[3] a) J. Chen, Palani, T. Hoye *J. Am. Chem. Soc.* **2016**, *138*, 4318–4321; b) A. Tran-Van, E. Huxol, J. Basler, M. Neuburger, J. Adjizian, C. Ewels, H. Wegner, *Org. Lett.* **2014**, *16*, 1594–1597; c) T. Gläsel, H. Jiao, M. Hapke, *ACS Catal.* **2021**, *11*, 13434–13444; d) J. Carney, P. Donoghue, O. Wuest, P. Helquist, *Org. Lett*. **2008**, *10*, 3903–3906.

[4] a) S. K. Monfared, M. R. Jafari, J. T. Patterson, P. Kitov, J. J. Dwyer, John M. Nuss, and R. Derda1, *Chem. Sci*., **2016**, *7*, 3785–3790; b) A. M. Spokoyny, Y. Zou, J. J. Ling, H. T. Yu, Y. S. Lin and B. L. Pentelute, *J. Am. Chem. Soc*. **2013**, *135*, 5946−5949.

[5] Q. Yu, L. Bai, X. Jiang, *Angew. Chem. Int. Ed*. **2023**, *62*, e202314379.

[6] M. J. Frisch et. al. Gaussian, Inc., Wallingford CT, **2016**.

[7] F. Neese Software update: The ORCA program system—Version 5.0. WIREs Comput Mol Sci. **2022**, *12*, 1606.

[8] (a) A. D. Becke, *J. Chem. Phys.* **1993**, 98, 5648-5652. (b) S. Grimme, S. Ehrlich, L. Goerigk, *J. Comput. Chem.* **2011**, *32*, *7*, 1456–1465.

[9] (a) S. Miertuš, E. Scrocco, *J. Tomasi, Chem. Phys.* **1981**, *55*, 117–129. (b) S. Miertuš, *J. Tomasi, Chem. Phys.* **1982**, *65*, 239–245. (c) J. L. Pascual-Ahuir, E. Silla, I. Tuñón, *J. Comp. Chem.* **1994**, *15*, 1127–1138. (d) J. Tomasi, B. Mennucci, R. Cammi, *Chem. Rev.* **2005**, *105*, 2999–3093.

[10] (a) A. Schaefer, H. Horn, R. Ahlrichs, *J. Chem. Phys.* **1992**, *97*, 2571–2577. (b) A. Schaefer, C. Huber, R. Ahlrichs, *J. Chem. Phys.* **1994**, *100*, 5829–5835. (c) F. Weigend, R. Ahlrichs, *Phys. Chem. Chem. Phys.* **2005**, *7*, 3297–3305. (d) F. Weigend, *Phys. Chem. Chem. Phys.* **2006**, *8*, 1057–1065.

[11] T. Lu, Q. Chen, *Comput. Theor. Chem.* **2021**, *1200*, 113249.

[12] (a) K. Fukui, *Acc. Chem. Res*. **1981**, *14*, 363–368. (b) H. P. Hratchian, H. B. Schlegel, in Theory and Applications of Computational Chemistry: The First 40 Years, Ed. C. E. Dykstra, G. Frenking, K. S. Kim, G. Scuseria, *Elsevier, Amsterdam*, **2005**, 195–249.

[13] L. Goerigk, S. Grimme, *J. Chem. Theory Comput.* **2011**, *7*, *2*, 291–309.

[14] A. V. Marenich, C. J. Cramer, D. G. Truhlar, *J. Phys. Chem. B*, **2009**, *113*, 6378–6396.

[15] (a) E. Papajak, J. Zheng, X. Xu, H. R. Leverentz, D. G. Truhlar, *J. Chem. Theory Comput.* **2011**, *7*, *10*, 3027–3034. (b) J. Zheng, X. Xu, D. G. Truhlar, *Theor Chem Acc*, **2011**, *128*, 295–305.

[16] J. Zhang, T. Lu, *Phys. Chem. Chem. Phys.* **2021**, *23*, 20323.

[17] T. Brinck, P. Caelqvist, J. H. Stenlid, *J. Phys. Chem. A.* **2016**, *120*, 10023.

[18] T. Lu, Q. Chen, WILEY-VCH GmbH: *Weinheim*, **2022**, 631–647.

[19] a) T. Lu, F. Chen, *J. Comput. Chem.* **2012**, *33*, 580–592. b) T. Lu, *J. Chem. Phys.* **2024**, *161*,

082503.

[20] W. Humphrey, A. Dalke, K. Schulten, *J. Molec. Graphics*, **1996**, *14*, 33–38.

[21] CYLview20; C. Y. Legault, Université de Sherbrooke, **2020** (http://www.cylview.org).

[22] N. Shepherd, H. Hoang, G. Abbenante, D. Fairlie, *J. Am. Chem. Soc*. **2005**, *127*, 2974–2983.

[23] M. Wang, D. Pan, Q. Zhang, Y. Lei, C. Wang, H. Jia, L. Mou, X. Miao, X. Ren, Z. Xu, *J. Am. Chem. Soc.* **2024**, *146*, 6675–6685.

[24] a) M. J. S. A. Silva, H. Faustino, J. A. S. Coelho, M. V. Pinto, A. Fernandes, I. Compañón, F. Corzana, G. Gasser, P. M. P. Gois, *Angew. Chem. Int. Ed*. **2021**, *60*, 10850–10857; b) Q. Yu, X. Zhang, X. Jiang, *Angew. Chem. Int. Ed*. **2024**, *63*, e202408158.

XIV. X-ray Crystal Data of 3a (CCDC 2405078).

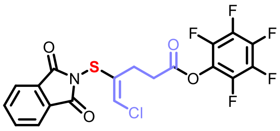

3a

≡

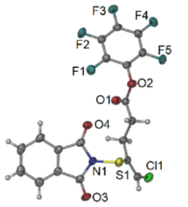

X-ray of **3a**

Datablock: exp_3751_auto

Bond precision: C-C = 0.0043 A Wavelength=1.54184

Cell: a=9.9278(10) b=10.2997(10) c=11.4351(12)
alpha=67.234(9) beta=70.293(9) gamma=66.582(9)

Temperature: 173 K

	Calculated	Reported
Volume	965.85(19)	965.8(2)
Space group	P -1	P -1
Hall group	-P 1	-P 1
Moiety formula	C19 H9 Cl F5 N O4 S	C19 H9 Cl F5 N O4 S
Sum formula	C19 H9 Cl F5 N O4 S	C19 H9 Cl F5 N O4 S
Mr	477.78	477.78
Dx, g cm-3	1.643	1.643
Z	2	2
Mu (mm-1)	3.479	3.479
F000	480.0	480.0
F000'	483.23	
h, k, lmax	11, 12, 13	11, 12, 13
Nref	3451	3420
Tmin, Tmax	0.378, 0.465	0.469, 1.000
Tmin'	0.286	

Correction method= # Reported T Limits: Tmin=0.469 Tmax=1.000 AbsCorr = MULTI-SCAN

Data completeness= 0.991 Theta(max)= 67.028

R(reflections)= 0.0561(2824) wR2(reflections)= 0.1633(3420)

S = 1.059 Npar= 281

```
The following ALERTS were generated. Each ALERT has the format
       test-name_ALERT_alert-type_alert-level.
Click on the hyperlinks for more details of the test.
```

Alert level C

```
PLAT340_ALERT_3_C Low Bond Precision on  C-C Bonds ...............    0.00433 Ang.
PLAT911_ALERT_3_C Missing FCF Refl Between Thmin & STh/L=    0.597          31 Report
                   1  2  0,   3  2  0,   1 -2  1,   1 -1  1,   1  0  2,   0  1  2,
                  -7  6  2,  -6  7  2,   8 -4  3,  -6  6  3,  -6  7  3,  -5  8  3,
                   0  2  4,  -6  6  4,  -5  6  4,  -5  7  4,  -6  6  5,  -5  6  5,
                  -5  7  5,  -4  8  5,   4 12  5,  -1 -8  6,  11  4  6,  -5  7  6,
                  -4  8  6,   3 12  6,   4 12  7,   8 -1  8,  10  7  8,  11  6  9,
                   1 -4 10,
```

Alert level G

```
PLAT072_ALERT_2_G SHELXL First  Parameter in WGHT  Unusually Large       0.10 Report
PLAT154_ALERT_1_G The s.u.'s on the Cell Angles are Equal ..(Note)      0.009 Degree
PLAT432_ALERT_2_G Short Inter X...Y Contact  O1         ..C7        .    2.92 Ang.
                                         1-x,1-y,1-z  =       2_666 Check
PLAT432_ALERT_2_G Short Inter X...Y Contact  O1         ..C6        .    2.94 Ang.
                                         1-x,1-y,1-z  =       2_666 Check
PLAT909_ALERT_3_G Percentage of I>2sig(I) Data at Theta(Max) Still        65% Note
PLAT933_ALERT_2_G Number of HKL-OMIT Records in Embedded .res File          2 Note
                   0  1  2,   1  0  2,
PLAT941_ALERT_3_G Average HKL Measurement Multiplicity ...........        2.7 Low
PLAT969_ALERT_5_G The 'Henn et al.' R-Factor-gap value ...........      2.079 Note
                 Predicted wR2: Based on SigI**2  7.85 or SHELX Weight 15.42
PLAT978_ALERT_2_G Number C-C Bonds with Positive Residual Density.          3 Info
PLAT992_ALERT_5_G Repd & Actual _reflns_number_gt Values Differ by          2 Check
```

```
  0 ALERT level A = Most likely a serious problem - resolve or explain
  0 ALERT level B = A potentially serious problem, consider carefully
  2 ALERT level C = Check. Ensure it is not caused by an omission or oversight
 10 ALERT level G = General information/check it is not something unexpected

  1 ALERT type 1 CIF construction/syntax error, inconsistent or missing data
  5 ALERT type 2 Indicator that the structure model may be wrong or deficient
  4 ALERT type 3 Indicator that the structure quality may be low
  0 ALERT type 4 Improvement, methodology, query or suggestion
  2 ALERT type 5 Informative message, check
```

X-ray of crystal data of **3a**. Supplementary crystallographic data for this compound have been deposited at Cambridge Crystallographic Data Centre (CCDC 2405078) and can be obtained free of charge via www.ccdc.cam.ac.uk/data_request/cif. **X-ray**

Crystal Data of 3n (CCDC 2405068).

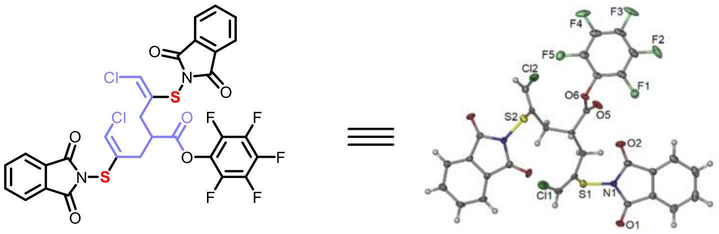

Datablock: exp_3991_auto

Bond precision:	C-C = 0.0040 A		Wavelength=1.54184
Cell:	a=15.0049(1)	b=14.4551(1)	c=31.5644(2)
	alpha=90	beta=90	gamma=90
Temperature: 173 K			

	Calculated	Reported
Volume	6846.23(8)	6846.23(8)
Space group	P b c a	P b c a
Hall group	-P 2ac 2ab	-P 2ac 2ab
Moiety formula	C30 H15 Cl2 F5 N2 O6 S2, C H Cl3	C30 H15 Cl2 F5 N2 O6 S2, C H Cl3
Sum formula	C31 H16 Cl5 F5 N2 O6 S2	C31 H16 Cl5 F5 N2 O6 S2
Mr	848.83	848.83
Dx, g cm-3	1.647	1.647
Z	8	8
Mu (mm-1)	5.679	5.679
F000	3408.0	3408.0
F000'	3438.08	
h, k, lmax	17, 17, 37	17, 17, 37
Nref	6106	6103
Tmin, Tmax	0.194, 0.228	0.194, 1.000
Tmin'	0.086	

Correction method= # Reported T Limits: Tmin=0.194 Tmax=1.000 AbsCorr = MULTI-SCAN

Data completeness= 1.000 Theta(max)= 67.073

R(reflections)= 0.0496(5669) wR2(reflections)= 0.1351(6103)

S = 1.074 Npar= 460

```
The following ALERTS were generated. Each ALERT has the format
       test-name_ALERT_alert-type_alert-level.
Click on the hyperlinks for more details of the test.
```

Alert level C

```
PLAT244_ALERT_4_C Low    'Solvent' Ueq as Compared to Neighbors of        C31 Check
PLAT431_ALERT_2_C Short Inter HL..A Contact  C12       ..S2        .       3.42 Ang.
                                         1/2-x,-1/2+y,z  =       8_655 Check
PLAT906_ALERT_3_C Large K Value in the Analysis of Variance ......      2.387 Check
PLAT911_ALERT_3_C Missing FCF Refl Between Thmin & STh/L=     0.597           3 Report
                 2   0   0,    1  17   1,    3   1   3,
```

Alert level G

```
PLAT083_ALERT_2_G SHELXL Second Parameter in WGHT  Unusually Large        11.53 Why ?
PLAT142_ALERT_4_G s.u.  on b - Axis Small or Missing .............     0.00010 Ang.
PLAT143_ALERT_4_G s.u.  on c - Axis Small or Missing .............     0.00020 Ang.
PLAT909_ALERT_3_G Percentage of I>2sig(I) Data at Theta(Max) Still          84% Note
PLAT933_ALERT_2_G Number of HKL-OMIT Records in Embedded .res File            2 Note
                 2   0   0,    3   1   3,
PLAT969_ALERT_5_G The 'Henn et al.' R-Factor-gap value ..........         3.708 Note
               Predicted wR2: Based on SigI**2  3.64 or SHELX Weight 12.58
PLAT978_ALERT_2_G Number C-C Bonds with Positive Residual Density.            9 Info
```

```
  0 ALERT level A = Most likely a serious problem - resolve or explain
  0 ALERT level B = A potentially serious problem, consider carefully
  4 ALERT level C = Check. Ensure it is not caused by an omission or oversight
  7 ALERT level G = General information/check it is not something unexpected

  0 ALERT type 1 CIF construction/syntax error, inconsistent or missing data
  4 ALERT type 2 Indicator that the structure model may be wrong or deficient
  3 ALERT type 3 Indicator that the structure quality may be low
  3 ALERT type 4 Improvement, methodology, query or suggestion
  1 ALERT type 5 Informative message, check
```

X-ray of crystal data of **3n**. Supplementary crystallographic data for this compound have been deposited at Cambridge Crystallographic Data Centre (CCDC 2405068) and can be obtained free of charge via www.ccdc.cam.ac.uk/data_request/cif. **X-ray**

XV. NMR Spectra of Stapling Reagents

^{1}H NMR 400 MHz $CDCl_3$ of **3a**

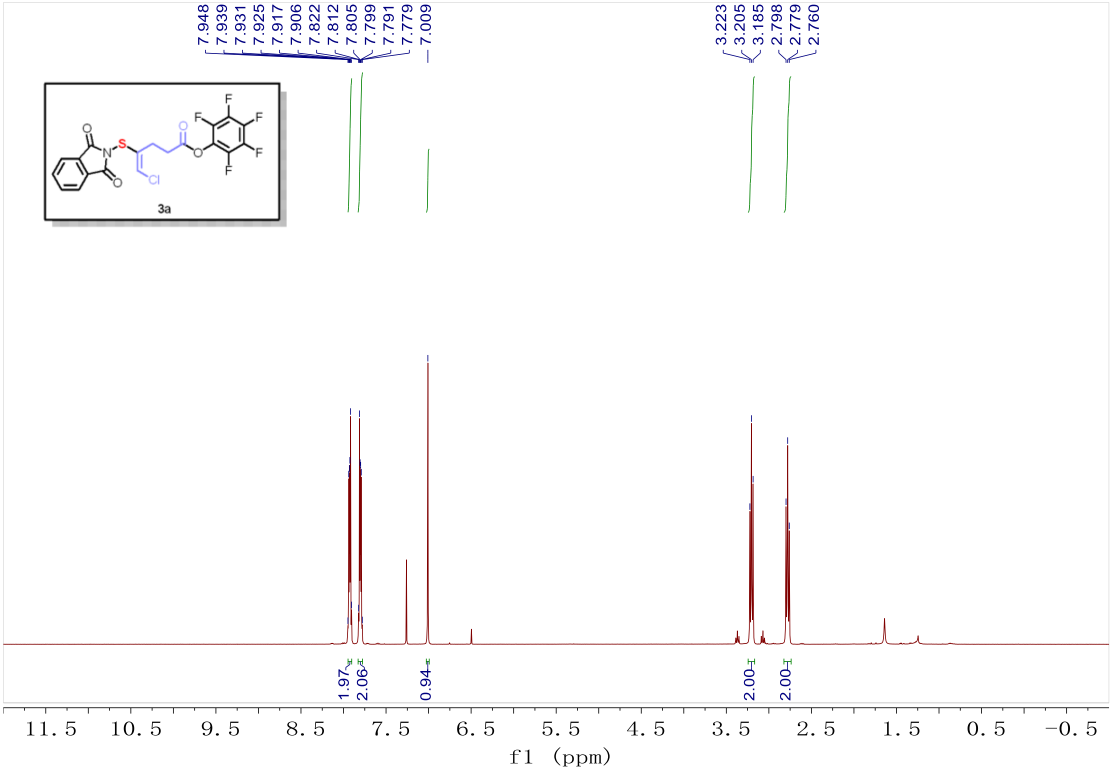

^{13}C NMR 125 MHz $CDCl_3$ of **3a**

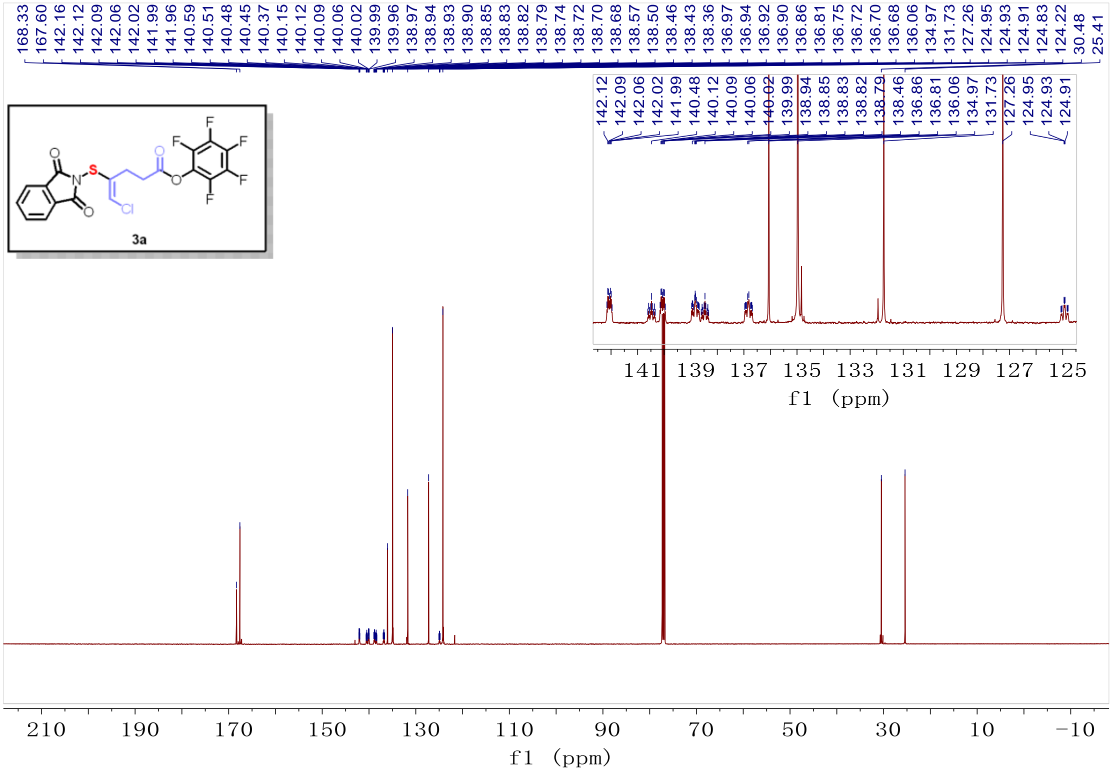

^{19}F NMR 376 MHz CDCl$_3$ of **3a**

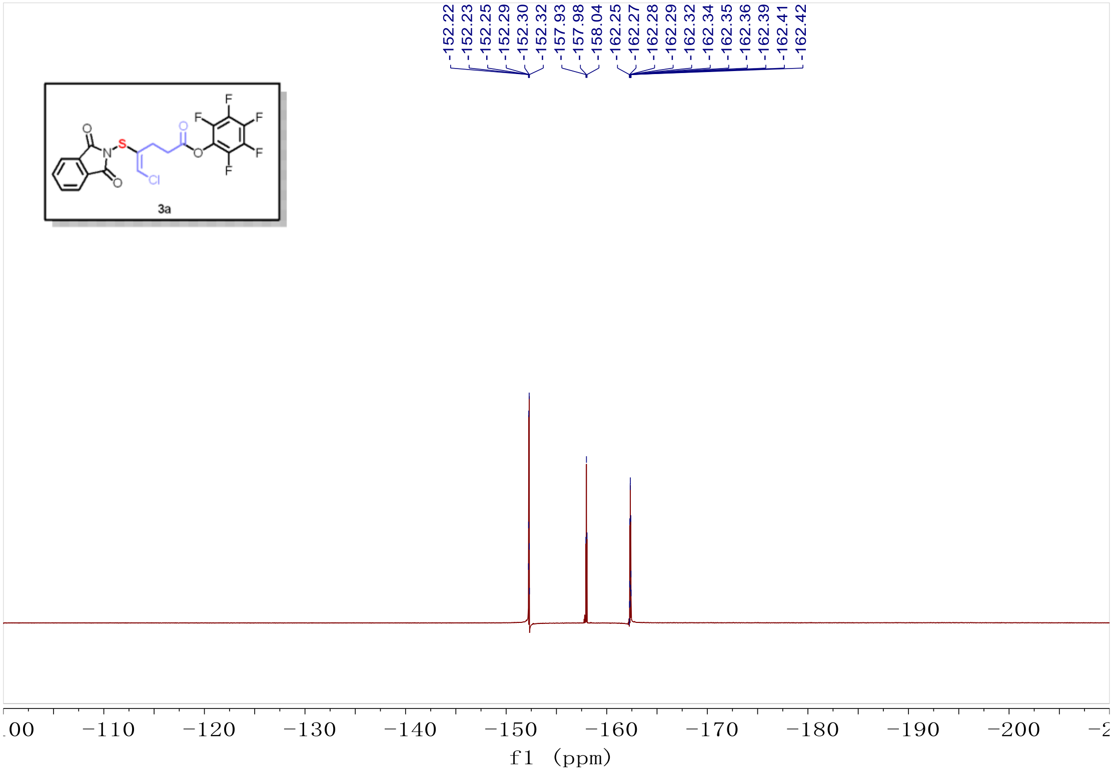

^{1}H NMR 400 MHz CDCl$_3$ of **3b**

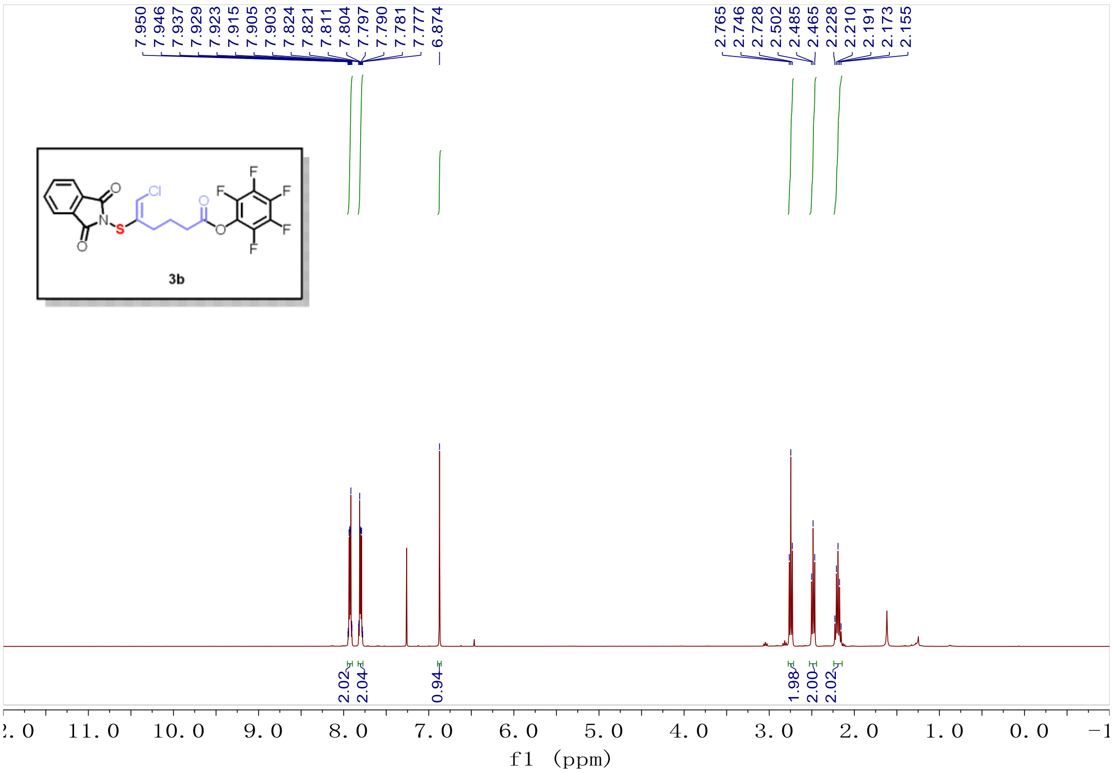

^{13}C NMR 125 MHz $CDCl_3$ of **3b**

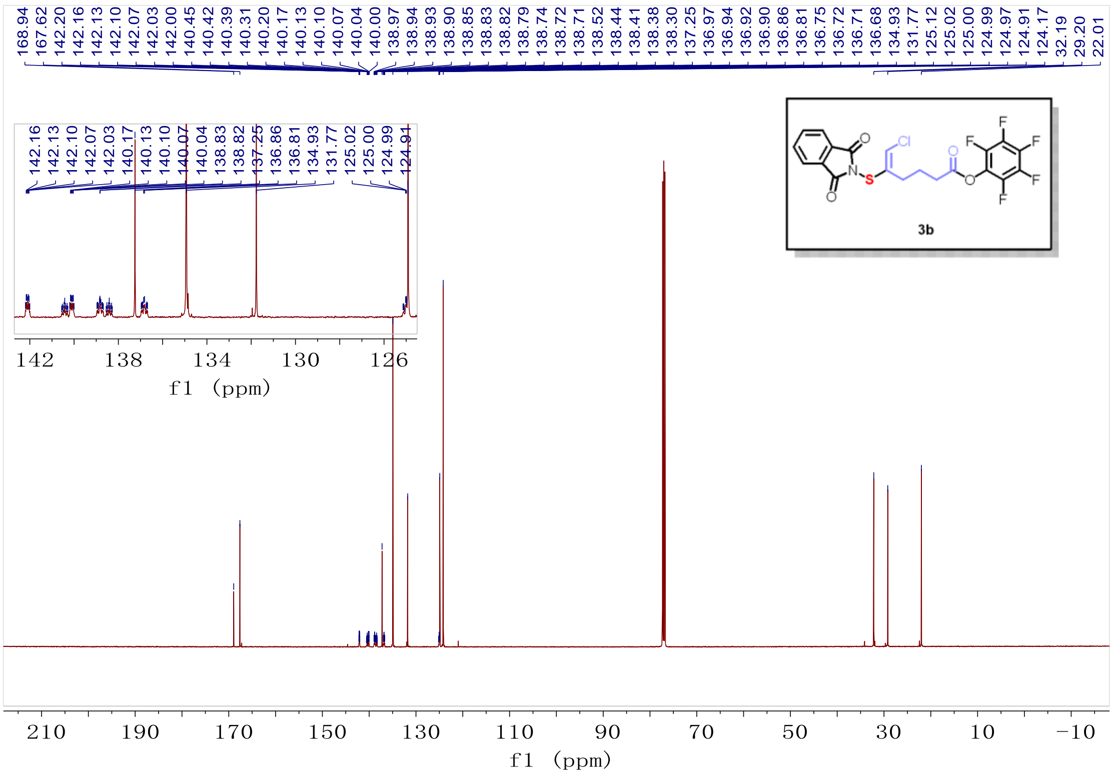

^{19}F NMR 376 MHz $CDCl_3$ of **3b**

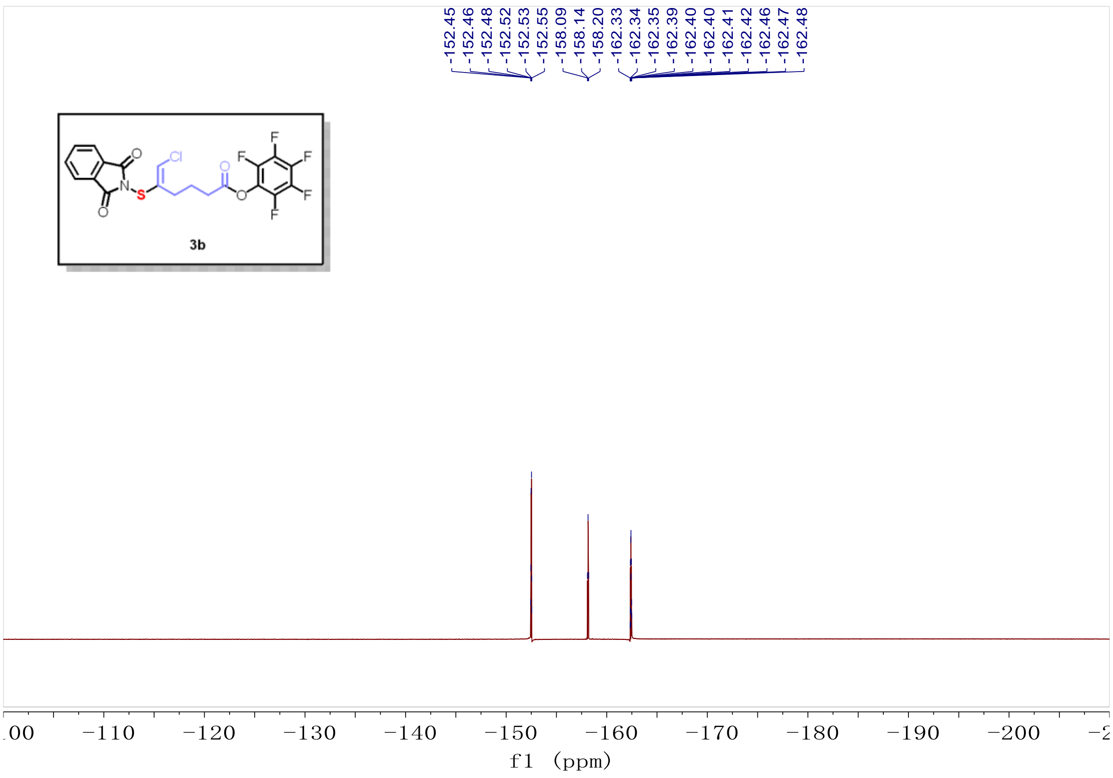

^{1}H NMR 400 MHz $CDCl_3$ of **3c**

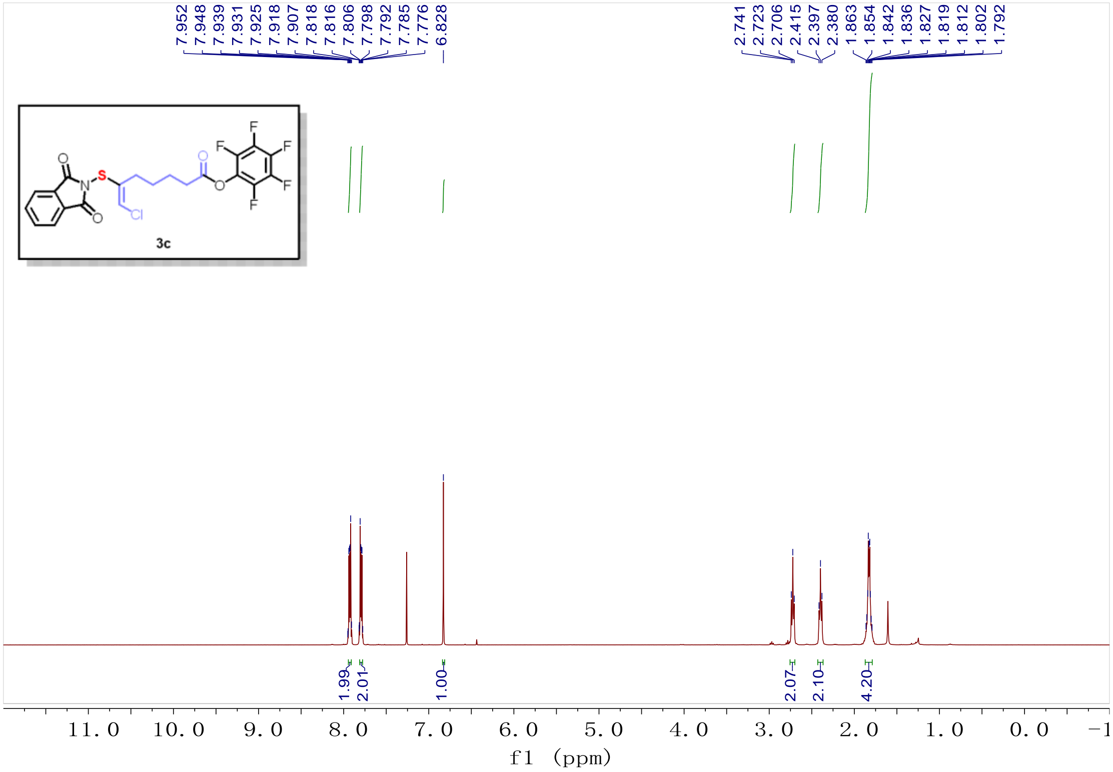

^{13}C NMR 125 MHz $CDCl_3$ of **3c**

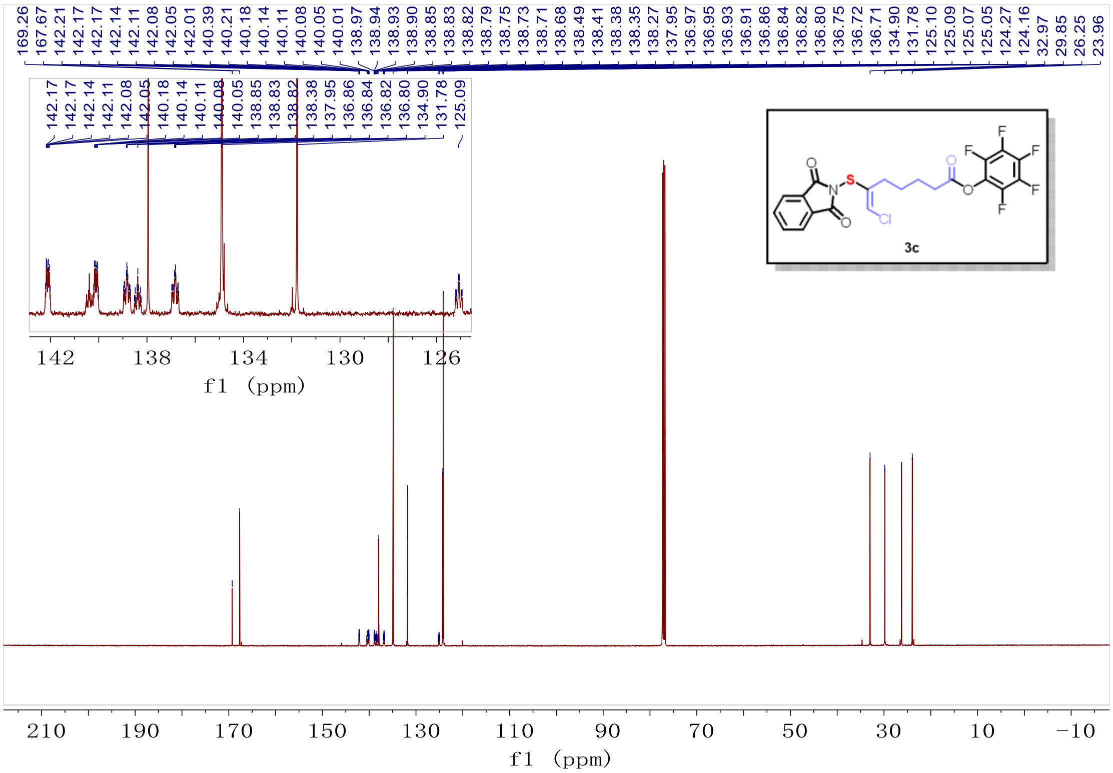

^{19}F NMR 376 MHz $CDCl_3$ of **3c**

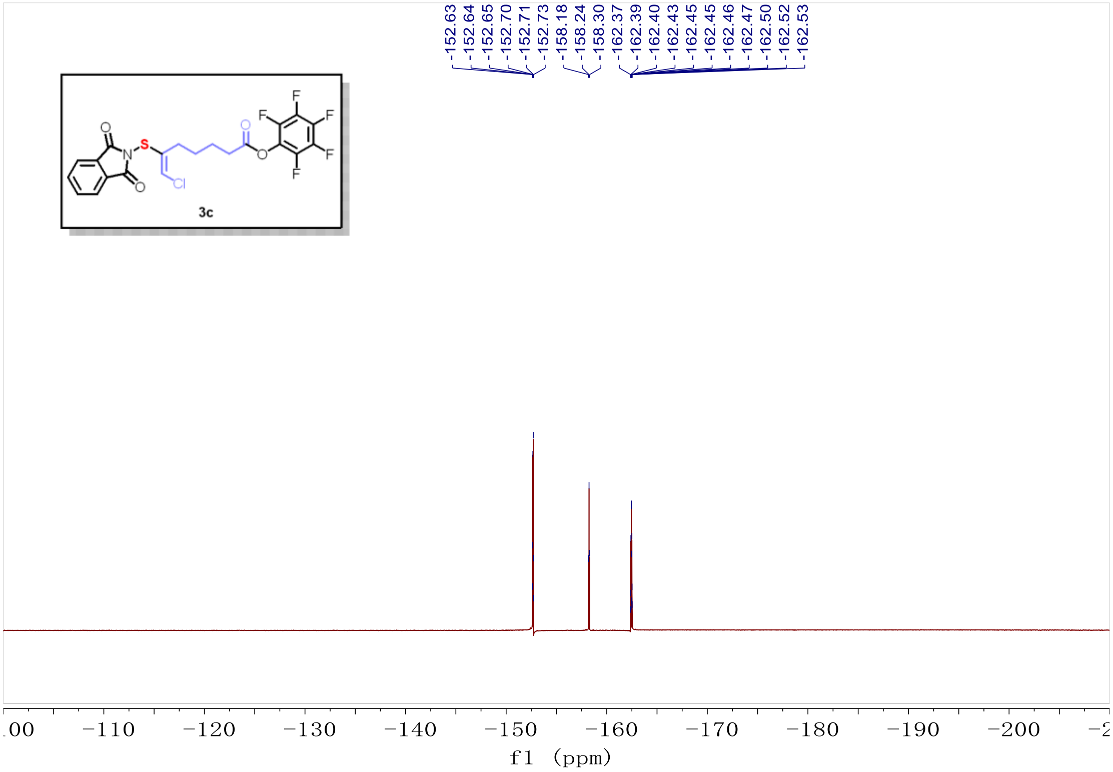

^{1}H NMR 400 MHz $CDCl_3$ of **3d**

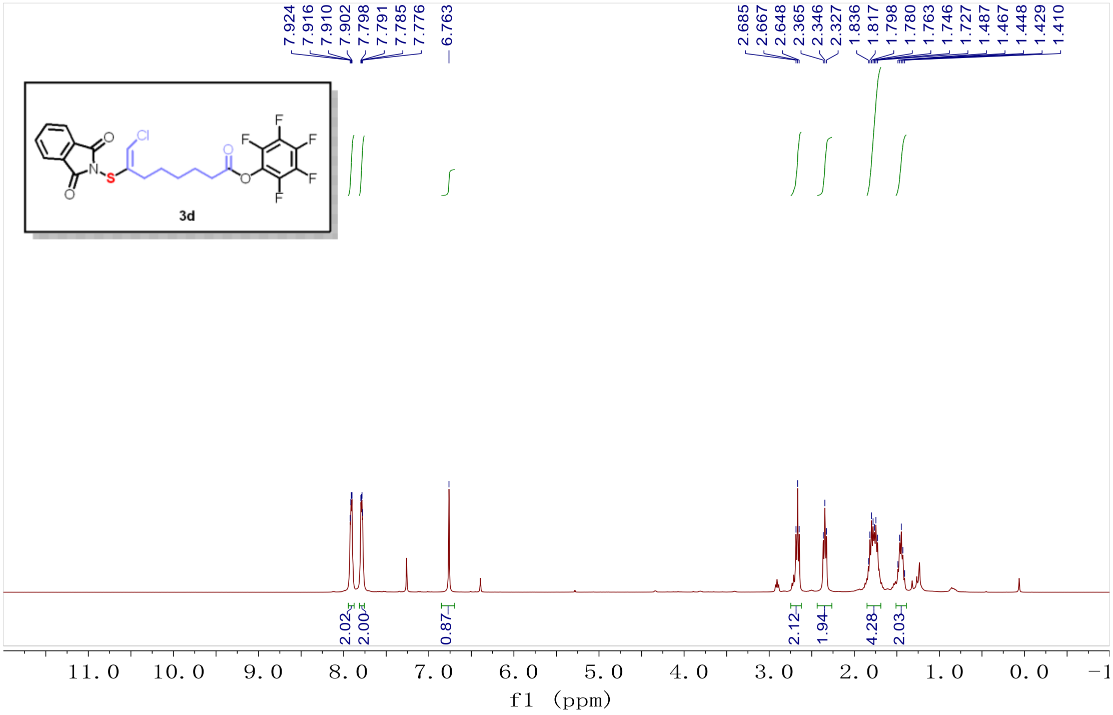

^{13}C NMR 100 MHz $CDCl_3$ of **3d**

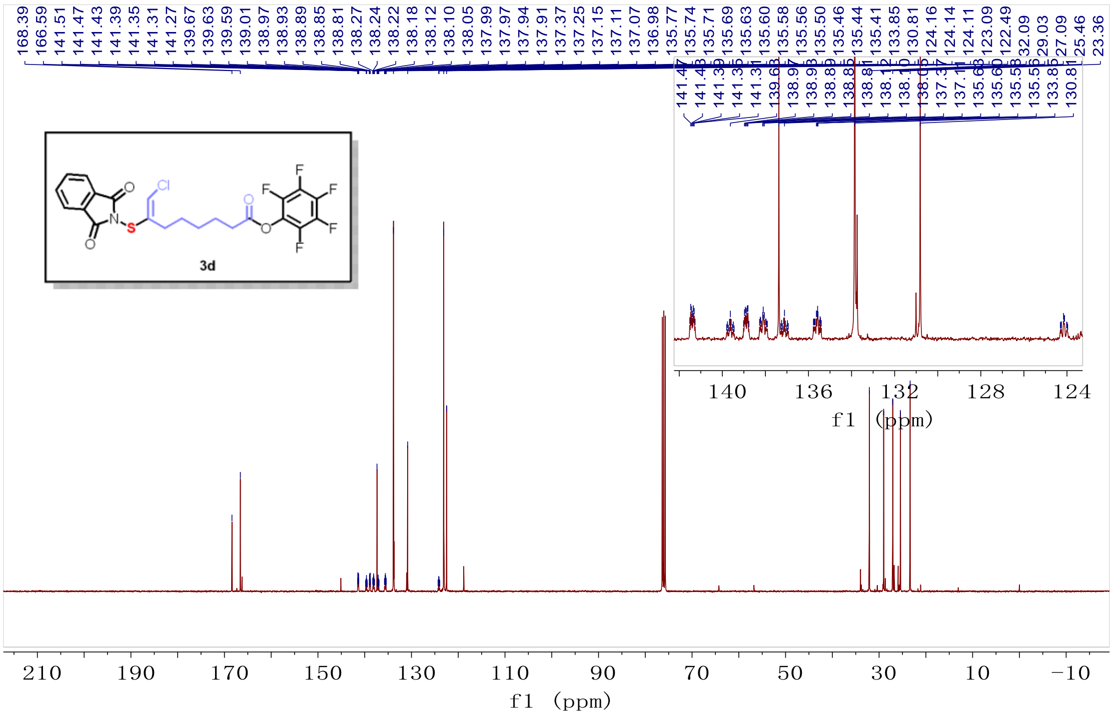

^{19}F NMR 376 MHz $CDCl_3$ of **3d**

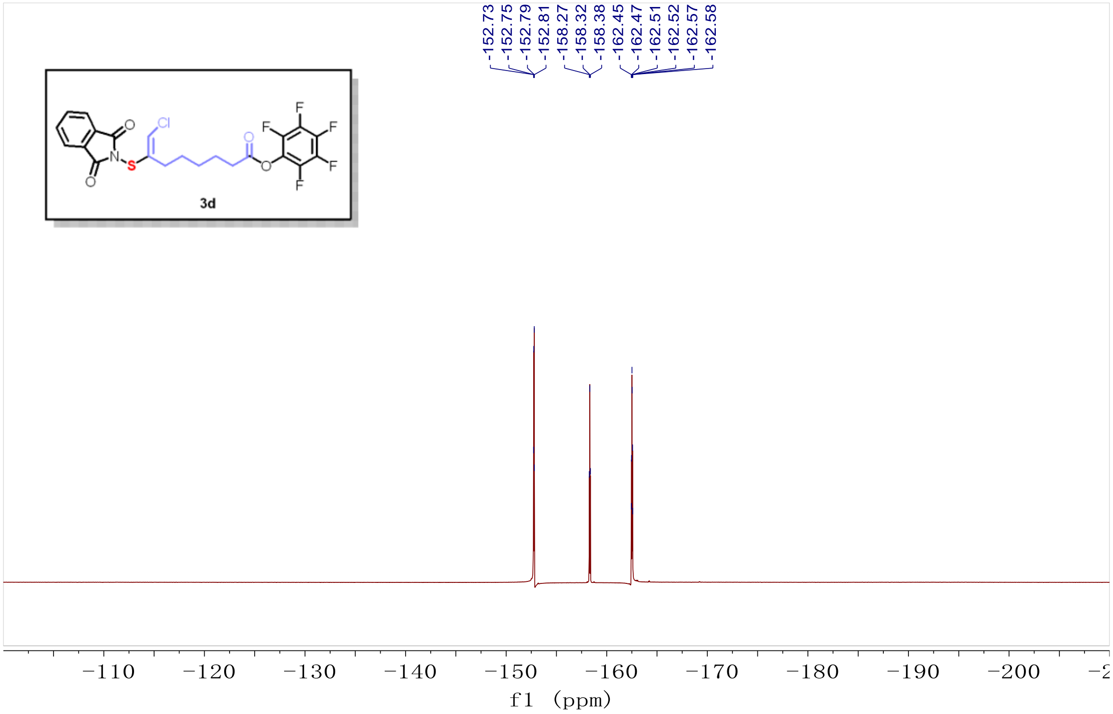

^{1}H NMR 400 MHz CDCl3 of **3e**

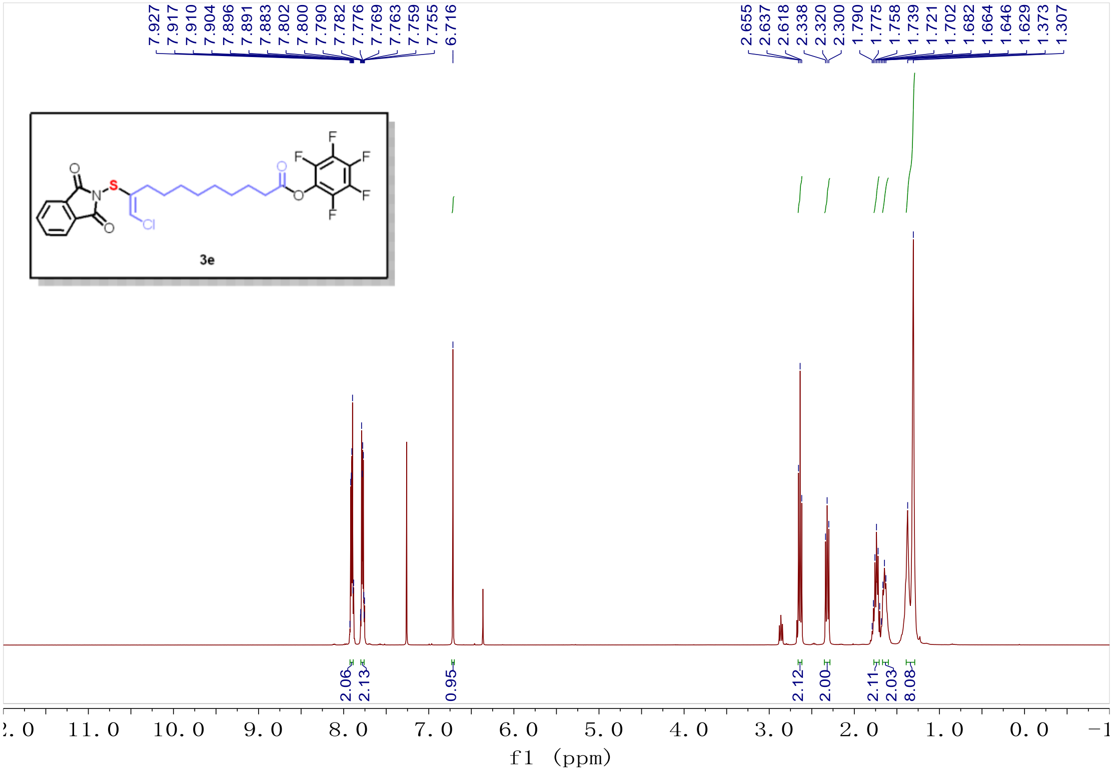

^{13}C NMR 125 MHz CDCl3 of **3e**

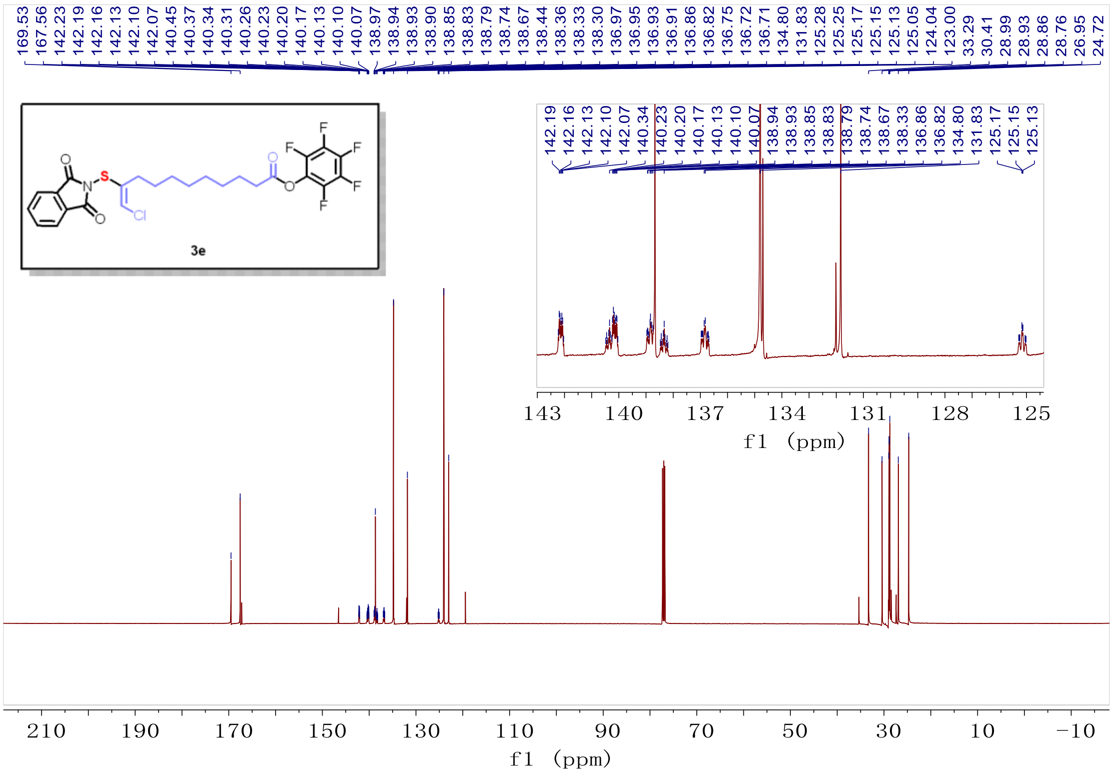

^{19}F NMR 376 MHz CDCl3 of **3e**

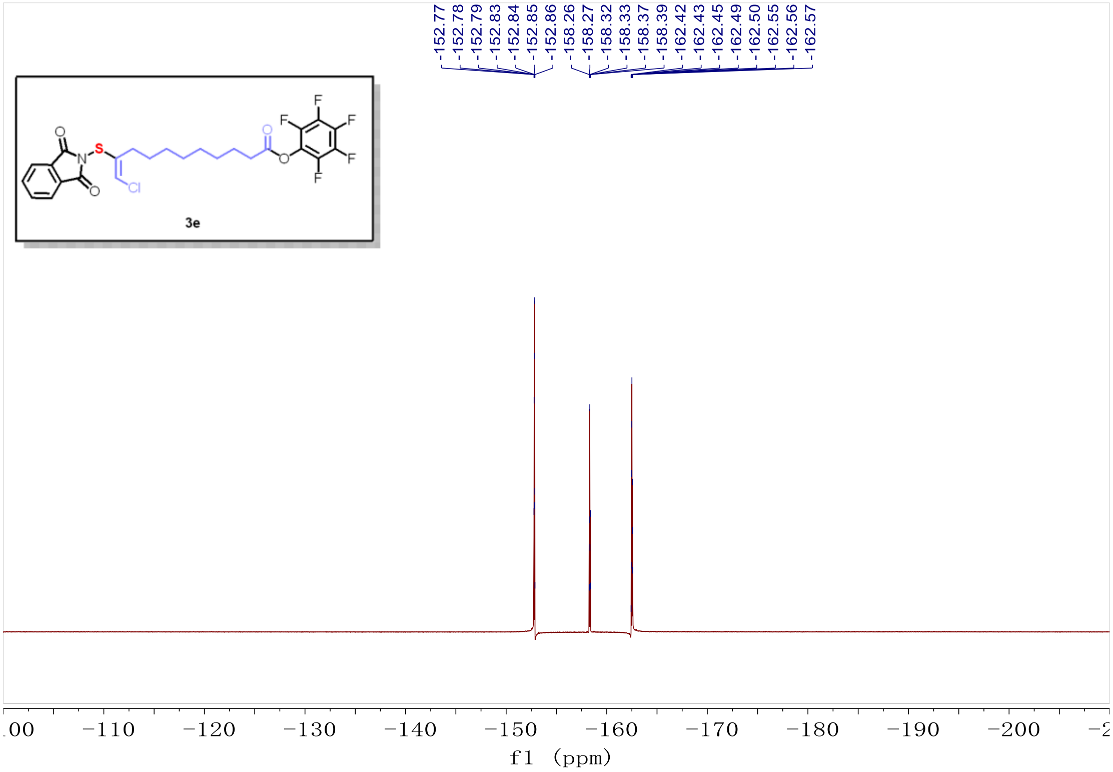

^{1}H NMR 500 MHz CDCl3 of **3f**

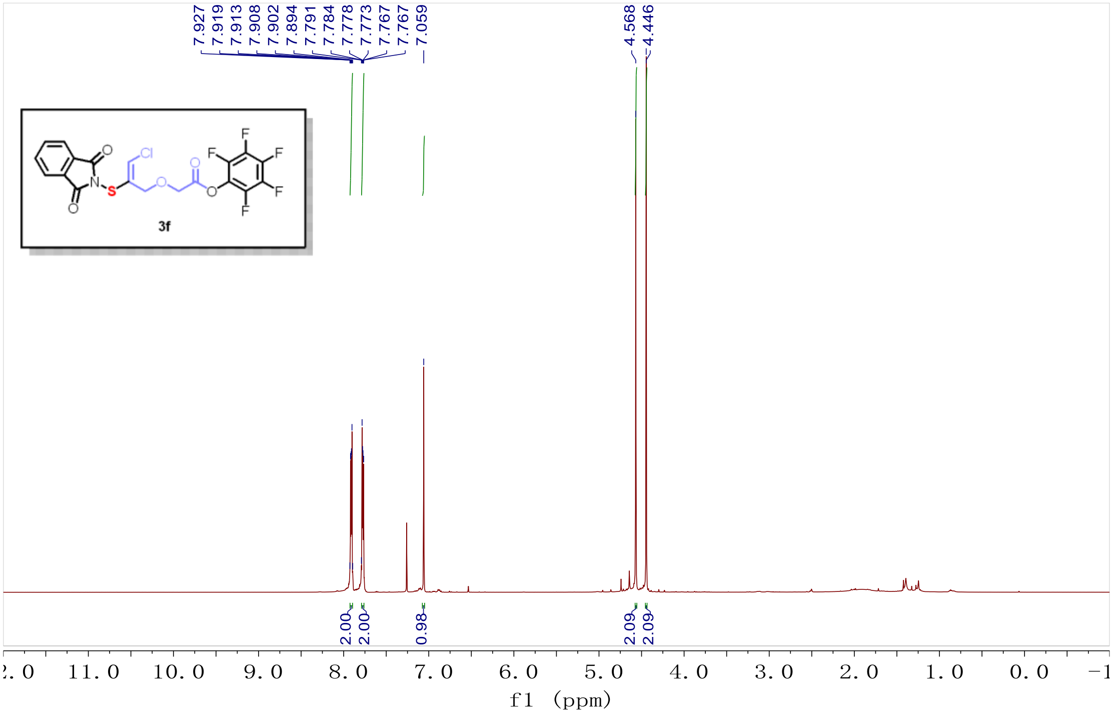

^{13}C NMR 125 MHz CDCl$_3$ of **3f**

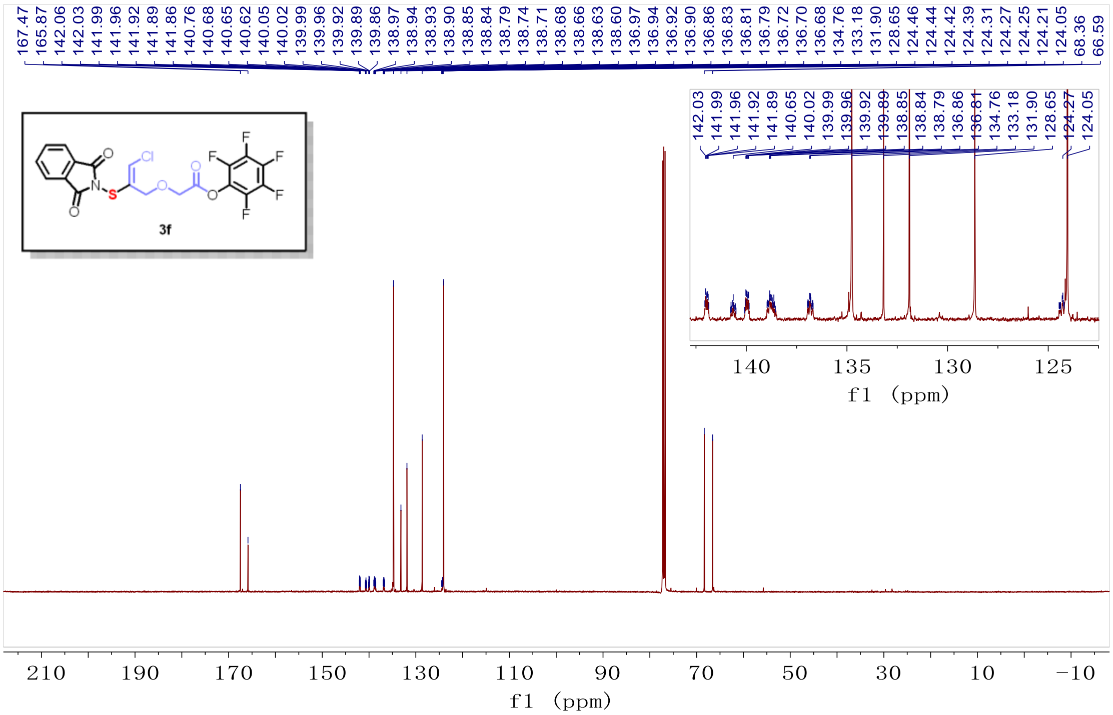

^{19}F NMR 470 MHz CDCl$_3$ of **3f**

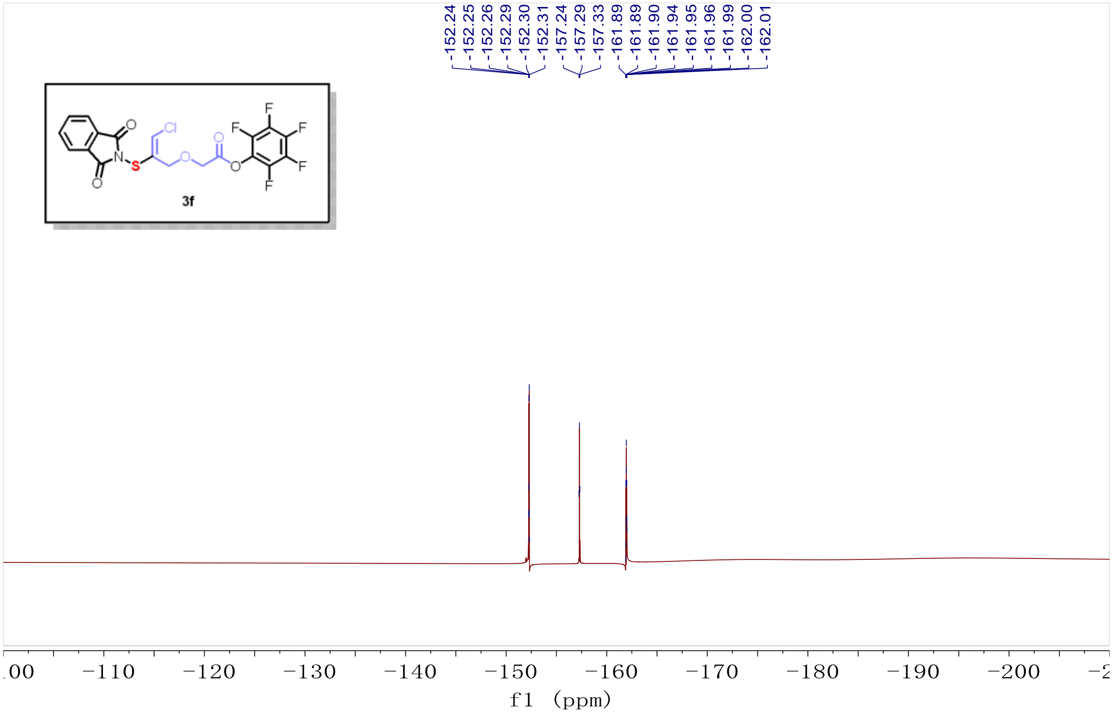

^{1}H NMR 400 MHz CD_3CN of **3g**

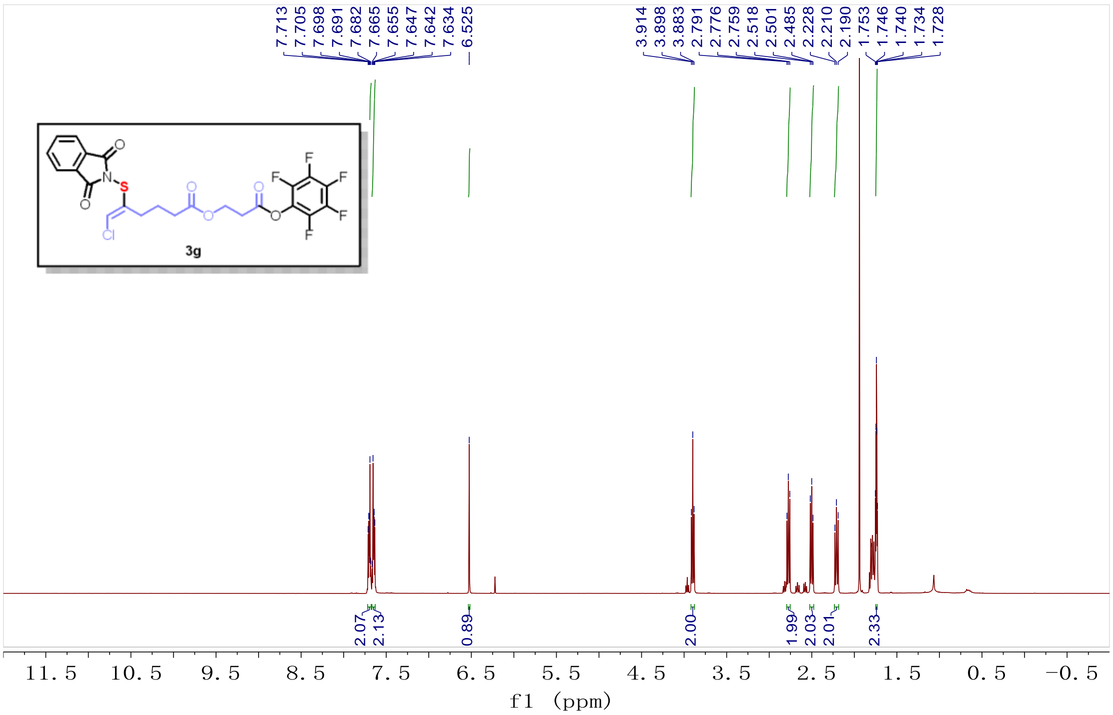

^{13}C NMR 125 MHz CD_3CN of **3g**

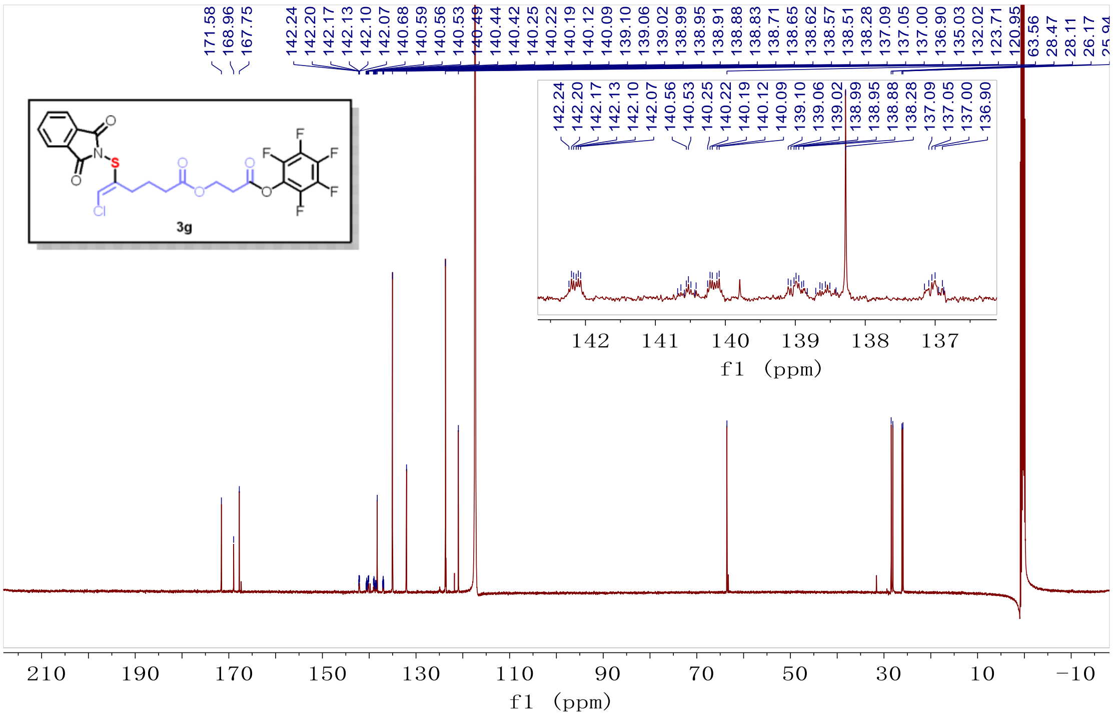

^{19}F NMR 376 MHz CDCl$_3$ of **3g**

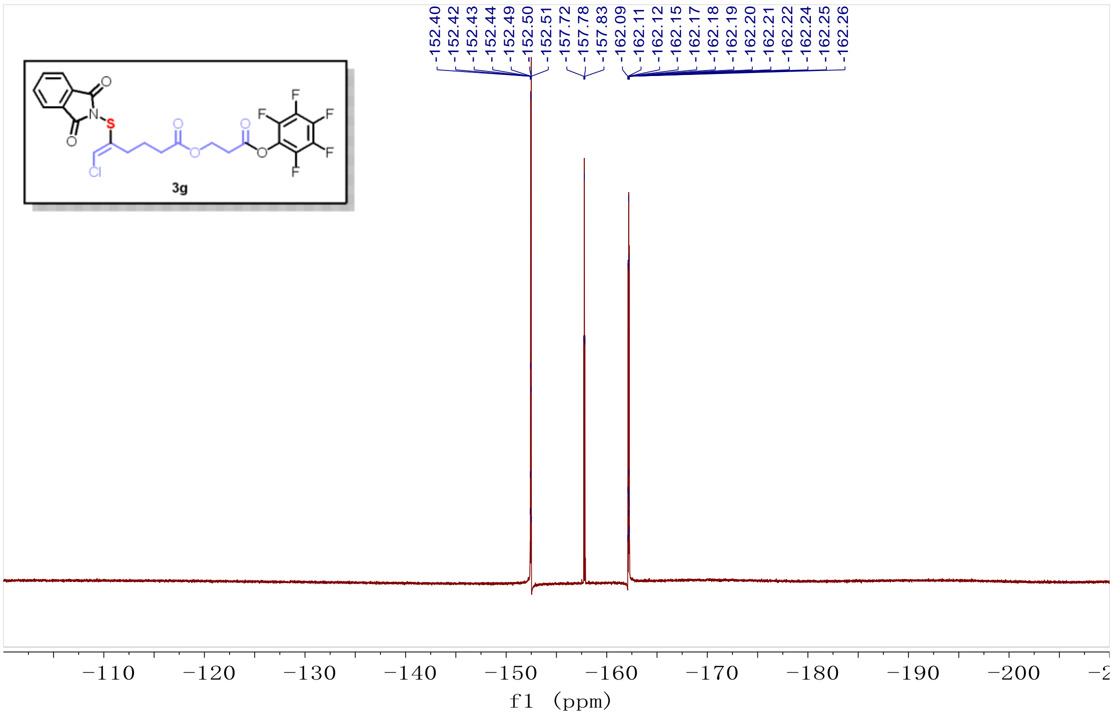

^{1}H NMR 400 MHz CDCl$_3$ of **3h**

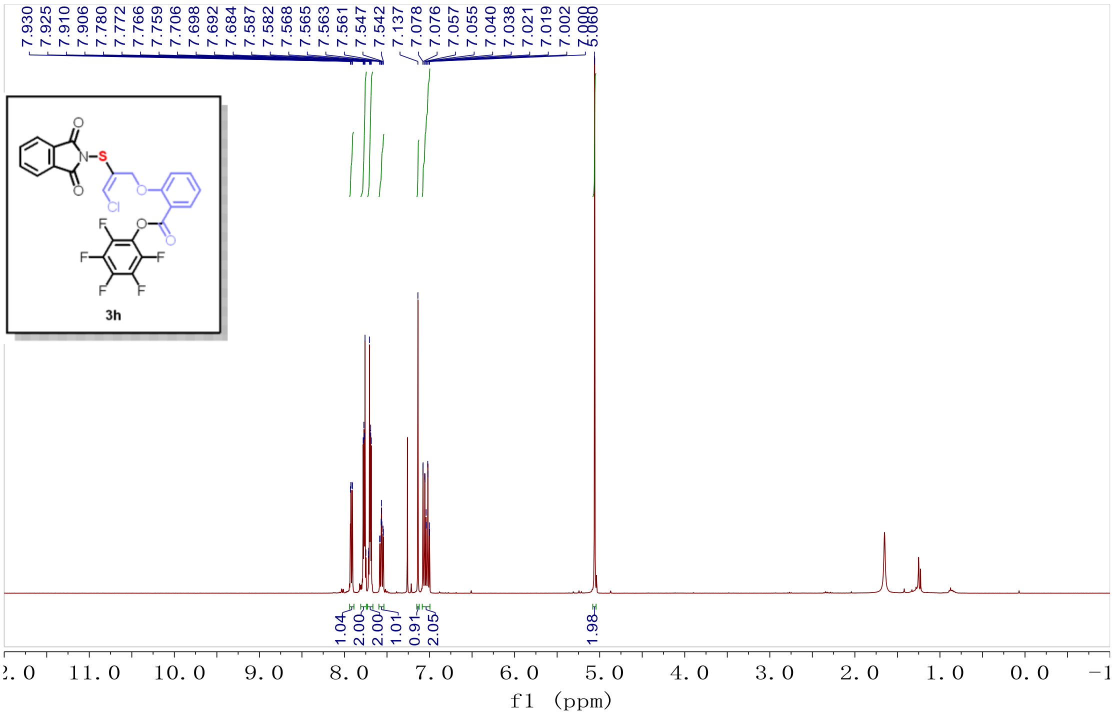

^{13}C NMR 100 MHz CDCl$_3$ of **3h**

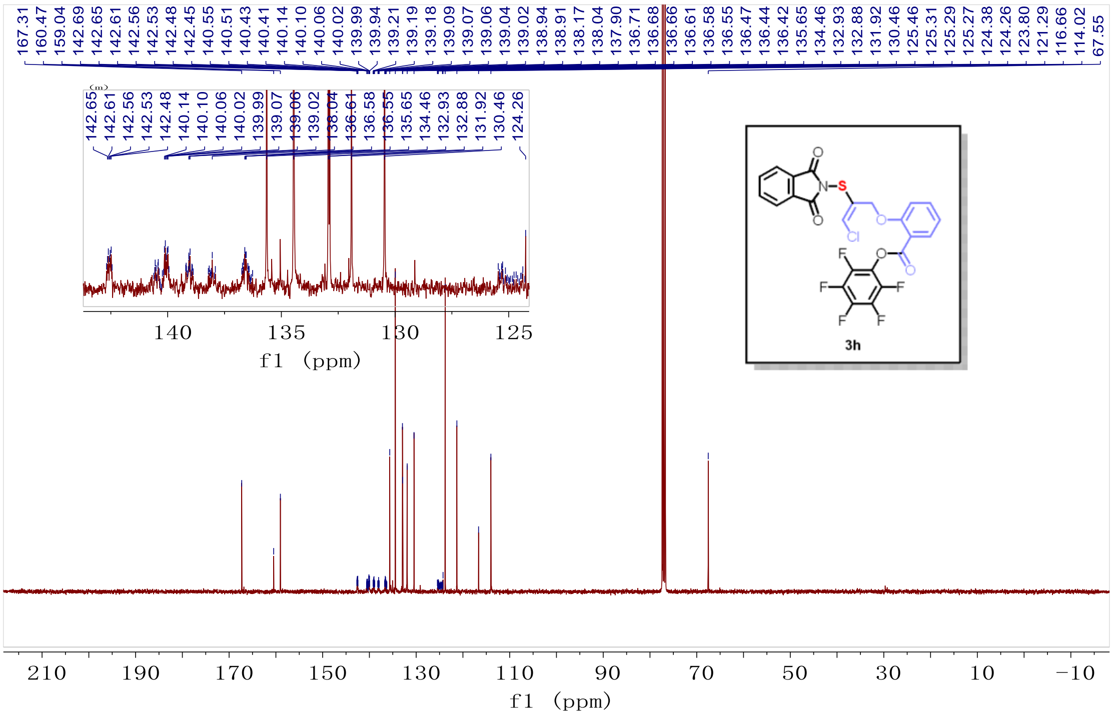

^{19}F NMR 376 MHz CDCl$_3$ of **3h**

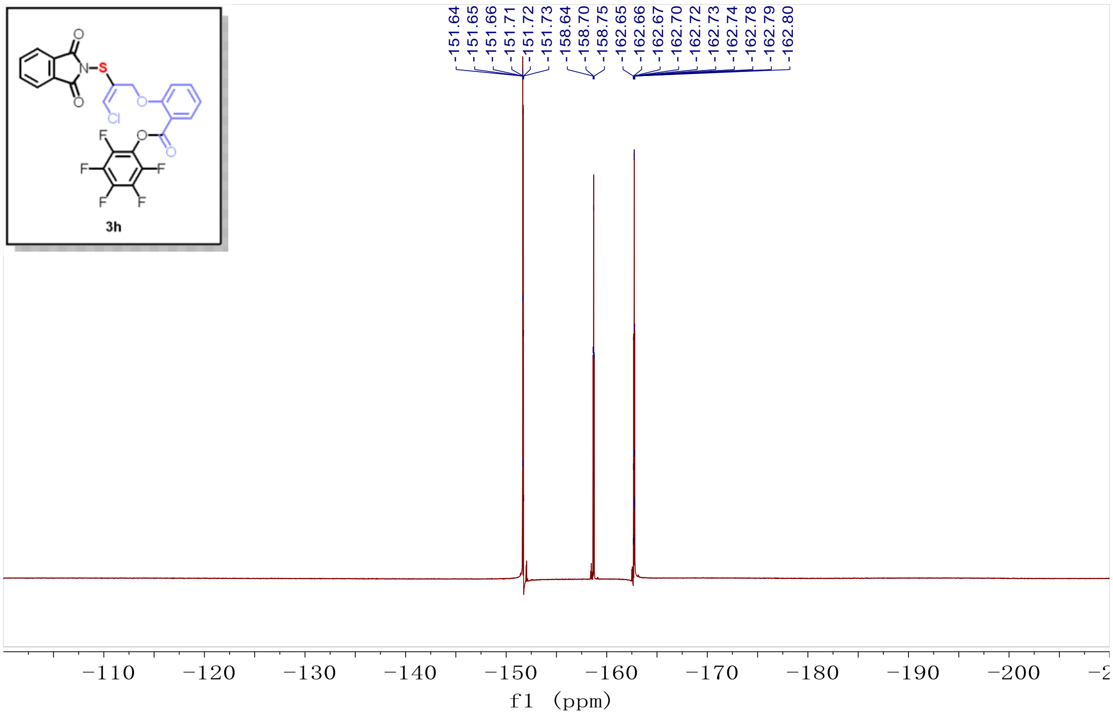

^{1}H NMR 400 MHz $CDCl_3$ of **3i**

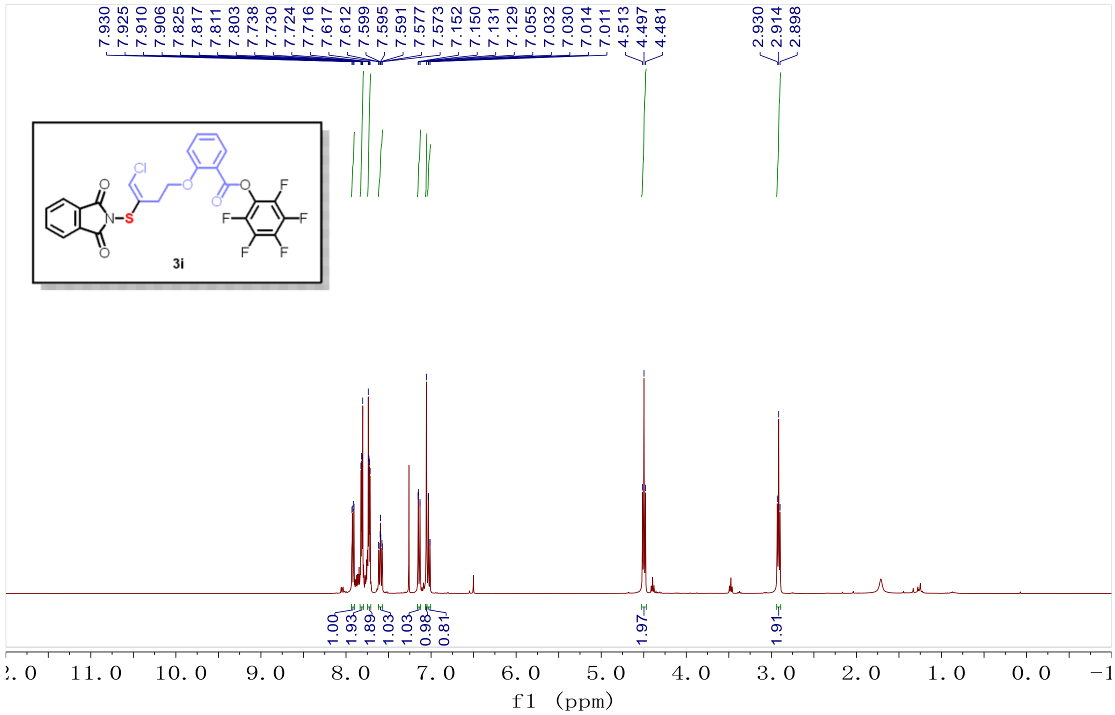

^{13}C NMR 125 MHz $CDCl_3$ of **3i**

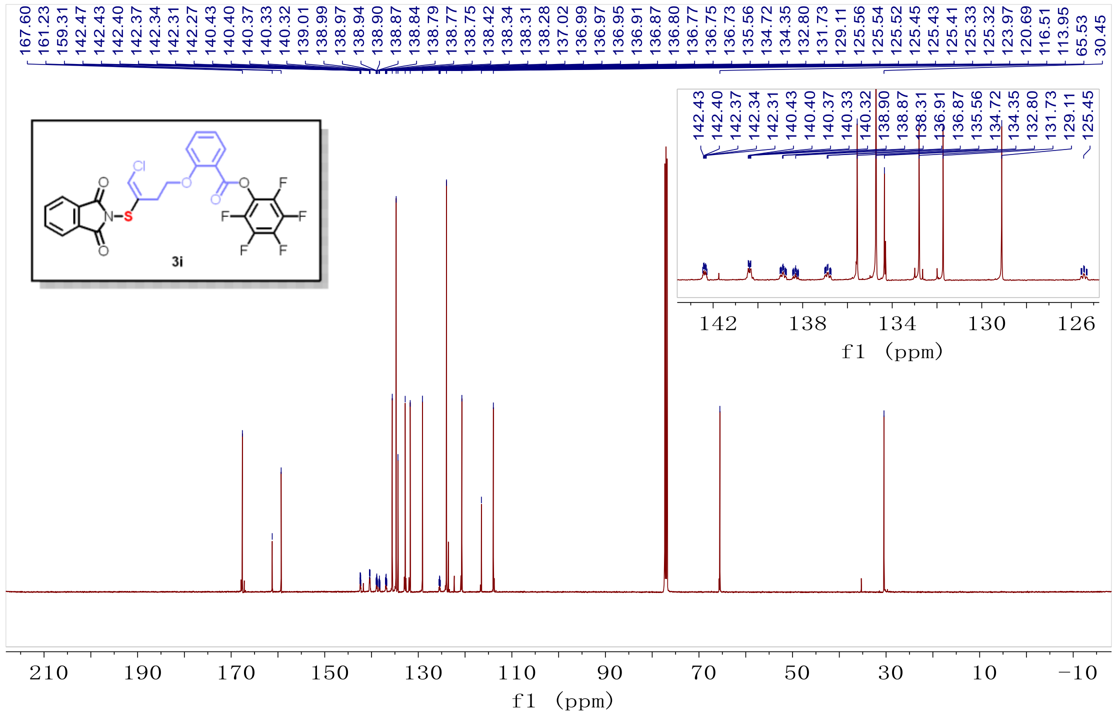

^{19}F NMR 376 MHz $CDCl_3$ of **3i**

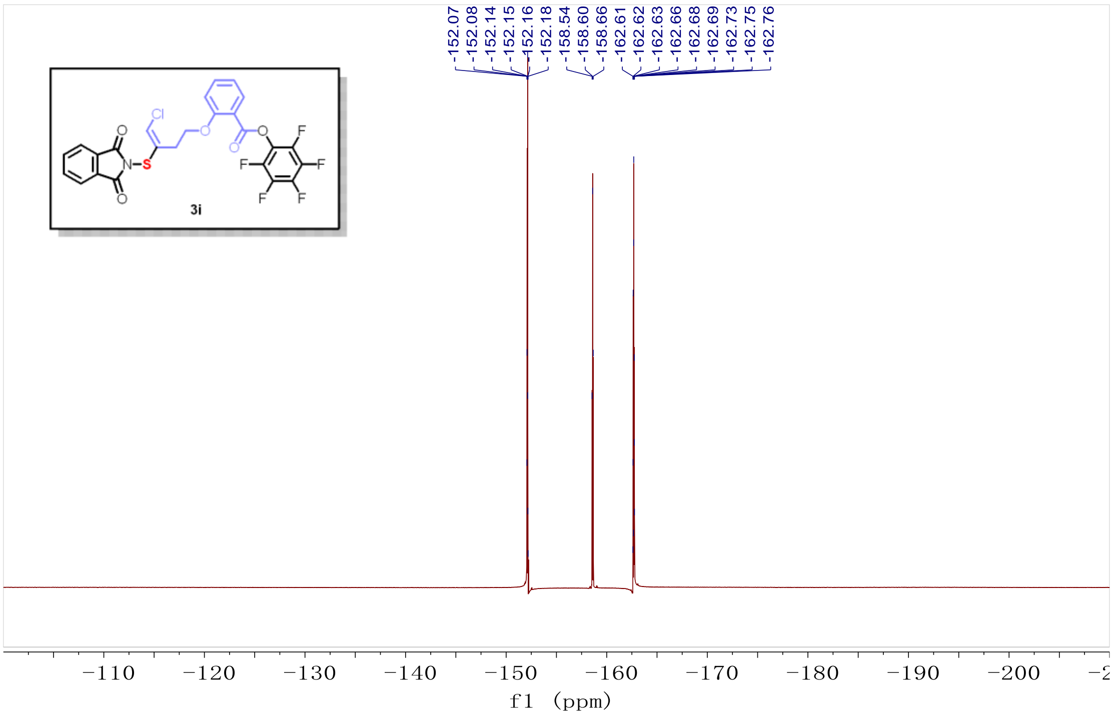

^{1}H NMR 300 MHz $CDCl_3$ of **3j**

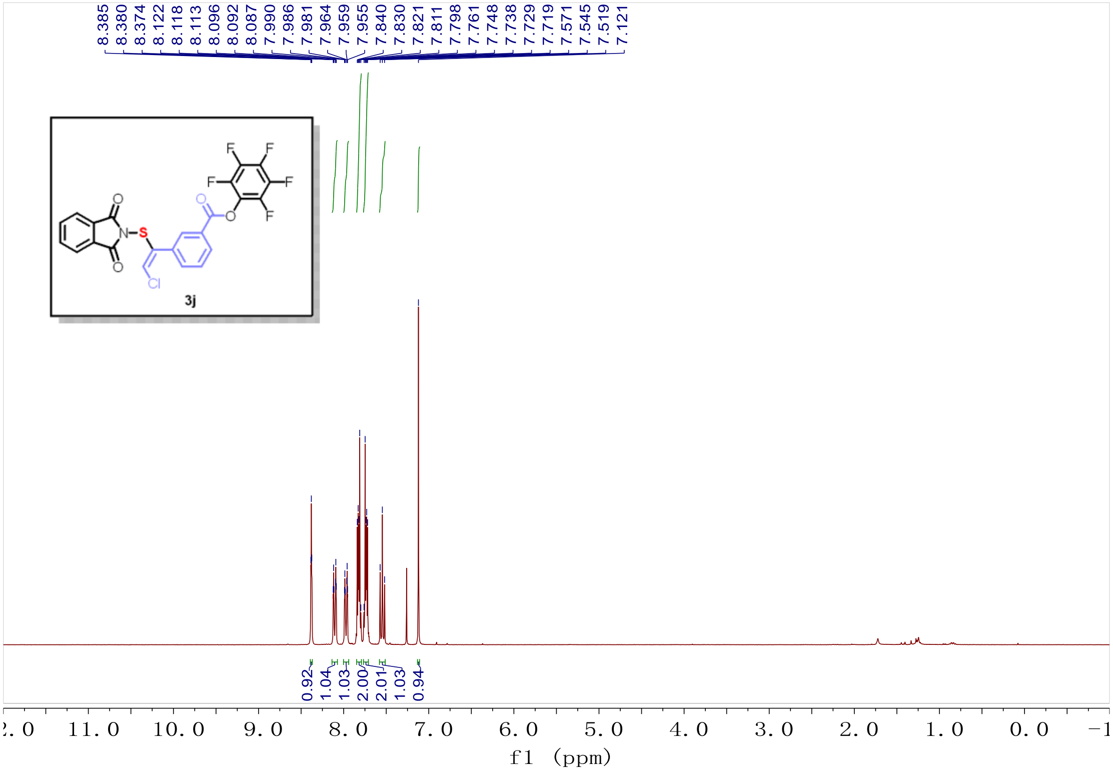

^{13}C NMR 125 MHz $CDCl_3$ of **3j**

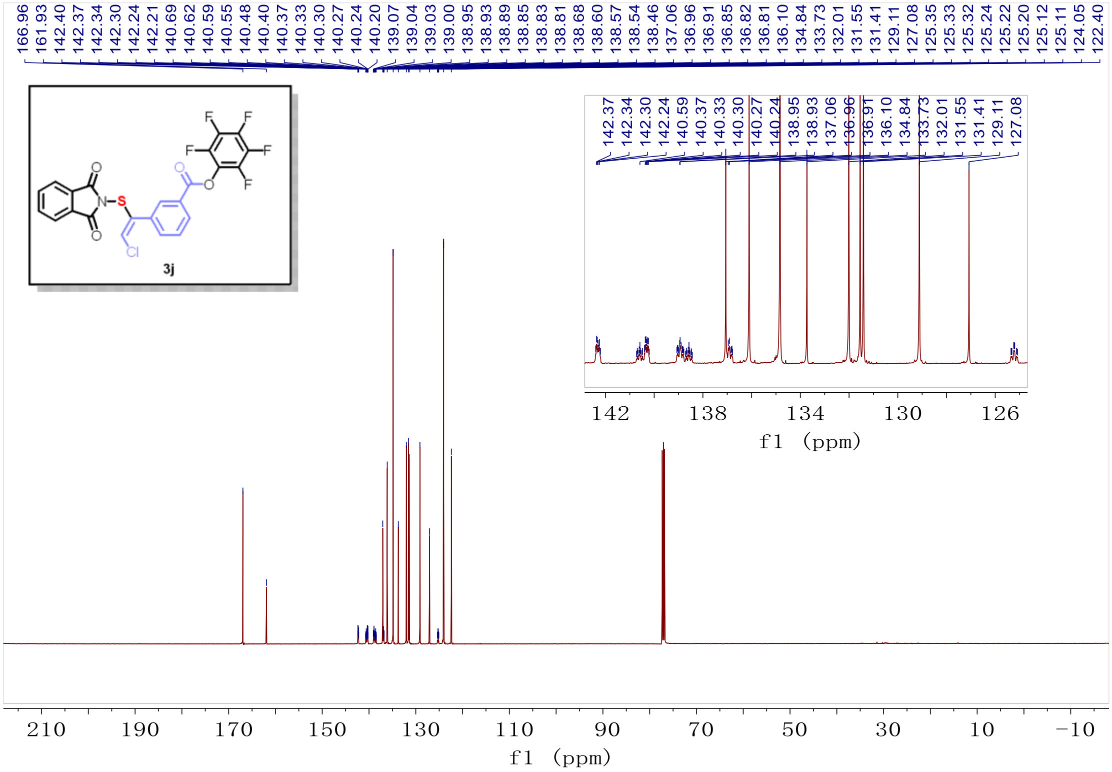

^{19}F NMR 282 MHz $CDCl_3$ of **3j**

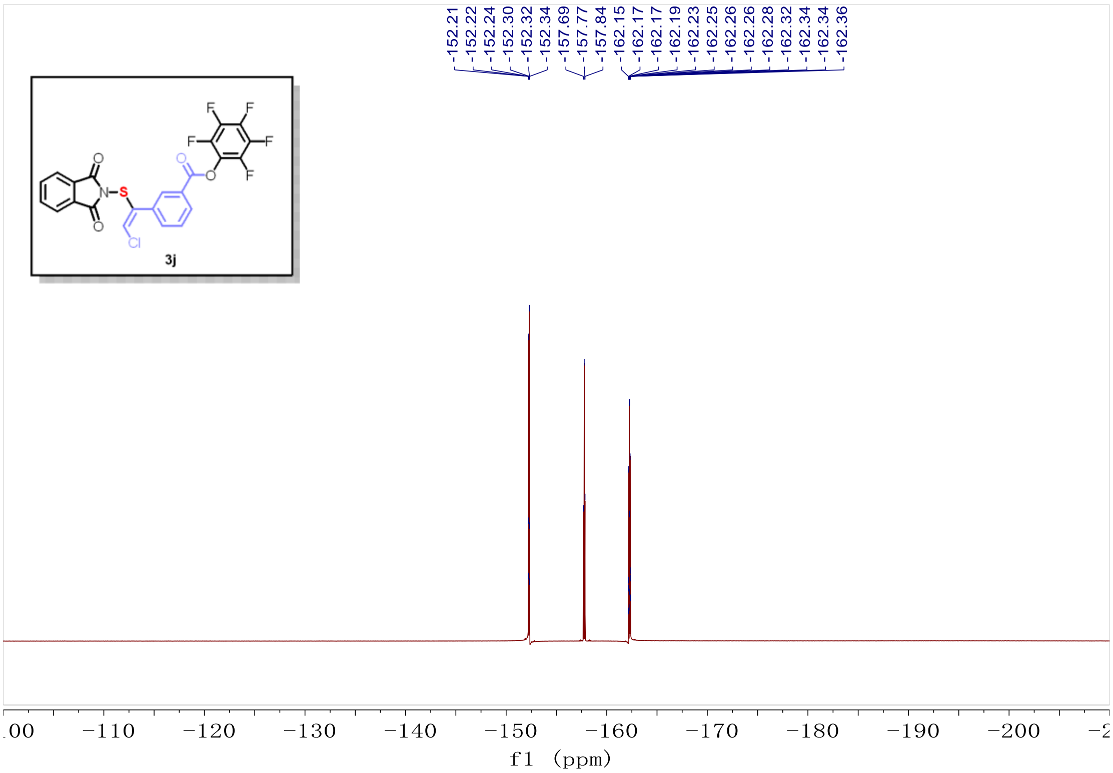

^{1}H NMR 400 MHz $CDCl_3$ of **3k**

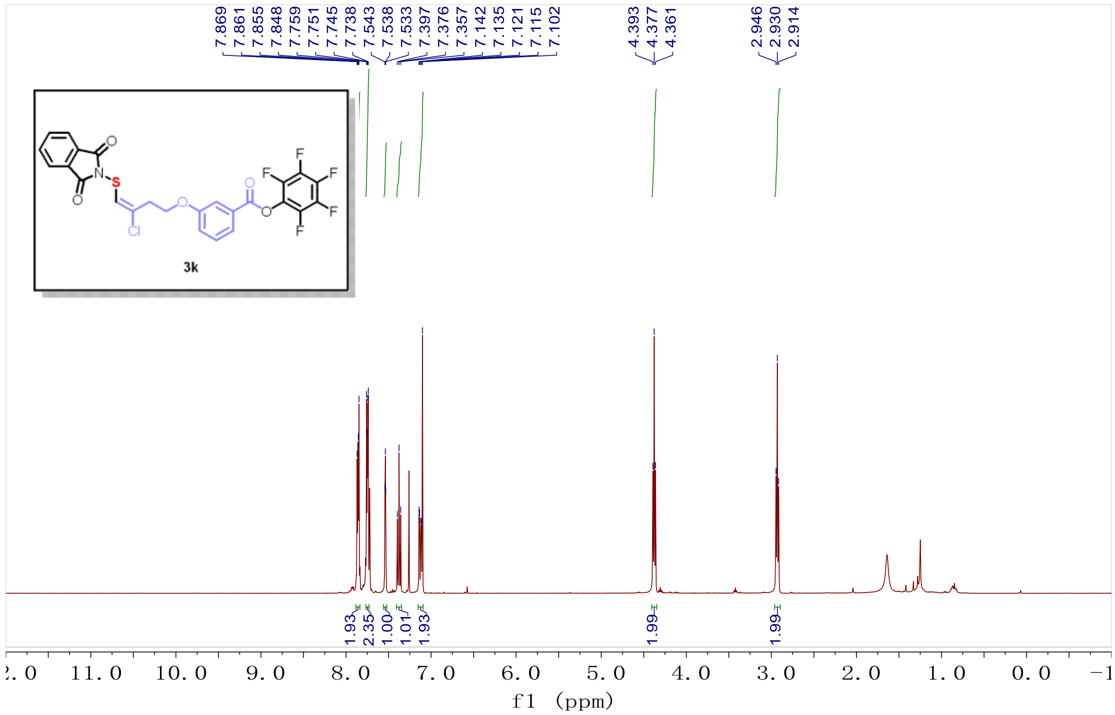

^{13}C NMR 100 MHz $CDCl_3$ of **3k**

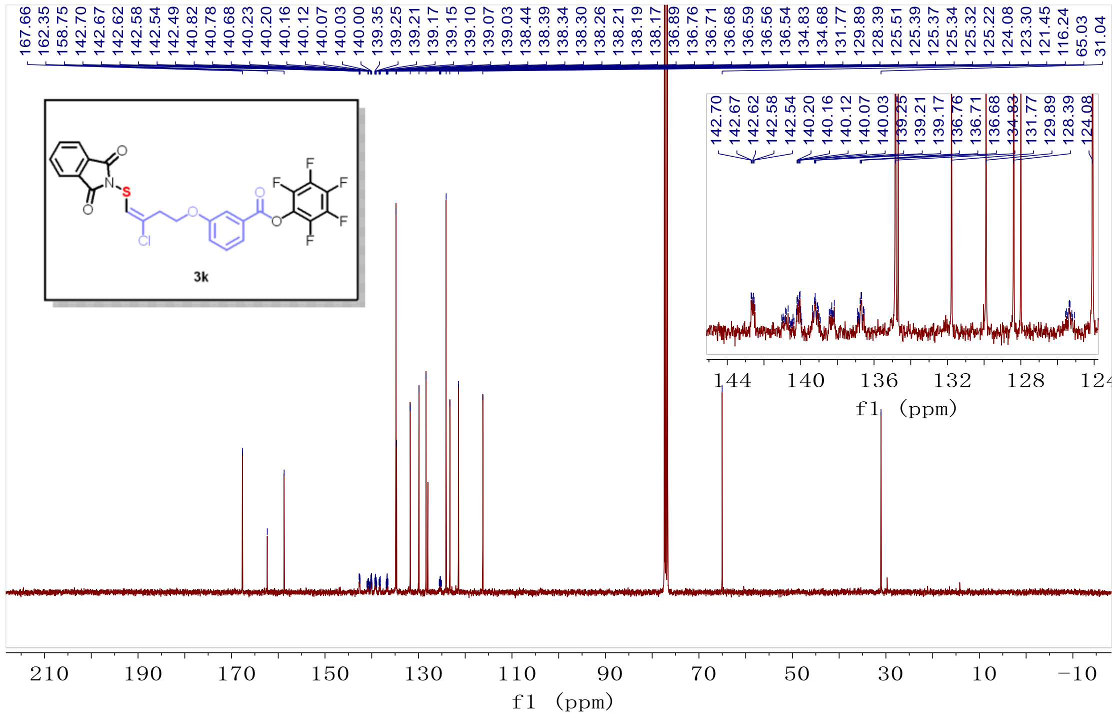

^{19}F NMR 376 MHz CDCl3 of **3k**

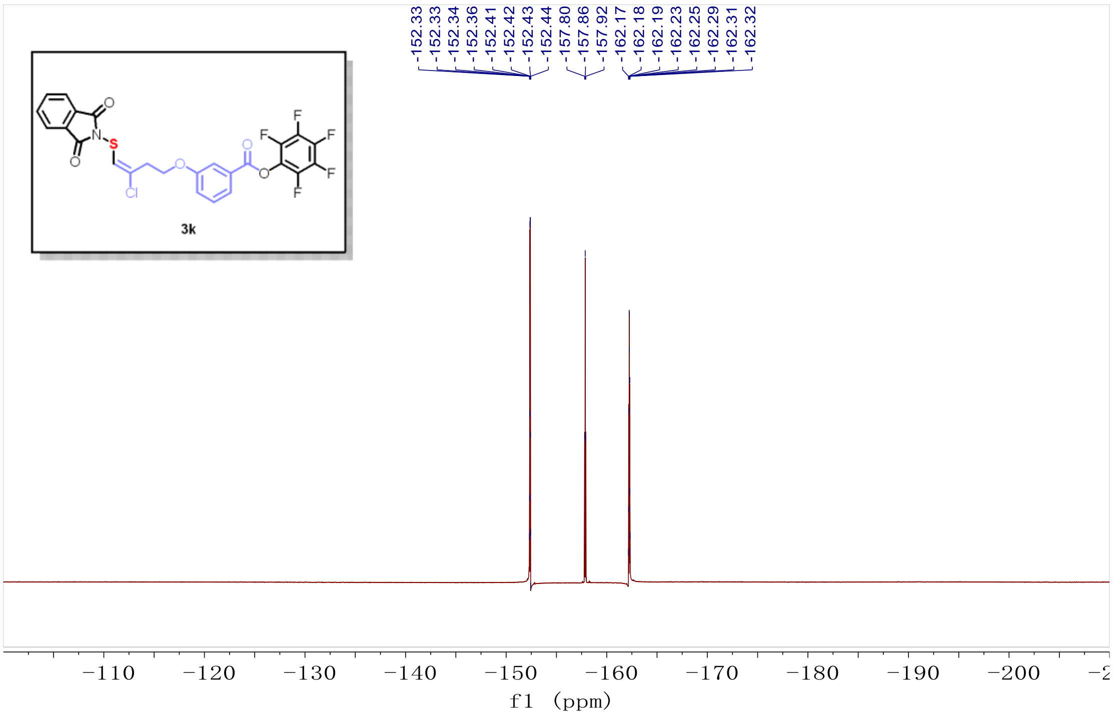

^{1}H NMR 400 MHz CDCl3 of **3l**

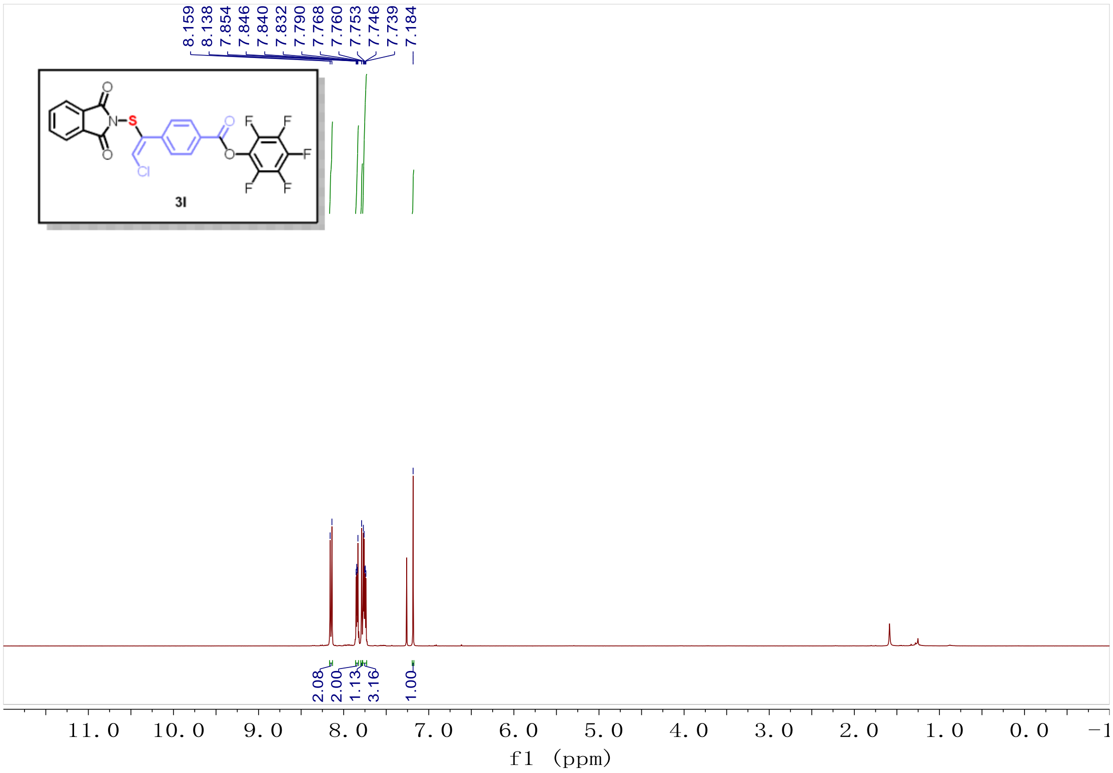

^{13}C NMR 125 MHz $CDCl_3$ of **3l**

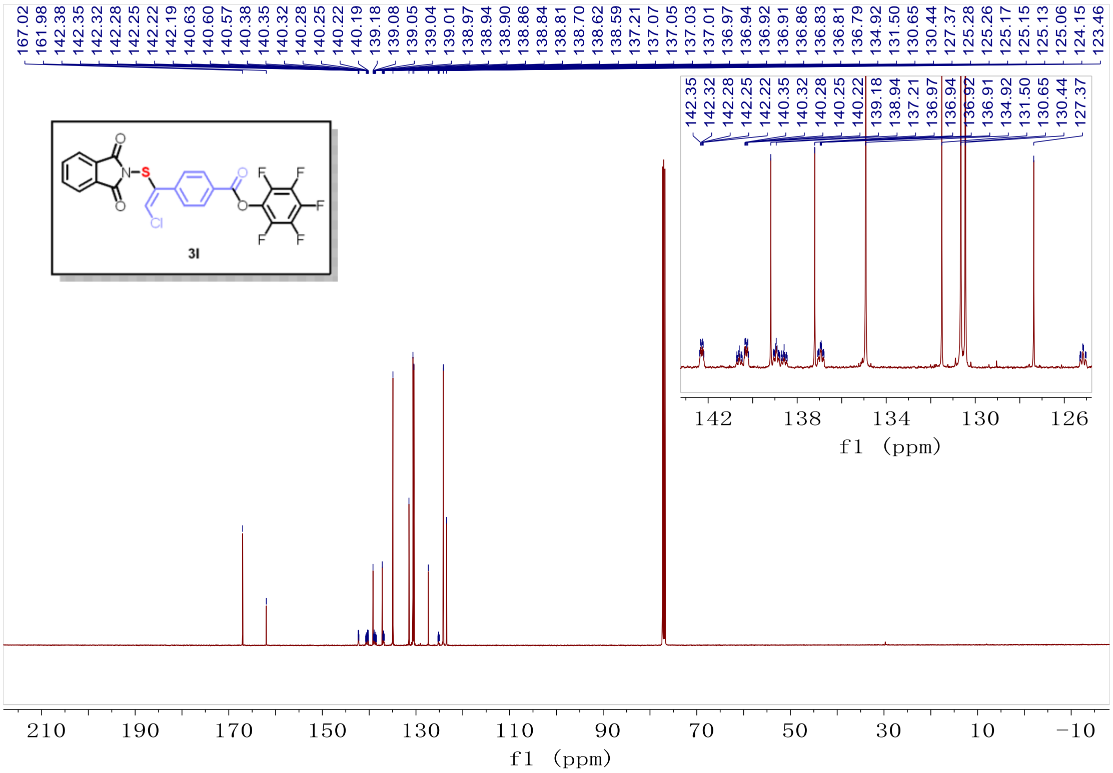

^{19}F NMR 376 MHz $CDCl_3$ of **3l**

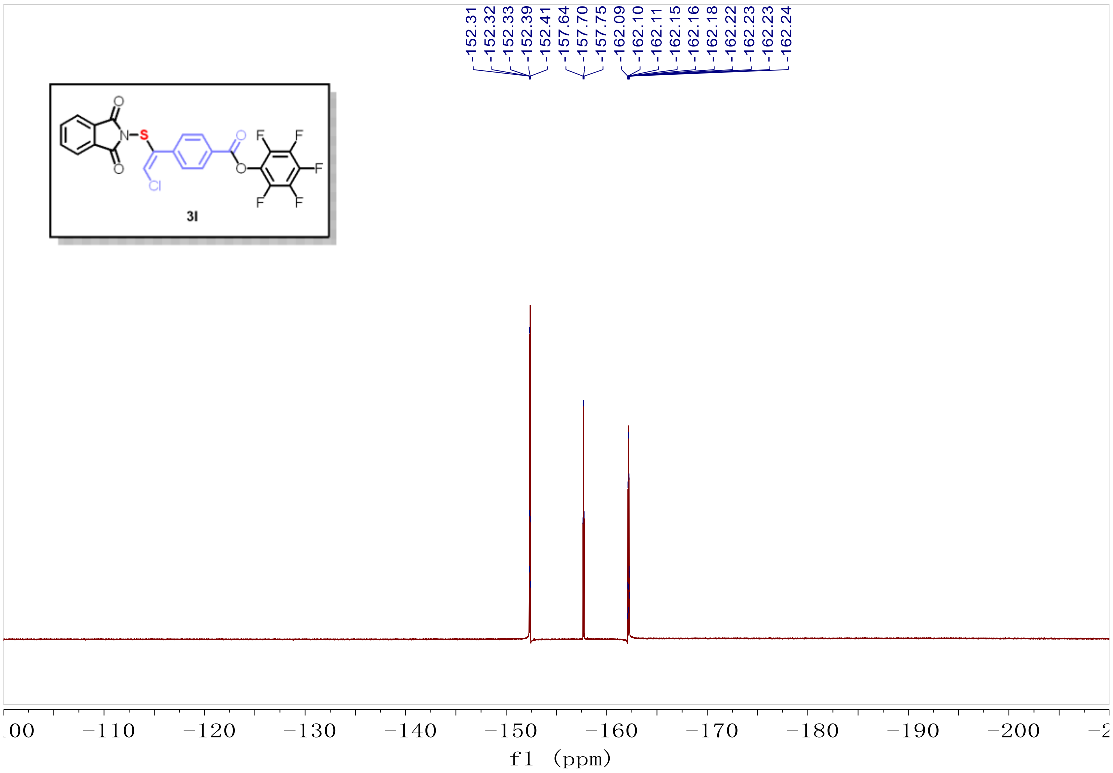

^{1}H NMR 400 MHz $CDCl_3$ of **3m**

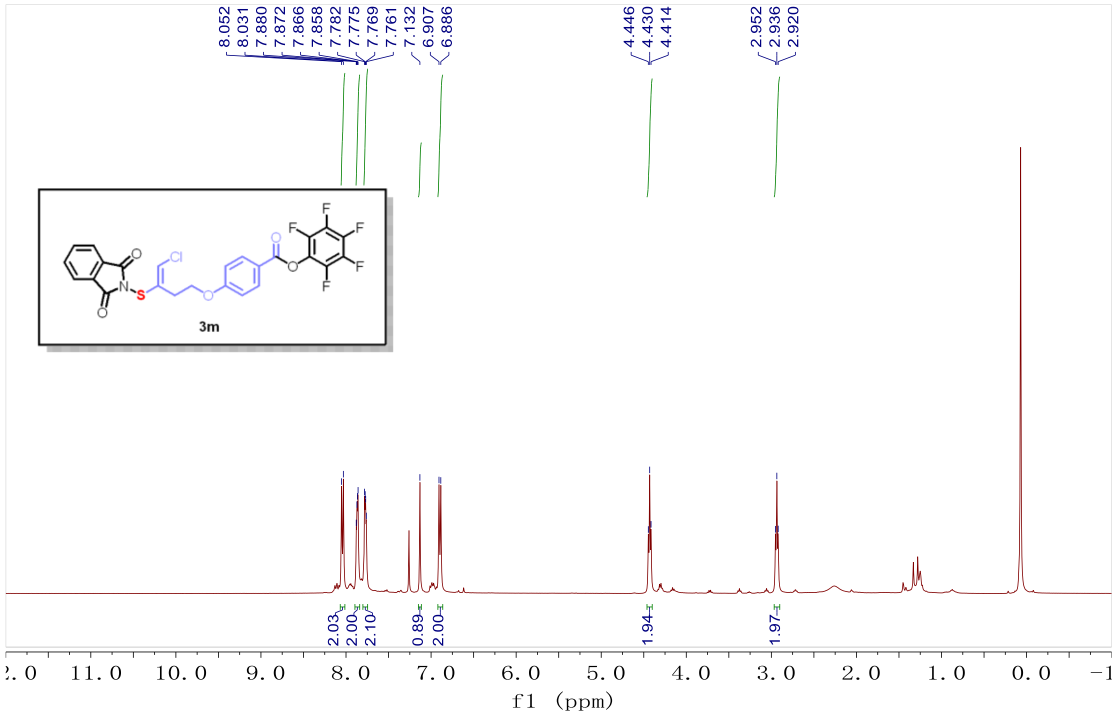

^{13}C NMR 100 MHz $CDCl_3$ of **3m**

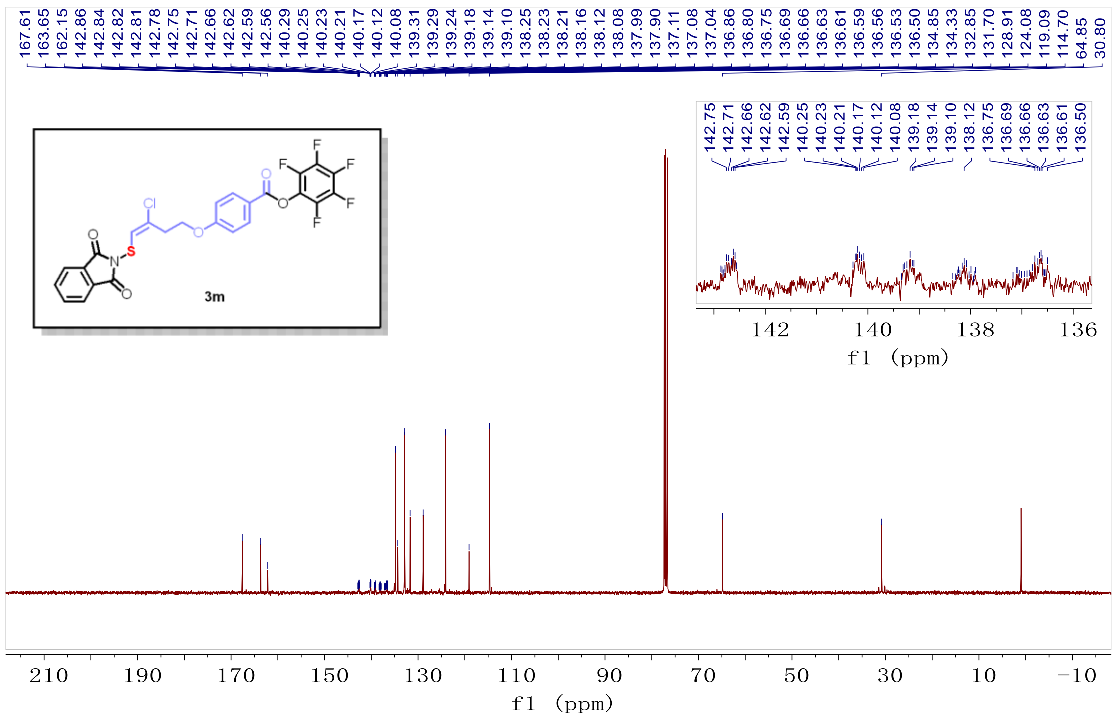

^{19}F NMR 376 MHz CDCl3 of **3m**

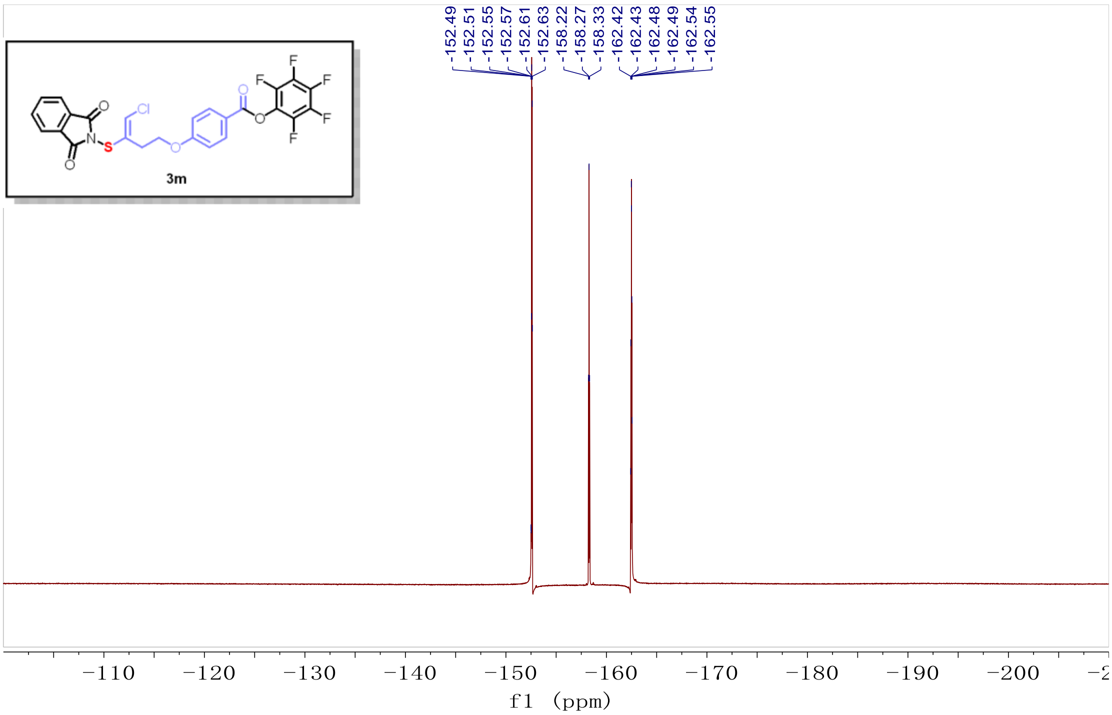

^{1}H NMR 400 MHz CDCl3 of **3n**

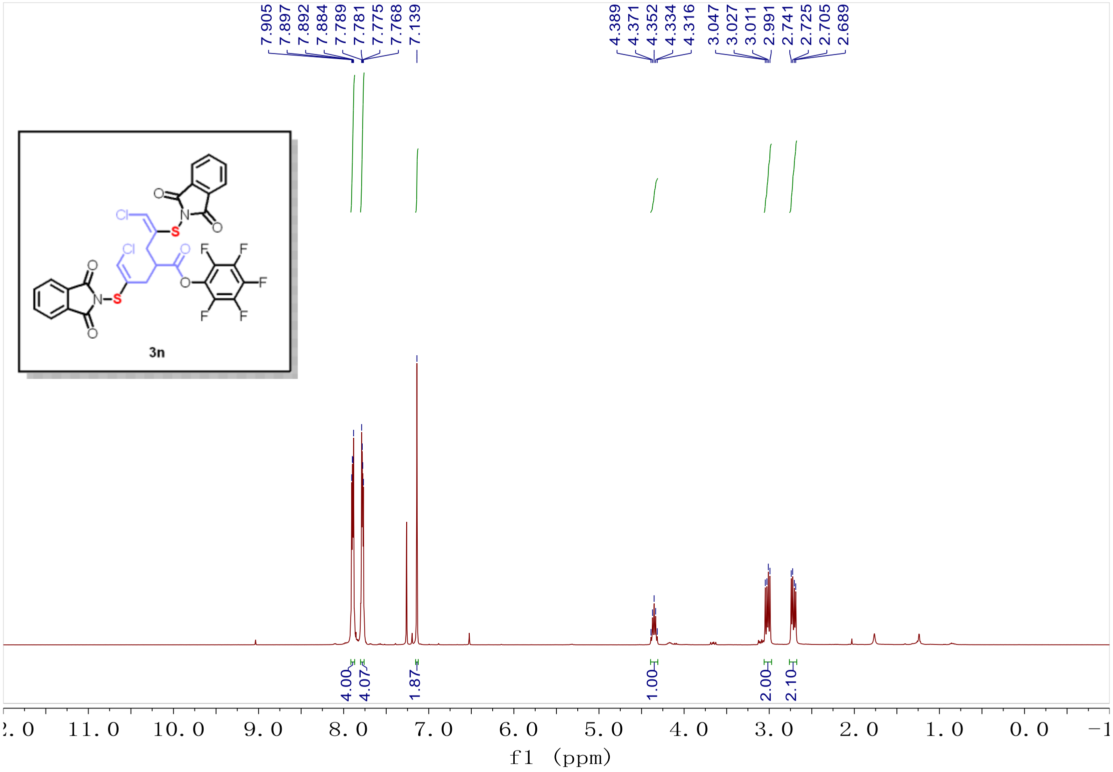

^{13}C NMR 125 MHz $CDCl_3$ of **3n**

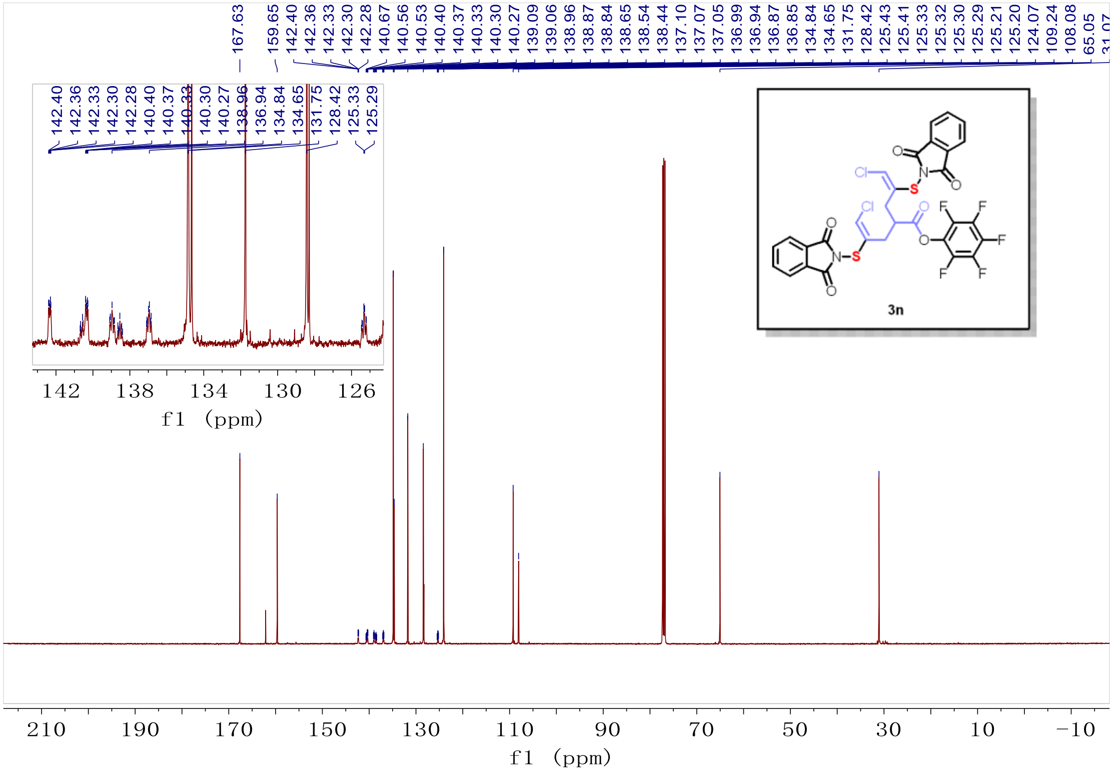

^{19}F NMR 376 MHz $CDCl_3$ of **3n**

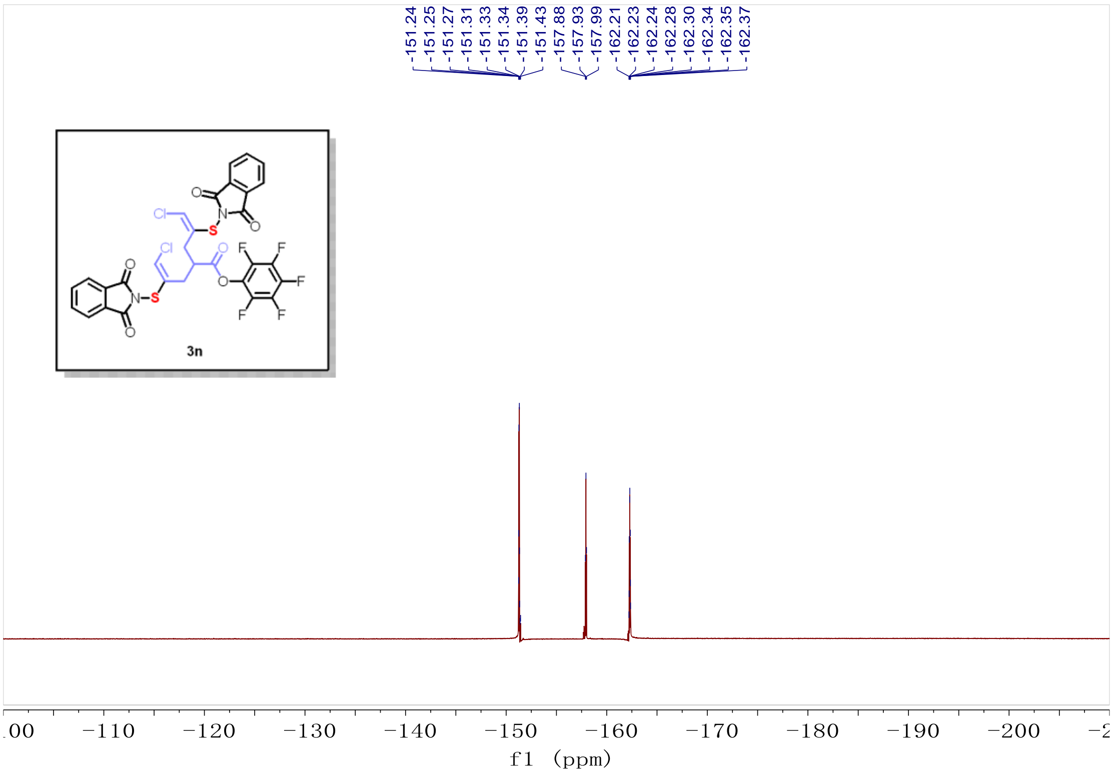

^{1}H NMR 500 MHz $CDCl_3$ of **3o**

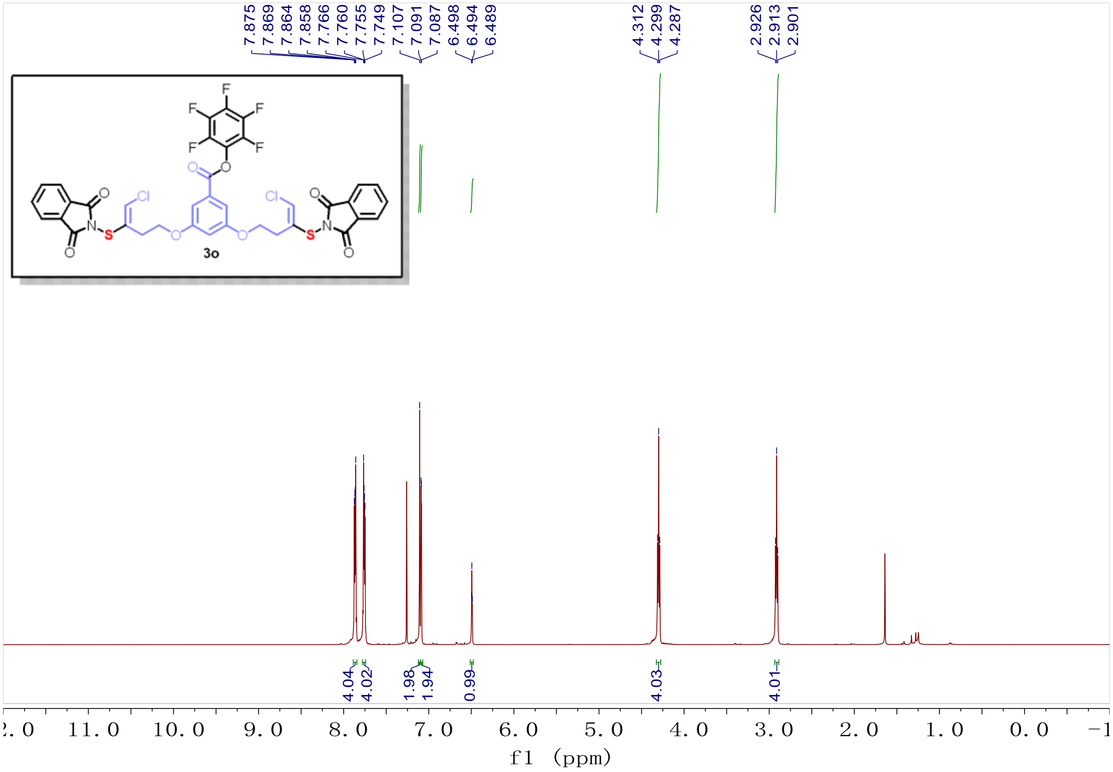

^{13}C NMR 125 MHz $CDCl_3$ of **3o**

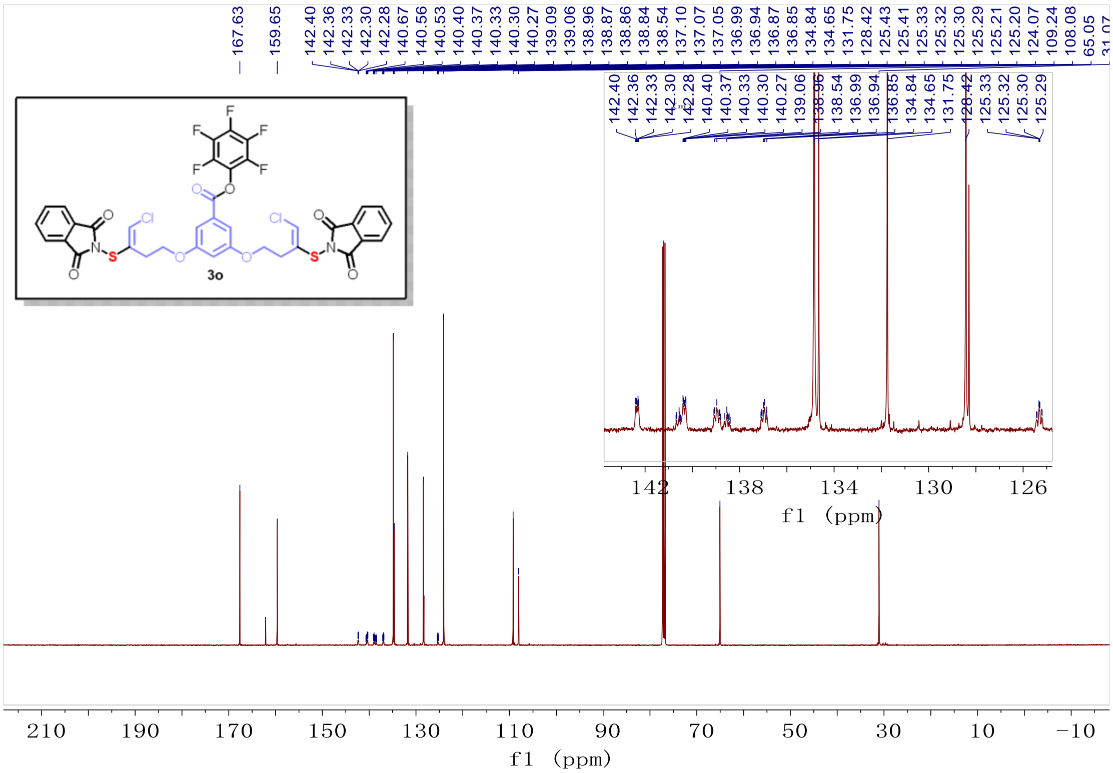

^{19}F NMR 282 MHz CDCl$_3$ of **3o**

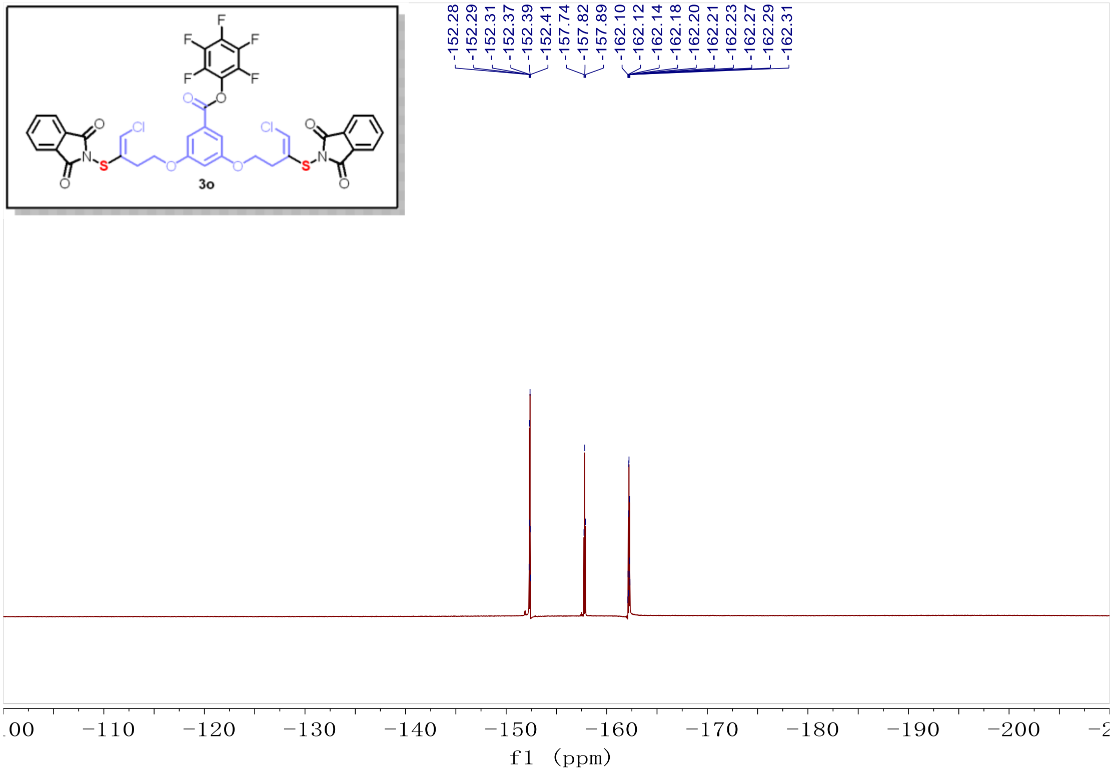

^{1}H NMR 400 MHz CDCl$_3$ of **3p**

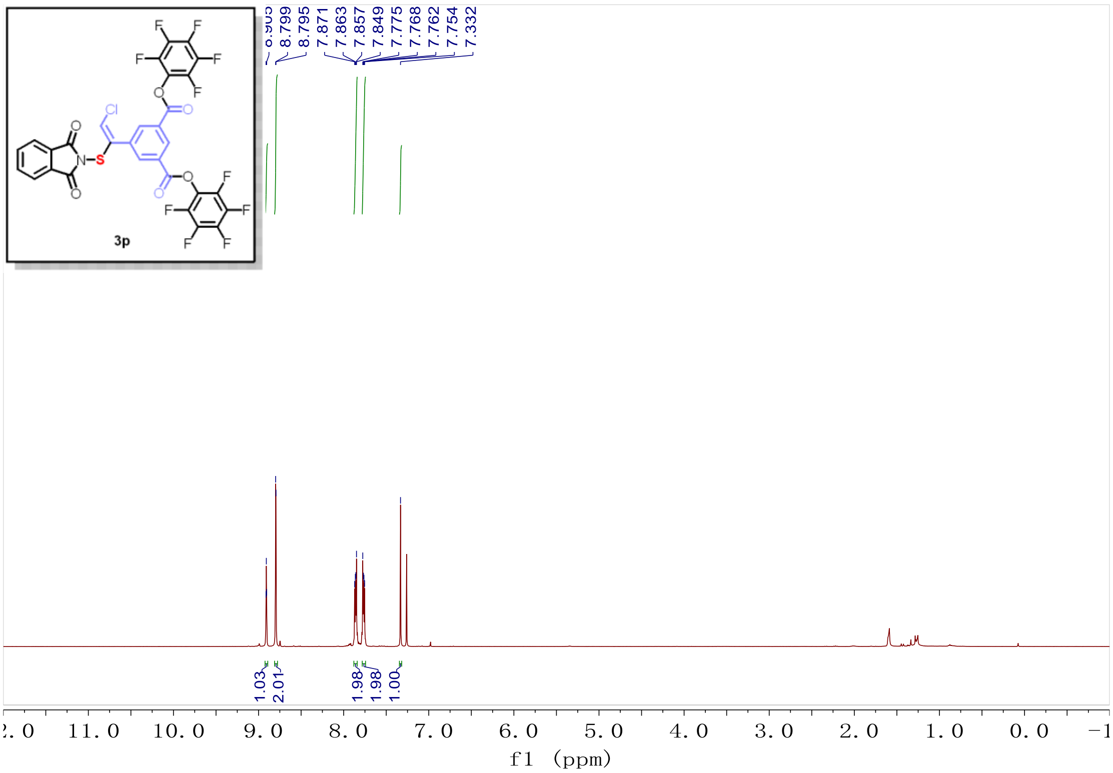

^{13}C NMR 125 MHz $CDCl_3$ of **3p**

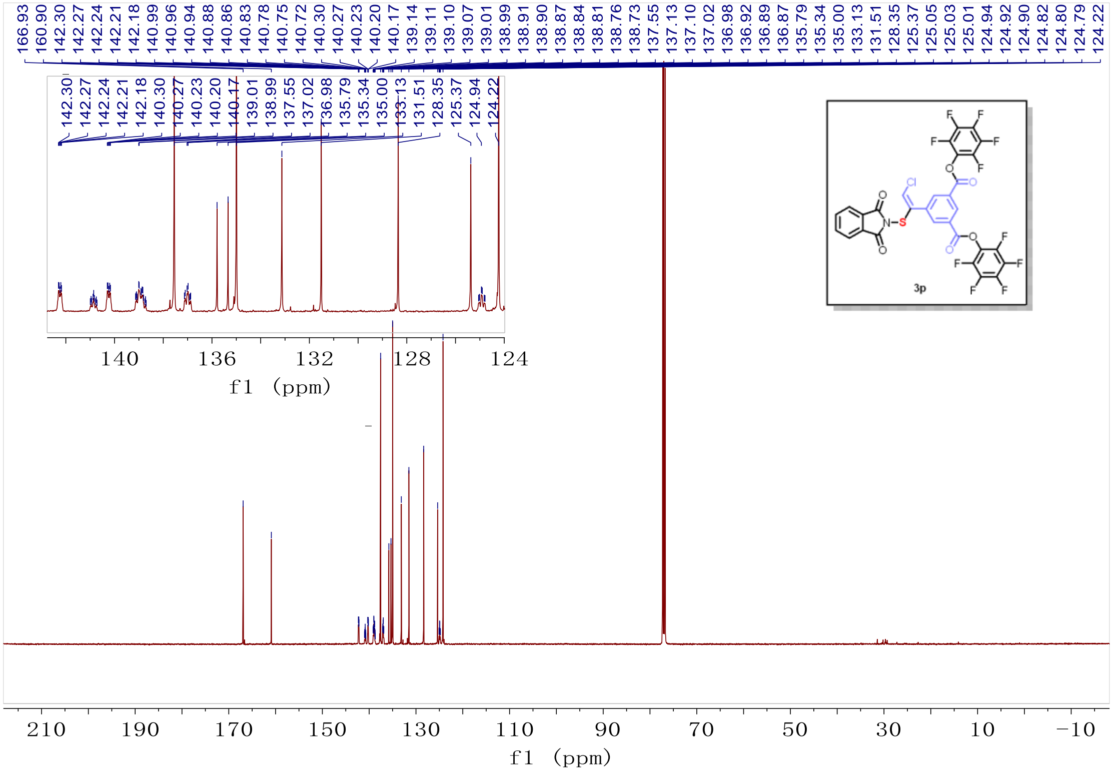

^{19}F NMR 282 MHz $CDCl_3$ of **3p**

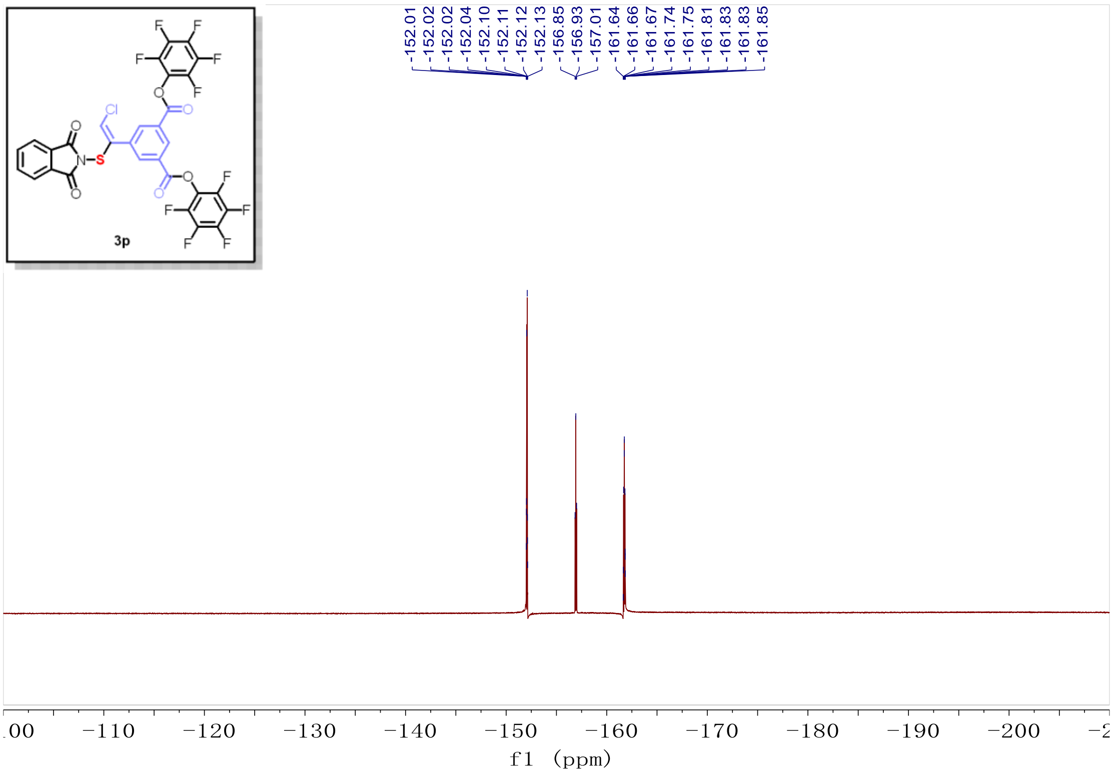

^{1}H NMR 400 MHz $CDCl_3$ of **3q**

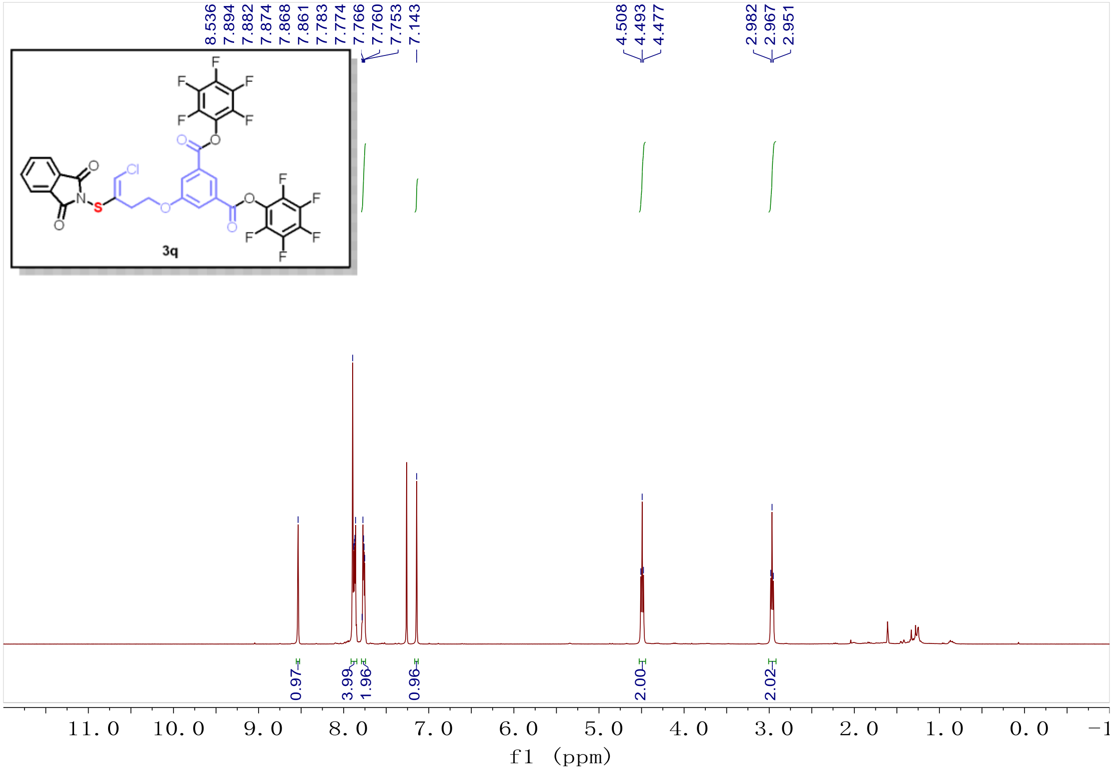

^{13}C NMR 125 MHz $CDCl_3$ of **3q**

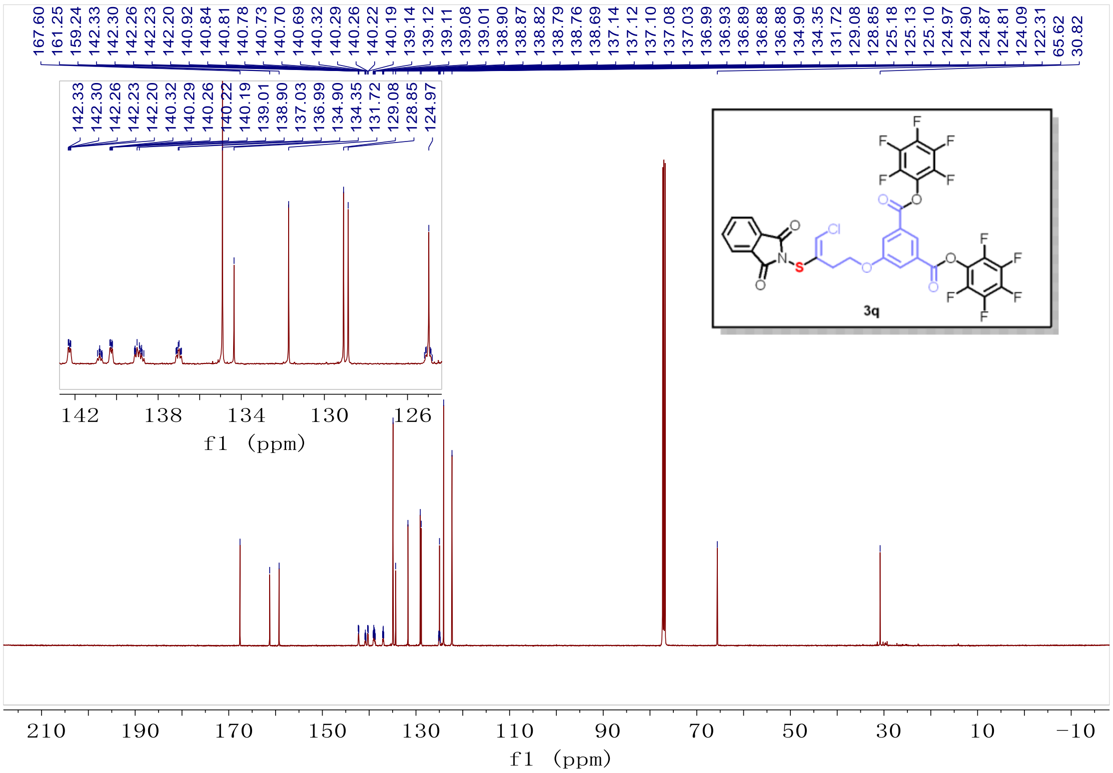

^{19}F NMR 282 MHz CDCl3 of **3q**

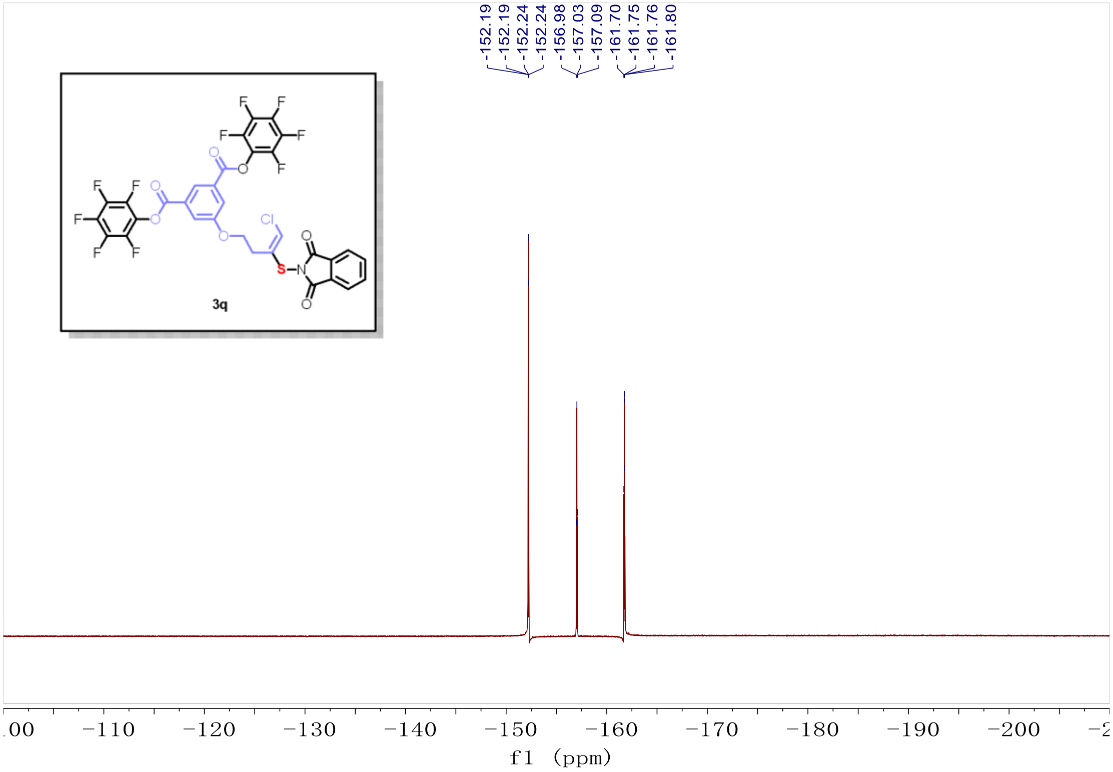

Supplement: nwaf406_Supplemental_Files [file nwaf406_supplemental_files.zip › Supporting_Information.pdf]
